# Addition of arylstannanes to alkynes giving *ortho*-alkenylarylstannanes catalysed cooperatively by a rhodium complex and zinc chloride†
†Electronic supplementary information (ESI) available: Experimental procedures, compound characterization data and X-ray crystallographic data of compound **3ag**. CCDC 1841177. For ESI and crystallographic data in CIF or other electronic format see DOI: 10.1039/c8sc02459f


**DOI:** 10.1039/c8sc02459f

**Published:** 2018-08-17

**Authors:** Jialin Ming, Qi Shi, Tamio Hayashi

**Affiliations:** a Division of Chemistry and Biological Chemistry , School of Physical and Mathematical Sciences , Nanyang Technological University , 21 Nanyang Link , Singapore 637371 , Singapore . Email: hayashi@ntu.edu.sg

## Abstract

The reaction of arylstannanes ArSnR_3_ with unfunctionalised alkynes was found to proceed in the presence of a rhodium catalyst and a catalytic amount of zinc chloride to give *ortho*-alkenylarylstannanes with high selectivity in high yields. The catalytic cycle is very unique, consisting of three transmetalation steps, from Sn to Rh, Rh to Zn, and Zn to Sn, in addition to arylrhodation of alkyne followed by 1,4-migration of Rh from 2-arylalkenyl carbon to *ortho*-alkenylaryl carbon.

## Abstract 2

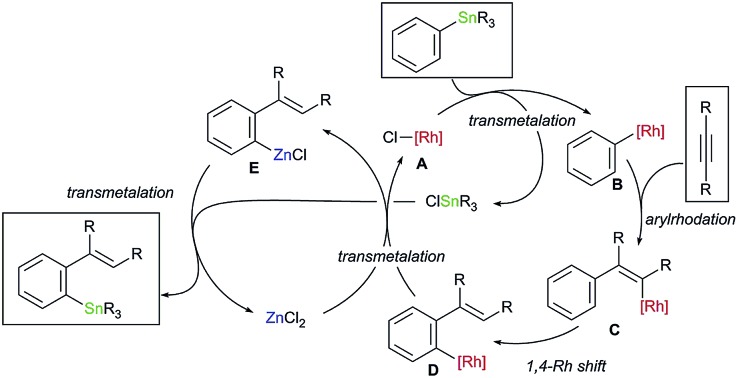
A Rh complex and ZnCl_2_ cooperatively catalyse the reaction of arylstannanes with alkynes giving *ortho*-alkenylarylstannanes.

## Introduction

**Scheme 1:**
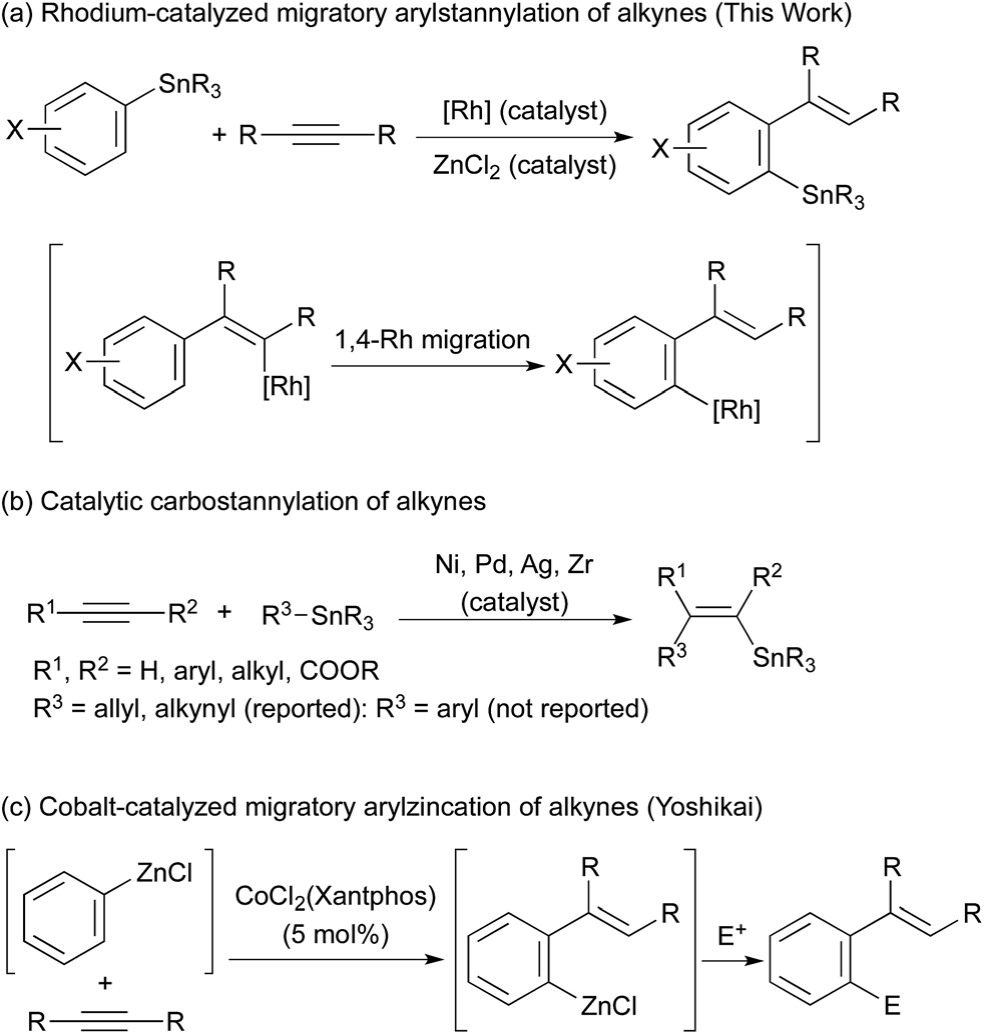
Arylstannylation of alkynes and 1,4-migration.

Organostannanes are organometallic reagents widely used for organic synthesis.^1^ They are easy to handle because of their inertness towards oxygen and moisture, while the organostannanes are reactive with various electrophiles in the presence of appropriate activators. A typical example is the palladium-catalysed cross-coupling reaction (Stille coupling).^2^ The organostannanes have been most commonly prepared by the reaction of highly reactive organometals (RLi, RMgX) with tin electrophiles, and considerable attention has been paid to the development of their new synthetic methods. Palladium- or nickel-catalysed stannylation of organic electrophiles with distannanes is one of the examples.^3^ Here we report our findings that migratory arylstannylation of unfunctionalised alkynes is catalysed by Rh complexes in the presence of a catalytic amount of ZnCl_2_ to give high yields of *ortho*-alkenylarylstannanes with high selectivity. The reaction is proposed to proceed through 1,4-migration of Rh from alkenyl carbon to aryl carbon^4^–^6^ (Scheme 1a). Carbostannylation of alkynes with allyl- and alkynylstannanes has been reported to be catalysed by transition metals such as Pd and Ni,^7^,^8^ but the addition of arylstannanes has not been reported, to the best of our knowledge (Scheme 1b). This type of arylmetalation reaction that is accompanied by the 1,4-migration of the metal has been reported by Yoshikai for arylzincation catalysed by a cobalt complex (Scheme 1c).^9^

## Results and discussion

The results obtained for the reaction of 4-octyne (**2a**) with PhSnBu_3_ (**1a**, 2.0 equiv. to **2a**) under various conditions are summarised in Table 1. The migratory arylstannylation was found to proceed in the presence of ZnCl_2_ (1.0 equiv. to **2a**) and a rhodium catalyst generated from [RhCl(coe)_2_]_2_ (5.0 mol% of Rh) and binap^10^ (5.5 mol%) in dioxane at 130 °C for 16 h to give 85% yield of 4-(2-stannylphenyl)-4-octene (**3aa**) with perfect *E* geometry^11^ (entry 1). The direct arylstannylation product **4aa** was not formed in a detectable amount under these reaction conditions. The yield of **3aa** was dependent on the amount of ZnCl_2_ to some extent. With 2.0 equiv. of ZnCl_2_, the yield was slightly increased (89%) (entry 2). The yields were lower with less amount of ZnCl_2_, being 73%, 71% and 27%, with 0.50, 0.25 and 0.10 equiv. of ZnCl_2_, respectively (entries 3–5). It is noted that ZnCl_2_ is working as a catalyst though a substoichiometric amount is necessary for a high yield of **3aa**. The presence of ZnCl_2_ is essential for the present arylstannylation, **3aa** being not formed at all in its absence (entry 6). At lower reaction temperature (100 °C), the yield of **3aa** was lower by 10% (entry 7). Other zinc halides, ZnBr_2_ and ZnI_2_ were less catalytically active than ZnCl_2_ (entries 8 and 9). It is difficult to find a substitute of ZnCl_2_ from other metal salts. CuCl^12^ gave a trace amount of **3aa** (entry 10). The binap ligand on Rh can be replaced by segphos or biphep, both of which are analogous to binap in that their backbones connecting two phosphino groups are biaryls. The yields with segphos^13^ and biphep were slightly lower (73% and 71%, respectively) than that with binap for the reaction of PhSnBu_3_ (**1a**) (entries 11 and 12), but for the reactions of some other arylstannanes, the yields of the arylstannylation products are higher with segphos ligand (see Table 2). The catalytic activity of other phosphine–Rh complexes were much lower. While dppf ligand gave a low yield (16%) of **3aa** (entry 13), the reaction did not take place with dppe, dppp, xantphos or PPh_3_ (entries 14–17). Rh complex with cyclooctadiene (cod) ligand or Ir/binap complex did not catalyse the reaction either (entries 18 and 19). The cobalt complex, CoCl_2_(xantphos), which has been reported to be an effective catalyst for the migratory arylzincation,^9^ is not a catalyst of choice for the present arylstannylation, yielding only 7% of **3aa** (entry 20).

**Table 1:** Rhodium-catalysed phenylstannylation of 4-octyne (**2a**) with PhSnBu_3_ (**1a**)^*a*^

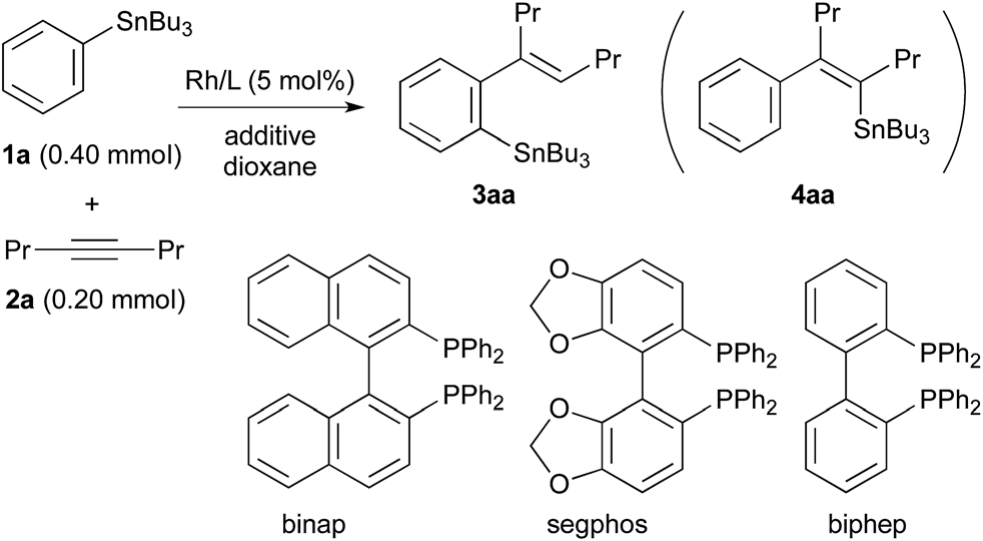			
Entry	Rh catalyst^*b*^ (5 mol%)	Additive (equiv. to **2a**)	Yield^*c*^ (%) of **3aa**
1	Rh/binap	ZnCl_2_ (1.0)	85
2	Rh/binap	ZnCl_2_ (2.0)	89
3	Rh/binap	ZnCl_2_ (0.50)	73
4	Rh/binap	ZnCl_2_ (0.25)	71
5	Rh/binap	ZnCl_2_ (0.10)	27
6	Rh/binap	—	0
7^*d*^	Rh/binap	ZnCl_2_ (1.0)	75
8	Rh/binap	ZnBr_2_ (1.0)	43
9	Rh/binap	ZnI_2_ (1.0)	18
10	Rh/binap	CuCl (1.0)	<3
11	Rh/segphos	ZnCl_2_ (1.0)	73
12	Rh/biphep	ZnCl_2_ (1.0)	71
13	Rh/dppf	ZnCl_2_ (1.0)	16
14	Rh/dppe	ZnCl_2_ (1.0)	0
15	Rh/dppp	ZnCl_2_ (1.0)	0
16	Rh/xantphos	ZnCl_2_ (1.0)	0
17	Rh/PPh_3_^*e*^	ZnCl_2_ (1.0)	<3
18	Rh/cod^*f*^	ZnCl_2_ (1.0)	0
19	Ir/binap^*g*^	ZnCl_2_ (1.0)	0
20	Co/xantphos^*h*^	ZnCl_2_ (1.0)	7

^*a*^Reaction conditions: 4-octyne (**2a**) (0.20 mmol), PhSnBu_3_ (**1a**) (0.40 mmol) and ZnCl_2_ (0.20 mmol) in dioxane (1.0 mL) at 130 °C (bath temp.) for 16 h.

^*b*^Rh catalyst (5 mol% of Rh) was generated *in situ* from [RhCl(coe)_2_]_2_ (10 μmol of Rh) and bisphosphine (11 μmol).

^*c*^Isolated yield.

^*d*^At 100 °C.

^*e*^RhCl(PPh_3_)_3_ (10 μmol).

^*f*^[RhCl(cod)]_2_ (10 μmol of Rh).

^*g*^[IrCl(coe)_2_]_2_ (10 μmol of Ir) + binap (11 μmol).

^*h*^CoCl_2_(xantphos) (10 μmol).

**Table 2:** Rhodium-catalysed arylstannylation of 4-octyne (**2a**) with ArSnR_3_**1**^*a*^

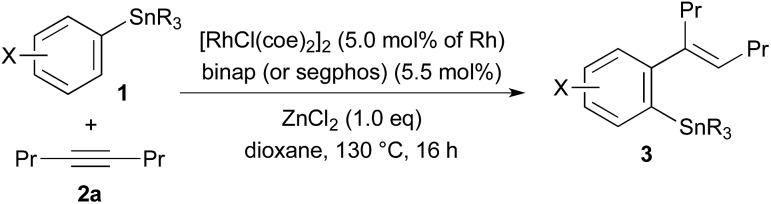			
Entry	ArSnR_3_**1**	L on Rh^*b*^	Yield^*c*^ (%) of **3**
1	PhSnBu_3_ (**1a**)	Binap	85 (**3aa**)
2	PhSnMe_3_ (**1b**)	Binap^*d*^	77 (**3ba**)
3	PhSnPr_3_ (**1c**)	Binap	89 (**3ca**)
4	PhSnOct_3_ (**1d**)	Binap	78 (**3da**)
5	4-MeC_6_H_4_SnBu_3_ (**1e**)	Binap	87 (**3ea**)
6	4-PhC_6_H_4_SnBu_3_ (**1f**)	Binap	91 (**3fa**)
7	4-Me_3_SiC_6_H_4_SnBu_3_ (**1g**)	Binap	83 (**3ga**)
8	4-CF_3_OC_6_H_4_SnBu_3_ (**1h**)	Binap	83 (**3ha**)
9	4-MeOC_6_H_4_SnBu_3_ (**1i**)	Binap^*d*^	65 (**3ia**)
10	4-FC_6_H_4_SnBu_3_ (**1j**)	Binap	77 (**3ja**)
11	4-ClC_6_H_4_SnBu_3_ (**1k**)	Binap	73 (**3ka**)
12	4-ClC_6_H_4_SnBu_3_ (**1k**)	Segphos	84 (**3ka**)
13	4-BrC_6_H_4_SnBu_3_ (**1l**)	Binap	47 (**3la**)
14	4-BrC_6_H_4_SnBu_3_ (**1l**)	Segphos	78 (**3la**)
15	4-NCC_6_H_4_SnBu_3_ (**1m**)	Binap	51 (**3ma**)
16	4-NCC_6_H_4_SnBu_3_ (**1m**)	Segphos	67 (**3ma**)
17	4-MeOOCC_6_H_4_SnBu_3_ (**1n**)	Binap	61 (**3na**)
18	4-MeOOCC_6_H_4_SnBu_3_ (**1n**)	Segphos	82 (**3na**)
19	4-CF_3_C_6_H_4_SnBu_3_ (**1o**)	Segphos	69 (**3oa**)
20	3-MeC_6_H_4_SnBu_3_ (**1p**)	Binap	77 (**3pa**)^*e*^
21	3-Me_3_SiC_6_H_4_SnBu_3_ (**1q**)	Binap	84 (**3qa**)^*e*^
22	3-CF_3_C_6_H_4_SnBu_3_ (**1r**)	Binap	67 (**3ra**)^*e*^
23	2-NaphthylSnBu_3_ (**1s**)	Binap	83 (**3sa**)^*e*^
24	2-MeC_6_H_4_SnBu_3_ (**1t**)	Binap	<3 (**3ta**)

^*a*^Reaction conditions: 4-octyne (**2a**) (0.20 mmol), ArSnR_3_**1** (0.40 mmol), ZnCl_2_ (0.20 mmol) and Rh catalyst (5 mol% of Rh) in dioxane (1.0 mL) at 130 °C (bath temp.) for 16 h.

^*b*^Rh catalyst (5 mol% of Rh) was generated *in situ* from [RhCl(coe)_2_]_2_ (10 μmol of Rh) and binap or segphos (11 μmol).

^*c*^Isolated yield.

^*d*^In THF at 90 °C.

^*e*^Regioselective 1,4-shift giving the products **3** shown below.
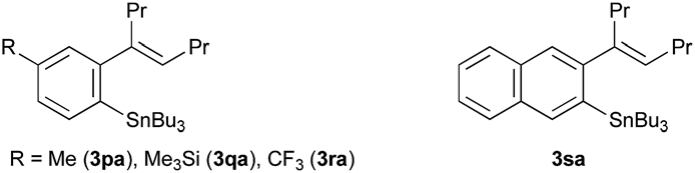

The reaction conditions optimised for the phenylstannylation with PhSnBu_3_ (**1a**), that is, with Rh/binap (5 mol%) and ZnCl_2_ (1.0 equiv.) at 130 °C (entry 1 in Table 1), were successfully applied to the reaction of several other aryltin reagents ArSnR_3_ with 4-octyne (**2a**) (Table 2). The phenyltin reagents PhSnR_3_, where R is methyl (**1b**), propyl (**1c**) and octyl (**1d**), all gave the corresponding phenylstannylation products **3ba–3da** in high yields (entries 2–4). The yields are generally high for *para*-substituted aryltin reagents, those substituted with Me, Ph, Me_3_Si and CF_3_O groups giving the corresponding products in 83–91% yields (entries 5–8). The lower yield (65%) for MeO-substituted one **1i** is mainly due to the instability of the product **3ia** under the reaction conditions (entry 9). For the reaction of aryltin reagents substituted with electron-withdrawing groups at *para*-position, Cl (**1k**), Br (**1l**), CN (**1m**) and COOMe (**1n**), the Rh/binap catalyst was not very effective resulting in lower yields of the corresponding arylstannylation products. The use of Rh/segphos as a catalyst instead of Rh/binap improved the reaction for these aryltin reagents (entries 11–18). A typical example is the reaction of 4-BrC_6_H_4_SnBu_3_, where the yields of the product **3la** are 47% and 78% with binap and segphos ligands, respectively (entries 13 and 14). In the arylstannylation with *meta*-substituted aryltin reagents, perfect regioselectivity at the 1,4-migration was observed. Thus, the reaction of those substituted with Me (**1p**), Me_3_Si (**1q**) and CF_3_ (**1r**) gave the corresponding 2,4-disubstituted aryltins **3pa–3ra**, which are the less hindered isomers, exclusively (entries 20–22). The regioselectivity was also high for the reaction of 2-naphthyltin **1s**, where the 1,4-migration took place to the less hindered 3-position selectively (entry 23). Unfortunately, the migratory arylstannylation did not take place for *ortho*-substituted tin reagent **1t** under the present conditions (entry 24).

The results obtained for the reaction of PhSnBu_3_ (**1a**) with several unfunctionalised alkynes substituted with alkyl and aryl groups are summarised in Table 3. The migratory arylstannylation proceeded well for longer-chained dialkylacetylenes, 5-decyne (**2b**) and 8-hexadecyne (**2c**), to give high yields of the corresponding products, **3ab** and **3ac**, respectively. In the reaction of unsymmetrically substituted dialkylacetylene **2d**, the regioselectivity at the addition to alkyne was low, resulting in the formation of a mixture of **3ad** and its regioisomer in a ratio of 1.2/1.0. Diarylacetylenes also underwent the migratory arylstannylation, although the yields are generally lower than those for dialkylacetylenes.^14^ The reaction of alkyl(aryl)alkynes **2h–2l**, proceeded with high regioselectivity for the bond formation between phenyl group of phenyltin **1a** and alkyl-substituted alkyne carbon. This selectivity is as expected from the reported regiochemistry at carbometalation of alkyl(aryl)alkynes.^15^

**Table 3:** Rhodium-catalysed arylstannylation of alkynes **2** with PhSnBu_3_ (**1a**)^*a*^

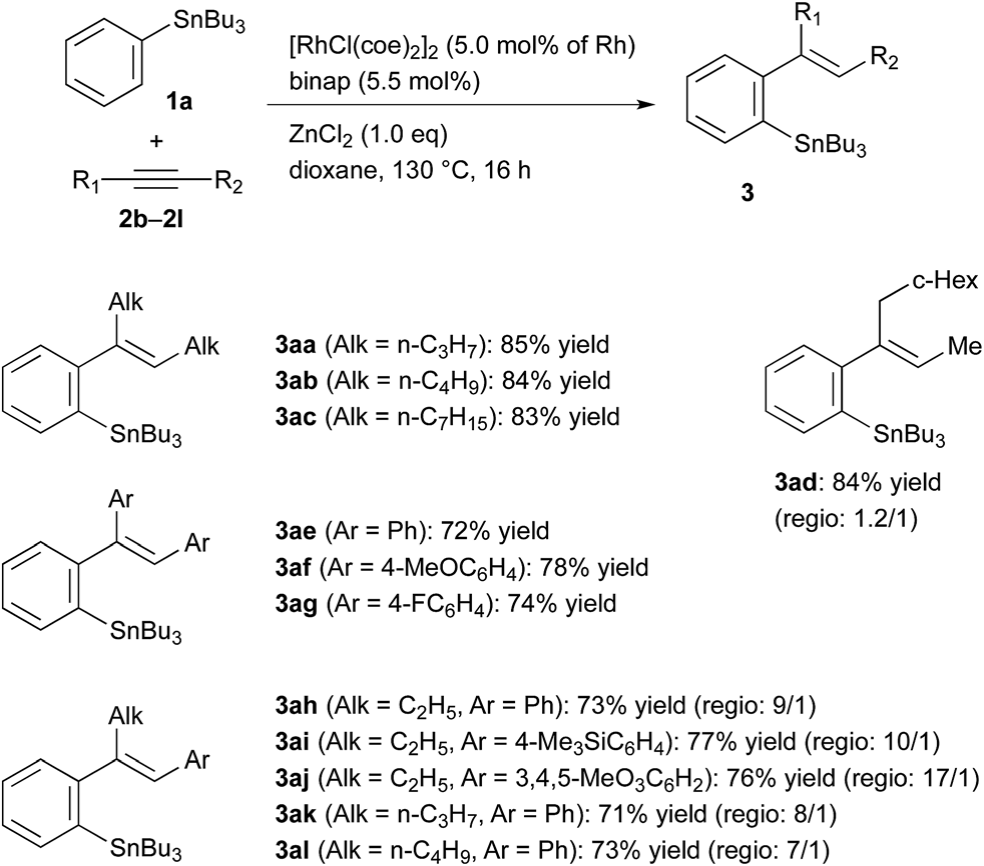

^*a*^Reaction conditions: alkyne **2** (0.20 mmol), PhSnBu_3_**1a** (0.40 mmol), ZnCl_2_ (0.20 mmol) and Rh catalyst (5 mol% of Rh), generated *in situ* from [RhCl(coe)_2_]_2_ (10 μmol of Rh) and binap (11 μmol), in dioxane (1.0 mL) at 130 °C (bath temp.) for 16 h. The structures of main regioisomers are shown for the products from unsymmetrically substituted alkynes.

**Scheme 2:**
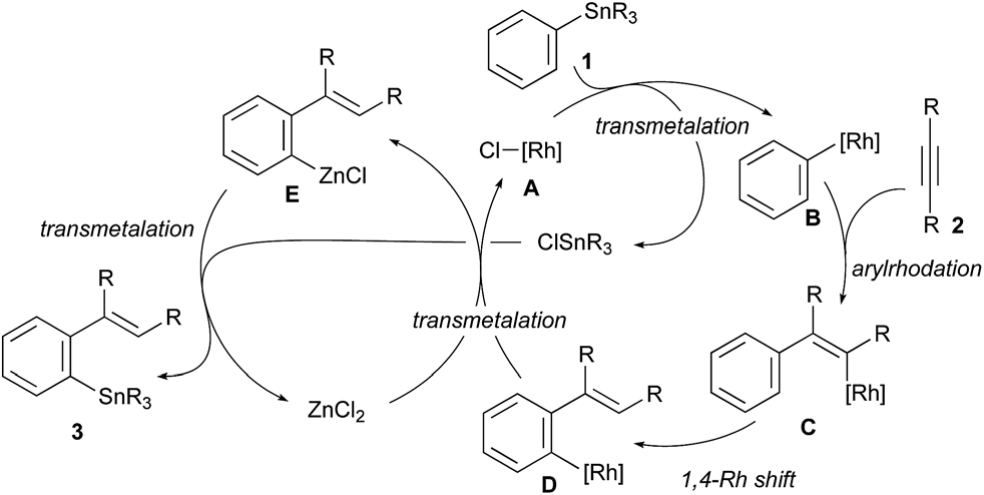
A catalytic cycle proposed for migratory arylstannylation of alkynes catalysed by Rh complex and ZnCl_2_.

The reaction pathway of the present migratory arylstannylation of alkynes, which is catalysed cooperatively by Rh complex and ZnCl_2_, is proposed as shown in Scheme 2. Thus, the transmetalation of phenyl group from Sn to Rh takes place at the reaction of PhSnR_3_**1** with a Cl–Rh species **A** to generate Ph–Rh intermediate **B** and ClSnR_3_,^16^ the latter being to be involved at the final step leading to the stannylation product **3**. The *syn*-addition of Ph–Rh **B** to alkyne **2** generates 2-arylalkenyl–Rh **C** and 1,4-migration of Rh from alkenyl to phenyl^4^,^5^ gives *ortho*-alkenylphenyl–Rh intermediate **D**, which has been reported to be thermodynamically more stable than **C**.5*e* Transmetalation between the *ortho*-alkenylpheny–Rh **D** and ZnCl_2_ takes place to give arylzinc chloride **E** and the Cl–Rh species **A**. Finally, the reaction of arylzinc chloride **E** with ClSnR_3_,^17^ which was formed at the transmetalation between PhSnR_3_**1** and Cl–Rh **A**, leads to *ortho*-alkenylphenystannane **3**, with regenerating ZnCl_2_. Direct transmetalation between the Ar–Rh intermediate **D** and PhSnR_3_**1** giving Ph–Rh **B** and the product **3** is less likely because a catalytic amount of ZnCl_2_ is essential for the present arylstannylation to take place (see entries 1–6 in Table 1).

**Scheme 3:**
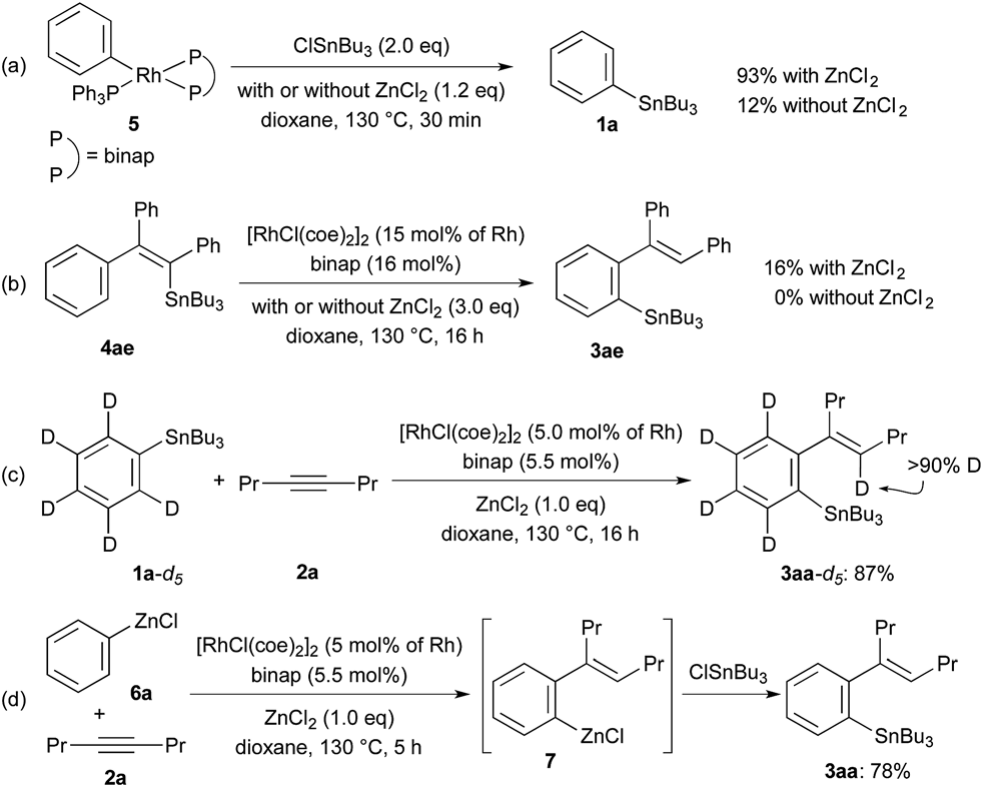
Reactions to support the catalytic cycle.

The reactions shown in Scheme 3 gave us further information on the catalytic cycle. Stoichiometric reactions of Ph–Rh complex, RhPh(PPh_3_) (binap) (**5**),^18^ with ClSnBu_3_ (2.0 equiv.) in the presence of ZnCl_2_ (1.2 equiv.) in dioxane at 130 °C gave 93% yield of PhSnBu_3_ (**1a**), while the yield of **1a** is very low (12%) in the absence of ZnCl_2_ under otherwise the same conditions (Scheme 3a). These reactions are related to the last transmetalation step producing **3** from Ar–Rh intermediate **D** in the catalytic cycle. The results show that the direct transmetalation between intermediate **D** and ClSnR_3_ is slow and that ZnCl_2_ greatly accelerates the transmetalation. The fast transmetalation in the presence of ZnCl_2_ is probably because of a lower energy caused by the double transmetalations from Rh to Zn and from Zn to Sn by way of arylzinc species **E**. Rhodium-catalysed 1,4-migration of Sn from alkenylstannane **4ae** to arylstannane **3ae** was observed in the presence of ZnCl_2_, albeit in a low yield (16%).^19^ The 1,4-migration of Sn did not take place in the absence of ZnCl_2_ (Scheme 3b). The catalytic cycle involving the 1,4-Rh shift from intermediates **C** to **D** is supported by these results. The deuterium-labeling study using C_6_D_5_SnBu_3_ (**1a**-d_5_) (Scheme 3c), where the deuterium is incorporated at olefinic carbon in **3aa**-d_5_, further supports this catalytic cycle involving the 1,4-Rh shift. The present Rh/ZnCl_2_ cocatalyst system is also applicable to the addition of PhZnCl (**6a**) to 4-octyne (**2a**) generating *ortho*-alkenylphenylzinc species **7**,^20^ the reaction of which with ClSnBu_3_ gave 78% yield of the tin compound **3aa** (Scheme 3d).

**Scheme 4:**
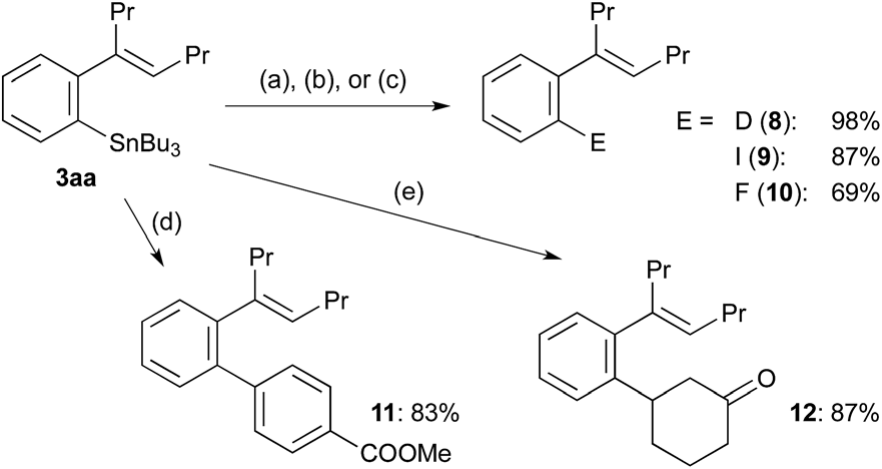
Transformation of *ortho*-alkenylarylstannane **3aa**. (a) (CF_3_CO)_2_O, D_2_O. (b) I_2_, CH_2_Cl_2_. (c) Selectfluor, AgOTf, acetone. (d) 4-IC_6_H_4_COOMe, PdCl_2_(PPh_3_)_2_ (10 mol%), CuI, DMF. (e) 2-Cyclohexenone, [RhCl(cod)]_2_ (5 mol% Rh), KOH, dioxane/H_2_O.

The synthetic utility of arylstannanes has been well established.^1^ According to the reported procedures,^21^–^23^ tributylstannyl group in **3aa** was converted into deuterio (**8**), iodo (**9**) and fluoro (**10**) successfully (Scheme 4). The palladium-catalysed cross-coupling with an aryl iodide^2^ and the rhodium-catalysed conjugate addition to 2-cyclohexenone^16^ gave high yields of the corresponding products, **11** and **12**, respectively, as expected.

## Conclusions

To summarise, migratory arylstannylation was found to take place in the reaction of arylstannanes ArSnR_3_ with unfunctionalised alkynes in the presence of a bisphosphine–rhodium catalyst and a catalytic amount of zinc chloride to produce *ortho*-alkenylarylstannanes in high yields. A catalytic cycle involving three transmetalation steps is proposed. That is, transmetalation of aryl groups from Sn to Rh, Rh to Zn, and Zn to Sn.

## Conflicts of interest

There are no conflicts to declare.

## Supplementary Material

Supplementary information

Crystal structure data
