# Supplementary material for: Addition of arylstannanes to alkynes giving ortho-alkenylarylstannanes catalysed cooperatively by a rhodium complex and zinc chloride
Source: Chem Sci. 2018 Aug 17;9(39):7700–4. doi: 10.1039/c8sc02459f (PMC6182567; doi:10.1039/c8sc02459f)
Supplement: Supplementary file 1 [file SC-009-C8SC02459F-s001.pdf]

Electronic Supplementary Material (ESI) for Chemical Science.
This journal is © The Royal Society of Chemistry 2018

Supporting Information

Contents:

1. General method S-2
2. Materials S-2
3. Prepartion of trioctyl(phenyl)stannane (**1d**) [143363-50-2] S-3
4. Prepartion of tributyl(4-(trifluoromethoxy)phenyl)stannane (**1h**) and tributyl(pentadeutero-phenyl)stannane (**1a**-d_5) S-3
5. Preparation of 1-(4-trimethylsilylphenyl)but-1-yne (**2i**) and 1-(3,4,5-trimethoxyphenyl)but-1-yne (**2j**) S-4
6. A typical procedure for migratory arylstannylation of alkynes catalysed by rhodium complex and zinc chloride (Table 1, entry 1) S-5
7. Reactions to support the catalytic cycle (Scheme 3) S-6
8. Transformations of (*E*)-4-(2-tributylstannylphenyl)oct-4-ene (**3aa**) (Scheme 4) S-9
9. Characterization of the products S-11
10. Single crystal X-ray diffraction data for compound **3ag** (CCDC 1841177) S-26
11. References S-28
12. NMR spectra S-29

1. General method

All anaerobic and moisture-sensitive manipulations were carried out with standard Schlenk techniques under dry nitrogen gas. NMR spectra were recorded on a Bruker AV 500MHz NMR, BBI probe (500 MHz for ^{1}H, 125 MHz for ^{13}C), Bruker AV 400MHz NMR, QNP probe (400 MHz for ^{1}H, 100 MHz for ^{13}C, 61 MHz for ^{2}H), Bruker AVIII 400MHz NMR, BBFO Probe (400 MHz for ^{1}H, 100 MHz for ^{13}C), JEOL ECA 400 MHz NMR (400 MHz for ^{1}H, 100 MHz for ^{13}C), or Bruker AV 300MHz NMR, QNP probe (300 MHz for ^{1}H, 75 MHz for ^{13}C). Chemical shifts are reported in δ (ppm) referenced to the residual peaks of $CDCl_3$ (δ 7.26) for ^{1}H NMR, $CDCl_3$ (δ 77.00) for ^{13}C NMR and acetone (δ 2.05) for ^{2}H NMR. The following abbreviations are used to describe the multiplicities; s: singlet, d: doublet, t: triplet, q: quartet, quint: quintet, sext: sextet, m: multiplet, br: broad. High resolution mass spectra (HRMS) were obtained with a Waters Q-Tof Premier mass spectrometer. For thin layer chromatography (TLC), Merck pre-coated TLC plates (Merck 60 F254) were used, and compounds were visualized with a UV light at 254 nm. Further visualization was achieved by basic aqueous $KMnO_4$ solution stain. Flash column chromatography was performed with Silica gel 60 (Merk). The products were further purified by GPC (Gel Permeation Chromatography) if necessary.

2. Materials

Alkynes (**2a**–**2c**, **2e**–**2h**, **2k** and **2l**), aryl halides, bisphosphine ligands, n-BuLi, D_2O, CuI, CuCl, AgOTf, KOH, Mg, $ZnCl_2$, $ZnBr_2$, ZnI_2, cyclohex-2-enone, trifluoroacetic anhydride, tributyltin chloride, $PhSnCl_3$, $PhSnBu_3$ (**1a**), $PhSnMe_3$ (**1b**), 4-$MeOC_6H_4SnBu_3$ (**1i**), 4-$FC_6H_4SnBu_3$ (**1j**), 4-$ClC_6H_4SnBu_3$ (**1k**), 1-chloromethyl-4-fluoro-1,4-diazoniabicyclo[2.2.2]octane bis(tetrafluoroborate), 1-bromooctane, 1-bromopropane, 2-bromo-1,1,2-triphenylethylene, $[IrCl(coe)_2]_2$, $CoCl_2$(xantphos), $PdCl_2(PPh_3)_2$, $RhCl(PPh_3)_3$ and iodine were purchased and used as received. Dioxane and THF were distilled over benzophenone ketyl under N_2.

$[RhCl(coe)_2]_2$,[1] $[RhCl(cod)]_2$,[2] RhPh(binap)(PPh_3) (**5**) [434314-09-7],[3] 4-$BrC_6H_4SnBu_3$ (**1l**) [17151-49-4],[4] 4-$NCC_6H_4SnBu_3$ (**1m**) [79048-30-9],[4] 4-$MeOOCC_6H_4SnBu_3$ (**1n**) [91734-76-8],[5] and but-2-yn-1-ylcyclohexane (**2d**) [57497-07-1][6] were prepared according to the reported procedures.

3. Preparation of trioctyl(phenyl)stannane (1d) [143363-50-2]

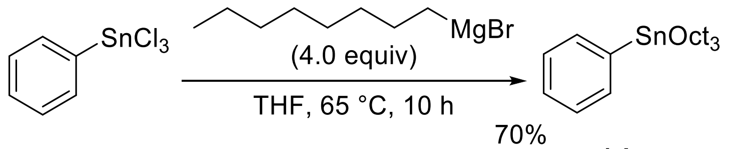

1d

To a solution of octylmagnesium bromide in THF (25.0 mL), prepared from magnesium turnings (593 mg, 24 mmol) and 1-bromooctane (3.86 g, 20.0 mmol), $PhSnCl_3$ (1.51 g, 5.0 mmol) was added dropwise over 15 min at room temperature. The mixture was stirred at 65 °C for 10 h before H_2O (10.0 mL) was added. The mixture was extracted with ethyl acetate, and the combined organic layer was dried over $MgSO_4$ and concentrated under reduced pressure. The residue was subjected to chromatography on silica gel (pre-treated with 1% Et_3N in hexanes) to give trioctyl(phenyl)stannane (**1d**) [143363-50-2] (70%, 1.87 g, 3.5 mmol) as colorless oil. The spectral data are in agreement with reported literature values.[7]

$PhSnPr_3$ (**1c**) [55335-05-2] was prepared in the same manner as above using propylmagnesium bromide, and the spectral data are in agreement with reported literature values.[8]

4. Preparation of tributyl(4-(trifluoromethoxy)phenyl)stannane (1h) and tributyl(penta-deuterophenyl)stannane (1a-*d*$_5$)

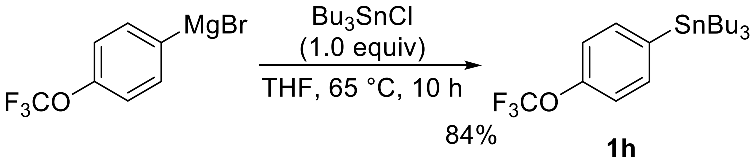

1h

To a solution of 4-(trifluoromethoxy)phenylmagnesium bromide in THF (10.0 mL), prepared from magnesium turnings (288 mg, 12 mmol) and 4-bromotrifluoromethoxybenzene (2.41 g, 10.0 mmol), Bu_3SnCl (3.26 g, 10.0 mmol) was added dropwise over 15 min at room temperature. The mixture was stirred at 65 °C for 10 h before H_2O (5.0 mL) was added. The mixture was extracted with ethyl acetate, and the combined organic layer was dried over $MgSO_4$ and concentrated under reduced pressure. The residue was subjected to chromatography on silica gel (pre-treated with 1% Et_3N in hexanes) to give tributyl(4-(trifluoromethoxy)phenyl)stannane (**1h**) (84%, 3.79 g, 8.4 mmol) as colorless oil. R_f = 0.7 (hexanes). **^{1}H NMR** (400 MHz, $CDCl_3$) δ 0.92 (t, $J_{H,H}$ = 7.3 Hz, 9H), 1.09 (t, $J_{H,H}$ = 8.1 Hz, $J_{H,^{119}Sn}$ = 51.2 Hz, 6H), 1.36 (sext, $J_{H,H}$ = 7.3 Hz, 6H), 1.45–1.65 (m, 6H), 7.19 (d, $J_{H,H}$ = 7.6 Hz, 2H), 7.50 (d, $J_{H,H}$ = 8.1 Hz, $J_{H,^{119}Sn}$ = 35.7 Hz, 2H); **^{13}C NMR** (100 MHz, $CDCl_3$) δ 9.7 ($J_{C,^{119}Sn}$ = 343.4 Hz, $J_{C,^{117}Sn}$ = 328.2 Hz), 13.6, 27.4 ($J_{C,^{119}Sn}$ = 55.9 Hz), 29.0 ($J_{C,^{119}Sn}$ = 20.3 Hz), 120.3 ($J_{C,^{119}Sn}$ = 41.7 Hz), 120.6 (q, $J_{C,^{19}F}$ = 256.8

Hz), 137.6 ($J_{C,^{119}Sn}$ = 33.5 Hz), 140.6 ($J_{C,^{119}Sn}$ = 368.7 Hz, $J_{C,^{117}Sn}$ = 352.7 Hz), 149.5 (q, $J_{C,^{19}F}$ = 1.4 Hz). **HRMS** (ESI) calcd for $C_{19}H_{31}OF_3Na^{120}Sn$ $[M+Na]^+$ 475.1247, found 475.1272.

$C_6D_5SnBu_3$ (**1a**-*d*$_5$) was prepared in the same manner as above using pentadeutero-phenylmagnesium bromide and Bu_3SnCl. Thus, the reaction of a solution of pentadeuterophenyl-magnesium bromides in THF (10.0 mL), prepared from magnesium turnings (299 mg, 12 mmol) and pentadeuterophenyl bromide (1.62g, 10.0 mmol), with Bu_3SnCl (3.26 g, 10.0 mmol) at 65 °C for 10 h gave tributyl(pentadeuterophenyl)stannane (**1a**-*d*$_5$) (83% yield, 3.09 g, 8.3 mmol) as colorless oil. R_f = 0.9 (hexanes). **^{1}H NMR** (300 MHz, $CDCl_3$) δ 0.93 (t, $J_{H,H}$ = 7.3 Hz, 9H), 1.10 (t, $J_{H,H}$ = 8.1 Hz, $J_{H,^{119}Sn}$ = 51.1 Hz, 6H), 1.37 (sext, $J_{H,H}$ = 7.3 Hz, 6H), 1.44–1.75 (m, 6H); **^{13}C NMR** (75 MHz, $CDCl_3$) δ 9.5 ($J_{C,^{119}Sn}$ = 339.6 Hz, $J_{C,^{117}Sn}$ = 324.6 Hz), 13.7, 27.4 ($J_{C,^{119}Sn}$ = 56.6 Hz), 29.1 ($J_{C,^{119}Sn}$ = 20.1 Hz), 127.4 (t, $J_{C,^2H}$ = 24.1 Hz), 136.0 (t, $J_{C,^2H}$ = 24.0 Hz), 141.7. **HRMS** (ESI) calcd for $C_{18}H_{27}D_5Na^{120}Sn$ $[M+Na]^+$ 396.1738, found 396.1733.

4-$MeC_6H_4SnBu_3$ (**1e**) [31614-66-1],[7] 4-$PhC_6H_4SnBu_3$ (**1f**) [51533-89-2],[4] 4-$Me_3SiC_6H_4SnBu_3$ (**1g**) [38860-01-4],[9] 4-$CF_3C_6H_4SnBu_3$ (**1o**) [86487-19-6],[5] 3-$MeC_6H_4SnBu_3$ (**1p**) [68971-88-0],[5] 3-$Me_3SiC_6H_4SnBu_3$ (**1q**) [1026787-60-9],[10] 3-$CF_3C_6H_4SnBu_3$ (**1r**) [53566-38-4],[11] 2-naphthyl$SnBu_3$ (**1s**) [972-11-2],[11] and 2-$MeC_6H_4SnBu_3$ (**1t**) [68971-87-9][5] were prepared in the same manner as above from the corresponding arylmagnesium bromides and Bu_3SnCl. Their spectral data are in agreement with reported literature values.

5. Preparation of 1-(4-trimethylsilylphenyl)but-1-yne (2i) and 1-(3,4,5-trimethoxyphenyl)but-1-yne (2j)

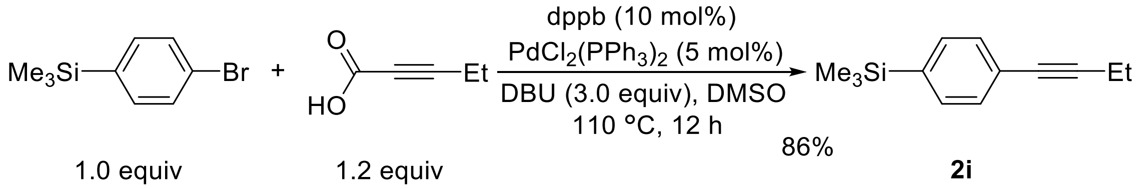

$PdCl_2(PPh_3)_2$ (351.0 mg, 0.50 mmol, 5.0 mol%) and 1,4-bis(diphenylphosphino)butane (426.5 mg, 1.0 mmol, 10.0 mol%) were placed in a 100 mL flask under nitrogen. DMSO (25 mL) was added and the mixture was stirred at room temperature for 10 min. To the mixure, 1-bromo-4-(trimethylsilyl)benzene (2.29 g, 10.0 mmol), 2-pentynoic acid (1.18 g, 12.0 mmol) and DBU (4.58 g, 30.0 mmol) were added. The mixture was heated at 110 °C for 12 h. The mixture was diluted with 20.0 mL of diethyl ether, and it was passed through a short pad of silica gel with ethyl acetate as an eluent. The solution was washed with H_2O and brine, dried over anhydrous $MgSO_4$, and concentrated under reduced pressure. The residue was subjected to chromatography on silica gel to give 1-(4-trimethylsilylphenyl)but-1-yne (**2i**) (86%, 1.74 g, 8.6 mmol) as pale yellow oil. R_f = 0.7 (hexanes). **^{1}H NMR** (400 MHz, $CDCl_3$) δ 0.25 (s, 9H), 1.24 (t, $J_{H,H}$ = 7.5 Hz, 3H), 2.42 (q, $J_{H,H}$ = 7.5 Hz, 2H), 7.36 (d,

$J_{H,H}$ = 8.1 Hz, 2H), 7.43 (d, $J_{H,H}$ = 8.1 Hz, 2H); **^{13}C NMR** (100 MHz, $CDCl_3$) δ –1.2, 13.1, 13.9, 80.0, 92.1, 124.3, 130.6, 133.1, 139.9. **HRMS** (ESI) calcd for $C_{13}H_{19}Si$ $[M+H]^+$ 203.1256, found 203.1218.

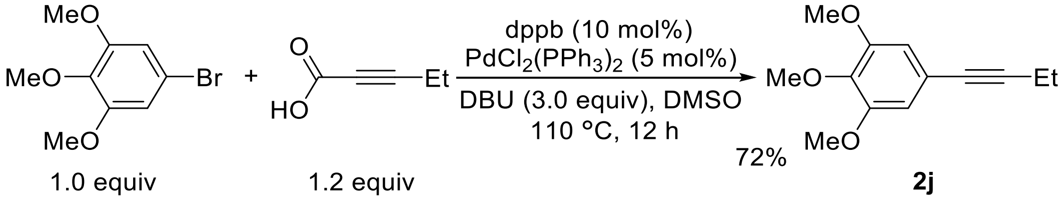

The alkyne, 1-(3,4,5-trimethoxyphenyl)but-1-yne (**2j**), was prepared in the same manner as above using 5-bromo-1,2,3-trimethoxybenzene. Thus, the reaction of $PdCl_2(PPh_3)_2$ (351.0 mg, 0.50 mmol, 5.0 mol%), 1,4-bis(diphenylphosphino)butane (426.5 mg, 1.0 mmol, 10.0 mol%), 5-bromo-1,2,3-trimethoxybenzene (2.47 g, 10.0 mmol), 2-pentynoic acid (1.18 g, 12.0 mmol) and DBU (4.58 g, 30.0 mmol) at 110 °C for 12 h gave **2j** (72% yield, 1.59 g, 7.2 mmol) as colorless solid. R_f= 0.1 (hexanes). **^{1}H NMR** (400 MHz, $CDCl_3$) δ 1.24 (t, $J_{H,H}$ = 7.5 Hz, 3H), 2.41 (q, $J_{H,H}$ = 7.5 Hz, 2H), 3.83 (s, 3H), 3.84 (s, 6H), 6.63 (s, 2H); **^{13}C NMR** (100 MHz, $CDCl_3$) δ 12.9, 13.8, 55.9, 60.8, 79.7, 90.6, 108.6, 119.0, 138.1, 152.8. **HRMS** (ESI) calcd for $C_{13}H_{17}O_3$ $[M+H]^+$ 221.1178, found 221.1190.

6. A typical procedure for migratory arylstannylation of alkynes catalysed by rhodium complex and zinc chloride (Table 1, entry 1)

$[RhCl(coe)_2]_2$ (3.58 mg, 0.0050 mmol, 5.0 mol% of Rh), binap (6.85 mg, 0.0110 mmol, 5.5 mol%), $ZnCl_2$ (27.3 mg, 0.20 mmol) and $PhSnBu_3$ (**1a**) (146.9 mg, 0.40 mmol) were placed in a 10 mL Schlenk tube under nitrogen. Dioxane (1.0 mL) was added and the mixture was stirred at room temperature for 10 min. Before the tube was sealed, 4-octyne (**2a**) (22.0 mg, 0.20 mmol) was added. The tube was placed in a preheated oil bath at 130 °C and the mixture was stirred for 16 h. The reaction mixture was passed through a short pad of basic aluminum oxide with ethyl acetate as the eluent. The solvent was removed on a rotary evaporator. The residue was subjected to chromatography on silica gel (pre-treated with 1% Et_3N in hexanes) to give (*E*)-4-(2-tributylstannylphenyl)oct-4-ene (**3aa**) (85%, 81.2 mg, 0.17 mmol) as colorless oil.

The procedures shown above in Section 6 for the reaction of $PhSnBu_3$ (**1a**) with 4-octyne (**2a**) (Table 1, entry 1) were used for the reaction of $ArSnR_3$ (**1c**–**1h**, **1j**–**1n** and **1p**–**1t**) with 4-octyne (**2a**) (Table 2, entries 3–8, 10, 11, 13, 15, 17 and 20–24), and that of $PhSnBu_3$ (**1a**) with unfunctionalised alkynes (**2b**–**2l**) (Table 3).

In the reaction of $ArSnR_3$ (**1k**–**1o**) with 4-octyne (**2a**) (Table 2, entries 12, 14, 16, 18 and 19), the procedures shown in Section 6 were modified by use of segphos instead of binap.

In Table 2 entry 2, the procedures shown in Section 6 were modified by using THF as solvent and decreasing the reaction temperature to 90 °C. Thus, the reaction of $[RhCl(coe)_2]_2$ (3.58 mg, 0.0050 mmol,

5.0 mol% of Rh), binap (6.85 mg, 0.0110 mmol, 5.5 mol%), $ZnCl_2$ (27.3 mg, 0.20 mmol), $PhSnMe_3$ (**1b**) (96.4 mg, 0.40 mmol) and 4-octyne (**2a**, 22.0 mg, 0.20 mmol) in THF (1.0 mL) at 90 °C for 16 h gave (*E*)-4-(2-trimethylstannylphenyl)oct-4-ene (**3ba**) (77% yield, 54.1 mg, 0.15 mmol) as colorless oil. The same procedures for the reaction of $PhSnMe_3$ (**1b**) with 4-octyne (**2a**) were also applied to the reaction of 4-$MeOC_6H_4SnBu_3$ (**1i**) with 4-octyne (**2a**) (Table 2, entry 9).

7 Reactions to support the catalytic cycle (Scheme 3)

Scheme 3a

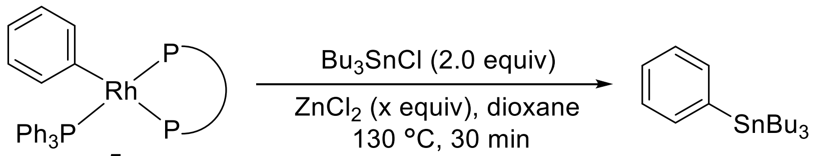

5 **1a**

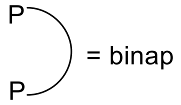

entry	x	yield of 1a
1	1.2	93%
2	0	12%

Scheme 3a, entry 1: In an oven-dried Schlenk tube, $RhPh(PPh_3)$(binap) (**5**) (106.0 mg, 0.10 mmol) and $ZnCl_2$ (16.4 mg, 0.12 mmol) were placed under nitrogen. Dioxane (1.0 mL) and Bu_3SnCl (65.3 mg, 0.20 mmol) were added and the mixture was heated at 130 °C for 30 min. The reaction was quenched with saturated aqueous solution of Na_2CO_3 (1.0 mL). The mixture was extracted with diethyl ether, and the combined organic layer was dried over $MgSO_4$ and concentrated under reduced pressure. The residue was subjected to short chromatography on silica gel (pre-treated with 1% Et_3N in hexanes) to give tributyl(phenyl)stannane (**1a**) (93%, 34.5 mg, 0.09 mmol) as colorless oil. R_f= 0.9 (hexanes). The spectral data are in agreement with reported literature values.[10b]

Scheme 3a, entry 2: The procedures for entry 1 in Scheme 3a were modified by removing $ZnCl_2$. The reaction of $RhPh(PPh_3)$(binap) (107.1 mg, 0.10 mmol) and Bu_3SnCl (66.0 mg, 0.20 mmol) in dioxane (1.0 mL) at 130 °C for 30 min gave tributyl(phenyl)stannane (**1a**) (12%, 4.5 mg, 0.01 mmol) as colorless oil.

Scheme 3b

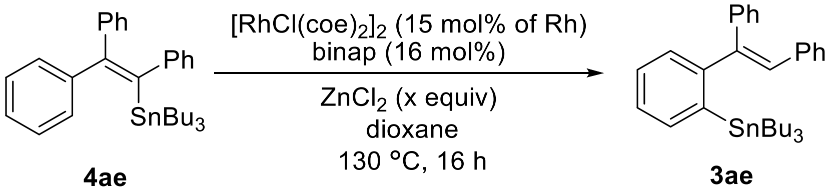

4ae **3ae**

entry	x	yield of 3ae
1	3	16%
2	0	0%

Preparation of compound 4ae: To a solution of 2-bromo-1,1,2-triphenylethylene (3.35 g, 10.0 mmol) in diethyl ether (20.0 mL), n-BuLi (5.0 mL, 10.0 mmol, 2.0 M in cyclohexane) was added dropwise at –78 °C over 30 min. The mixture was allowed to warm to 0 °C and stirred for 2 h. Bu_3SnCl (3.30 g, 10.1 mmol) was added to the mixture at 0 °C. The mixture was stirred at room temperature for 12 h before H_2O (5.0 mL) was added. The mixture was extracted with ethyl acetate, and the combined organic layer was dried over $MgSO_4$ and concentrated under reduced pressure. The residue was subjected to chromatography on silica gel (pre-treated with 1% Et_3N in hexanes) to give **4ae** (88%, 4.78 g, 8.8 mmol) as pale yellow oil. R_f = 0.8 (hexanes). **^{1}H NMR** (400 MHz, $CDCl_3$) δ 0.53 (t, $J_{H,H}$ = 8.2 Hz, $J_{H,^{119}Sn}$ = 50.8 Hz, 6H), 0.80 (t, $J_{H,H}$ = 7.2 Hz, 9H), 1.16 (sext, $J_{H,H}$ = 7.2 Hz, 6H), 1.21–1.32 (m, 6H), 6.87–7.03 (m, 8H), 7.12 (t, $J_{H,H}$ = 7.6 Hz, 2H), 7.28–7.35 (m, 5H); **^{13}C NMR** (100 MHz, $CDCl_3$) δ 11.6 ($J_{C,^{119}Sn}$ = 334.2 Hz, $J_{C,^{117}Sn}$ = 319.6 Hz), 13.6, 27.3 ($J_{C,^{119}Sn}$ = 61.6 Hz), 29.0 ($J_{C,^{119}Sn}$ = 19.3 Hz), 124.4, 125.9, 127.1, 127.2, 127.6, 128.0 ($J_{C,^{119}Sn}$ = 15.6 Hz), 128.1, 129.4, 130.0, 142.5 ($J_{C,^{119}Sn}$ = 49.2 Hz), 145.9 ($J_{C,^{119}Sn}$ = 26.0 Hz), 146.2 ($J_{C,^{119}Sn}$ = 25.4 Hz), 148.3, 152.7 ($J_{C,^{119}Sn}$ = 24.7 Hz). **HRMS** (ESI) calcd for $C_{32}H_{42}Na^{120}Sn$ $[M+Na]^+$ 569.2206, found 569.2206.

Scheme 3b, entry 1: In an oven-dried Schlenk tube, $[RhCl(coe)_2]_2$ (10.9 mg, 0.015 mmol, 15 mol% of Rh), binap (19.9 mg, 0.032 mmol, 16 mol%), $ZnCl_2$ (81.5 mg, 0.60 mmol) and compound **4ae** (110.2 mg, 0.20 mmol) were placed under nitrogen. Before the tube was sealed, dioxane (1.0 mL) was added. The reaction mixture was heated at 130 °C for 16 h. The reaction mixture was passed through a short pad of basic aluminum oxide with ethyl acetate as the eluent. The solvent was removed on a rotary evaporator. The residue was subjected to chromatography on silica gel (pre-treated with 1% Et_3N in hexanes) to give **3ae** (16%, 17.6 mg, 0.32 mmol) as colorless oil.

Scheme 3b, entry 2: The procedures for entry 2 in Scheme 3b were modified by removing $ZnCl_2$. The reaction of compound **4ae** (112.2 mg, 0.21 mmol), $[RhCl(coe)_2]_2$ (10.5 mg, 0.015 mmol, 15 mol% of Rh) and binap (19.5 mg, 0.032 mmol, 16 mol%) in dioxane (1.0 mL) at 130 °C for 16 h did not give **3ae**. Compound **3ae** was not detected by ^{1}H NMR or GC.

Scheme 3c

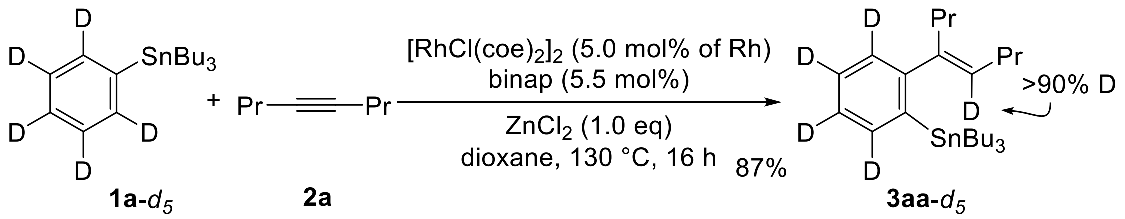

Scheme 3c: The procedures shown in Section 6 for the reaction of $PhSnBu_3$ (**1a**) with 4-octyne (**2a**) (Table 1, entry 1) were used for the reaction of $C_6D_5SnBu_3$ (**1a**-*d*$_5$) with 4-octyne (**2a**). Thus, the reaction of $[RhCl(coe)_2]_2$ (3.61 mg, 0.0050 mmol, 5.0 mol% of Rh), binap (6.92 mg, 0.0110 mmol, 5.5 mol%), $ZnCl_2$ (27.6 mg, 0.20 mmol), $C_6D_5SnBu_3$ (**1a**-*d*$_5$) (148.9 mg, 0.40 mmol) and 4-octyne (**2a**) (22.3 mg, 0.20 mmol) in dioxane (1.0 mL) at 130 °C for 16 h gave **3aa**-*d*$_5$ (87% yield, 83.9 mg, 0.17 mmol) as colorless oil.

Scheme 3d

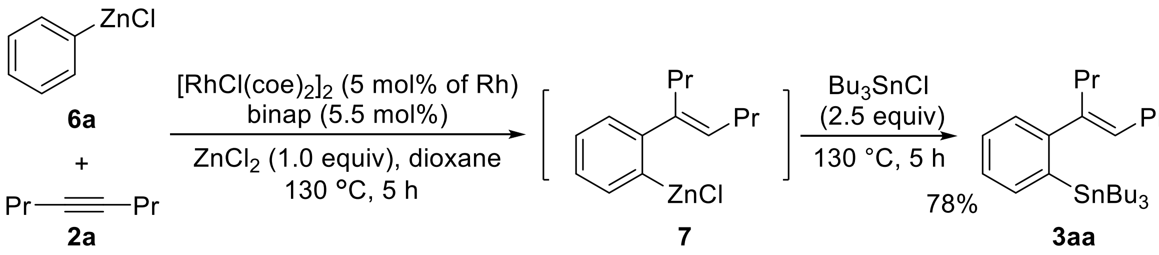

Preparation of PhZnCl (6a): An oven-dried Schlenk flask equipped with a stir bar was charged with a solution of bromobenzene (3.39 g, 22.0 mmol) in THF (12.0 mL). The solution was cooled down to –80 °C and n-BuLi (7.4 mL, 20.0 mmol, 2.69 M in hexanes) was added dropwise over 30 min. Precipitates were formed immediately. The reaction mixture was stirred at –80 °C for 90 min. To the THF solution of phenyllithium thus generated, the THF solution of $ZnCl_2$ (30.0 mL, 30 mmol, 1.0 M) was added dropwise at –80 °C. The reaction mixture was allowed to warm to room temperature to give a solution of phenylzinc chloride (**6a**) (0.4 M) and $ZnCl_2$ (0.2 M) in THF.

Scheme 3d: In an oven-dried Schlenk tube, $[RhCl(coe)_2]_2$ (3.58 mg, 0.0050 mmol, 5.0 mol% of Rh) and binap (6.85 mg, 0.0110 mmol, 5.5 mol%) were placed under nitrogen. Dioxane (1.2 mL) was added and the mixture was stirred at room temperature for 10 min. To the mixture, 4-octyne (**2a**) (22.3 mg, 0.20 mmol) and 1.0 mL of the THF solution containing phenylzinc chloride (**6a**) (0.40 mmol, 0.4 M) and $ZnCl_2$ (0.4 mmol, 0.2 M), whose preparation is shown above, were added at room temperature. After the mixture was concentrated to *ca.* 1.0 mL under the flow of dry N_2, the reaction mixture was heated at 130 °C for 5 h. Bu_3SnCl (163.1 mg, 0.50 mmol) was added to the mixture at room temperature and the mixture was heated at 130 °C for another 5 h. The reaction mixture was passed through a short pad of basic aluminum oxide with ethyl acetate as the eluent. The solvent was removed on a rotary evaporator.

The residue was subjected to chromatography on silica gel (pre-treated with 1% Et_3N in hexanes) to give (*E*)-4-(2-tributylstannylphenyl)oct-4-ene (**3aa**) (78%, 74.6 mg, 0.16 mmol) as colorless oil.

8. Transformations of (*E*)-4-(2-tributylstannylphenyl)oct-4-ene (3aa) (Scheme 4)

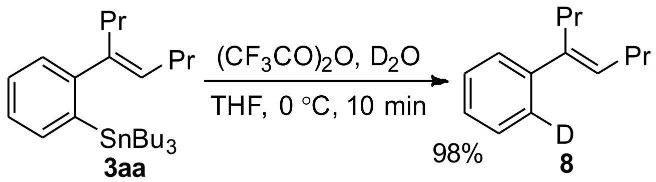

Deuterodestannylation of **3aa** was carried out according to a reported procedure.[12] To a mixture of trifluoroacetic anhydride (3.02g, 14.4 mmol) and **3aa** (85.9 mg, 0.18 mmol) in THF (1.0 mL) at 0 °C was added deuterium oxide (0.27 g, 13.5 mmol). The mixture was stirred at 0 °C for 10 min and was neutralized by addition of 6 N aqueous sodium hydroxide. The mixture was extracted with Et_2O, and the combined organic layer was dried over $MgSO_4$ and concentrated under reduced pressure. The residue was subjected to chromatography on silica gel to give (*E*)-4-(2-deuteriophenyl)oct-4-ene (**8**) (98%, 33.5 mg, 0.18 mmol) as colorless oil.

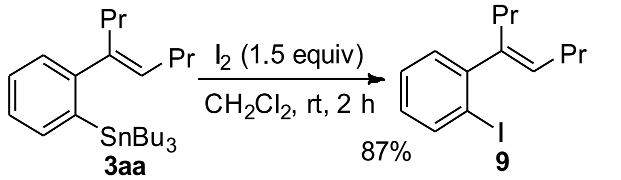

Iododestannylation of **3aa** was carried out according to a reported procedure[11] with minor modifications. An oven-dried flask was charged with **3aa** (114.7 mg, 0.24 mmol), I_2 (91.4 mg, 0.36 mmol) and CH_2Cl_2 (5.0 mL). The mixture was stirred at room temperatue for 2 h before saturated aqueous solution of $Na_2S_2O_3$ (3.0 mL) was added. The mixture was extracted with Et_2O, and the combined organic layer was dried over $MgSO_4$ and concentrated under reduced pressure. The residue was subjected to chromatography on silica gel (pre-treated with 1% Et_3N in hexanes) to give (*E*)-4-(2-iodophenyl)oct-4-ene (**9**) (87%, 65.5 mg, 0.21 mmol) as colorless oil.

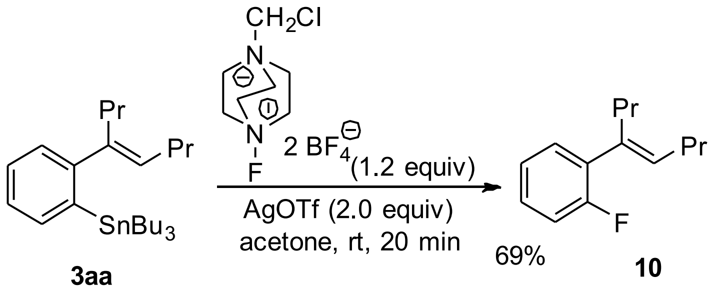

Fluorodestannylation of **3aa** was carried out according to a reported procedure.[13] Under nitrogen at room temperature, to a solution of **3aa** (90.6 mg, 0.19 mmol) in dry acetone (2.0 mL) was added silver triflate (97.6 mg, 0.38 mmol) and 1-chloromethyl-4-fluoro-1,4-diazoniabicyclo[2.2.2]octane

bis(tetrafluoroborate) (80.9 mg, 0.23 mmol). The mixture was stirred at room temperature for 20 min, and it was passed through a short pad of silica gel with ethyl acetate as the eluent. The solvent was removed on a rotary evaporator. The residue was subjected to chromatography on silica gel to give (*E*)-4-(2-fluorophenyl)oct-4-ene (**10**) (69%, 27.1 mg, 0.13 mmol) as colorless oil.

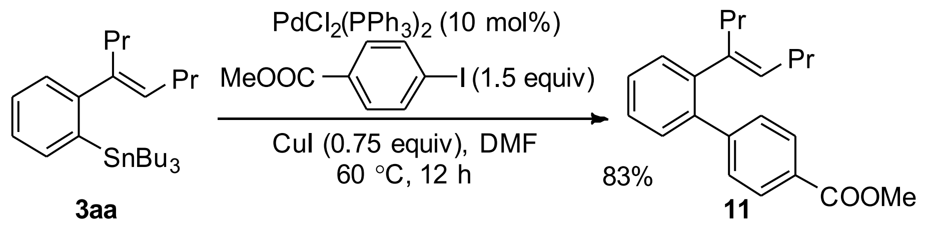

The cross-coupling of **3aa** was performed according to a reported procedure[14] with some modifications. Under nitrogen, **3aa** (95.5 mg, 0.20 mmol), methyl 4-iodobenzoate (78.6 mg, 0.30 mmol), $PdCl_2(PPh_3)_2$ (14.0 mg, 0.020 mmol, 10 mol%) and CuI (28.6 mg, 0.15 mmol) were placed in a 10 mL Schlenk tube. DMF (4.0 mL) was added by syringe and the mixture was allowed to stir at 60 °C for 12 h. The mixture was passed through a short pad of silica gel with ethyl acetate as the eluent. The solvent was removed on a rotary evaporator. The residue was subjected to chromatography on silica gel to give (*E*)-4-(2-(4-methoxycarbonylphenyl)phenyl)oct-4-ene (**11**) (83%, 53.5 mg, 0.17 mmol) as pale yellow oil.

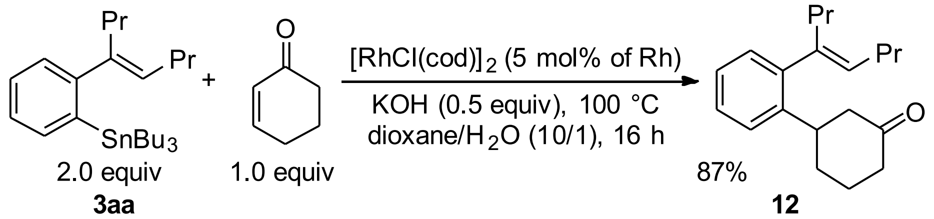

$[RhCl(cod)]_2$ (2.46 mg, 0.0050 mmol, 5 mol% of Rh), **3aa** (190.9 mg, 0.40 mmol), 2-cyclohexenone (19.2 mg, 0.20 mmol) and KOH (5.61 mg, 0.10 mmol) were placed in a 10 mL Schlenk tube under nitrogen. Dioxane (1.0 mL) and water (0.1 mL) were added by syringe, and the mixture was heated at 100 °C for 16 h. The mixture was passed through a short pad of silica gel with ethyl acetate as the eluent. The solvent was removed on a rotary evaporator. The residue was subjected to chromatography on silica gel to give the product **12** (87%, 49.5 mg, 0.17 mmol) as colorless oil.

9. Characterization of the products

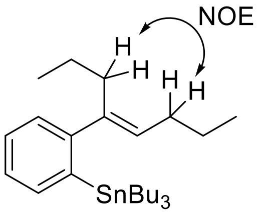

3aa

Compound 3aa. (Table 2, entry 1, 85% yield, colorless oil, R_f= 0.9 (hexanes)). The *E* geometry was assigned by NOESY NMR study. **^{1}H NMR** (400 MHz, $CDCl_3$) δ 0.88 (t, $J_{H,H}$ = 7.3 Hz, 9H), 0.90 (t, $J_{H,H}$ = 7.2 Hz, 3H), 0.97 (t, $J_{H,H}$ = 7.3 Hz, 3H), 0.99 (t, $J_{H,H}$ = 8.0 Hz, $J_{H,^{119}Sn}$ = 51.4 Hz, 6H), 1.32 (sext, $J_{H,H}$ = 7.2 Hz, 6H+2H), 1.42–1.54 (m, 8H), 2.14 (q, $J_{H,H}$ = 7.5 Hz, 2H), 2.32 (t, $J_{H,H}$ = 8.0 Hz, 2H), 5.27 (t, $J_{H,H}$ = 7.0 Hz, 1H), 7.15 (d, $J_{H,H}$ = 7.4 Hz, 1H), 7.18 (td, $J_{H,H}$ = 7.1 Hz, 1.5 Hz, 1H), 7.23 (td, $J_{H,H}$ = 7.3 Hz, 1.6 Hz, 1H), 7.42 (dd, $J_{H,H}$ = 7.0 Hz, 1.2 Hz, $J_{H,^{119}Sn}$ = 42.9 Hz, 1H); **^{13}C NMR** (100 MHz, $CDCl_3$) δ 10.9 ($J_{C,^{119}Sn}$ = 339.7 Hz, $J_{C,^{117}Sn}$ = 324.5 Hz), 13.6, 14.1, 14.4, 21.6, 22.9, 27.5 ($J_{C,^{119}Sn}$ = 60.9 Hz), 29.2 ($J_{C,^{119}Sn}$ = 18.9 Hz), 30.6, 34.9, 125.6 ($J_{C,^{119}Sn}$ = 42.4 Hz), 127.4 ($J_{C,^{119}Sn}$ = 9.6 Hz), 127.9 ($J_{C,^{119}Sn}$ = 35.0 Hz), 129.5, 136.9 ($J_{C,^{119}Sn}$ = 32.7 Hz), 140.7 ($J_{C,^{119}Sn}$ = 412.3 Hz, $J_{C,^{117}Sn}$ = 393.8 Hz), 144.3 ($J_{C,^{119}Sn}$ = 14.6 Hz), 152.7 ($J_{C,^{119}Sn}$ = 28.4 Hz). **HRMS** (ESI) calcd for $C_{26}H_{46}Na^{120}Sn$ $[M+Na]^+$ 501.2519, found 501.2537.

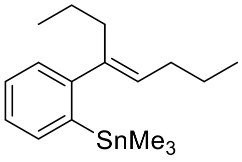

3ba

Compound 3ba. (Table 2, entry 2, 77% yield, colorless oil, R_f= 0.9 (hexanes)). **^{1}H NMR** (400 MHz, $CDCl_3$) δ 0.24 (t, $J_{H,^{119}Sn}$ = 53.0 Hz, 9H), 0.91 (t, $J_{H,H}$ = 7.3 Hz, 3H), 0.97 (t, $J_{H,H}$ = 7.4 Hz, 3H), 1.37 (sext, $J_{H,H}$ = 7.8 Hz, 2H), 1.46 (sext, $J_{H,H}$ = 7.4 Hz, 2H), 2.15 (q, $J_{H,H}$ = 7.4 Hz, 2H), 2.34 (t, $J_{H,H}$ = 8.1 Hz, 2H), 5.31 (t, $J_{H,H}$ = 7.1 Hz, 1H), 7.19 (d, $J_{H,H}$ = 7.5 Hz, 1H), 7.22 (t, $J_{H,H}$ = 6.9 Hz, 1H), 7.24–7.28 (m, 1H), 7.47 (d, $J_{H,H}$ = 6.9 Hz, $J_{H,^{119}Sn}$ = 49.4 Hz, 1H); **^{13}C NMR** (100 MHz, $CDCl_3$) δ –7.4 ($J_{C,^{119}Sn}$ = 350.4 Hz, $J_{C,^{117}Sn}$ = 334.8 Hz), 14.1, 14.5, 21.6, 22.9, 30.5, 34.8, 125.9 ($J_{C,^{119}Sn}$ = 47.8 Hz), 127.6 ($J_{C,^{119}Sn}$ = 38.2 Hz), 127.8 ($J_{C,^{119}Sn}$ = 9.5 Hz), 130.0, 136.3 ($J_{C,^{119}Sn}$ = 38.6 Hz), 141.0 ($J_{C,^{119}Sn}$ = 497.6 Hz, $J_{C,^{117}Sn}$ = 475.1 Hz), 144.2 ($J_{C,^{119}Sn}$ = 16.4 Hz), 152.4 ($J_{C,^{119}Sn}$ = 31.1 Hz). **HRMS** (ESI) calcd for $C_{17}H_{28}Na^{120}Sn$ $[M+Na]^+$ 375.1111, found 375.1118.

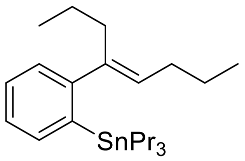

3ca

Compound 3ca. (Table 2, entry 3, 89% yield, colorless oil, R_f = 0.9 (hexanes)). **^{1}H NMR** (400 MHz, $CDCl_3$) δ 0.90 (t, $J_{H,H}$ = 7.3 Hz, 3H), 0.96 (t, $J_{H,H}$ = 7.2 Hz, 9H), 0.98 (t, $J_{H,H}$ = 7.4 Hz, 3H), 1.00 (t, $J_{H,H}$ = 8.3 Hz, $J_{H,^{119}Sn}$ = 50.8 Hz, 6H),1.36 (sext, $J_{H,H}$ = 7.6 Hz, 2H), 1.47 (sext, $J_{H,H}$ = 7.5 Hz, 2H), 1.55 (sext, $J_{H,H}$ = 7.7 Hz, 6H), 2.14 (q, $J_{H,H}$ = 7.4 Hz, 2H), 2.32 (t, $J_{H,H}$ = 8.0 Hz, 2H), 5.27 (t, $J_{H,H}$ = 7.0 Hz, 1H), 7.16 (dd, $J_{H,H}$ = 7.6 Hz, 1.3 Hz, 1H), 7.18 (td, $J_{H,H}$ = 7.1 Hz, 1.6 Hz, 1H), 7.23 (td, $J_{H,H}$ = 7.3 Hz, 1.6 Hz, 1H), 7.43 (dd, $J_{H,H}$ = 7.0 Hz, 1.4 Hz, $J_{H,^{119}Sn}$ = 43.0 Hz, 1H); **^{13}C NMR** (100 MHz, $CDCl_3$) δ 13.9 ($J_{C,^{119}Sn}$ = 339.0 Hz, $J_{C,^{117}Sn}$ = 323.8 Hz), 14.1, 14.4, 19.1 ($J_{C,^{119}Sn}$ = 62.6 Hz, $J_{C,^{117}Sn}$ = 59.2 Hz), 20.4 ($J_{C,^{119}Sn}$ = 18.6 Hz), 21.6, 22.9, 30.6, 34.9, 125.6 ($J_{C,^{119}Sn}$ = 41.0 Hz), 127.5 ($J_{C,^{119}Sn}$ = 9.5 Hz), 128.0 ($J_{C,^{119}Sn}$ = 35.1 Hz), 129.5, 136.9 ($J_{C,^{119}Sn}$ = 32.4 Hz), 140.7 ($J_{C,^{119}Sn}$ = 412.6 Hz, $J_{C,^{117}Sn}$ = 394.1 Hz), 144.3 ($J_{C,^{119}Sn}$ = 14.7 Hz), 152.7 ($J_{C,^{119}Sn}$ = 27.7 Hz). **HRMS** (ESI) calcd for $C_{23}H_{40}Na^{120}Sn$ $[M+Na]^+$ 459.2059, found 459.2050.

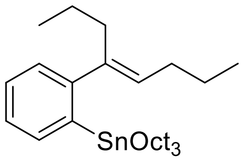

3da

Compound 3da. (Table 2, entry 4, 78% yield, colorless oil, R_f = 0.9 (hexanes)). **^{1}H NMR** (400 MHz, $CDCl_3$) δ 0.88 (t, $J_{H,H}$ = 7.0 Hz, 9H), 0.90 (t, $J_{H,H}$ = 7.2 Hz, 3H), 0.97 (t, $J_{H,H}$ = 7.2 Hz, 3H), 0.98 (t, $J_{H,H}$ = 8.3 Hz, $J_{H,^{119}Sn}$ = 50.7 Hz, 6H), 1.20–1.40 (br m, 32H), 1.41–1.60 (m, 8H), 2.14 (q, $J_{H,H}$ = 7.4 Hz, 2H), 2.31 (t, $J_{H,H}$ = 8.0 Hz, 2H), 5.26 (t, $J_{H,H}$ = 7.0 Hz, 1H), 7.15 (d, $J_{H,H}$ = 7.6 Hz, 1H), 7.17 (td, $J_{H,H}$ = 7.1 Hz, 1.5 Hz, 1H), 7.22 (td, $J_{H,H}$ = 7.3 Hz, 1.6 Hz, 1H), 7.41 (dd, $J_{H,H}$ = 7.0 Hz, 1.3 Hz, $J_{H,^{119}Sn}$ = 42.8 Hz, 1H); **^{13}C NMR** (100 MHz, $CDCl_3$) δ 11.2 ($J_{C,^{119}Sn}$ = 339.2 Hz, $J_{C,^{117}Sn}$ = 323.0 Hz), 14.09, 14.13, 14.4, 21.6, 22.7, 23.0, 26.9 ($J_{C,^{119}Sn}$ = 18.6 Hz), 29.2, 29.3, 30.6, 31.9, 34.5 ($J_{C,^{119}Sn}$ = 58.1 Hz), 34.9, 125.6 ($J_{C,^{119}Sn}$ = 41.7 Hz), 127.4, 127.9, 129.5, 136.9 ($J_{C,^{119}Sn}$ = 32.9 Hz), 140.8, 144.3, 152.7. **HRMS** (ESI) calcd for $C_{38}H_{71}{}^{120}Sn$ $[M+H]^+$ 647.4578, found 647.4565.

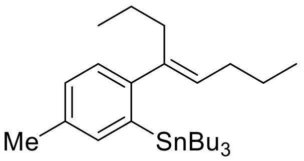

3ea

Compound 3ea. (Table 2, entry 5, 87% yield, colorless oil, R_f = 0.9 (hexanes)). **^{1}H NMR** (400 MHz, $CDCl_3$) δ 0.88 (t, $J_{H,H}$ = 7.3 Hz, 9H), 0.89 (t, $J_{H,H}$ = 7.3 Hz, 3H), 0.96 (t, $J_{H,H}$ = 7.3 Hz, 3H), 0.98

(t, $J_{H,H}$ = 7.8 Hz, $J_{H,^{119}Sn}$ = 49.6 Hz, 6H), 1.32 (sext, $J_{H,H}$ = 7.3 Hz, 6H), 1.28–1.40 (m, 2H), 1.41–1.54 (m, 8H), 2.12 (q, $J_{H,H}$ = 7.4 Hz, 2H), 2.30 (t, $J_{H,H}$ = 8.2 Hz, 2H), 2.32 (s, 3H), 5.25 (t, $J_{H,H}$ = 7.0 Hz, 1H), 7.02–7.08 (m, 2H), 7.21 (s, $J_{H,^{119}Sn}$ = 44.3 Hz, 1H); **^{13}C NMR** (100 MHz, $CDCl_3$) δ 10.8 ($J_{C,^{119}Sn}$ = 338.7 Hz, $J_{C,^{117}Sn}$ = 323.7 Hz), 13.6, 14.1, 14.4, 21.1, 21.6, 23.0, 27.5 ($J_{C,^{119}Sn}$ = 62.3 Hz, $J_{C,^{117}Sn}$ = 59.4 Hz), 29.2 ($J_{C,^{119}Sn}$ = 18.7 Hz), 30.6, 35.0, 127.7 ($J_{C,^{119}Sn}$ = 37.0 Hz), 128.3, 129.4, 134.8, 137.6, 140.5, 144.1, 149.8. **HRMS** (ESI) calcd for $C_{27}H_{48}Na^{120}Sn$ $[M+Na]^+$ 515.2676, found 515.2684.

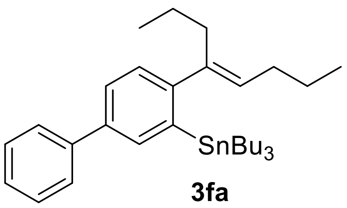

3fa

Compound 3fa. (Table 2, entry 6, 91% yield, colorless oil, R_f= 0.8 (hexanes)). **^{1}H NMR** (400 MHz, $CDCl_3$) δ 0.89 (t, $J_{H,H}$ = 7.3 Hz, 9H), 0.92 (t, $J_{H,H}$ = 7.4 Hz, 3H), 0.98 (t, $J_{H,H}$ = 7.4 Hz, 3H), 1.02 (t, $J_{H,H}$ = 8.3 Hz, $J_{H,^{119}Sn}$ = 47.2 Hz, 6H), 1.33 (sext, $J_{H,H}$ = 7.3 Hz, 6H), 1.32–1.42 (m, 2H), 1.44–1.55 (m, 8H), 2.16 (q, $J_{H,H}$ = 7.4 Hz, 2H), 2.35 (t, $J_{H,H}$ = 8.0 Hz, 2H), 5.32 (t, $J_{H,H}$ = 7.0 Hz, 1H), 7.23 (d, $J_{H,H}$ = 8.0 Hz, $J_{H,^{119}Sn}$ = 16.0 Hz, 1H), 7.32 (t, $J_{H,H}$ = 7.3 Hz, 1H), 7.43 (t, $J_{H,H}$ = 7.7 Hz, 2H), 7.45 (d, $J_{H,H}$ = 8.0 Hz, 1H), 7.58 (d, $J_{H,H}$ = 7.1 Hz, 2H), 7.63 (d, $J_{H,H}$ = 1.9 Hz, $J_{H,^{119}Sn}$ = 43.6 Hz, 1H); **^{13}C NMR** (100 MHz, $CDCl_3$) δ 11.0 ($J_{C,^{119}Sn}$ = 339.7 Hz, $J_{C,^{117}Sn}$ = 324.9 Hz), 13.7, 14.1, 14.5, 21.7, 23.0, 27.5 ($J_{C,^{119}Sn}$ = 61.4 Hz), 29.2 ($J_{C,^{119}Sn}$ = 18.7 Hz), 30.6, 34.9, 126.4 ($J_{C,^{119}Sn}$ = 9.7 Hz), 126.9, 127.1, 128.1 ($J_{C,^{119}Sn}$ = 36.1 Hz), 128.7, 129.7, 135.6 ($J_{C,^{119}Sn}$ = 32.9 Hz), 138.2, 141.3, 141.5, 144.0 ($J_{C,^{119}Sn}$ = 13.9 Hz), 151.8. **HRMS** (ESI) calcd for $C_{32}H_{50}Na^{120}Sn$ $[M+Na]^+$ 577.2832, found 577.2823.

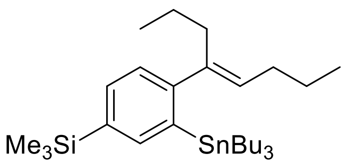

3ga

Compound 3ga. (Table 2, entry 7, 83% yield, colorless oil, R_f= 0.9 (hexanes)). **^{1}H NMR** (400 MHz, $CDCl_3$) δ 0.26 (s, 9H), 0.89 (t, $J_{H,H}$ = 7.2 Hz, 9H+3H), 0.96 (t, $J_{H,H}$ = 7.4 Hz, 3H), 0.99 (t, $J_{H,H}$ = 8.4 Hz, $J_{H,^{119}Sn}$ = 48.8 Hz, 6H), 1.33 (sext, $J_{H,H}$ = 7.3 Hz, 6H+2H), 1.43–1.53 (m, 8H), 2.14 (q, $J_{H,H}$ = 7.4 Hz, 2H), 2.32 (t, $J_{H,H}$ = 8.0 Hz, 2H), 5.27 (t, $J_{H,H}$ = 7.0 Hz, 1H), 7.15 (d, $J_{H,H}$ = 7.5 Hz, $J_{H,^{119}Sn}$ = 15.0 Hz, 1H), 7.39 (dd, $J_{H,H}$ = 7.5 Hz, 1.4 Hz, 1H), 7.58 (s, $J_{H,^{119}Sn}$ = 42.3 Hz, 1H); **^{13}C NMR** (100 MHz, $CDCl_3$) δ –1.0, 11.0 ($J_{C,^{119}Sn}$ = 337.9 Hz, $J_{C,^{117}Sn}$ = 322.1 Hz), 13.7, 14.1, 14.4, 21.7, 22.9, 27.5 ($J_{C,^{119}Sn}$ = 60.1 Hz), 29.2 ($J_{C,^{119}Sn}$ = 19.5 Hz), 30.6, 34.8, 127.2, 129.7, 132.6, 136.7, 139.7, 142.2, 144.3, 153.1. **HRMS** (ESI) calcd for $C_{29}H_{54}NaSi^{120}Sn$ $[M+Na]^+$ 573.2914, found 573.2903.

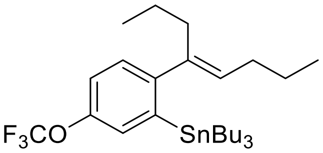

3ha

Compound 3ha. (Table 2, entry 8, 83% yield, colorless oil, R_f = 0.8 (hexanes)). **^{1}H NMR** (400 MHz, $CDCl_3$) δ 0.88 (t, $J_{H,H}$ = 7.2 Hz, 9H), 0.90 (t, $J_{H,H}$ = 7.1 Hz, 3H), 0.97 (t, $J_{H,H}$ = 7.4 Hz, 3H), 1.01 (t, $J_{H,H}$ = 8.2 Hz, $J_{H,^{119}Sn}$ = 49.9 Hz, 6H), 1.32 (sext, $J_{H,H}$ = 7.2 Hz, 6H+2H), 1.43–1.54 (m, 8H), 2.13 (q, $J_{H,H}$ = 7.4 Hz, 2H), 2.30 (t, $J_{H,H}$ = 8.0 Hz, 2H), 5.27 (t, $J_{H,H}$ = 7.1 Hz, 1H), 7.04 (dd, $J_{H,H}$ = 8.4 Hz, 1.4 Hz, 1H), 7.14 (d, $J_{H,H}$ = 8.4 Hz, $J_{H,^{119}Sn}$ = 16.5 Hz, 1H), 7.23 (d, $J_{H,H}$ = 1.5 Hz, $J_{H,^{119}Sn}$ = 43.4 Hz, 1H); **^{13}C NMR** (100 MHz, $CDCl_3$) δ 11.0 ($J_{C,^{119}Sn}$ = 343.7 Hz, $J_{C,^{117}Sn}$ = 328.4 Hz), 13.6, 14.1, 14.3, 21.5, 22.9, 27.4 ($J_{C,^{119}Sn}$ = 62.7 Hz, $J_{C,^{117}Sn}$ = 59.2 Hz), 29.0 ($J_{C,^{119}Sn}$ = 19.2 Hz), 30.6, 34.9, 119.7, 120.6 (q, $J_{C,^{19}F}$ = 256.5 Hz), 128.6 ($J_{C,^{119}Sn}$ = 34.4 Hz), 128.9 ($J_{C,^{119}Sn}$ = 37.0 Hz), 130.2, 143.3 ($J_{C,^{119}Sn}$ = 13.1 Hz), 143.4 ($J_{C,^{119}Sn}$ = 380.6 Hz, $J_{C,^{117}Sn}$ = 343.7 Hz), 147.3, 151.2 ($J_{C,^{119}Sn}$ = 25.0 Hz). **HRMS** (ESI) calcd for $C_{27}H_{45}F_3ONa^{120}Sn$ $[M+Na]^+$ 585.2342, found 585.2335.

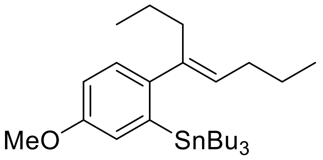

3ia

Compound 3ia. (Table 2, entry 9, 65% yield, colorless oil, R_f = 0.8 (hexanes)). **^{1}H NMR** (400 MHz, $CDCl_3$) δ 0.88 (t, $J_{H,H}$ = 7.3 Hz, 9H), 0.89 (t, $J_{H,H}$ = 7.3 Hz, 3H), 0.96 (t, $J_{H,H}$ = 7.4 Hz, 3H), 0.98 (t, $J_{H,H}$ = 8.4 Hz, $J_{H,^{119}Sn}$ = 50.5 Hz, 6H), 1.32 (sext, $J_{H,H}$ = 7.3 Hz, 6H+2H), 1.42–1.52 (m, 8H), 2.11 (q, $J_{H,H}$ = 7.4 Hz, 2H), 2.29 (t, $J_{H,H}$ = 8.0 Hz, 2H), 3.80 (s, 3H), 5.24 (t, $J_{H,H}$ = 7.0 Hz, 1H), 6.76 (dd, $J_{H,H}$ = 8.4 Hz, 2.8 Hz, 1H), 6.97 (d, $J_{H,H}$ = 2.8 Hz, $J_{H,^{119}Sn}$ = 46.6 Hz, 1H), 7.09 (d, $J_{H,H}$ = 8.4 Hz, $J_{H,^{119}Sn}$ = 17.6 Hz, 1H); **^{13}C NMR** (100 MHz, $CDCl_3$) δ 10.9 ($J_{C,^{119}Sn}$ = 340.5 Hz, $J_{C,^{117}Sn}$ = 325.5 Hz), 13.6, 14.1, 14.4, 21.6, 23.0, 27.5 ($J_{C,^{119}Sn}$ = 60.6 Hz), 29.1 ($J_{C,^{119}Sn}$ = 18.9 Hz), 30.6, 35.0, 55.1, 112.2, 122.4, 128.6, 129.3, 142.2, 143.8, 145.2, 157.2. **HRMS** (ESI) calcd for $C_{27}H_{48}ONa^{120}Sn$ $[M+Na]^+$ 531.2625, found 531.2617.

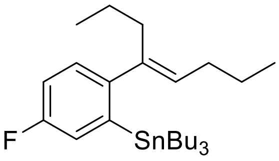

3ja

Compound 3ja. (Table 2, entry 10, 77% yield, colorless oil, R_f = 0.8 (hexanes)). **^{1}H NMR** (400 MHz, $CDCl_3$) δ 0.886 (t, $J_{H,H}$ = 7.3 Hz, 9H), 0.894 (t, $J_{H,H}$ = 7.3 Hz, 3H), 0.97 (t, $J_{H,H}$ = 7.4 Hz, 3H), 0.99 (t, $J_{H,H}$ = 8.3 Hz, $J_{H,^{119}Sn}$ = 50.9 Hz, 6H), 1.32 (sext, $J_{H,H}$ = 7.3 Hz, 6H), 1.27–1.37 (m, 2H), 1.41–1.53 (m, 8H), 2.12 (q, $J_{H,H}$ = 7.4 Hz, 2H), 2.28 (t, $J_{H,H}$ = 8.0 Hz, 2H), 5.24 (t, $J_{H,H}$ = 7.0 Hz, 1H), 6.88

(td, $J_{H,H} = J_{H,^{19}F} = 8.6$ Hz, 2.8 Hz, 1H), 7.09 (dd, $J_{H,H} = 2.0$ Hz, $J_{H,^{19}F} = 8.4$ Hz, $J_{H,^{119}Sn} = 43.6$ Hz, 1H), 7.10 (d, $J_{H,H} = 8.3$ Hz, 1H); **^{13}C NMR** (100 MHz, $CDCl_3$) δ 10.9 ($J_{C,^{119}Sn} = 343.0$ Hz, $J_{C,^{117}Sn} = 327.7$ Hz), 13.6, 14.1, 14.4, 21.5, 22.9, 27.4 ($J_{C,^{119}Sn} = 61.7$ Hz), 29.1 ($J_{C,^{119}Sn} = 19.1$ Hz), 30.6, 35.0, 114.0 (d, $J_{C,^{19}F} = 20.9$ Hz, $J_{C,^{119}Sn} = 8.7$ Hz), 122.7 (d, $J_{C,^{19}F} = 17.5$ Hz, $J_{C,^{119}Sn} = 34.1$ Hz), 129.2 (d, $J_{C,^{19}F} = 6.5$ Hz, $J_{C,^{119}Sn} = 39.4$ Hz), 129.9, 143.4 ($J_{C,^{119}Sn} = 13.2$ Hz), 143.6 (d, $J_{C,^{19}F} = 2.1$ Hz), 148.4 (d, $J_{C,^{19}F} = 3.1$ Hz, $J_{C,^{119}Sn} = 25.8$ Hz), 161.1 (d, $J_{C,^{19}F} = 248.6$ Hz). **HRMS** (ESI) calcd for $C_{26}H_{45}FNa^{120}Sn$ $[M+Na]^+$ 519.2425, found 519.2407.

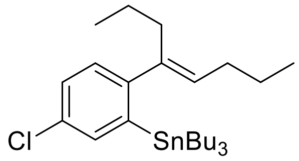

3ka

Compound 3ka. (Table 2, entry 12, 84% yield, colorless oil, $R_f = 0.8$ (hexanes)). **^{1}H NMR** (400 MHz, $CDCl_3$) δ 0.89 (t, $J_{H,H} = 7.3$ Hz, 9H+3H), 0.96 (t, $J_{H,H} = 7.3$ Hz, 3H), 1.00 (t, $J_{H,H} = 8.3$ Hz, $J_{H,^{119}Sn} = 51.7$ Hz, 6H), 1.32 (sext, $J_{H,H} = 7.3$ Hz, 6H+2H), 1.42–1.51 (m, 8H), 2.12 (q, $J_{H,H} = 7.4$ Hz, 2H), 2.28 (t, $J_{H,H} = 8.0$ Hz, 2H), 5.25 (t, $J_{H,H} = 7.0$ Hz, 1H), 7.06 (d, $J_{H,H} = 8.2$ Hz, $J_{H,^{119}Sn} = 16.2$ Hz, 1H), 7.18 (dd, $J_{H,H} = 8.2$ Hz, 2.3 Hz, 1H), 7.34 (d, $J_{H,H} = 2.3$ Hz, $J_{H,^{119}Sn} = 42.1$ Hz, 1H); **^{13}C NMR** (100 MHz, $CDCl_3$) δ 10.9 ($J_{C,^{119}Sn} = 342.6$ Hz, $J_{C,^{117}Sn} = 327.6$ Hz), 13.6, 14.1, 14.4, 21.5, 22.9, 27.4 ($J_{C,^{119}Sn} = 62.1$ Hz), 29.1 ($J_{C,^{119}Sn} = 19.0$ Hz), 30.5, 34.8, 127.4 ($J_{C,^{119}Sn} = 8.5$ Hz), 129.2 ($J_{C,^{119}Sn} = 35.7$ Hz), 130.0, 132.0 ($J_{C,^{119}Sn} = 53.4$ Hz), 136.1 ($J_{C,^{119}Sn} = 34.6$ Hz), 143.3($J_{C,^{119}Sn} = 13.0$ H), 143.4, 150.8 ($J_{C,^{119}Sn} = 25.4$ Hz). **HRMS** (ESI) calcd for $C_{26}H_{45}NaCl^{120}Sn$ $[M+Na]^+$ 535.2129, found 535.2140.

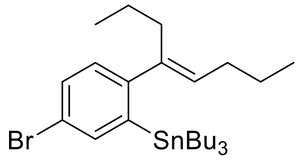

3la

Compound 3la. (Table 2, entry 14, 78% yield, colorless oil, $R_f = 0.8$ (hexanes)). **^{1}H NMR** (400 MHz, $CDCl_3$) δ 0.89 (t, $J_{H,H} = 7.3$ Hz, 9H+3H), 0.96 (t, $J_{H,H} = 7.3$ Hz, 3H), 1.00 (t, $J_{H,H} = 8.3$ Hz, $J_{H,^{119}Sn} = 51.2$ Hz, 6H), 1.32 (sext, $J_{H,H} = 7.3$ Hz, 6H+2H), 1.42–1.51 (m, 8H), 2.12 (q, $J_{H,H} = 7.3$ Hz, 2H), 2.28 (t, $J_{H,H} = 8.0$ Hz, 2H), 5.26 (t, $J_{H,H} = 7.0$ Hz, 1H), 7.00 (d, $J_{H,H} = 8.1$ Hz, $J_{H,^{119}Sn} = 16.1$ Hz, 1H), 7.32 (dd, $J_{H,H} = 8.2$ Hz, 2.2 Hz, 1H), 7.48 (d, $J_{H,H} = 2.1$ Hz, $J_{H,^{119}Sn} = 41.4$ Hz, 1H); **^{13}C NMR** (100 MHz, $CDCl_3$) δ 11.0 ($J_{C,^{119}Sn} = 343.0$ Hz, $J_{C,^{117}Sn} = 327.1$ Hz), 13.6, 14.1, 14.4, 21.5, 22.9, 27.4 ($J_{C,^{119}Sn} = 61.5$ Hz), 29.1 ($J_{C,^{119}Sn} = 19.2$ Hz), 30.5, 34.8, 120.7, 129.7, 130.0, 130.3, 139.0, 143.3, 144.1, 151.2. **HRMS** (ESI) calcd for $C_{26}H_{45}NaBr^{120}Sn$ $[M+Na]^+$ 579.1624, found 579.1625.

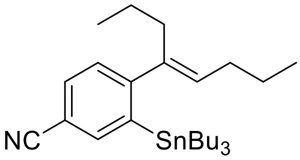

3ma

Compound 3ma. (Table 2, entry 16, 67% yield, colorless oil, R_f = 0.6 (hexanes)). **^{1}H NMR** (400 MHz, $CDCl_3$) δ 0.886 (t, $J_{H,H}$ = 7.3 Hz, 9H), 0.892 (t, $J_{H,H}$ = 7.3 Hz, 3H), 0.97 (t, $J_{H,H}$ = 7.4 Hz, 3H), 1.01 (t, $J_{H,H}$ = 8.3 Hz, $J_{H,^{119}Sn}$ = 51.2 Hz, 6H), 1.31 (sext, $J_{H,H}$ = 7.3 Hz, 6H), 1.25–1.36 (m, 2H), 1.41–1.50 (m, 8H), 2.14 (q, $J_{H,H}$ = 7.4 Hz, 2H), 2.31 (t, $J_{H,H}$ = 8.0 Hz, 2H), 5.28 (t, $J_{H,H}$ = 7.1 Hz, 1H), 7.20 (d, $J_{H,H}$ = 7.9 Hz, $J_{H,^{119}Sn}$ = 14.4 Hz, 1H), 7.50 (dd, $J_{H,H}$ = 7.9 Hz, 1.8 Hz, 1H), 7.66 (d, $J_{H,H}$ = 1.7 Hz, $J_{H,^{119}Sn}$ = 39.2 Hz, 1H); **^{13}C NMR** (100 MHz, $CDCl_3$) δ 11.0 ($J_{C,^{119}Sn}$ = 345.3 Hz, $J_{C,^{117}Sn}$ = 329.8 Hz), 13.6, 14.1, 14.3, 21.6, 22.8, 27.3 ($J_{C,^{119}Sn}$ = 62.3 Hz), 29.0 ($J_{C,^{119}Sn}$ = 19.3 Hz), 30.5, 34.5, 109.6 ($J_{C,^{119}Sn}$ = 44.8 Hz), 119.8, 128.2 ($J_{C,^{119}Sn}$ = 30.9 Hz), 131.0, 131.1 ($J_{C,^{119}Sn}$ = 8.5 Hz), 140.3 ($J_{C,^{119}Sn}$ = 35.6 Hz), 142.9, 143.5 ($J_{C,^{119}Sn}$ = 12.2 H), 157.2 ($J_{C,^{119}Sn}$ = 24.8 Hz). **HRMS** (ESI) calcd for $C_{27}H_{45}NNa^{120}Sn$ $[M+Na]^+$ 526.2472, found 526.2480.

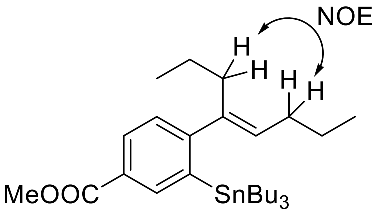

3na

Compound 3na. (Table 2, entry 18, 82% yield, colorless oil, R_f = 0.5 (hexanes)). The *E* geometry was assigned by NOESY NMR study. **^{1}H NMR** (400 MHz, $CDCl_3$) δ 0.88 (t, $J_{H,H}$ = 7.3 Hz, 9H), 0.89 (t, $J_{H,H}$ = 7.3 Hz, 3H), 0.97 (t, $J_{H,H}$ = 7.4 Hz, 3H), 1.02 (t, $J_{H,H}$ = 8.3 Hz, $J_{H,^{119}Sn}$ = 49.9 Hz, 6H), 1.32 (sext, $J_{H,H}$ = 7.3 Hz, 6H+2H), 1.42–1.53 (m, 8H), 2.14 (q, $J_{H,H}$ = 7.4 Hz, 2H), 2.33 (t, $J_{H,H}$ = 8.0 Hz, 2H), 3.90 (s, 3H), 5.30 (t, $J_{H,H}$ = 7.0 Hz, 1H), 7.20 (d, $J_{H,H}$ = 8.0 Hz, $J_{H,^{119}Sn}$ = 15.0 Hz, 1H), 7.87 (dd, $J_{H,H}$ = 8.0 Hz, 1.8 Hz, 1H), 8.09 (d, $J_{H,H}$ = 1.8 Hz, $J_{H,^{119}Sn}$ = 43.2 Hz, 1H); **^{13}C NMR** (75 MHz, $CDCl_3$) δ 10.9 ($J_{C,^{119}Sn}$ = 342.4 Hz, $J_{C,^{117}Sn}$ = 327.3 Hz), 13.6, 14.1, 14.3, 21.6, 22.9, 27.4 ($J_{C,^{119}Sn}$ = 62.3 Hz, $J_{C,^{117}Sn}$ = 59.6 Hz), 29.1 ($J_{C,^{119}Sn}$ = 19.1 Hz), 30.6, 34.6, 51.9, 127.1 ($J_{C,^{119}Sn}$ = 41.3 Hz), 127.8 ($J_{C,^{119}Sn}$ = 32.9 Hz), 128.8 ($J_{C,^{119}Sn}$ = 8.8 Hz), 130.3, 138.1 ($J_{C,^{119}Sn}$ = 35.4 Hz), 141.2, 143.9 ($J_{C,^{119}Sn}$ = 13.1 Hz), 157.5 ($J_{C,^{119}Sn}$ = 27.5 Hz), 167.6. **HRMS** (ESI) calcd for $C_{28}H_{48}O_2Na^{120}Sn$ $[M+Na]^+$ 559.2574, found 559.2605.

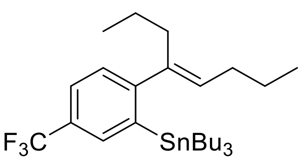

3oa

Compound 3oa. (Table 2, entry 19, 69% yield, colorless oil, R_f = 0.8 (hexanes)). **^{1}H NMR** (400

MHz, $CDCl_3$) δ 0.89 (t, $J_{H,H}$ = 7.3 Hz, 9H), 0.90 (t, $J_{H,H}$ = 7.2 Hz, 3H), 0.97 (t, $J_{H,H}$ = 7.3 Hz, 3H), 1.02 (t, $J_{H,H}$ = 8.3 Hz, $J_{H,^{119}Sn}$ = 52.2 Hz, 6H), 1.32 (sext, $J_{H,H}$ = 7.3 Hz, 6H), 1.27–1.37 (m, 2H), 1.42–1.52 (m, 8H), 2.15 (q, $J_{H,H}$ = 7.4 Hz, 2H), 2.32 (t, $J_{H,H}$ = 8.0 Hz, 2H), 5.29 (t, $J_{H,H}$ = 7.1 Hz, 1H), 7.23 (d, $J_{H,H}$ = 7.9 Hz, $J_{H,^{119}Sn}$ = 14.5 Hz, 1H), 7.46 (dd, $J_{H,H}$ = 8.0 Hz, 1.5 Hz, 1H), 7.63 (s, $J_{H,^{119}Sn}$ = 42.4 Hz, 1H); **^{13}C NMR** (100 MHz, $CDCl_3$) δ 10.9 ($J_{C,^{119}Sn}$ = 343.6 Hz, $J_{C,^{117}Sn}$ = 328.7 Hz), 13.6, 14.1, 14.4, 21.6, 22.9, 27.4 ($J_{C,^{119}Sn}$ = 62.0 Hz), 29.1 ($J_{C,^{119}Sn}$ = 19.2 Hz), 30.5, 34.7, 124.4 (q, $J_{C,^{19}F}$ = 3.8 Hz), 124.7 (q, $J_{C,^{19}F}$ = 272.2 Hz), 127.5 (q, $J_{C,^{19}F}$ = 31.6 Hz), 127.9, 130.5, 133.2 (q, $J_{C,^{19}F}$ = 3.4 Hz), 142.0, 143.6, 156.2. **HRMS** (ESI) calcd for $C_{27}H_{45}F_3Na^{120}Sn$ $[M+Na]^+$ 569.2393, found 569.2371.

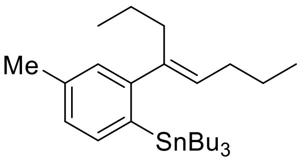

3pa

Compound 3pa. (Table 2, entry 20, 77% yield, colorless oil, R_f = 0.9 (hexanes)). **^{1}H NMR** (400 MHz, $CDCl_3$) δ 0.88 (t, $J_{H,H}$ = 7.3 Hz, 9H+3H), 0.97 (t, $J_{H,H}$ = 8.2 Hz, $J_{H,^{119}Sn}$ = 50.2 Hz, 6H), 0.97 (t, $J_{H,H}$ = 8.2 Hz, 3H), 1.32 (sext, $J_{H,H}$ = 7.4 Hz, 6H), 1.29–1.38 (m, 2H) 1.43–1.53 (m, 8H), 2.13 (q, $J_{H,H}$ = 7.4 Hz, 2H), 2.31 (t, $J_{H,H}$ = 8.2 Hz, 2H), 2.32 (s, 3H), 5.26 (t, $J_{H,H}$ = 7.0 Hz, 1H), 6.99 (s, 1H), 7.02 (d, $J_{H,H}$ = 7.5 Hz, 1H), 7.31 (d, $J_{H,H}$ = 7.4 Hz, $J_{H,^{119}Sn}$ = 42.9 Hz, 1H); **^{13}C NMR** (100 MHz, $CDCl_3$) δ 10.8 ($J_{C,^{119}Sn}$ = 339.9 Hz, $J_{C,^{117}Sn}$ = 324.5 Hz), 13.6, 14.1, 14.4, 21.3, 21.7, 23.0, 27.5 ($J_{C,^{119}Sn}$ = 61.0 Hz), 29.2 ($J_{C,^{119}Sn}$ = 18.6 Hz), 30.6, 34.9, 126.6 ($J_{C,^{119}Sn}$ = 44.5 Hz), 128.8 ($J_{C,^{119}Sn}$ = 36.6 Hz), 129.4, 136.7, 136.9, 137.1, 144.4 ($J_{C,^{119}Sn}$ = 14.5 Hz), 152.7 ($J_{C,^{119}Sn}$ = 29.1 Hz). **HRMS** (ESI) calcd for $C_{27}H_{48}Na^{120}Sn$ $[M+Na]^+$ 515.2676, found 515.2698.

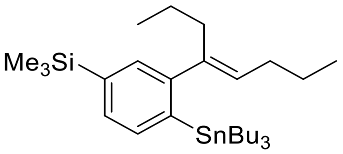

3qa

Compound 3qa. (Table 2, entry 21, 84% yield, colorless oil, R_f = 0.9 (hexanes)). **^{1}H NMR** (500 MHz, $CDCl_3$) δ 0.27 (s, 9H), 0.90 (t, $J_{H,H}$ = 7.3 Hz, 9H), 0.92 (t, $J_{H,H}$ = 7.4 Hz, 3H), 0.987 (t, $J_{H,H}$ = 7.2 Hz, 3H), 0.993 (t, $J_{H,H}$ = 8.4 Hz, $J_{H,^{119}Sn}$ = 50.7 Hz, 6H), 1.33 (sext, $J_{H,H}$ = 7.3 Hz, 6H), 1.33–1.41 (m, 2H), 1.44–1.52 (m, 8H), 2.15 (q, $J_{H,H}$ = 7.4 Hz, 2H), 2.33 (t, $J_{H,H}$ = 8.0 Hz, 2H), 5.28 (t, $J_{H,H}$ = 7.0 Hz, 1H), 7.28 (s, $J_{H,^{119}Sn}$ = 16.4 Hz, 1H), 7.34 (d, $J_{H,H}$ = 7.2 Hz, 1H), 7.43 (d, $J_{H,H}$ = 7.1 Hz, $J_{H,^{119}Sn}$ = 43.0 Hz, 1H); **^{13}C NMR** (125 MHz, $CDCl_3$) δ –1.1, 10.8 ($J_{C,^{119}Sn}$ = 339.0 Hz, $J_{C,^{117}Sn}$ = 323.9 Hz), 13.6, 14.2, 14.4, 21.6, 23.0, 27.5 ($J_{C,^{119}Sn}$ = 60.4 Hz), 29.2 ($J_{C,^{119}Sn}$ = 18.5 Hz), 30.6, 35.0, 129.5, 130.5, 132.6, 136.3, 139.1, 141.6, 144.5, 151.8. **HRMS** (ESI) calcd for $C_{29}H_{54}NaSi^{120}Sn$ $[M+Na]^+$ 573.2914, found 573.2917.

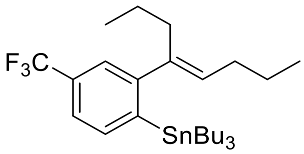

3ra

Compound 3ra. (Table 2, entry 22, 67% yield, colorless oil, R_f = 0.9 (hexanes)). **^{1}H NMR** (400 MHz, $CDCl_3$) δ 0.89 (t, $J_{H,H}$ = 7.3 Hz, 9H), 0.91 (t, $J_{H,H}$ = 7.4 Hz, 3H), 0.98 (t, $J_{H,H}$ = 7.4 Hz, 3H), 1.02 (t, $J_{H,H}$ = 8.2 Hz, $J_{H,^{119}Sn}$ = 50.0 Hz, 6H), 1.32 (sext, $J_{H,H}$ = 7.3 Hz, 6H), 1.26–1.42 (m, 2H), 1.42–1.51 (m, 8H), 2.15 (q, $J_{H,H}$ = 7.4 Hz, 2H), 2.32 (t, $J_{H,H}$ = 8.0 Hz, 2H), 5.29 (t, $J_{H,H}$ = 7.0 Hz, 1H), 7.34 (s, 1H), 7.40 (d, $J_{H,H}$ = 7.6 Hz, 1H), 7.53 (d, $J_{H,H}$ = 7.6 Hz, $J_{H,^{119}Sn}$ = 40.0 Hz, 1H); **^{13}C NMR** (125 MHz, $CDCl_3$) δ 10.9 ($J_{C,^{119}Sn}$ = 343.1 Hz, $J_{C,^{117}Sn}$ = 328.1 Hz), 13.6, 14.1, 14.4, 21.6, 22.9, 27.4 ($J_{C,^{119}Sn}$ = 61.7 Hz), 29.1 ($J_{C,^{119}Sn}$ = 19.2 Hz), 30.6, 34.8, 121.8 (q, $J_{C,^{19}F}$ = 3.7 Hz, $J_{C,^{119}Sn}$ = 41.0 Hz), 124.1 (q, $J_{C,^{19}F}$ = 3.6 Hz, $J_{C,^{119}Sn}$ = 34.0 Hz), 124.4 (q, $J_{C,^{19}F}$ = 272.2 Hz), 129.6 (q, $J_{C,^{19}F}$ = 31.8 Hz), 130.6, 137.1 ($J_{C,^{119}Sn}$ = 33.0 Hz), 143.4 ($J_{C,^{119}Sn}$ = 13.0 Hz), 146.3, 153.2 ($J_{C,^{119}Sn}$ = 28.2 Hz). **HRMS** (ESI) calcd for $C_{27}H_{45}F_3Na^{120}Sn$ $[M+Na]^+$ 569.2393, found 569.2393.

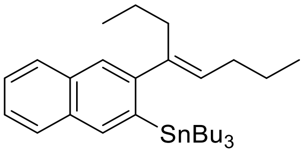

3sa

Compound 3sa. (Table 2, entry 23, 83% yield, colorless oil, R_f = 0.9 (hexanes)). **^{1}H NMR** (400 MHz, $CDCl_3$) δ 0.89 (t, $J_{H,H}$ = 7.3 Hz, 9H+3H), 1.00 (t, $J_{H,H}$ = 7.4 Hz, 3H), 1.06 (t, $J_{H,H}$ = 8.3 Hz, $J_{H,^{119}Sn}$ = 50.8 Hz, 6H), 1.34 (sext, $J_{H,H}$ = 7.3 Hz, 6H), 1.25–1.41 (m, 2H), 1.47–1.54 (m, 8H), 2.19 (q, $J_{H,H}$ = 7.4 Hz, 2H), 2.39 (t, $J_{H,H}$ = 7.9 Hz, 2H), 5.38 (t, $J_{H,H}$ = 7.0 Hz, 1H), 7.40–7.45 (m, 2H), 7.56 (s, $J_{H,^{119}Sn}$ = 14.3 Hz, 1H), 7.73–7.80 (m, 2H), 7.89 (s, $J_{H,^{119}Sn}$ = 48.3 Hz, 1H); **^{13}C NMR** (100 MHz, $CDCl_3$) δ 11.0 ($J_{C,^{119}Sn}$ = 340.0 Hz, $J_{C,^{117}Sn}$ = 325.2 Hz), 13.7, 14.2, 14.3, 21.6, 23.0, 27.5 ($J_{C,^{119}Sn}$ = 62.1 Hz), 29.2 ($J_{C,^{119}Sn}$ = 19.0 Hz), 30.6, 34.8, 125.2, 125.7, 125.9, 127.3, 127.5, 129.9, 131.7, 133.0, 137.1 ($J_{C,^{119}Sn}$ = 30.6 Hz), 140.0, 144.1($J_{C,^{119}Sn}$ = 11.6 Hz), 149.3. **HRMS** (ESI) calcd for $C_{30}H_{48}Na^{120}Sn$ $[M+Na]^+$ 551.2676, found 551.2660.

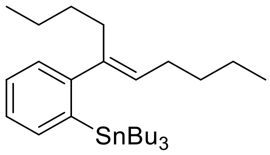

3ab

Compound 3ab. (Table 3, 84% yield, colorless oil, R_f = 0.9 (hexanes)). **^{1}H NMR** (400 MHz, $CDCl_3$) δ 0.88 (t, $J_{H,H}$ = 7.0 Hz, 3H), 0.89 (t, $J_{H,H}$ = 7.3 Hz, 9H), 0.94 (t, $J_{H,H}$ = 7.1 Hz, 3H), 0.99 (t, $J_{H,H}$ = 8.3 Hz, $J_{H,^{119}Sn}$ = 50.6 Hz, 6H), 1.28–1.52 (m, 20H), 2.16 (q, $J_{H,H}$ = 7.1 Hz, 2H), 2.34 (t, $J_{H,H}$ = 7.5 Hz,

2H), 5.25 (t, $J_{H,H}$ = 7.0 Hz, 1H), 7.16 (d, $J_{H,H}$ = 7.4 Hz, 1.3 Hz, 1H), 7.19 (td, $J_{H,H}$ = 7.1 Hz, 1.6 Hz, 1H), 7.24 (td, $J_{H,H}$ = 7.3 Hz, 1.7 Hz, 1H), 7.42 (dd, $J_{H,H}$ = 7.0 Hz, 1.3 Hz, $J_{H,^{119}Sn}$ = 43.0 Hz, 1H); **^{13}C NMR** (100 MHz, $CDCl_3$) δ 10.9 ($J_{C,^{119}Sn}$ = 339.7 Hz, $J_{C,^{117}Sn}$ = 324.5 Hz), 13.6, 13.9, 14.0, 22.6, 23.1, 27.5 ($J_{C,^{119}Sn}$ = 61.0 Hz), 28.1, 29.2 ($J_{C,^{119}Sn}$ = 18.9 Hz), 30.6, 32.0, 32.5, 125.6 ($J_{C,^{119}Sn}$ = 42.6 Hz), 127.5 ($J_{C,^{119}Sn}$ = 9.5 Hz), 127.9 ($J_{C,^{119}Sn}$ = 34.8 Hz), 129.6, 136.9 ($J_{C,^{119}Sn}$ = 30.5 Hz), 140.7 ($J_{C,^{119}Sn}$ = 413.7 Hz, $J_{C,^{117}Sn}$ = 395.4 Hz), 144.4 ($J_{C,^{119}Sn}$ = 14.4 Hz), 152.7 ($J_{C,^{119}Sn}$ = 28.3 Hz). **HRMS** (ESI) calcd for $C_{28}H_{50}Na^{120}Sn$ $[M+Na]^+$ 529.2832, found 529.2819.

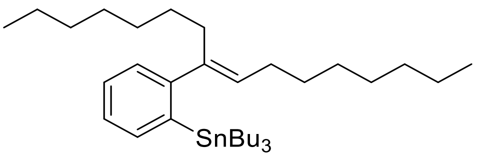

3ac

Compound 3ac. (Table 3, 83% yield, colorless oil, R_f = 0.8 (hexanes)). **^{1}H NMR** (400 MHz, $CDCl_3$) δ 0.89 (t, $J_{H,H}$ = 7.2 Hz, 9H), 0.85–0.92 (m, 6H), 0.99 (t, $J_{H,H}$ = 8.3 Hz, $J_{H,^{119}Sn}$ = 50.8 Hz, 6H), 1.18–1.40 (m, 24H), 1.42–1.54 (m, 8H), 2.15 (q, $J_{H,H}$ = 7.2 Hz, 2H), 2.33 (t, $J_{H,H}$ = 7.5 Hz, 2H), 5.25 (t, $J_{H,H}$ = 7.0 Hz, 1H), 7.16 (d, $J_{H,H}$ = 7.4 Hz, 1H), 7.19 (td, $J_{H,H}$ = 7.1 Hz, 1.5 Hz, 1H), 7.24 (td, $J_{H,H}$ = 7.3 Hz, 1.6 Hz, 1H), 7.42 (dd, $J_{H,H}$ = 7.0 Hz, 1.5 Hz, $J_{H,^{119}Sn}$ = 42.8 Hz, 1H); **^{13}C NMR** (100 MHz, $CDCl_3$) δ 10.9 ($J_{C,^{119}Sn}$ = 339.6 Hz, $J_{C,^{117}Sn}$ = 324.6 Hz), 13.7, 14.06, 14.09, 22.6, 22.7, 27.5 ($J_{C,^{119}Sn}$ = 62.1 Hz), 28.5, 29.16, 29.17, 29.3, 29.6, 29.8, 30.0, 31.9, 32.8, 125.6 ($J_{C,^{119}Sn}$ = 42.3 Hz), 127.4 ($J_{C,^{119}Sn}$ = 9.4 Hz), 127.9 ($J_{C,^{119}Sn}$ = 34.9 Hz), 129.6, 136.9 ($J_{C,^{119}Sn}$ = 32.4 Hz), 140.7 ($J_{C,^{119}Sn}$ = 414.0 Hz, $J_{C,^{117}Sn}$ = 395.7 Hz), 144.4 ($J_{C,^{119}Sn}$ = 14.5 Hz), 152.7 ($J_{C,^{119}Sn}$ = 28.1 Hz). **HRMS** (ESI) calcd for $C_{34}H_{62}Na^{120}Sn$ $[M+Na]^+$ 613.3771, found 613.3762.

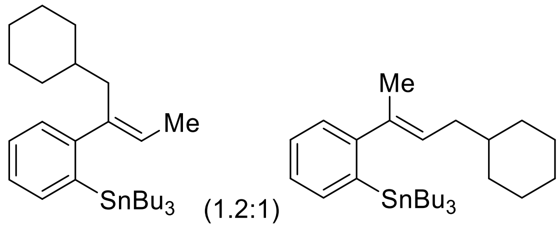

3ad **3ad'**

Compound 3ad:3ad' (1.2:1). (Table 3, 84% yield, colorless oil, R_f = 0.9 (hexanes)). **^{1}H NMR** (400 MHz, $CDCl_3$) for **3ad** δ 0.88 (t, $J_{H,H}$ = 7.3 Hz, 9H), 0.83–0.91 (m, 2H), 0.98 (t, $J_{H,H}$ = 8.3 Hz, 6H), 1.32 (sext, $J_{H,H}$ = 7.3 Hz, 6H), 1.10–1.41 (m, 4H), 1.43–1.60 (m, 6H), 1.73 (d, $J_{H,H}$ = 6.8 Hz, 3H), 1.60–1.83 (m, 5H), 2.22 (d, $J_{H,H}$ = 7.1 Hz, 2H), 5.39 (q, $J_{H,H}$ = 6.8 Hz, 1H), 7.10 (dd, $J_{H,H}$ = 7.4 Hz, 1.3 Hz, 1H), 7.16–7.29 (m, 2H), 7.41 (dd, $J_{H,H}$ = 6.9 Hz, 1.6 Hz, 1H); **3ad'** δ 0.89 (t, $J_{H,H}$ = 7.3 Hz, 9H), 0.83–0.91 (m, 2H), 0.97 (t, $J_{H,H}$ = 8.3 Hz, 6H), 1.32 (sext, $J_{H,H}$ = 7.3 Hz, 6H), 1.10–1.40 (m, 4H), 1.43–1.60 (m, 6H), 1.60–1.83 (m, 5H), 1.96 (s, 3H), 2.03 (t, $J_{H,H}$ = 7.0 Hz, 2H), 5.31 (td, $J_{H,H}$ = 7.3 Hz, 1.4 Hz, 1H), 7.16–7.29 (m, 3H), 7.43 (dd, $J_{H,H}$ = 7.0 Hz, 1.4 Hz, 1H); **^{13}C NMR** (100 MHz, $CDCl_3$) for **3ad** δ

10.7 ($J_{C,^{119}Sn}$ = 339.4 Hz, $J_{C,^{117}Sn}$ = 323.9 Hz), 13.6, 14.1, 26.3, 26.6, 27.5 ($J_{C,^{119}Sn}$ = 60.7 Hz), 29.2 ($J_{C,^{119}Sn}$ = 19.0 Hz), 33.6, 36.4, 38.4, 125.5, 127.0, 127.5 ($J_{C,^{119}Sn}$ = 10.0 Hz), 128.2, 136.8, 140.5, 143.7, 153.2; **3ad'** δ 10.8 ($J_{C,^{119}Sn}$ = 340.9 Hz, $J_{C,^{117}Sn}$ = 326.5 Hz), 13.6, 18.9, 26.4, 26.6, 27.5 ($J_{C,^{119}Sn}$ = 60.7 Hz), 29.2 ($J_{C,^{119}Sn}$ = 19.0 Hz), 33.4, 36.6, 40.1, 124.6, 125.8, 127.6 ($J_{C,^{119}Sn}$ = 10.0 Hz), 128.3, 137.0, 140.4, 140.7, 154.0. **HRMS** (ESI) calcd for $C_{28}H_{48}Na^{120}Sn$ $[M+Na]^+$ 527.2676, found 527.2703.

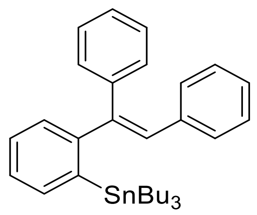

3ae

Compound 3ae. (Table 3, 72% yield, colorless oil, R_f = 0.8 (hexanes)). **^{1}H NMR** (400 MHz, $CDCl_3$) δ 0.83 (t, $J_{H,H}$ = 7.3 Hz, 9H), 0.98 (t, $J_{H,H}$ = 8.3 Hz, $J_{H,^{119}Sn}$ = 50.6 Hz, 6H), 1.27 (sext, $J_{H,H}$ = 7.3 Hz, 6H), 1.4–1.5 (m, 6H), 6.58 (s, 1H), 7.01 (dd, $J_{H,H}$ = 7.6 Hz, 1.0 Hz, 1H), 7.05–7.30 (m, 12H), 7.54 (dd, $J_{H,H}$ = 7.2 Hz, 1.1 Hz, $J_{H,^{119}Sn}$ = 41.6 Hz, 1H); **^{13}C NMR** (100 MHz, $CDCl_3$) δ 11.0 ($J_{C,^{119}Sn}$ = 342.2 Hz, $J_{C,^{117}Sn}$ = 327.0 Hz), 13.6, 27.4 ($J_{C,^{119}Sn}$ = 61.6 Hz), 29.2 ($J_{C,^{119}Sn}$ = 18.9 Hz), 126.7, 126.8, 127.5, 127.6 ($J_{C,^{119}Sn}$ = 9.3 Hz), 128.0, 128.2, 129.4, 129.5, 129.6, 130.6, 137.2, 137.4, 140.8, 142.0, 146.3 ($J_{C,^{119}Sn}$ = 14.3 Hz), 152.4 ($J_{C,^{119}Sn}$ = 25.1 Hz). **HRMS** (ESI) calcd for $C_{32}H_{42}Na^{120}Sn$ $[M+Na]^+$ 569.2206, found 569.2222.

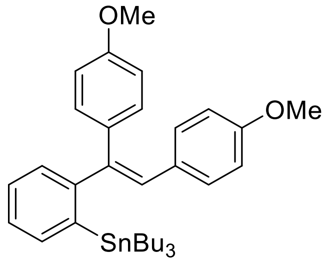

3af

Compound 3af. (Table 3, 78% yield, colorless oil, R_f = 0.3 (hexanes)). **^{1}H NMR** (400 MHz, $CDCl_3$) δ 0.83 (t, $J_{H,H}$ = 7.3 Hz, 9H), 0.96 (t, $J_{H,H}$ = 7.3 Hz, $J_{H,^{119}Sn}$ = 50.6 Hz, 6H), 1.26 (sext, $J_{H,H}$ = 7.3 Hz, 6H), 1.40–1.48 (m, 6H), 3.78 (s, 3H), 3.81 (s, 3H), 6.43 (s, 1H), 6.72 (d, $J_{H,H}$ = 8.8 Hz, 2H), 6.78 (d, $J_{H,H}$ = 8.8 Hz, 2H), 7.00 (dd, $J_{H,H}$ = 7.6 Hz, 1.1 Hz, 1H), 7.06 (d, $J_{H,H}$ = 8.6 Hz, 2H), 7.11 (d, $J_{H,H}$ = 8.8 Hz, 2H), 7.19 (td, $J_{H,H}$ = 7.4 Hz, 1.5 Hz, 1H), 7.24 (td, $J_{H,H}$ = 7.2 Hz, 1.4 Hz, 1H), 7.52 (dd, $J_{H,H}$ = 7.2 Hz, 1.1 Hz, $J_{H,^{119}Sn}$ = 41.7 Hz, 1H); **^{13}C NMR** (100 MHz, $CDCl_3$) δ 11.0 ($J_{C,^{119}Sn}$ = 341.9 Hz, $J_{C,^{117}Sn}$ = 326.7 Hz), 13.6, 27.5 ($J_{C,^{119}Sn}$ = 61.7 Hz), 29.2 ($J_{C,^{119}Sn}$ = 18.9 Hz), 55.1, 55.2, 113.4, 113.6, 126.5, 127.5 ($J_{C,^{119}Sn}$ = 9.4 Hz), 128.3, 129.5, 130.2, 130.6, 131.8, 133.3, 137.3 ($J_{C,^{119}Sn}$ = 32.1 Hz), 142.0, 144.1 ($J_{C,^{119}Sn}$ = 14.6 Hz), 152.8, 158.3, 158.9. **HRMS** (ESI) calcd for $C_{34}H_{46}O_2Na^{120}Sn$ $[M+Na]^+$ 629.2417, found 629.2411.

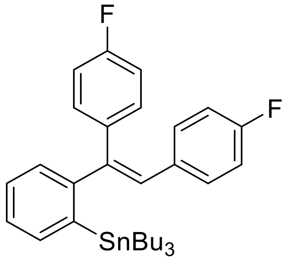

3ag

Compound 3ag. (Table 3, 74% yield, colorless solid, R_f = 0.7 (hexanes)). **^{1}H NMR** (400 MHz, $CDCl_3$) δ 0.83 (t, $J_{H,H}$ = 7.3 Hz, 9H), 0.96 (t, $J_{H,H}$ = 8.3 Hz, $J_{H,^{119}Sn}$ = 52.1 Hz, 6H), 1.26 (sext, $J_{H,H}$ = 7.3 Hz, 6H), 1.40–1.48 (m, 6H), 6.51 (s, 1H), 6.88 (t, $J_{H,H}$ = $J_{H,^{19}F}$ = 8.7 Hz, 2H), 6.94 (t, $J_{H,H}$ = $J_{H,^{19}F}$ = 8.7 Hz, 2H), 6.97 (dd, $J_{H,H}$ = 7.1 Hz, 1.0 Hz, 1H), 7.06 (dd, $J_{H,H}$ = 8.6 Hz, $J_{H,^{19}F}$ = 5.5 Hz, 2H), 7.13 (dd, $J_{H,H}$ = 8.7 Hz, $J_{H,^{19}F}$ = 5.5 Hz, 2H), 7.20 (td, $J_{H,H}$ = 7.5 Hz, 1.5 Hz, 1H), 7.27 (td, $J_{H,H}$ = 7.3 Hz, 1.4 Hz, 1H), 7.54 (dd, $J_{H,H}$ = 7.2 Hz, 1.2 Hz, $J_{H,^{119}Sn}$ = 41.5 Hz, 1H); **^{13}C NMR** (100 MHz, $CDCl_3$) δ 11.1 ($J_{C,^{119}Sn}$ = 343.4 Hz, $J_{C,^{117}Sn}$ = 326.9 Hz), 13.6, 27.4 ($J_{C,^{119}Sn}$ = 61.8 Hz), 29.1 ($J_{C,^{119}Sn}$ = 18.9 Hz), 115.2 (d, $J_{C,^{19}F}$ = 21.3 Hz), 115.4 (d, $J_{C,^{19}F}$ = 21.4 Hz), 126.9, 127.7, 128.4, 129.5, 130.9 (d, $J_{C,^{19}F}$ = 7.9 Hz), 132.3 (d, $J_{C,^{19}F}$ = 7.9 Hz), 133.2 (d, $J_{C,^{19}F}$ = 3.2 Hz), 136.4 (d, $J_{C,^{19}F}$ = 2.6 Hz), 137.5, 142.0, 145.1, 151.9, 161.6 (d, $J_{C,^{19}F}$ = 247.4 Hz), 162.3 (d, $J_{C,^{19}F}$ = 247.7 Hz). **HRMS** (ESI) calcd for $C_{32}H_{40}F_2Na^{120}Sn$ $[M+Na]^+$ 605.2018, found 605.2023. The structure of compound **3ag** was confirmed by single crystal X-ray diffraction (Section 9).

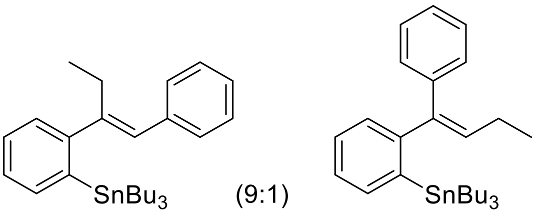

3ah **3ah'**

Compound 3ah:3ah' (9:1). (Table 3, 73% yield, colorless oil, R_f = 0.8 (hexanes)). **^{1}H NMR** (400 MHz, $CDCl_3$) for **3ah** δ 0.83 (t, $J_{H,H}$ = 7.3 Hz, 9H), 1.00 (t, $J_{H,H}$ = 8.4 Hz, 6H), 1.04 (t, $J_{H,H}$ = 7.8 Hz, 3H), 1.26 (sext, $J_{H,H}$ = 7.3 Hz, 6H), 1.40–1.50 (m, 6H), 2.68 (q, $J_{H,H}$ = 7.5 Hz, 2H), 6.33 (s, 1H), 7.15–7.38 (m, 8H), 7.49 (d, $J_{H,H}$ = 7.0 Hz, $J_{H,^{119}Sn}$ = 42.7 Hz, 1H), (**3ah'** δ 1.12 (t, $J_{H,H}$ = 7.5 Hz, 3H), 2.31 (quint, $J_{H,H}$ = 7.5 Hz, 2H), 5.63 (t, $J_{H,H}$ = 7.5 Hz, 1H)); **^{13}C NMR** (100 MHz, $CDCl_3$) for **3ah** δ 11.0 ($J_{C,^{119}Sn}$ = 338.4 Hz, $J_{C,^{117}Sn}$ = 323.7 Hz), 12.9, 13.6, 25.9, 27.4 ($J_{C,^{119}Sn}$ = 61.2 Hz), 29.1 ($J_{C,^{119}Sn}$ = 18.8 Hz), 126.2, 126.5, 127.4, 127.6, 128.2, 128.7, 129.9, 137.3, 137.8, 141.1, 148.4, 151.7. **HRMS** (ESI) calcd for $C_{26}H_{46}Na^{120}Sn$ $[M+Na]^+$ 521.2206, found 521.2206.

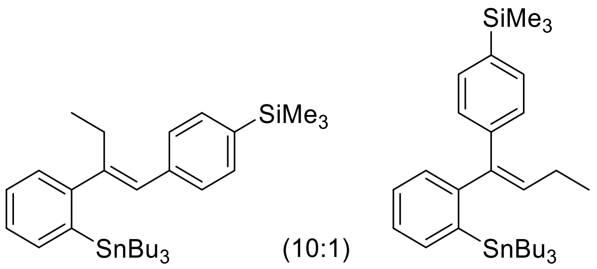

3ai 3ai'

Compound 3ai:3ai' (10:1). (Table 3, 77% yield, colorless oil, R_f = 0.8 (hexanes)). **^{1}H NMR** (400 MHz, $CDCl_3$) for **3ai** δ 0.29 (s, 9H), 0.83 (t, $J_{H,H}$ = 7.3 Hz, 9H), 1.01 (t, $J_{H,H}$ = 8.4 Hz, $J_{H,^{119}Sn}$ = 50.3 Hz, 6H), 1.05 (t, $J_{H,H}$ = 7.5 Hz, 3H), 1.28 (sext, $J_{H,H}$ = 7.3 Hz, 6H), 1.41–1.50 (m, 6H), 2.70 (q, $J_{H,H}$ = 7.5 Hz, 2H), 6.33 (s, 1H), 7.23–7.30 (m, 3H), 7.32 (d, $J_{H,H}$ = 7.8 Hz, 2H), 7.50 (d, $J_{H,H}$ = 7.0 Hz, $J_{H,^{119}Sn}$ = 41.6 Hz, 1H), 7.53 (d, $J_{H,H}$ = 8.0 Hz, 2H), (**3ai'** δ 0.26 (s, 9H), 1.13 (t, $J_{H,H}$ = 7.5 Hz, 3H), 2.34 (quint, $J_{H,H}$ = 7.5 Hz, 2H), 5.63 (t, $J_{H,H}$ = 7.5 Hz, 1H)); **^{13}C NMR** (100 MHz, $CDCl_3$) for **3ai** δ –1.1, 11.1 ($J_{C,^{119}Sn}$ = 339.9 Hz, $J_{C,^{117}Sn}$ = 325.0 Hz), 12.9, 13.6, 26.0, 27.4 ($J_{C,^{119}Sn}$ = 61.9 Hz), 29.1 ($J_{C,^{119}Sn}$ = 19.0 Hz), 126.2 ($J_{C,^{119}Sn}$ = 40.5 Hz), 127.4, 127.6 ($J_{C,^{119}Sn}$ = 9.5 Hz), 128.0, 128.7, 133.3, 137.3 ($J_{C,^{119}Sn}$ = 31.6 Hz), 138.2, 138.5, 141.1, 148.6 ($J_{C,^{119}Sn}$ = 13.9 Hz), 151.7. **HRMS** (ESI) calcd for $C_{31}H_{50}NaSi^{120}Sn$ $[M+Na]^+$ 593.2601, found 593.2617.

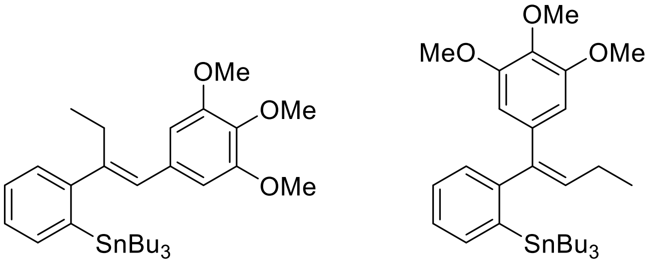

3aj 3aj'

Compound 3aj:3aj' (17:1). (Table 3, 76% yield, colorless oil, R_f = 0.3 (hexanes)). **^{1}H NMR** (400 MHz, $CDCl_3$) for **3aj** δ 0.84 (t, $J_{H,H}$ = 7.3 Hz, 9H), 1.02 (t, $J_{H,H}$ = 8.4 Hz, 6H), 1.07 (t, $J_{H,H}$ = 7.5 Hz, 3H), 1.29 (sext, $J_{H,H}$ = 7.3 Hz, 6H), 1.43–1.51 (m, 6H), 2.71 (q, $J_{H,H}$ = 7.6 Hz, 2H), 3.87 (s, 6H), 3.88 (s, 3H), 6.27 (s, 1H), 6.57 (s, 2H), 7.20–7.37 (m, 3H), 7.49 (d, $J_{H,H}$ = 7.0 Hz, $J_{H,^{119}Sn}$ = 42.5 Hz, 1H), (**3aj'** δ 1.34 (t, $J_{H,H}$ = 7.5 Hz, 3H), 2.33 (quint, $J_{H,H}$ = 7.5 Hz, 2H), 5.60 (t, $J_{H,H}$ = 7.5 Hz, 1H)); **^{13}C NMR** (100 MHz, $CDCl_3$) for **3aj** δ 11.1 ($J_{C,^{119}Sn}$ = 340.4 Hz, $J_{C,^{117}Sn}$ = 325.2 Hz), 12.9, 13.6, 26.1, 27.5, 29.1 ($J_{C,^{119}Sn}$ = 18.7 Hz), 56.0, 60.9, 105.9, 126.3, 127.4, 127.6, 128.7, 133.5, 136.9, 137.3, 141.1, 148.1, 151.6, 153.0. **HRMS** (ESI) calcd for $C_{31}H_{48}O_3Na^{120}Sn$ $[M+Na]^+$ 611.2523, found 611.2550.

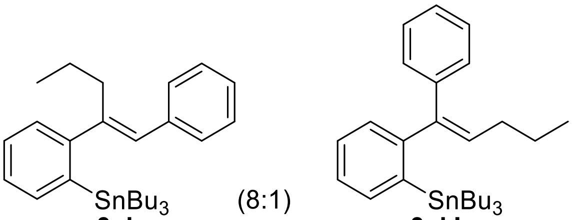

3ak **3ak'**

Compound 3ak:3ak' (8:1). (Table 3, 71% yield, colorless oil, R_f = 0.8 (hexanes)). **^{1}H NMR** (400 MHz, $CDCl_3$) for **3ak** δ 0.84 (t, $J_{H,H}$ = 7.3 Hz, 9H), 0.91 (t, $J_{H,H}$ = 7.4 Hz, 3H), 1.01 (t, $J_{H,H}$ = 8.4 Hz, $J_{H,^{119}Sn}$ = 51.4 Hz, 6H), 1.28 (sext, $J_{H,H}$ = 7.3 Hz, 6H), 1.41–1.50 (m, 8H), 2.61 (deformed t, $J_{H,H}$ = 8.2 Hz, 2H), 6.35 (s, 1H), 7.18–7.39 (m, 8H), 7.49 (d, $J_{H,H}$ = 7.0 Hz, $J_{H,^{119}Sn}$ = 41.8 Hz, 1H), (**3ak'** δ 2.28 (q, $J_{H,H}$ = 7.5 Hz, 2H), 5.68 (t, $J_{H,H}$ = 7.4 Hz, 1H)); **^{13}C NMR** (100 MHz, $CDCl_3$) for **3ak** δ 11.0 ($J_{C,^{119}Sn}$ = 340.1 Hz, $J_{C,^{117}Sn}$ = 325.1 Hz), 13.6, 14.5, 21.7, 27.4 ($J_{C,^{119}Sn}$ = 62.1 Hz), 29.1 ($J_{C,^{119}Sn}$ = 19.1 Hz), 35.3, 126.1, 126.5, 127.5, 127.6, 128.2, 128.7, 129.0, 137.2, 137.9, 140.8, 147.3, 152.2. **HRMS** (ESI) calcd for $C_{29}H_{44}Na^{120}Sn$ $[M+Na]^+$ 535.2363, found 535.2394.

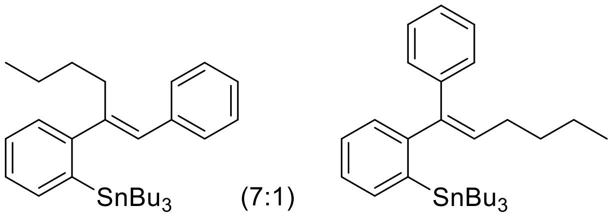

3al **3al'**

Compound 3al:3al' (7:1). (Table 3, 73% yield, colorless oil, R_f = 0.9 (hexanes)). **^{1}H NMR** (400 MHz, $CDCl_3$) for **3al** δ 0.84 (t, $J_{H,H}$ = 7.3 Hz, 9H), 0.87 (t, $J_{H,H}$ = 7.2 Hz, 3H), 1.01 (t, $J_{H,H}$ = 8.4 Hz, $J_{H,^{119}Sn}$ = 51.2 Hz, 6H), 1.24 (t, $J_{H,H}$ = 7.3 Hz, 6H), 1.27–1.35 (m, 2H), 1.39–1.51 (m, 8H), 2.64 (deformed t, $J_{H,H}$ = 8.1 Hz, 2H), 6.34 (s, 1H), 7.16–7.39 (m, 8H), 7.50 (d, $J_{H,H}$ = 7.0 Hz, $J_{H,^{119}Sn}$ = 41.9 Hz, 1H), (**3al'** δ 2.30 (q, $J_{H,H}$ = 7.5 Hz, 2H), 5.67 (t, $J_{H,H}$ = 7.4 Hz, 1H)); **^{13}C NMR** (100 MHz, $CDCl_3$) for **3al** δ 11.0 ($J_{C,^{119}Sn}$ = 340.3 Hz, $J_{C,^{117}Sn}$ = 325.1 Hz), 13.6, 13.9, 23.1, 27.4 ($J_{C,^{119}Sn}$ = 62.0 Hz), 29.1 ($J_{C,^{119}Sn}$ = 19.7 Hz), 30.6, 32.9, 126.2, 126.5, 127.5, 127.6, 128.2, 128.7, 128.9, 137.3, 137.9, 140.9, 147.4, 152.2. **HRMS** (ESI) calcd for $C_{26}H_{46}Na^{120}Sn$ $[M+Na]^+$ 549.2519, found 549.2546.

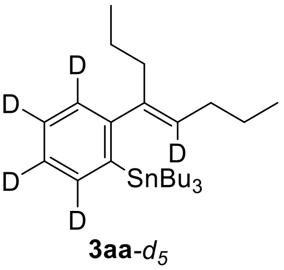

3aa-*d*5

Compound 3aa-*d*5. (equotion 1, 87% yield, colorless oil, R_f = 0.9 (hexanes)). **^{1}H NMR** (400 MHz, $CDCl_3$) δ 0.89 (t, $J_{H,H}$ = 7.1 Hz, 9H+3H), 0.96 (t, $J_{H,H}$ = 6.7 Hz, 3H), 0.99 (t, $J_{H,H}$ = 8.1 Hz, $J_{H,^{119}Sn}$ = 51.0 Hz, 6H), 1.32 (sext, $J_{H,H}$ = 7.1 Hz, 6H+2H), 1.42–1.51 (m, 8H), 2.14 (t, $J_{H,H}$ = 7.5 Hz, 2H), 2.32 (t, $J_{H,H}$ =

8.0 Hz, 2H); **^{13}C NMR** (100 MHz, $CDCl_3$) δ 10.8 ($J_{C,^{119}Sn}$ = 339.6 Hz, $J_{C,^{117}Sn}$ = 324.7 Hz), 13.6, 14.1, 14.4, 21.6, 22.9, 27.5 ($J_{C,^{119}Sn}$ = 61.0 Hz), 29.2 ($J_{C,^{119}Sn}$ = 18.9 Hz), 30.5, 34.9, 125.1 (t, $J_{C,^2H}$ = 23.8 Hz), 126.9 (t, $J_{C,^2H}$ = 23.9 Hz), 127.5 (t, $J_{C,^2H}$ = 23.2 Hz), 129.2 (t, $J_{C,^2H}$ = 22.8 Hz), 136.5 (t, $J_{C,^2H}$ = 23.9 Hz), 140.5, 144.2 ($J_{C,^{119}Sn}$ = 14.6 Hz), 152.6 ($J_{C,^{119}Sn}$ = 28.6 Hz); **^{2}H{^{1}H} NMR** (61 MHz, acetone) δ 5.26 (s, 1H), 7.19 (s, 2H), 7.32 (s, 1H), 7.43 (s, 1H). **HRMS** (ESI) calcd for $C_{26}H_{41}D_5Na^{120}Sn$ $[M+Na]^+$ 506.2833, found 506.2838.

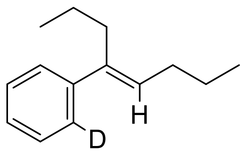

8

Compound 8 [374107-00-3]. (Scheme 3, 98% yield, colorless oil, R_f = 0.9 (hexanes)). **^{1}H NMR** (500 MHz, $CDCl_3$) δ 0.89 (t, $J_{H,H}$ = 7.4 Hz, 3H), 0.97 (t, $J_{H,H}$ = 7.4 Hz, 3H), 1.37 (sext, $J_{H,H}$ = 7.6 Hz, 2H), 1.48 (sext, $J_{H,H}$ = 7.4 Hz, 2H), 2.18 (q, $J_{H,H}$ = 7.3 Hz, 2H), 2.48 (t, $J_{H,H}$ = 7.7 Hz, 2H), 5.67 (t, $J_{H,H}$ = 7.3 Hz, 1H), 7.21 (td, $J_{H,H}$ = 7.4 Hz, 1.3 Hz, 1H), 7.30 (t, $J_{H,H}$ = 6.9 Hz, 1H), 7.30 (d, $J_{H,H}$ = 6.7 Hz, 1H), 7.34 (d, $J_{H,H}$ = 7.7 Hz, 1H); **^{13}C NMR** (100 MHz, $CDCl_3$) δ 13.9, 14.0, 21.8, 23.1, 30.6, 31.7, 126.0 (t, $J_{C,^2H}$ = 24.1 Hz), 126.3, 128.0, 128.1, 129.2, 140.0, 143.5. The spectral data are in agreement with reported literature values.[15]

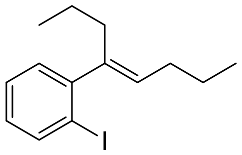

9

Compound 9. (Scheme 3, 96% yield, colorless oil, R_f = 0.8 (hexanes)). **^{1}H NMR** (400 MHz, $CDCl_3$) δ 0.98 (t, $J_{H,H}$ = 7.3 Hz, 3H), 1.07 (t, $J_{H,H}$ = 7.4 Hz, 3H), 1.39 (sext, $J_{H,H}$ = 7.8 Hz, 2H), 1.57 (sext, $J_{H,H}$ = 7.3 Hz, 2H), 2.25 (q, $J_{H,H}$ = 7.3 Hz, 2H), 2.46 (t, $J_{H,H}$ = 7.8 Hz, 2H), 5.37 (t, $J_{H,H}$ = 7.3 Hz, 1H), 6.95 (td, $J_{H,H}$ = 7.7 Hz, 1.7 Hz, 1H), 7.17 (dd, $J_{H,H}$ = 7.5 Hz, 1.7 Hz, 1H), 7.31 (td, $J_{H,H}$ = 7.5 Hz, 1.2 Hz, 1H), 7.89 (dd, $J_{H,H}$ = 7.9 Hz, 1.1 Hz, 1H); **^{13}C NMR** (100 MHz, $CDCl_3$) δ 14.0, 14.2, 21.2, 22.7, 30.0, 33.5, 99.4, 127.5, 127.8, 129.6, 131.3, 139.0, 143.3, 149.1. **HRMS** (ESI) calcd for $C_{14}H_{20}I$ $[M+H]^+$ 315.0610, found 315.0613.

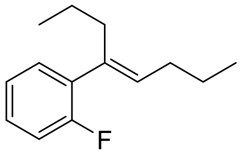

10

Compound 10 [1092526-15-2]. (Scheme 3, 69% yield, colorless oil, R_f = 0.9 (hexanes)). **^{1}H NMR** (400 MHz, $CDCl_3$) δ 0.86 (t, $J_{H,H}$ = 7.3 Hz, 3H), 0.96 (t, $J_{H,H}$ = 7.4 Hz, 3H), 1.30 (sext, $J_{H,H}$ = 7.4 Hz, 2H), 1.47 (sext, $J_{H,H}$ = 7.4 Hz, 2H), 2.18 (q, $J_{H,H}$ = 7.3 Hz, 2H), 2.44 (t, $J_{H,H}$ = 7.6 Hz, 2H), 5.50

(t, $J_{H,H}$ = 7.2 Hz, 1H), 6.99 (dd, $J_{H,H}$ = 8.4 Hz, $J_{H,^{19}F}$ = 10.6 Hz, 1H), 7.06 (dd, $J_{H,H}$ = 7.4 Hz, 1.2 Hz, 1H), 7.17 (d, $J_{H,H}$ = 7.2 Hz, 1H), 7.19–7.23 (m, 1H); ^{13}**C NMR** (100 MHz, $CDCl_3$) δ 13.8, 21.5, 22.9, 30.3, 32.5, 115.4 (d, $J_{C,^{19}F}$ = 23.1 Hz), 123.7 (d, $J_{C,^{19}F}$ = 3.2 Hz), 126.4, 127.9 (d, $J_{C,^{19}F}$ = 8.0 Hz), 130.6 (d, $J_{C,^{19}F}$ = 4.6 Hz), 131.9 (d, $J_{C,^{19}F}$ = 14.6 Hz), 136.1, 160.0 (d, $J_{C,^{19}F}$ = 245.8 Hz). The spectral data are in agreement with reported literature values.[16]

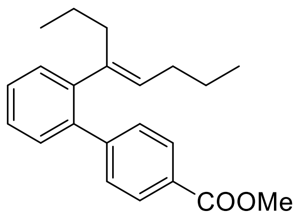

11

Compound 11. (Scheme 3, 83% yield, pale yellow oil, R_f= 0.4 (hexanes)). ^{1}**H NMR** (400 MHz, $CDCl_3$) δ 0.69 (t, $J_{H,H}$ = 7.3 Hz, 3H), 0.91 (t, $J_{H,H}$ = 7.4 Hz, 3H), 1.10 (sext, $J_{H,H}$ = 7.5 Hz, 2H), 1.40 (sext, $J_{H,H}$ = 7.3 Hz, 2H), 1.79 (t, $J_{H,H}$ = 7.7 Hz, 2H), 2.06 (q, $J_{H,H}$ = 7.3 Hz, 2H), 3.93 (s, 3H), 5.48 (t, $J_{H,H}$ = 7.3 Hz, 1H), 7.20–7.33 (m, 4H), 7.50 (d, $J_{H,H}$ = 8.3 Hz, 2H), 8.01 (d, $J_{H,H}$ = 8.3 Hz, 2H); ^{13}**C NMR** (100 MHz, $CDCl_3$) δ 13.9, 21.5, 22.8, 30.3, 32.9, 52.0, 126.8, 127.6, 128.3, 129.1, 129.2, 129.7, 130.4, 132.2, 138.6, 141.2, 143.6, 147.2, 167.2. **HRMS** (ESI) calcd for $C_{22}H_{27}O_2$ $[M+H]^+$ 323.2011, found 323.1995.

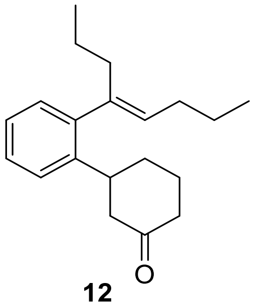

12

Compound 12. (Scheme 3, 87% yield, colorless oil, R_f= 0.4 (hexanes)). ^{1}**H NMR** (400 MHz, $CDCl_3$) δ 0.87 (t, $J_{H,H}$ = 7.3 Hz, 3H), 0.94 (t, $J_{H,H}$ = 7.4 Hz, 3H), 1.27 (sext d, $J_{H,H}$ = 7.8 Hz, 2.2 Hz, 2H), 1.43 (sext, $J_{H,H}$ = 7.3 Hz, 2H), 1.69 (qt, $J_{H,H}$ = 12.8 Hz, 4.5 Hz, 1H), 1.82 (qd, $J_{H,H}$ = 12.3 Hz, 3.2 Hz, 1H), 1.91 (br d, $J_{H,H}$ = 13.2 Hz, 1H), 2.14 (q, $J_{H,H}$ = 7.4 Hz, 2H), 2.11–2.20 (m, 1H), 2.22–2.31 (m, 2H), 2.38 (td, $J_{H,H}$ = 14.5 Hz, 6.6 Hz, 1H), 2.4–2.5 (m, 1H), 2.45 (br d, $J_{H,H}$ = 14.1 Hz, 1H), 2.53 (t, $J_{H,H}$ = 13.8 Hz, 1H), 3.23 (tt, $J_{H,H}$ = 12.2 Hz, 3.9 Hz, 1H), 5.21 (t, $J_{H,H}$ = 7.3 Hz, 1H), 7.05 (dd, $J_{H,H}$ = 7.5 Hz, 1.0 Hz, 1H), 7.15 (td, $J_{H,H}$ =7.3 Hz, 1.6 Hz, 1H), 7.25 (td, $J_{H,H}$ = 7.8 Hz, 1.2 Hz, 1H), 7.29 (dd, $J_{H,H}$ = 7.7 Hz, 1.4 Hz, 1H); ^{13}**C NMR** (100 MHz, $CDCl_3$) δ 13.9, 14.2, 21.3, 23.0, 25.9, 30.1, 33.4, 34.9, 40.6, 41.2, 49.3, 125.6, 125.8, 126.8, 129.5, 130.4, 139.6, 141.7, 143.8, 211.1. **HRMS** (ESI) calcd for $C_{20}H_{29}O$ $[M+H]^+$ 285.2218, found 285.2221.

10. Single crystal X-ray diffraction data for compound 3ag (CCDC 1841177)

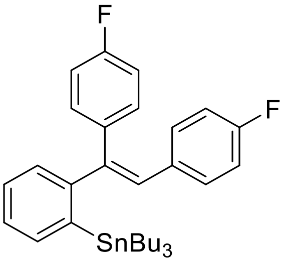

3ag

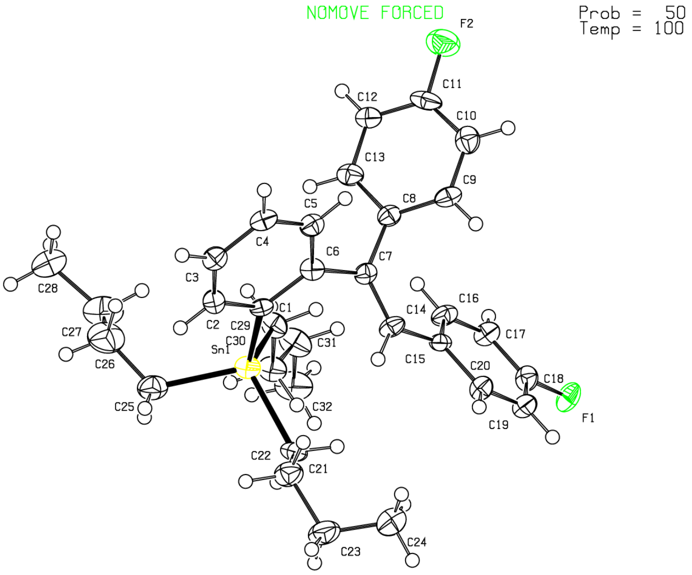

Table S1. Crystal data and structure refinement for compound 3ag

Empirical formula	$C_{32}H_{40}F_2Sn$	
Formula weight	581.33 g/mol	
Temperature	100(2) K	
Wavelength	0.71073 Å	
Crystal size	0.020 x 0.040 x 0.320 mm	
Crystal habit	colorless needle	
Crystal system	Monoclinic	
Space group	P 1 21/c 1	
Unit cell dimensions	a = 17.1715(13) Å	α = 90°.
	b = 17.8894(14) Å	β = 101.309(3)°.
	c = 9.4518(7) Å	γ = 90°.
Volume	2847.1(4) $Å^3$	
Z	4	
Density (calculated)	1.356 g/cm^3	
Absorption coefficient	0.928 mm^{-1}	
F(000)	1200	
Theta range for data collection	2.48 to 27.94°.	
Index ranges	–22<=h<=20, –22<=k<=23, –12<=l<=11	
Reflections collected	16876	
Independent reflections	6610 [R(int) = 0.0945]	

Coverage of independent reflections	96.5%	
Absorption correction	Multi-Scan	
Max. and min. transmission	0.9820 and 0.7560	
Structure solution technique	direct methods	
Structure solution program	XT, VERSION 2014/5	
Refinement method	Full-matrix least-squares on F^2	
Refinement program	SHELXL-2016/6 (Sheldrick, 2016)	
Function minimized	$\Sigma w(F_o^2 - F_c^2)^2$	
Data / restraints / parameters	6610 / 0 / 319	
Goodness-of-fit on F^2	1.014	
Final R indices	3523 data; $I>2\sigma(I)$	R1 = 0.0663, wR2 = 0.1362
	all data	R1 = 0.1468, wR2 = 0.1726
Weighting scheme	$w=1/[\sigma^2(F_o^2)+(0.0685P)^2]$	
	where $P=(F_o^2+2F_c^2)/3$	
Largest diff. peak and hole	1.424 and –1.680 eÅ^{-3}	
R.M.S. deviation from mean	0.155 eÅ^{-3}	

11. Reference

[1] A. van der Ent and A. L. Onderdelinden, *Inorg. Synth.*, 1990, **28**, 90.

[2] G. Giordano and R. H. Crabtree, *Inorg. Synth.*, 1979, **19**, 218.

[3] (a)T. Hayashi, M. Takahashi, Y. Takaya and M. Ogasawara, *J. Am. Chem. Soc.*, 2002. **124**, 5052. (b) G. E. Rudebusch, L. N. Zakharov and S. Y. Liu, *Angew. Chem. Int. Ed.*, 2013, **52**, 9316.

[4] P. Tang, T. Furuya and T. Ritter, *J. Am. Chem. Soc.,* 2010, **132**, 12150.

[5] Y. Horino, M. Sugata, I. Mutsuura, K. Tomohara and H. Abe, *Org. Lett.*, 2017, **19**, 5968.

[6] T. Kusakabe, Y. Ito, M. Kamimura, T. Shirai, K. Takahashi, T. Mochida and K. Kato, *Asian J. Org. Chem.*, 2017, **6**, 1086.

[7] M. B. Faraoni, L. C. Koll, S. D. Mandolesi, A. E. Zúñiga and J. C. Podestá, *J. Organomet. Chem.*, 2000, **613**, 236.

[8] (a) K. Mochida, *Bull. Chem. Soc. Jpn.*, 1987, **60**, 3299; (b) V. V. Sharutin, O. K. Sharutina, V. S. Senchurin, T. A. Kovaleva, V. I. Shcherbakov and E. N. Gladyshev, *Russ. J. Gen. Chem.*, 2000, **70**, 64.

[9] K. Komeyama, R. Asakura and K. Takaki, *Org. Biomol. Chem.,* 2015, **13**, 8713.

[10] A. Nagaki, Y. Tomida, H. Usutani, H. Kim, N. Takabayashi, T. Nokami, H. Okamoto and J. Yoshida, *Chem. Asian J.*, 2007, **2**, 1513.

[11] (a) D. E. Seitz, G. L. Tonnesen, S. Hellman, R. N. Hanson and S. J. Adelstein, *J. Organomet. Chem.*, 1980, **186**, C33; (b) Y. Gu and R. Martin, *Angew. Chem. Int. Ed.*, 2017, **56**, 3187.

[12] D. E. Seitz, R. A. Milius and H. El-Wakil, *Synth. Commun.*, 1981, **11**, 281.

[13] T. Furuya, A. E. Strom and T. Ritter, *J. Am. Chem. Soc.*, 2009, **131**, 1662.

[14] G. P. Roth, V. Farina, L. S. Liebeskind and E. Peña-Cabrera, *Tetrahedron Lett.*, 1995, ***36***, 2191.

[15] T. Hayashi, K. Inoue, N. Taniguchi and M. Ogasawara, *J. Am. Chem. Soc.*, 2001, **123**, 9918.

[16] Y. Nakao, N. Kashihara, K. S. Kanyiva and T. Hiyama, *J. Am. Chem. Soc.*, 2008, **130**, 16170.

12. NMR spectra

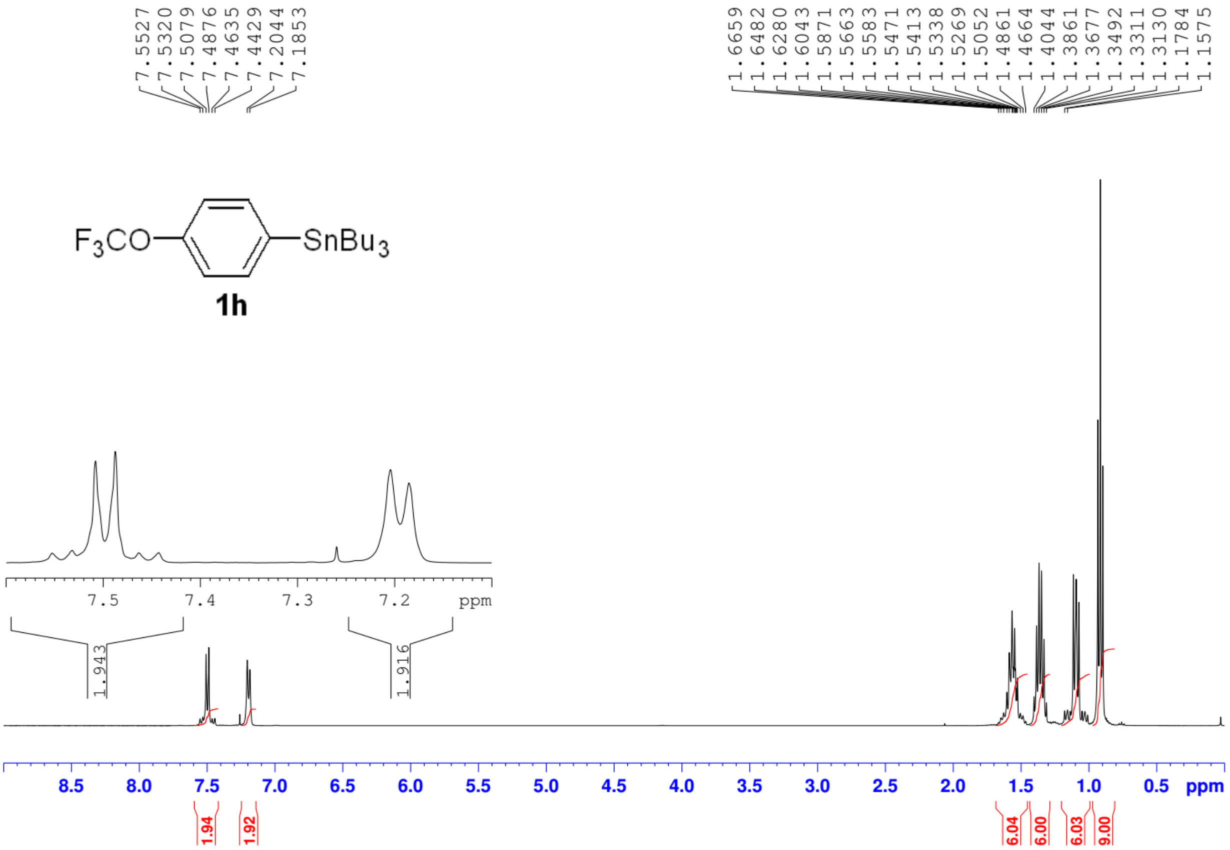

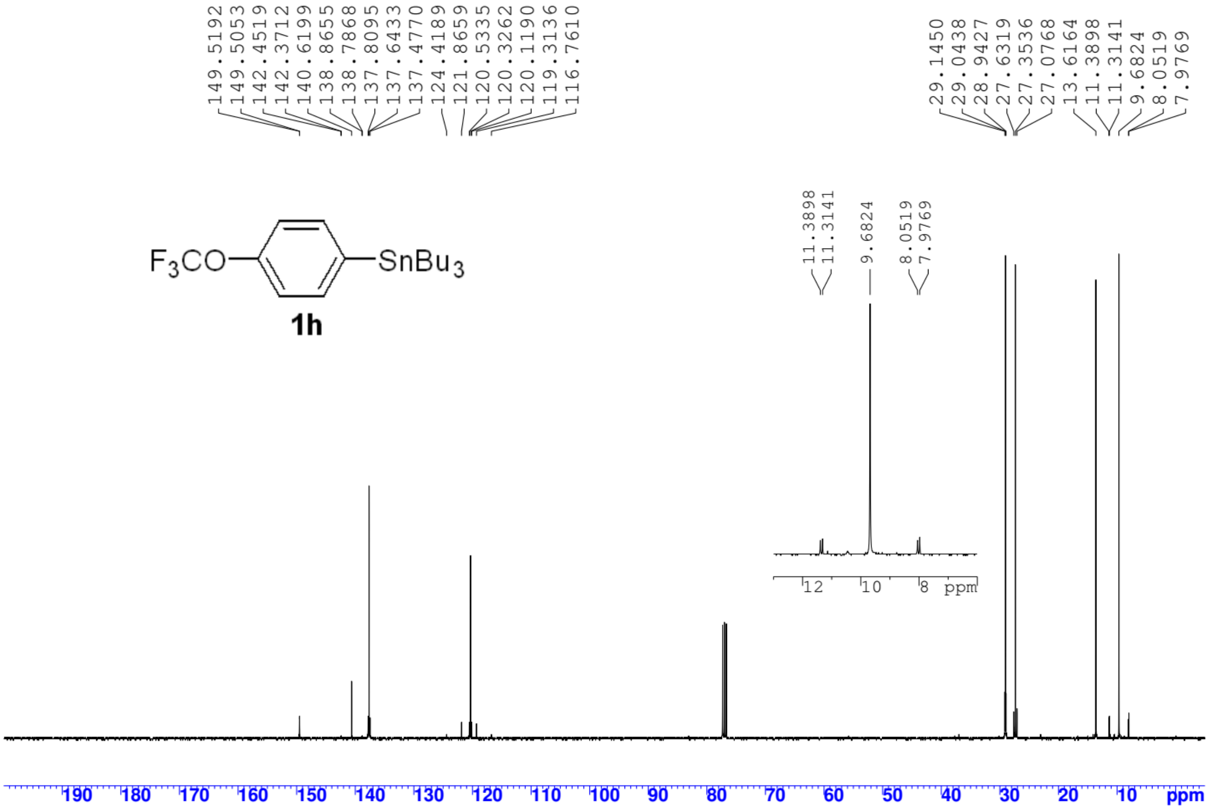

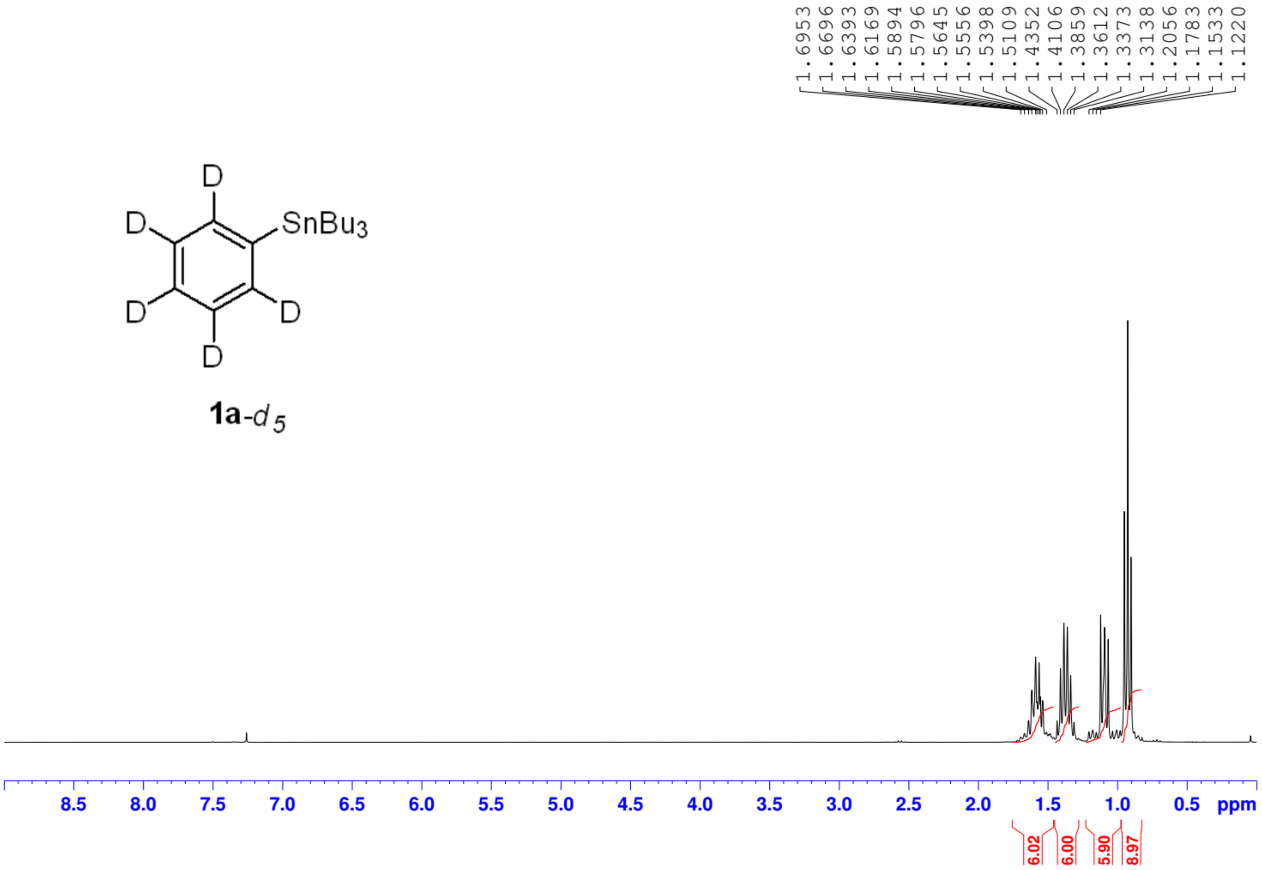
1.6953
1.6696
1.6393
1.6169
1.5894
1.5796
1.5645
1.5556
1.5398
1.5109
1.4352
1.4106
1.3859
1.3612
1.3373
1.3138
1.2056
1.1783
1.1533
1.1220
D
SnBu3
1a-d5
8.5 8.0 7.5 7.0 6.5 6.0 5.5 5.0 4.5 4.0 3.5 3.0 2.5 2.0 1.5 1.0 0.5 ppm
6.02
6.00
5.90
8.97

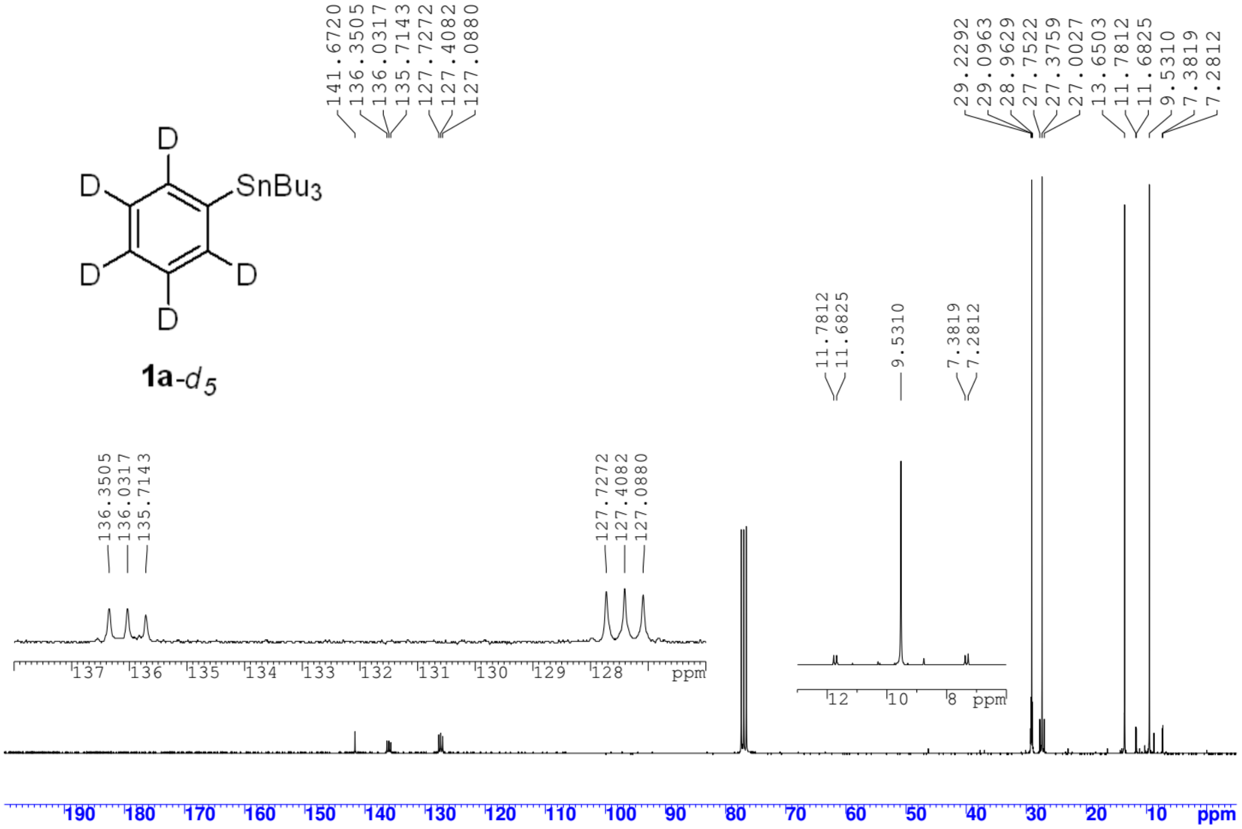
141.6720
136.3505
136.0317
135.7143
127.7272
127.4082
127.0880
29.2292
29.0963
28.9629
27.7522
27.3759
27.0027
13.6503
11.7812
11.6825
9.5310
7.3819
7.2812
D
SnBu3
1a-d5
11.7812
11.6825
9.5310
7.3819
7.2812
12 10 8 ppm
136.3505
136.0317
135.7143
127.7272
127.4082
127.0880
137 136 135 134 133 132 131 130 129 128 ppm
190 180 170 160 150 140 130 120 110 100 90 80 70 60 50 40 30 20 10 ppm

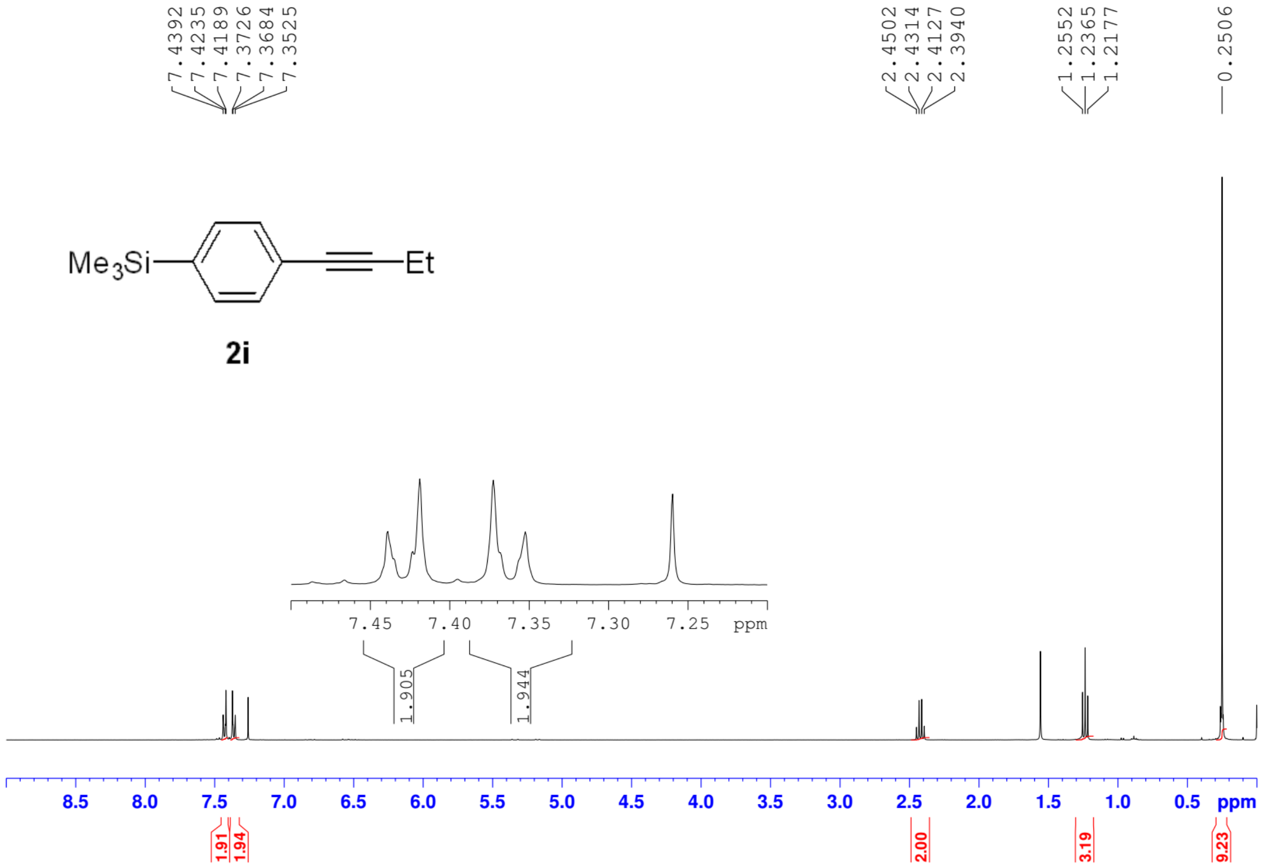
7.4392
7.4235
7.4189
7.3726
7.3684
7.3525
2.4502
2.4314
2.4127
2.3940
1.2552
1.2365
1.2177
0.2506
Me3Si
Et
2i
7.45 7.40 7.35 7.30 7.25 ppm
1.905
1.944
8.5 8.0 7.5 7.0 6.5 6.0 5.5 5.0 4.5 4.0 3.5 3.0 2.5 2.0 1.5 1.0 0.5 ppm
1.91
1.94
2.00
3.19
9.23

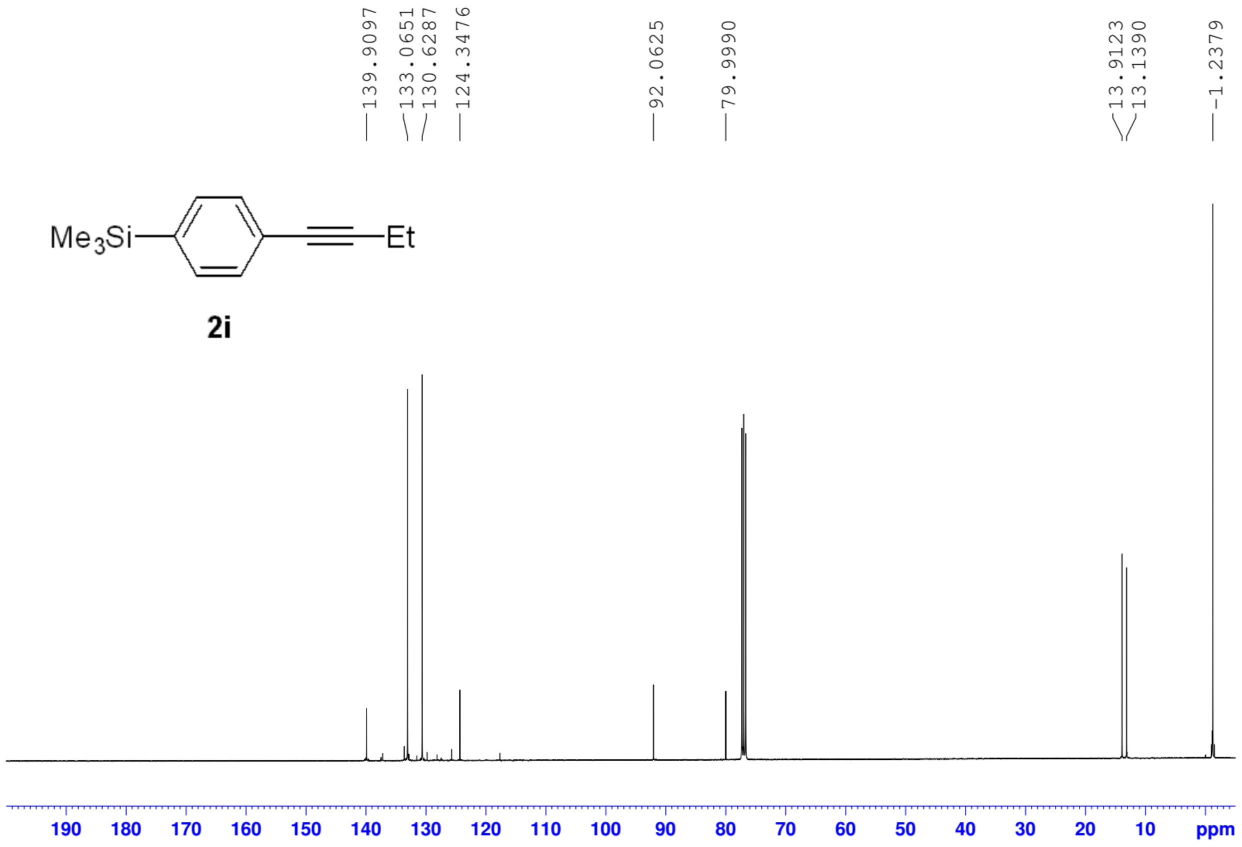
139.9097
133.0651
130.6287
124.3476
92.0625
79.9990
13.9123
13.1390
-1.2379
Me3Si
Et
2i
190 180 170 160 150 140 130 120 110 100 90 80 70 60 50 40 30 20 10 ppm

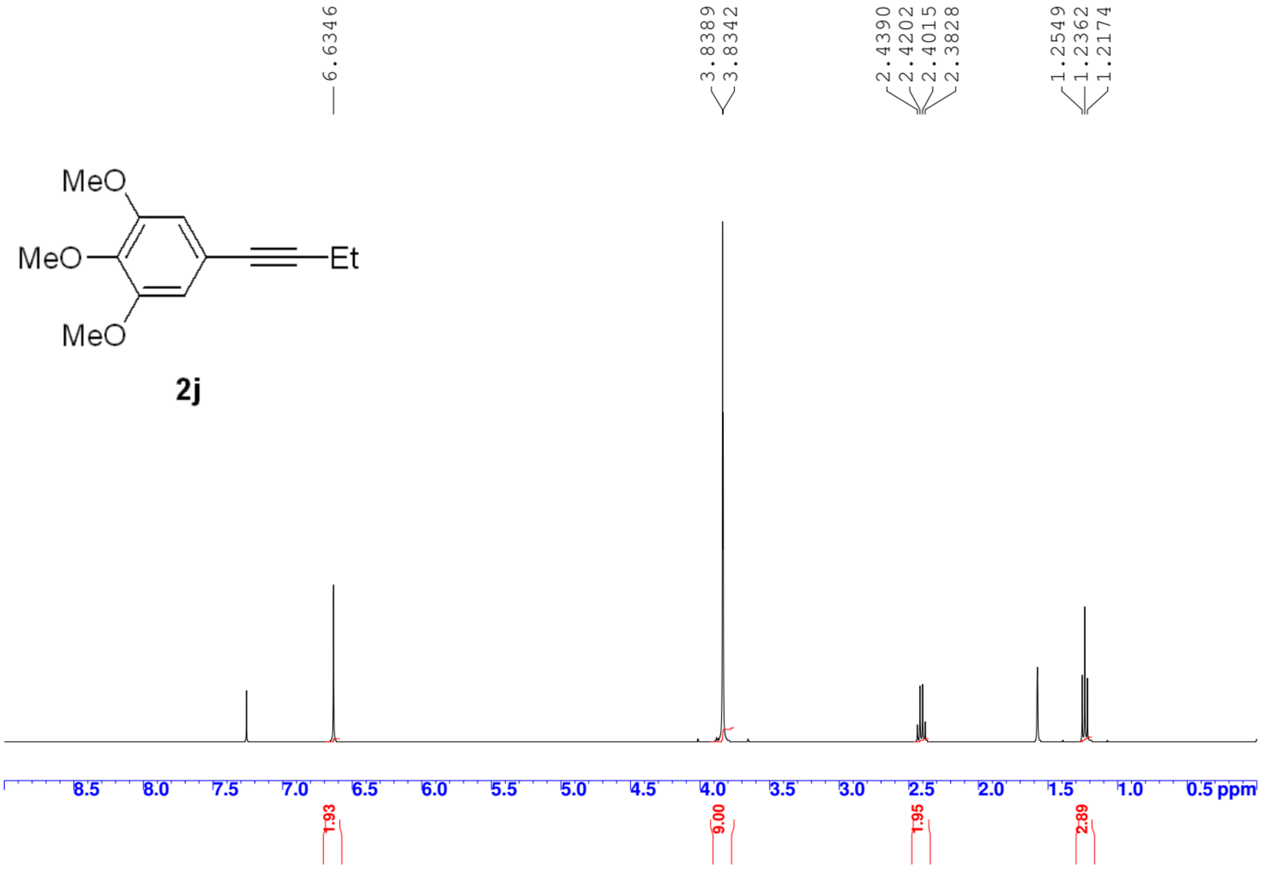

6.6346
3.8389
3.8342
2.4390
2.4202
2.4015
2.3828
1.2549
1.2362
1.2174
MeO
MeO
MeO
Et
2j
8.5
8.0
7.5
7.0
6.5
6.0
5.5
5.0
4.5
4.0
3.5
3.0
2.5
2.0
1.5
1.0
0.5 ppm
1.93
9.00
1.95
2.89

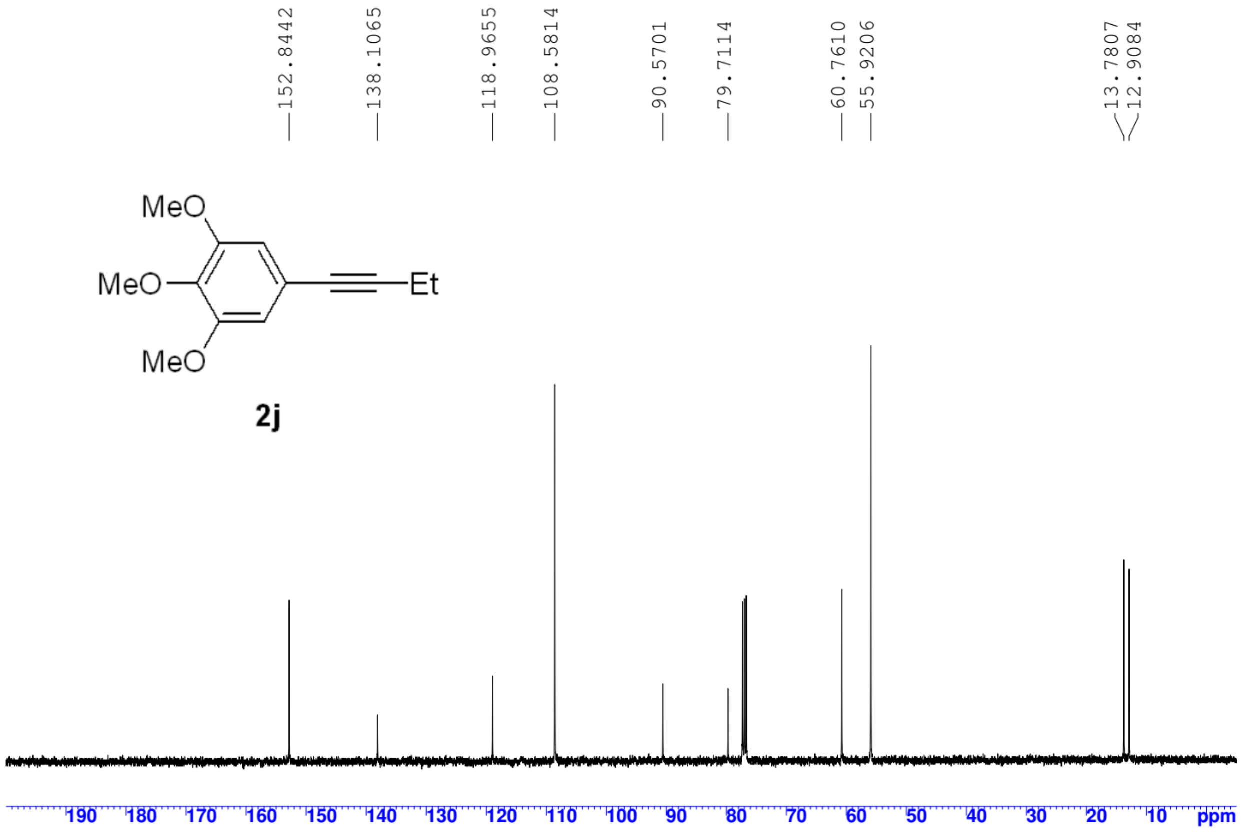

152.8442
138.1065
118.9655
108.5814
90.5701
79.7114
60.7610
55.9206
13.7807
12.9084
MeO
MeO
MeO
Et
2j
190
180
170
160
150
140
130
120
110
100
90
80
70
60
50
40
30
20
10
ppm

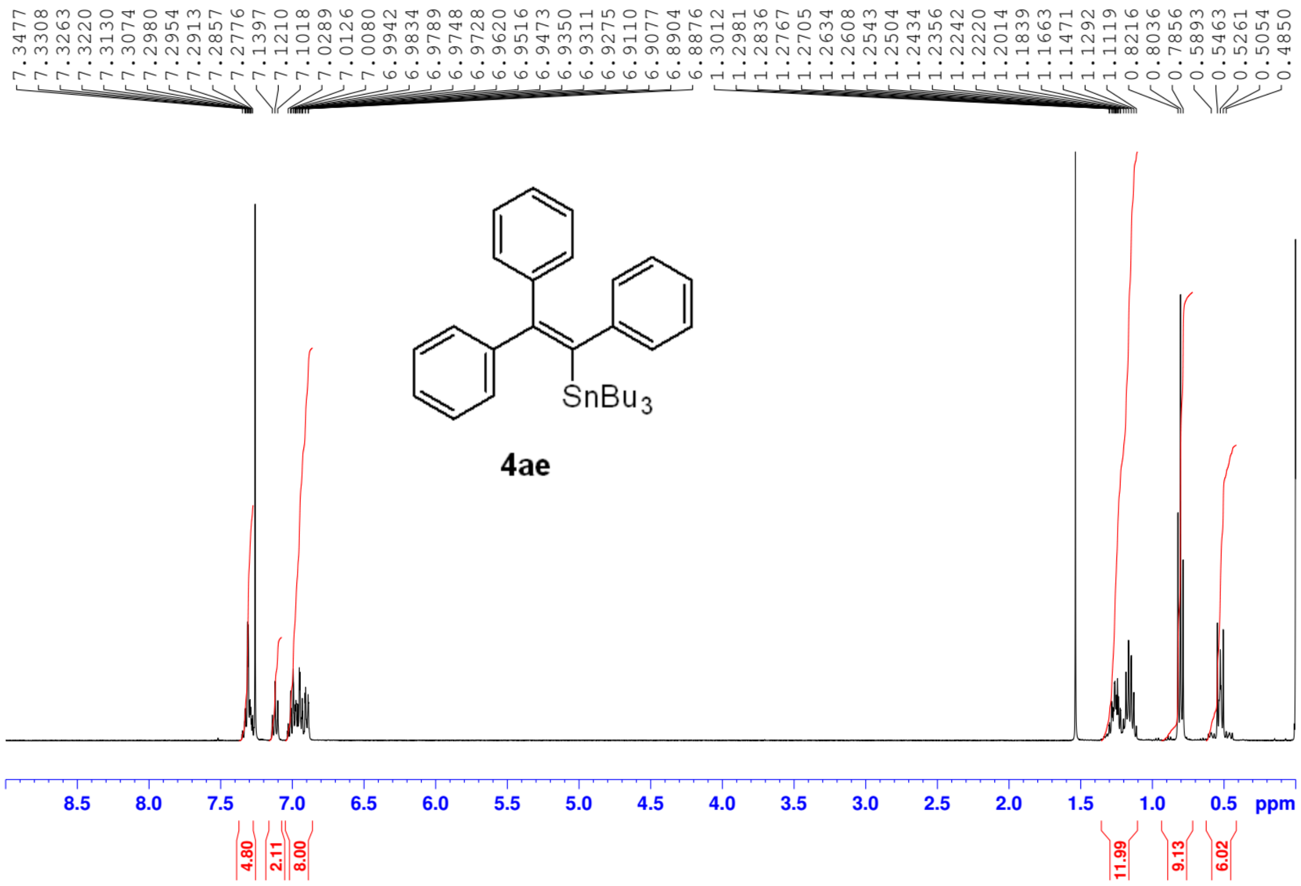
7.3477
7.3308
7.3263
7.3220
7.3130
7.3074
7.2980
7.2954
7.2913
7.2857
7.2776
7.1397
7.1210
7.1018
7.0289
7.0126
7.0080
6.9942
6.9834
6.9789
6.9748
6.9728
6.9620
6.9516
6.9473
6.9350
6.9311
6.9275
6.9110
6.9077
6.8904
6.8876
1.3012
1.2981
1.2836
1.2767
1.2705
1.2634
1.2608
1.2543
1.2504
1.2434
1.2356
1.2242
1.2220
1.2014
1.1839
1.1663
1.1471
1.1292
1.1119
0.8216
0.8036
0.7856
0.5893
0.5463
0.5261
0.5054
0.4850
SnBu3
4ae
8.5
8.0
7.5
7.0
6.5
6.0
5.5
5.0
4.5
4.0
3.5
3.0
2.5
2.0
1.5
1.0
0.5
ppm
4.80
2.11
8.00
11.99
9.13
6.02

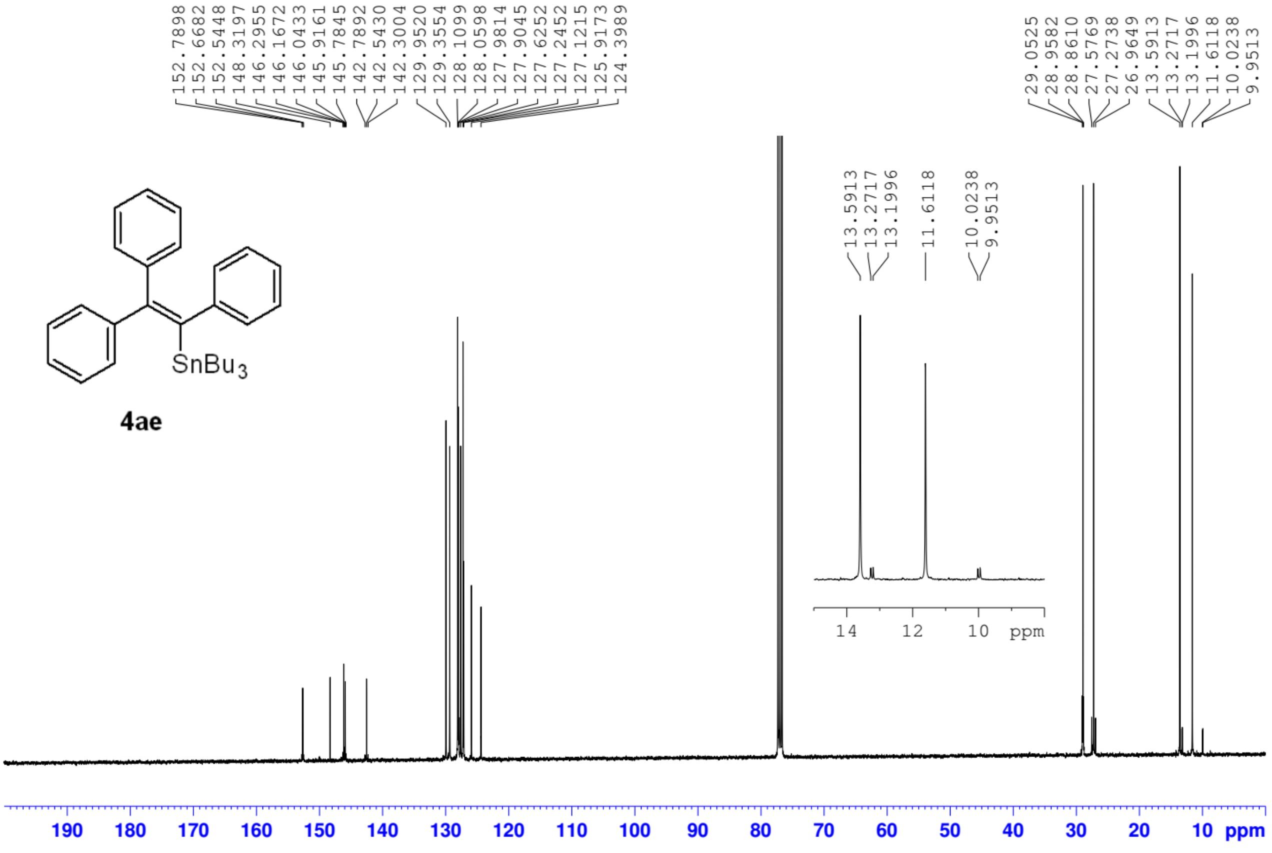
152.7898
152.6682
152.5448
148.3197
146.2955
146.1672
146.0433
145.9161
145.7845
142.7892
142.5430
142.3004
129.9520
129.3554
128.1099
128.0598
127.9814
127.9045
127.6252
127.2452
127.1215
125.9173
124.3989
29.0525
28.9582
28.8610
27.5769
27.2738
26.9649
13.5913
13.2717
13.1996
11.6118
10.0238
9.9513
SnBu3
4ae
14
12
10
ppm
190
180
170
160
150
140
130
120
110
100
90
80
70
60
50
40
30
20
10
ppm

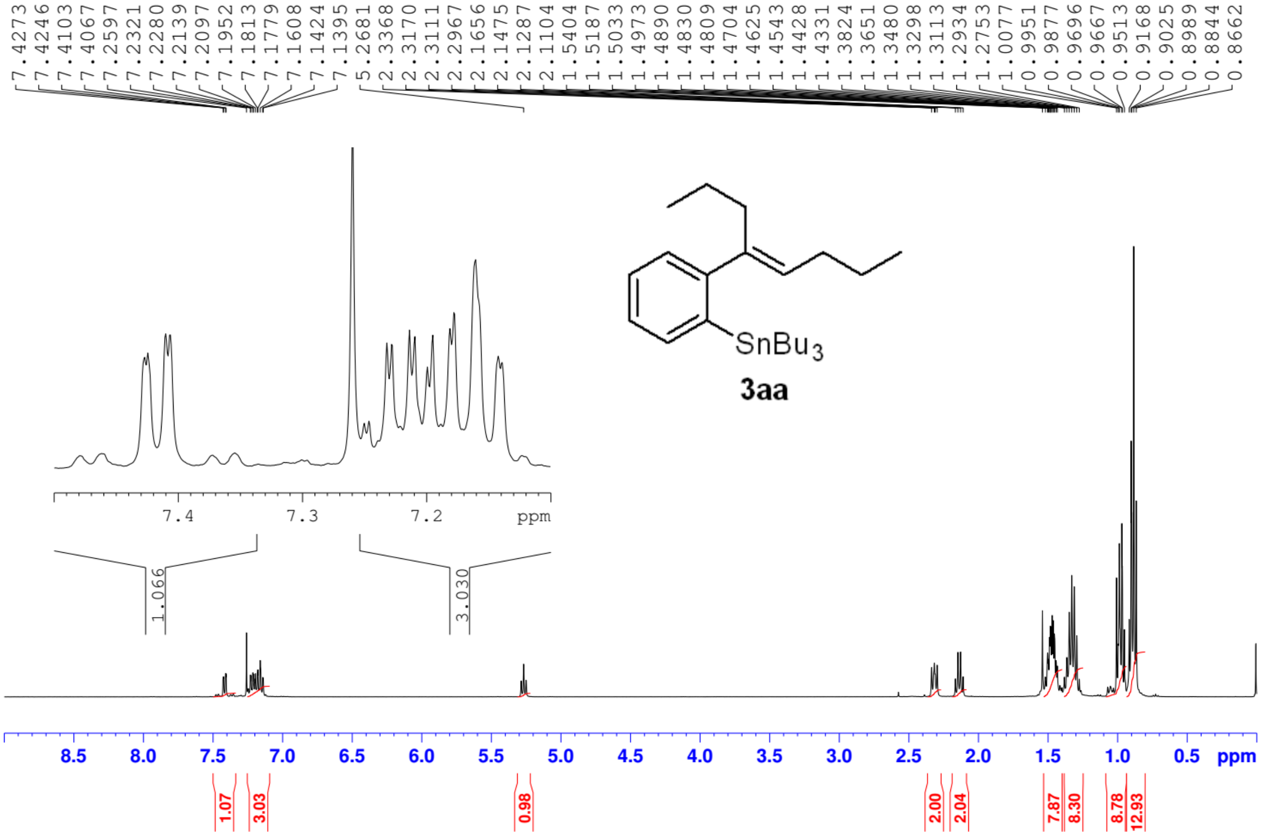
7.4273
7.4246
7.4103
7.4067
7.2597
7.2321
7.2280
7.2139
7.2097
7.1952
7.1813
7.1779
7.1608
7.1424
7.1395
5.2681
2.3368
2.3170
2.3111
2.2967
2.1656
2.1475
2.1287
2.1104
1.5404
1.5187
1.5033
1.4973
1.4890
1.4830
1.4809
1.4704
1.4625
1.4543
1.4428
1.4331
1.3824
1.3651
1.3480
1.3298
1.3113
1.2934
1.2753
1.0077
0.9951
0.9877
0.9696
0.9667
0.9513
0.9168
0.9025
0.8989
0.8844
0.8662
SnBu3
3aa
7.4
7.3
7.2
ppm
1.066
3.030
8.5
8.0
7.5
7.0
6.5
6.0
5.5
5.0
4.5
4.0
3.5
3.0
2.5
2.0
1.5
1.0
0.5
ppm
1.07
3.03
0.98
2.00
2.04
7.87
8.30
8.78
12.93

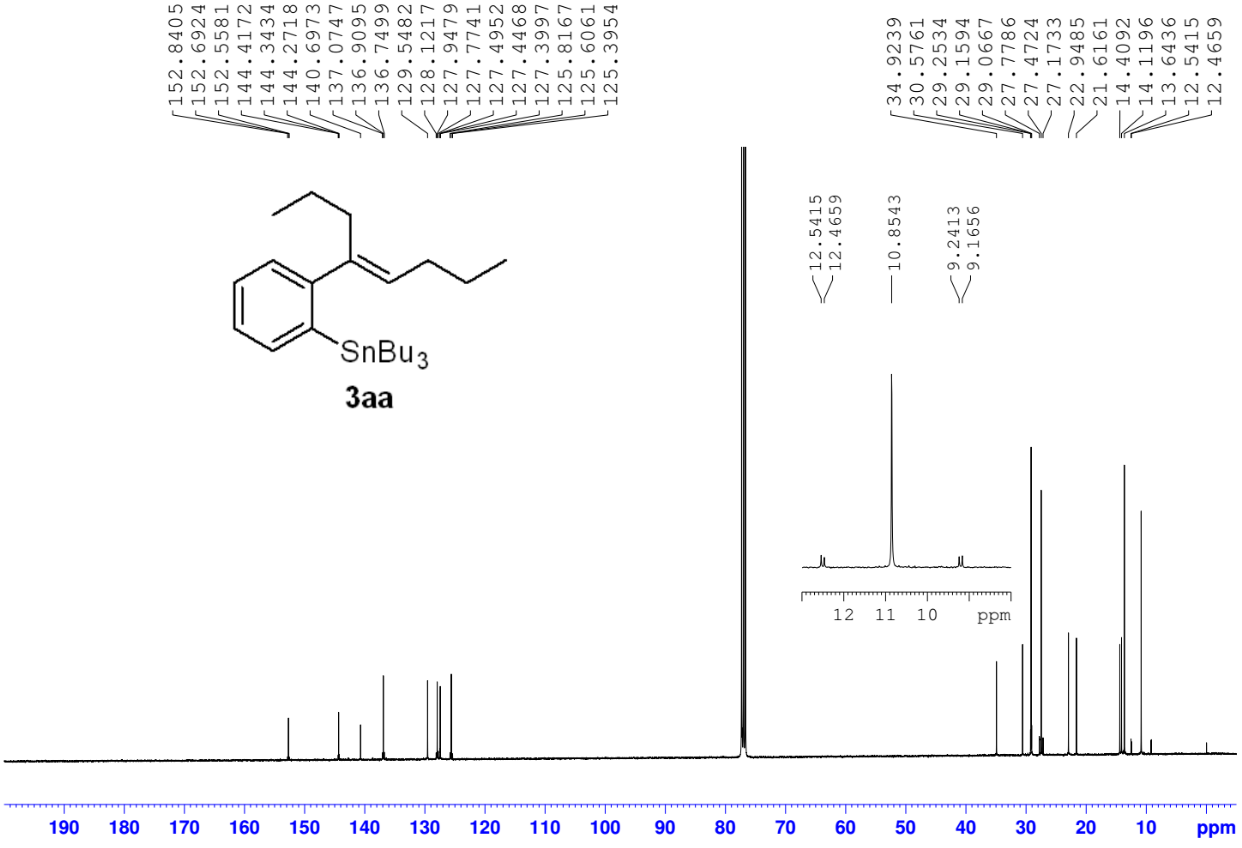
152.8405
152.6924
152.5581
144.4172
144.3434
144.2718
140.6973
137.0747
136.9095
136.7499
129.5482
128.1217
127.9479
127.7741
127.4952
127.4468
127.3997
125.8167
125.6061
125.3954
34.9239
30.5761
29.2534
29.1594
29.0667
27.7786
27.4724
27.1733
22.9485
21.6161
14.4092
14.1196
13.6436
12.5415
12.4659
12.5415
12.4659
10.8543
9.2413
9.1656
SnBu3
3aa
12
11
10
ppm
190
180
170
160
150
140
130
120
110
100
90
80
70
60
50
40
30
20
10
ppm

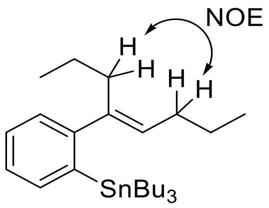

3aa

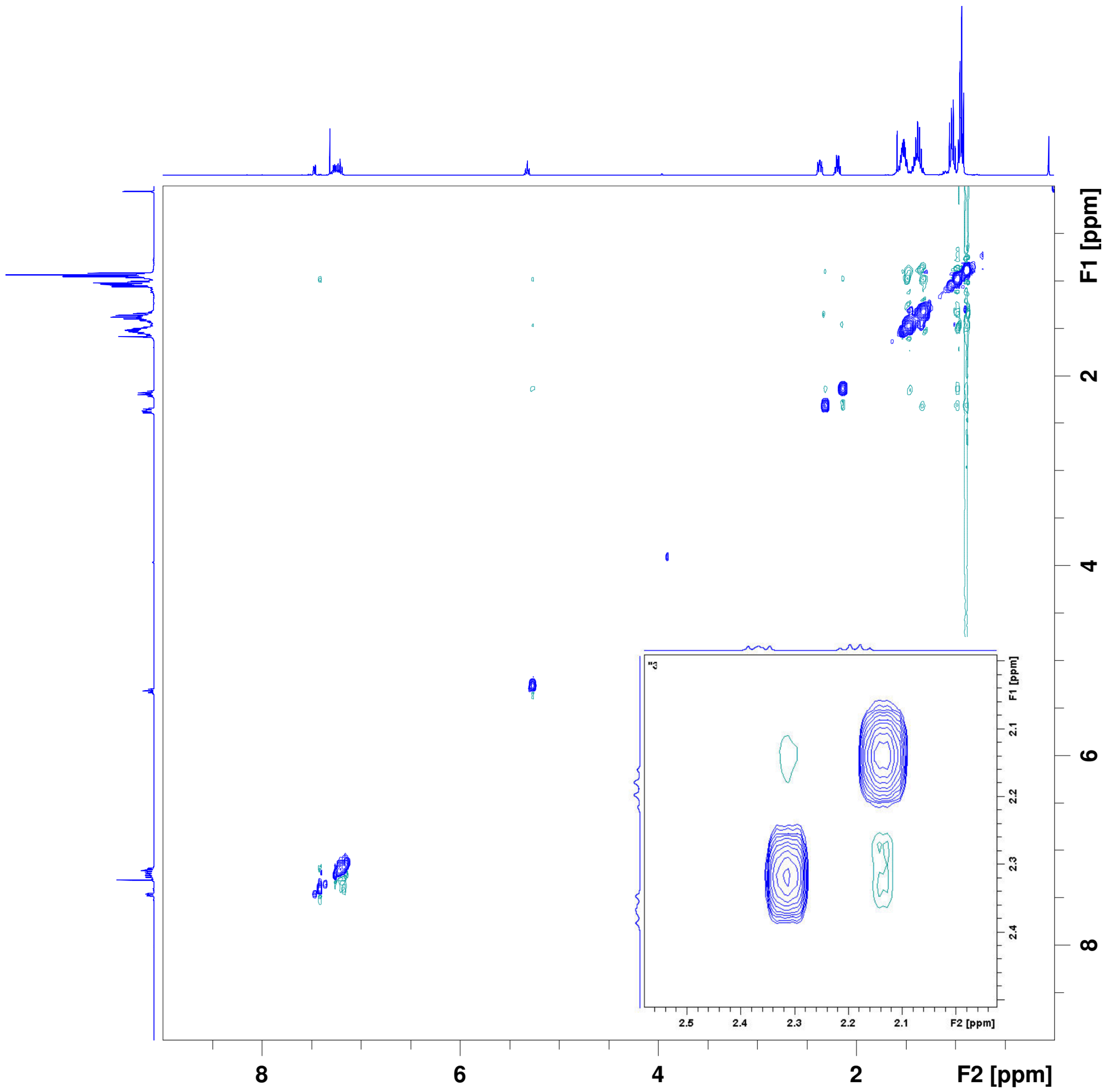

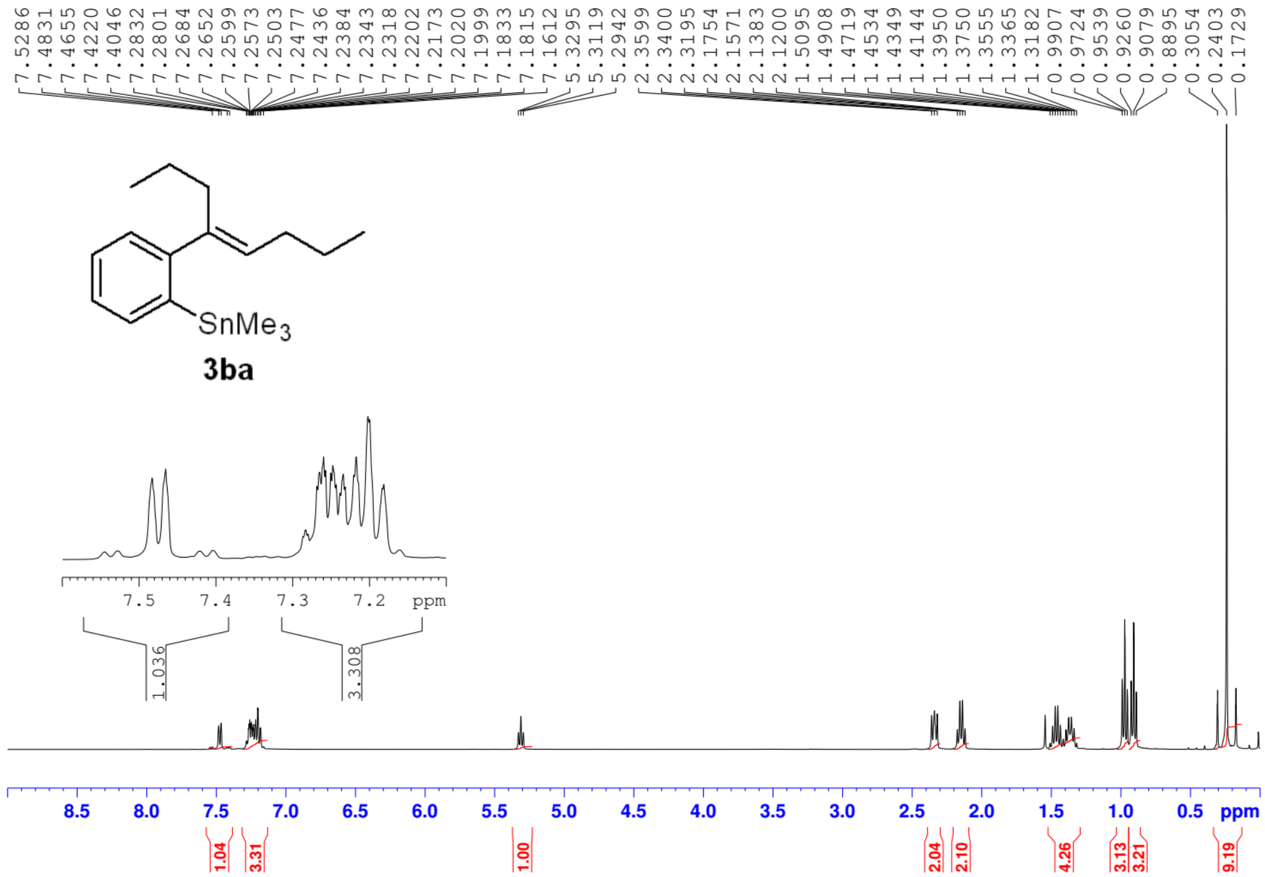
7.5286
7.4831
7.4655
7.4220
7.4046
7.2832
7.2801
7.2684
7.2652
7.2599
7.2573
7.2503
7.2477
7.2436
7.2384
7.2343
7.2318
7.2202
7.2173
7.2020
7.1999
7.1833
7.1815
7.1612
5.3295
5.3119
5.2942
2.3599
2.3400
2.3195
2.1754
2.1571
2.1383
2.1200
1.5095
1.4908
1.4719
1.4534
1.4349
1.4144
1.3950
1.3750
1.3555
1.3365
1.3182
0.9907
0.9724
0.9539
0.9260
0.9079
0.8895
0.3054
0.2403
0.1729
SnMe3
3ba
7.5 7.4 7.3 7.2 ppm
1.036
3.308
8.5 8.0 7.5 7.0 6.5 6.0 5.5 5.0 4.5 4.0 3.5 3.0 2.5 2.0 1.5 1.0 0.5 ppm
1.04
3.31
1.00
2.04
2.10
4.26
3.13
3.21
9.19

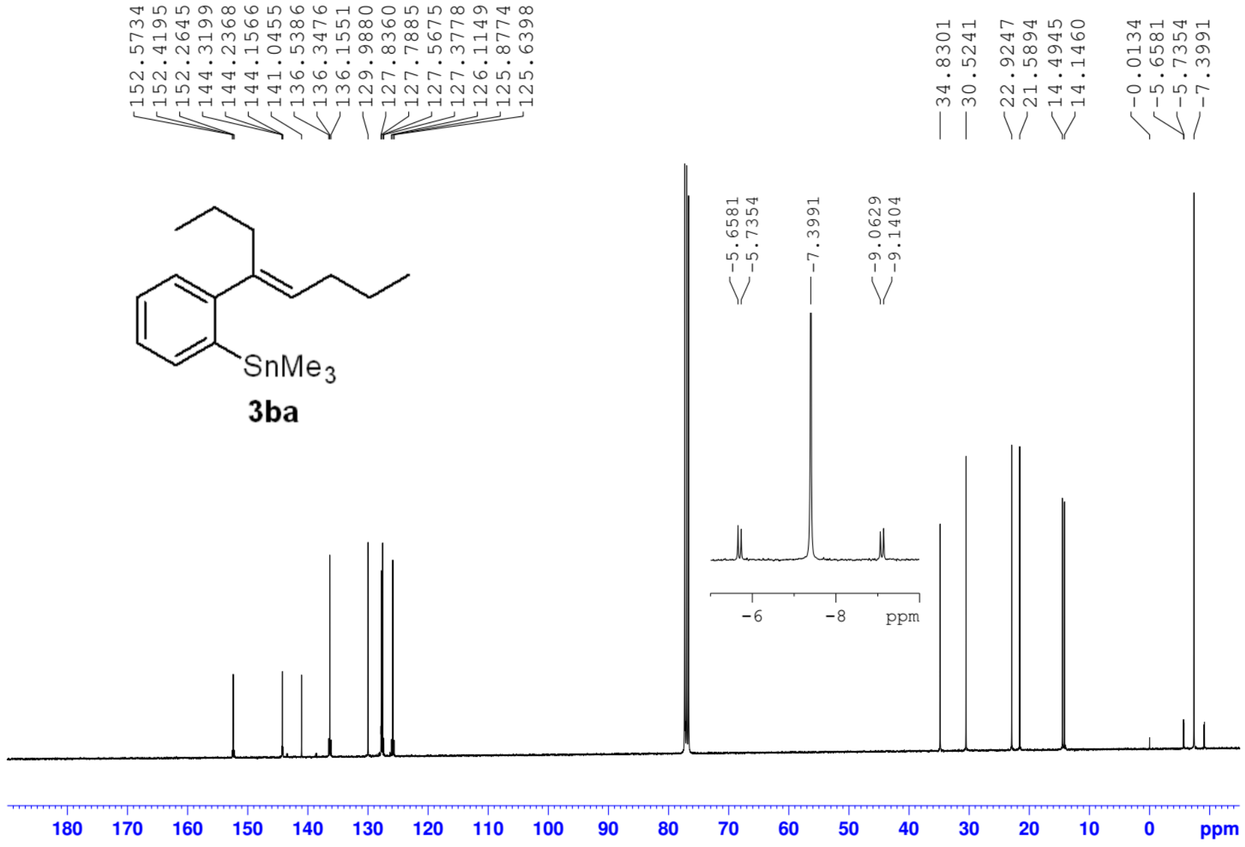
152.5734
152.4195
152.2645
144.3199
144.2368
144.1566
141.0455
136.5386
136.3476
136.1551
129.9880
127.8360
127.7885
127.5675
127.3778
126.1149
125.8774
125.6398
34.8301
30.5241
22.9247
21.5894
14.4945
14.1460
-0.0134
-5.6581
-5.7354
-7.3991
SnMe3
3ba
-5.6581
-5.7354
-7.3991
-9.0629
-9.1404
-6 -8 ppm
180 170 160 150 140 130 120 110 100 90 80 70 60 50 40 30 20 10 0 ppm

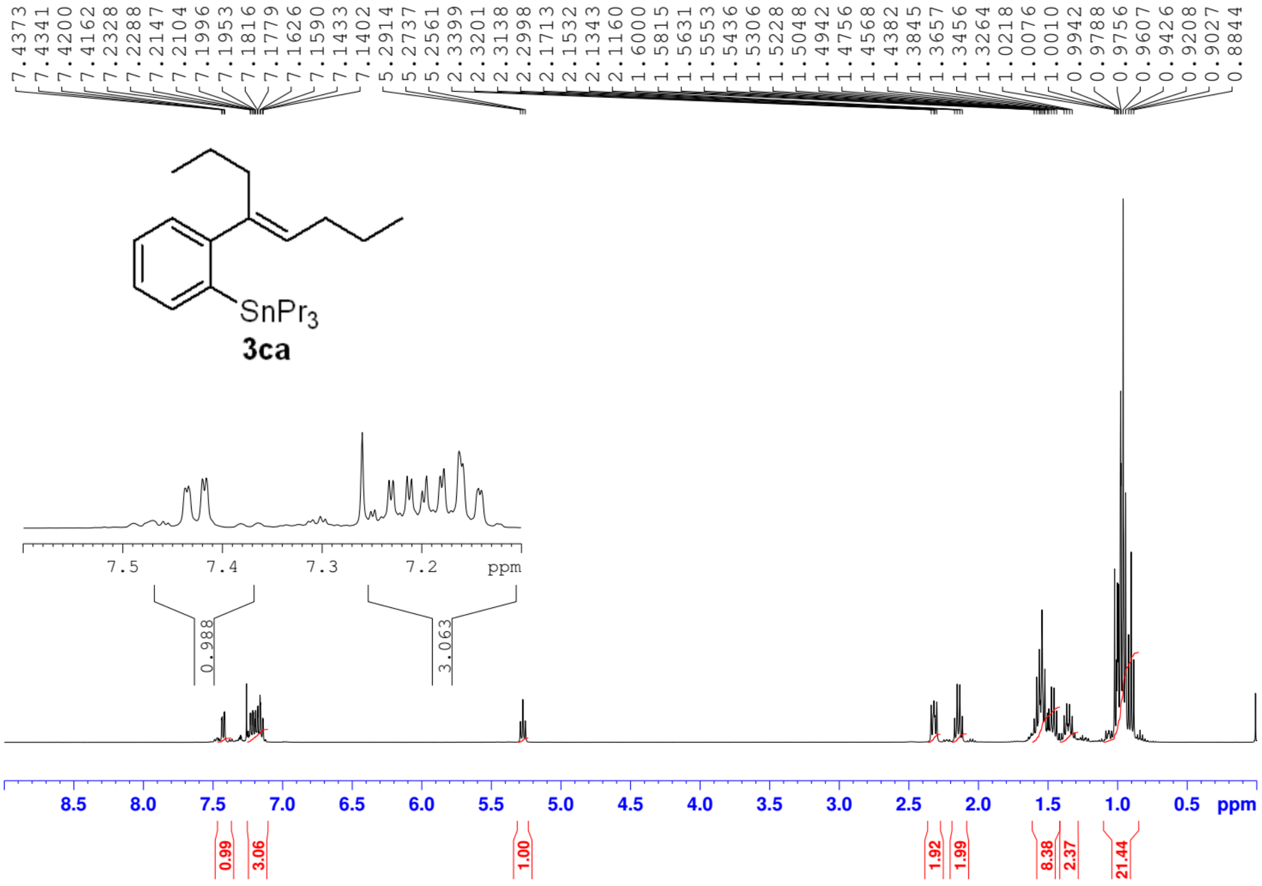
7.4373
7.4341
7.4200
7.4162
7.2328
7.2288
7.2147
7.2104
7.1996
7.1953
7.1816
7.1779
7.1626
7.1590
7.1433
7.1402
5.2914
5.2737
5.2561
2.3399
2.3201
2.3138
2.2998
2.1713
2.1532
2.1343
2.1160
1.6000
1.5815
1.5631
1.5553
1.5436
1.5306
1.5228
1.5048
1.4942
1.4756
1.4568
1.4382
1.3845
1.3657
1.3456
1.3264
1.0218
1.0076
1.0010
0.9942
0.9788
0.9756
0.9607
0.9426
0.9208
0.9027
0.8844
SnPr3
3ca
0.988
3.063
0.99
3.06
1.00
1.92
1.99
8.38
2.37
21.44
ppm

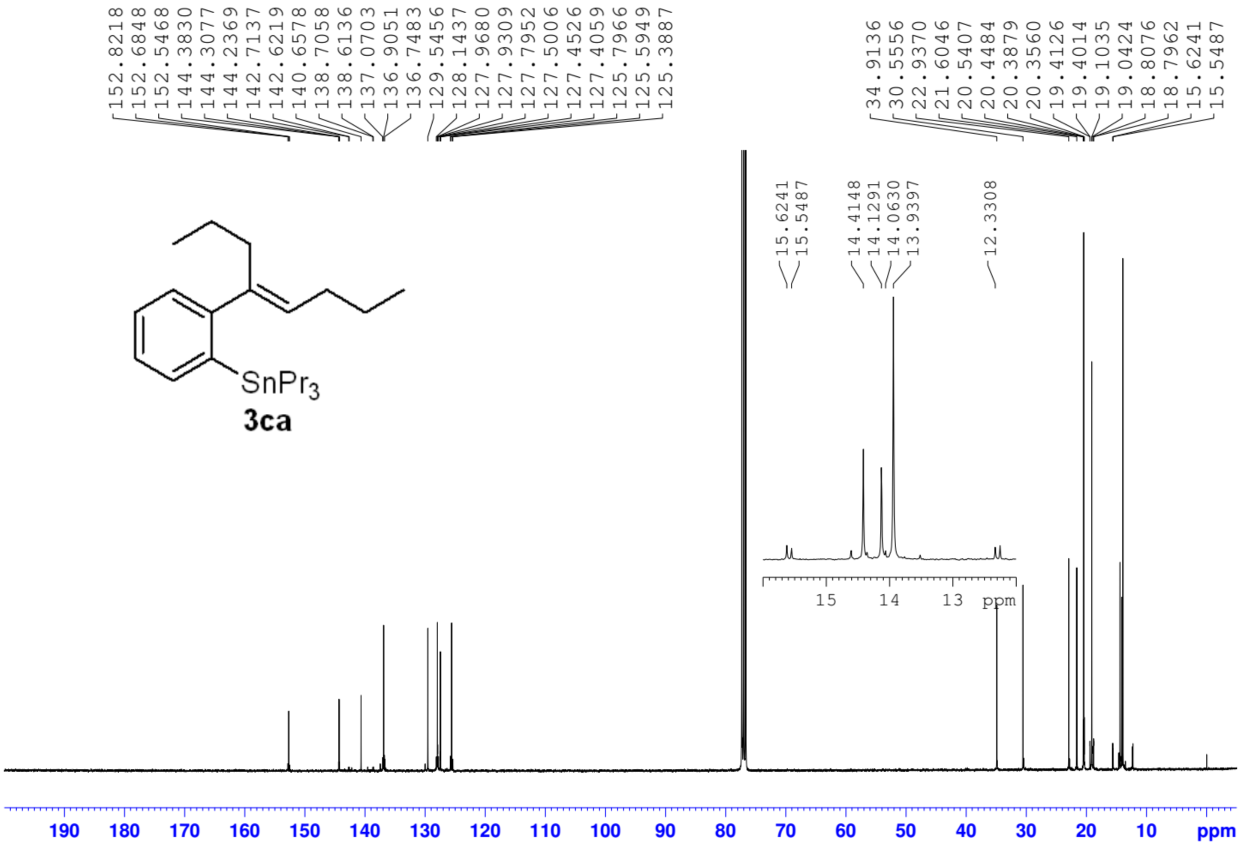
152.8218
152.6848
152.5468
144.3830
144.3077
144.2369
142.7137
142.6219
140.6578
138.7058
138.6136
137.0703
136.9051
136.7483
129.5456
128.1437
127.9680
127.9309
127.7952
127.5006
127.4526
127.4059
125.7966
125.5949
125.3887
34.9136
30.5556
22.9370
21.6046
20.5407
20.4484
20.3879
20.3560
19.4126
19.4014
19.1035
19.0424
18.8076
18.7962
15.6241
15.5487
14.4148
14.1291
14.0630
13.9397
12.3308
SnPr3
3ca
ppm

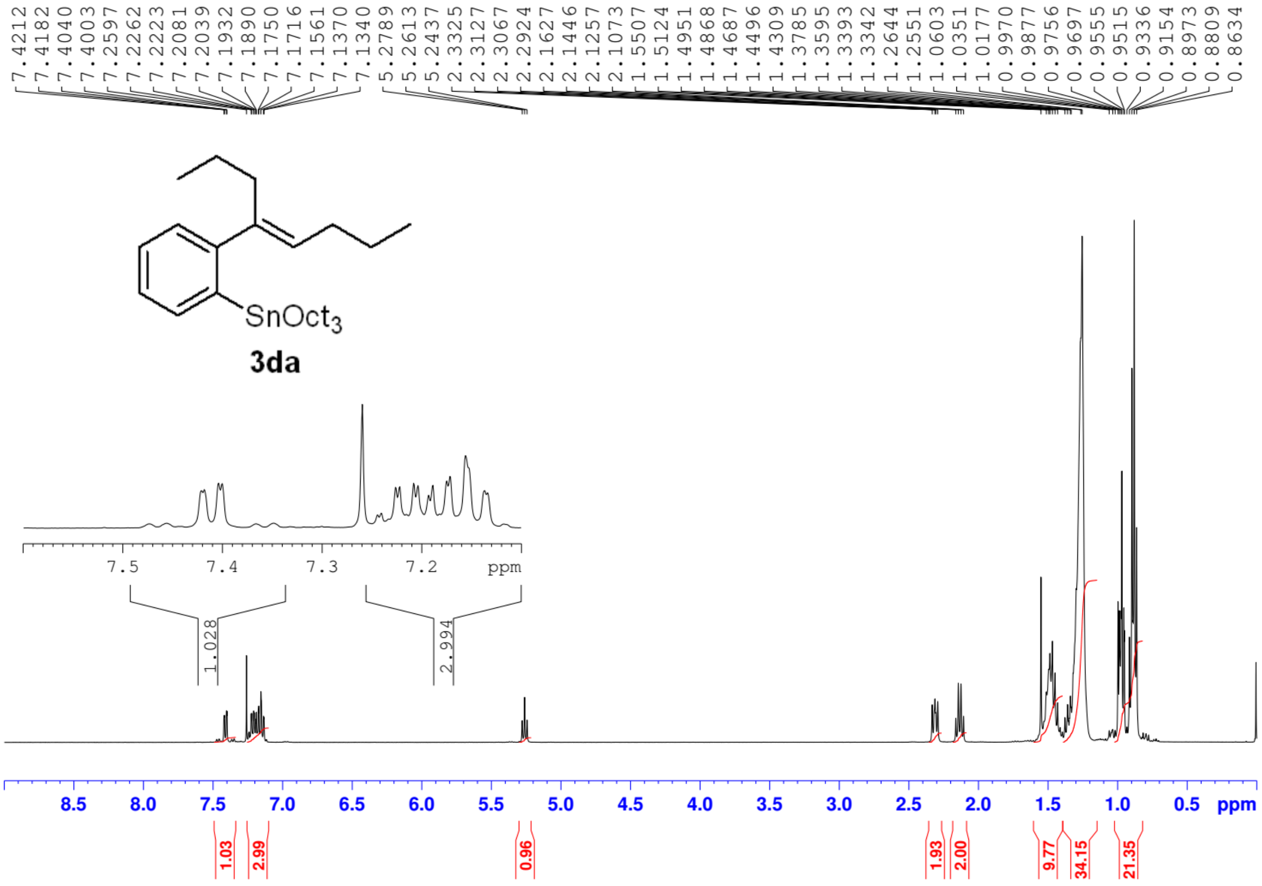

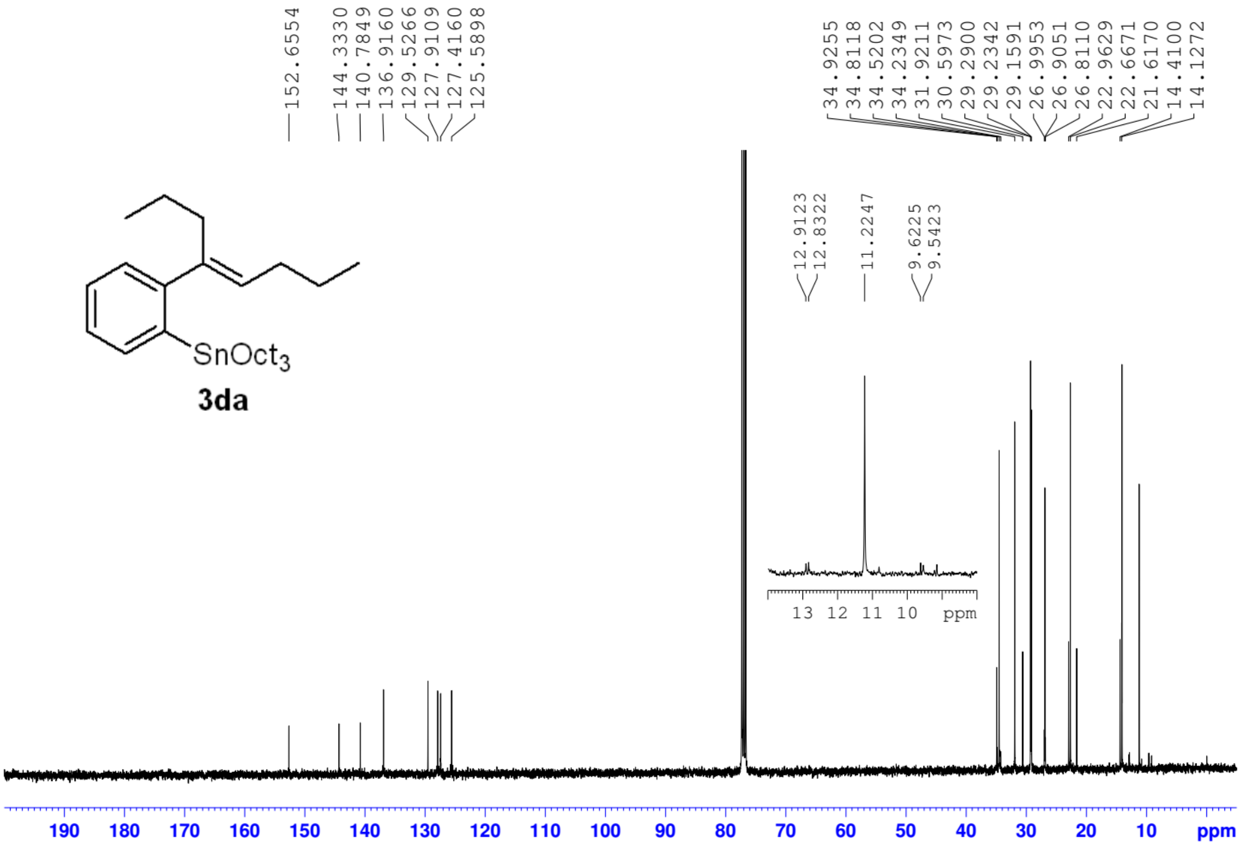

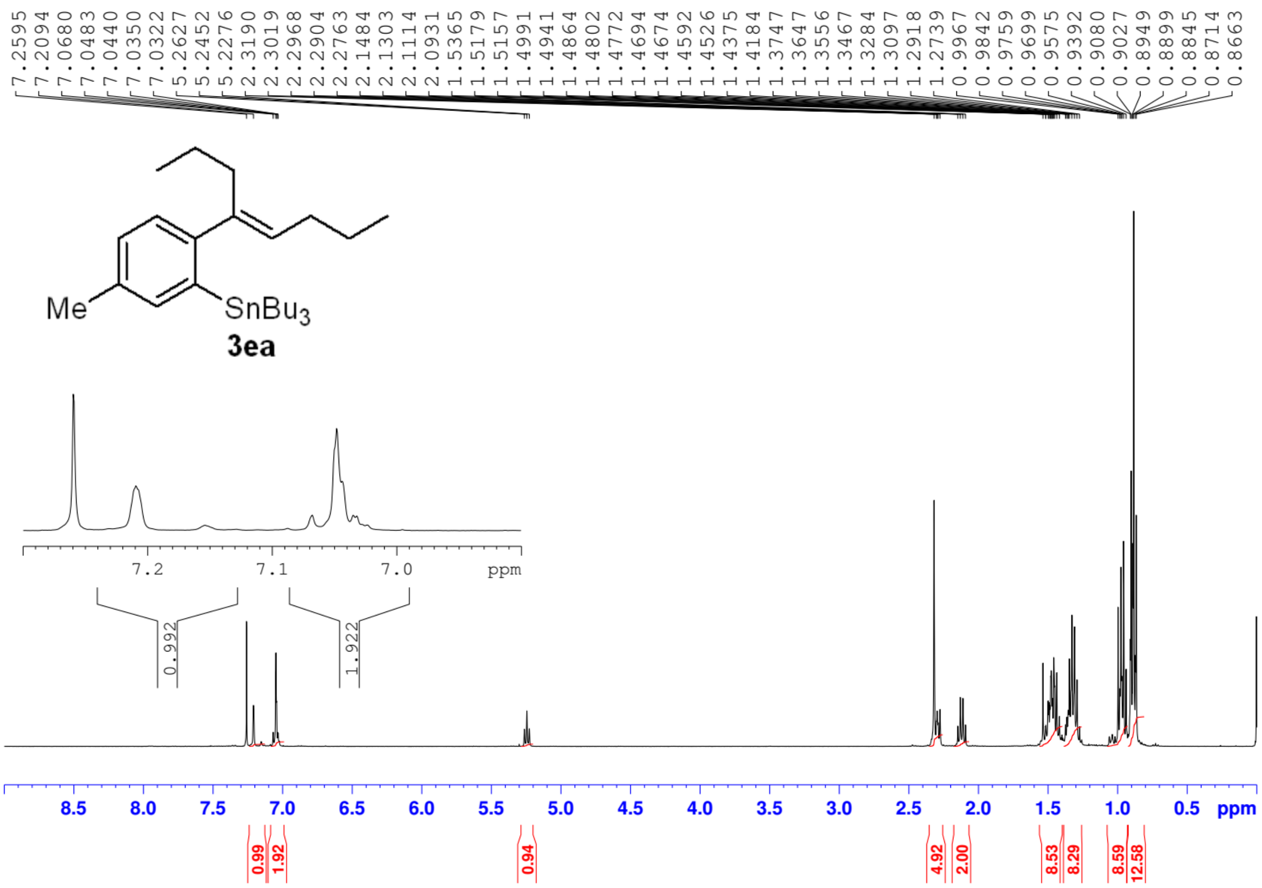

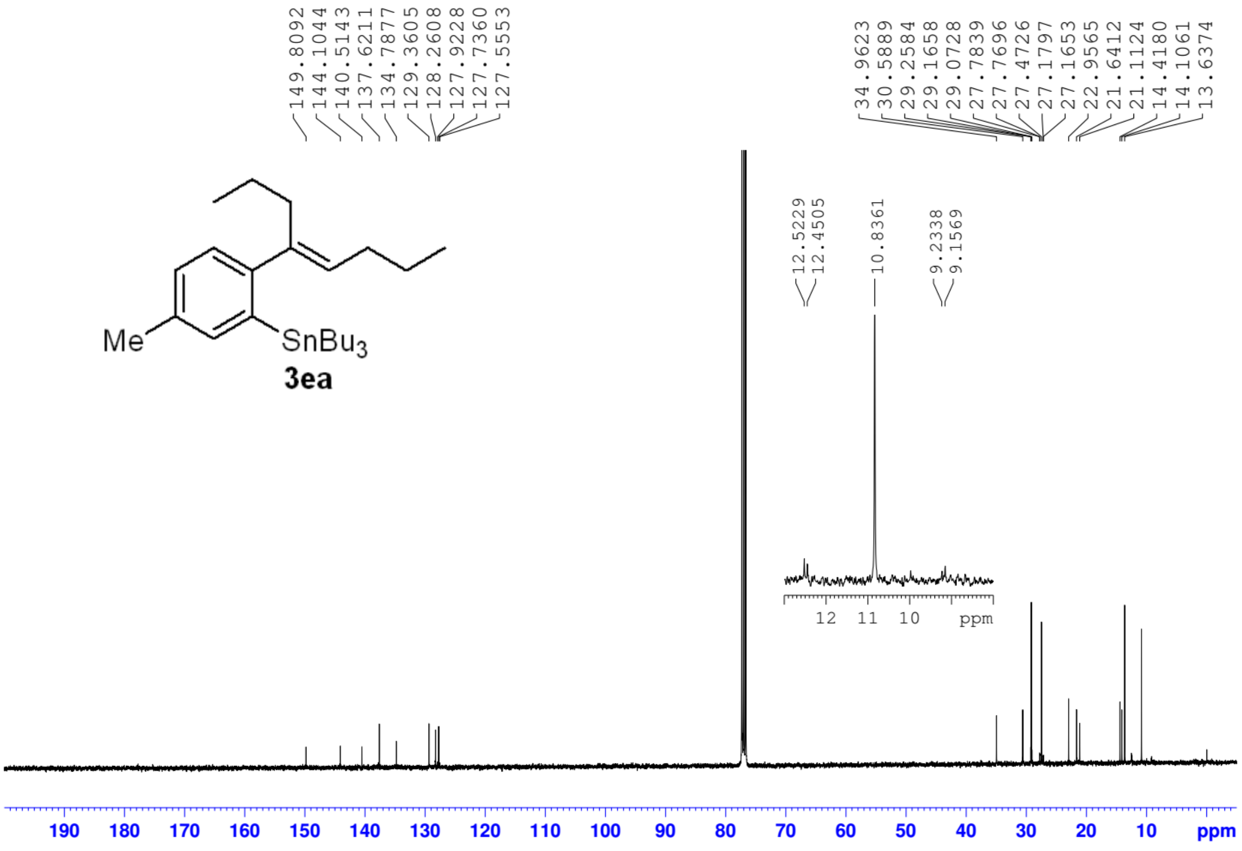

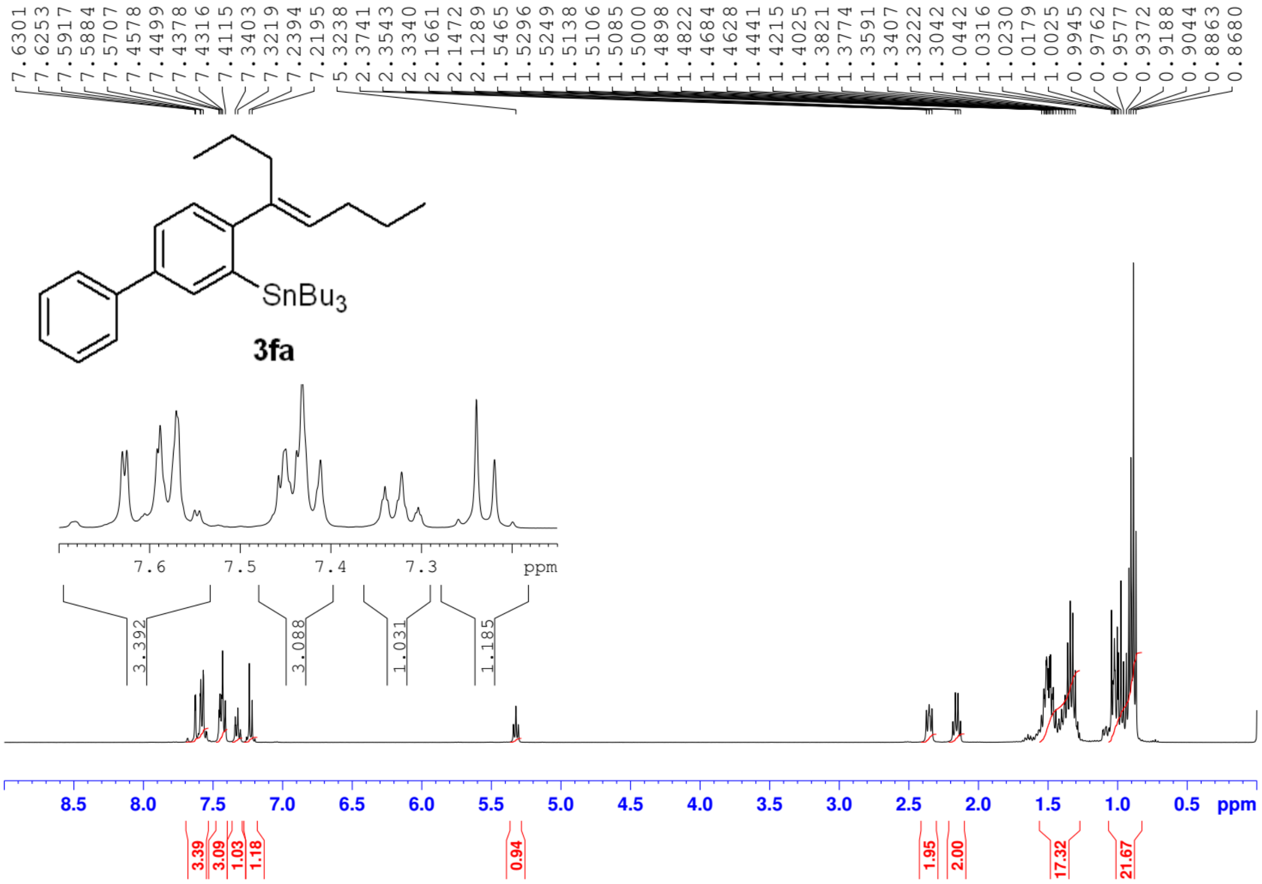

7.6301
7.6253
7.5917
7.5884
7.5707
7.4578
7.4499
7.4378
7.4316
7.4115
7.3403
7.3219
7.2394
7.2195
5.3238
2.3741
2.3543
2.3340
2.1661
2.1472
2.1289
1.5465
1.5296
1.5249
1.5138
1.5106
1.5085
1.5000
1.4898
1.4822
1.4684
1.4628
1.4441
1.4215
1.4025
1.3821
1.3774
1.3591
1.3407
1.3222
1.3042
1.0442
1.0316
1.0230
1.0179
1.0025
0.9945
0.9762
0.9577
0.9372
0.9188
0.9044
0.8863
0.8680
SnBu3
3fa
7.6
7.5
7.4
7.3
ppm
3.392
3.088
1.031
1.185
8.5 8.0 7.5 7.0 6.5 6.0 5.5 5.0 4.5 4.0 3.5 3.0 2.5 2.0 1.5 1.0 0.5 ppm
3.39
3.09
1.03
1.18
0.94
1.95
2.00
17.32
21.67

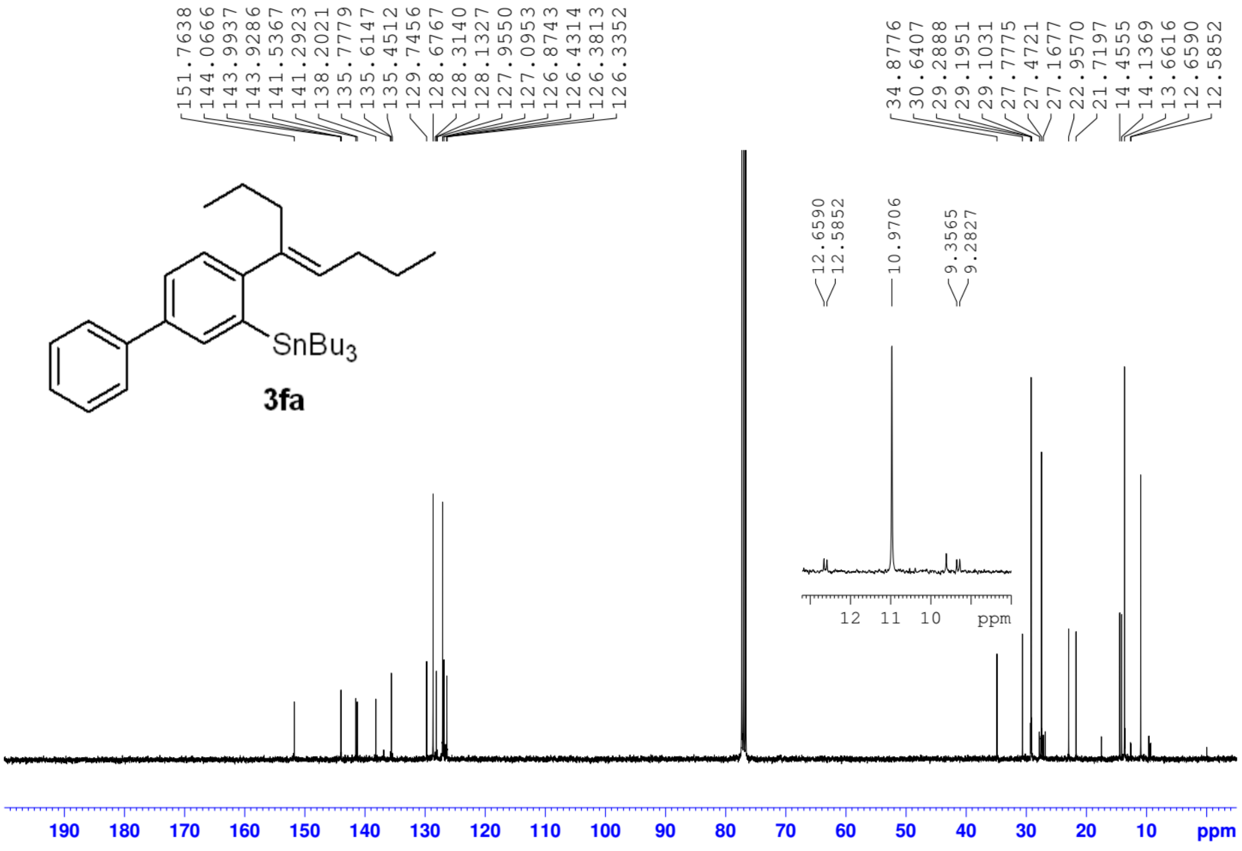

151.7638
144.0666
143.9937
143.9286
141.5367
141.2923
138.2021
135.7779
135.6147
135.4512
129.7456
128.6767
128.3140
128.1327
127.9550
127.0953
126.8743
126.4314
126.3813
126.3352
34.8776
30.6407
29.2888
29.1951
29.1031
27.7775
27.4721
27.1677
22.9570
21.7197
14.4555
14.1369
13.6616
12.6590
12.5852
12.6590
12.5852
10.9706
9.3565
9.2827
SnBu3
3fa
12 11 10 ppm
190 180 170 160 150 140 130 120 110 100 90 80 70 60 50 40 30 20 10 ppm

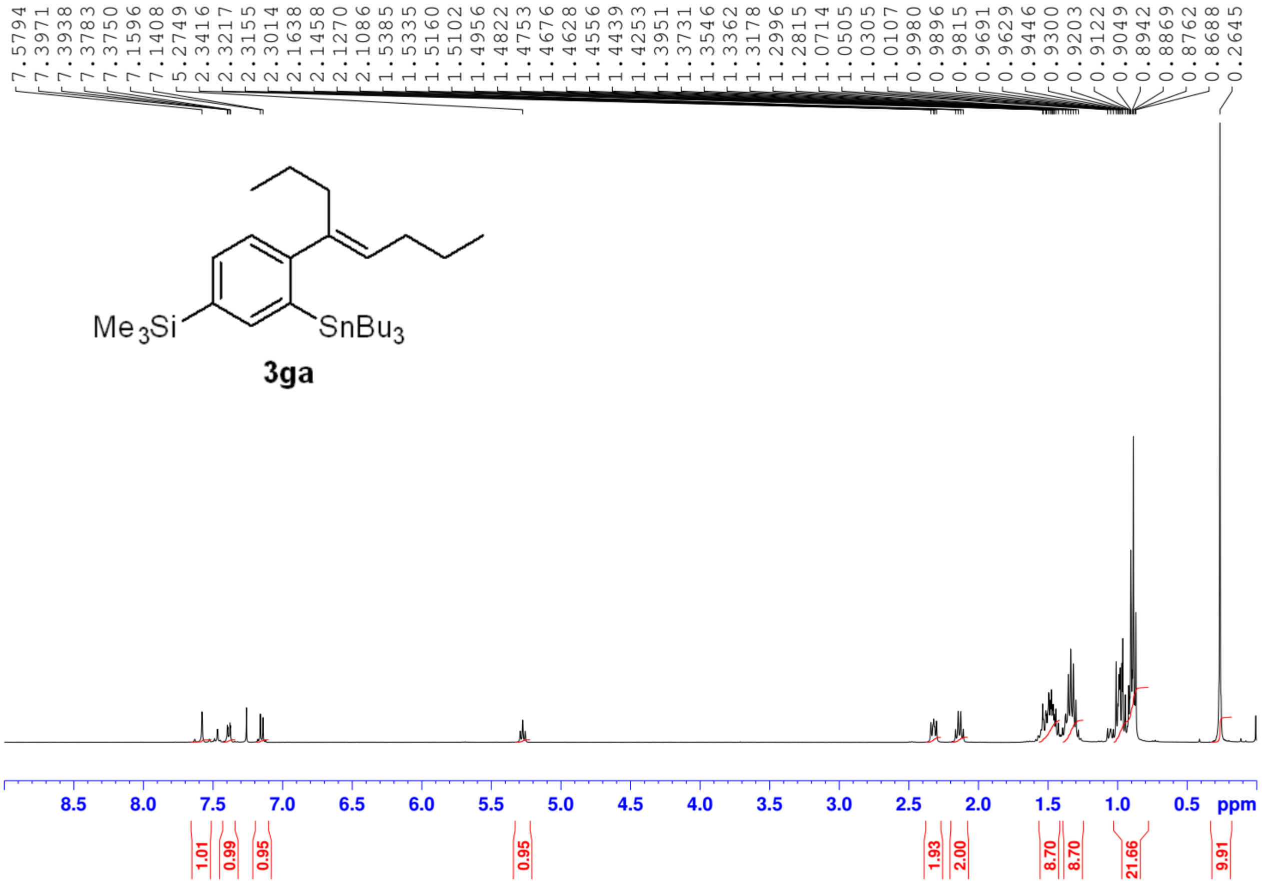
7.5794
7.3971
7.3938
7.3783
7.3750
7.1596
7.1408
5.2749
2.3416
2.3217
2.3155
2.3014
2.1638
2.1458
2.1270
2.1086
1.5385
1.5335
1.5160
1.5102
1.4956
1.4822
1.4753
1.4676
1.4628
1.4556
1.4439
1.4253
1.3951
1.3731
1.3546
1.3362
1.3178
1.2996
1.2815
1.0714
1.0505
1.0305
1.0107
0.9980
0.9896
0.9815
0.9691
0.9629
0.9446
0.9300
0.9203
0.9122
0.9049
0.8942
0.8869
0.8762
0.8688
0.2645
Me3Si
SnBu3
3ga
1.01
0.99
0.95
0.95
1.93
2.00
8.70
8.70
21.66
9.91
ppm

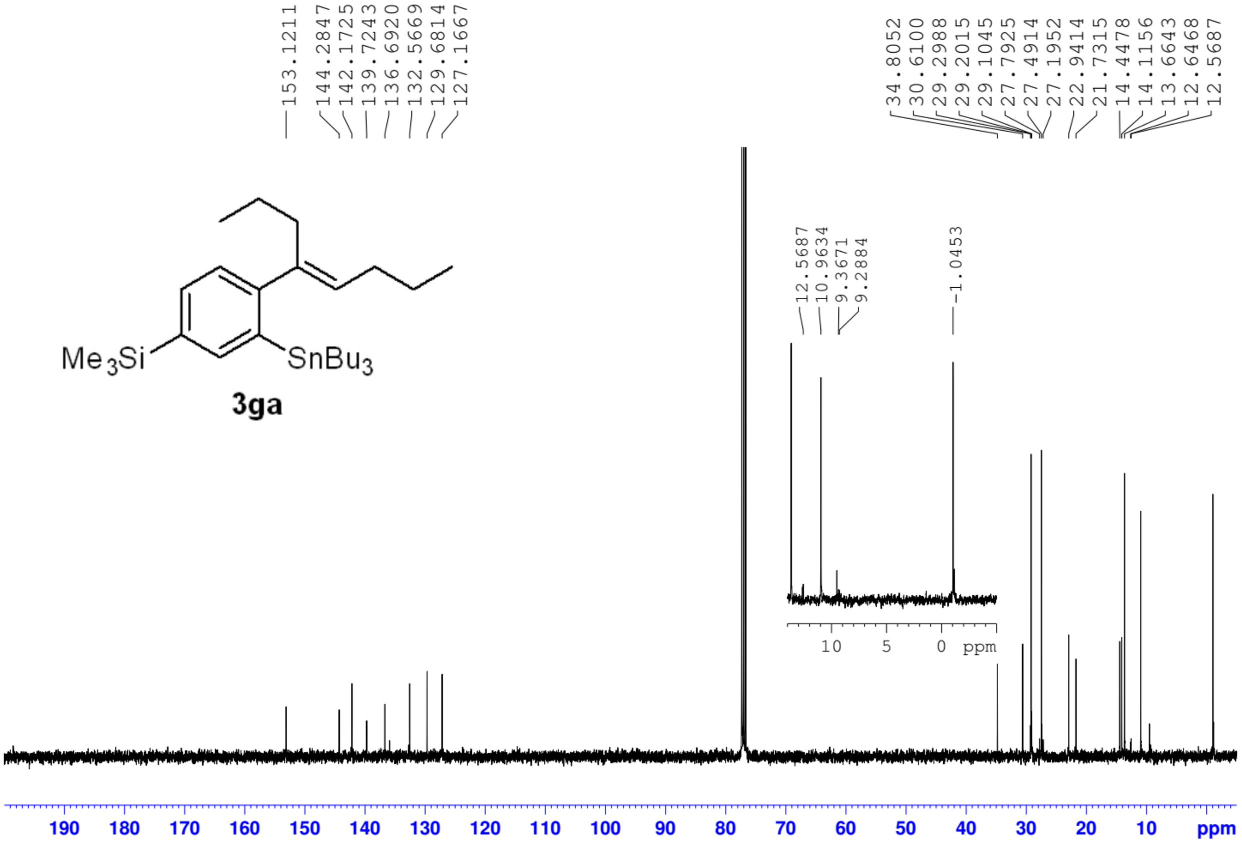
153.1211
144.2847
142.1725
139.7243
136.6920
132.5669
129.6814
127.1667
34.8052
30.6100
29.2988
29.2015
29.1045
27.7925
27.4914
27.1952
22.9414
21.7315
14.4478
14.1156
13.6643
12.6468
12.5687
Me3Si
SnBu3
3ga
12.5687
10.9634
9.3671
9.2884
-1.0453
ppm
ppm

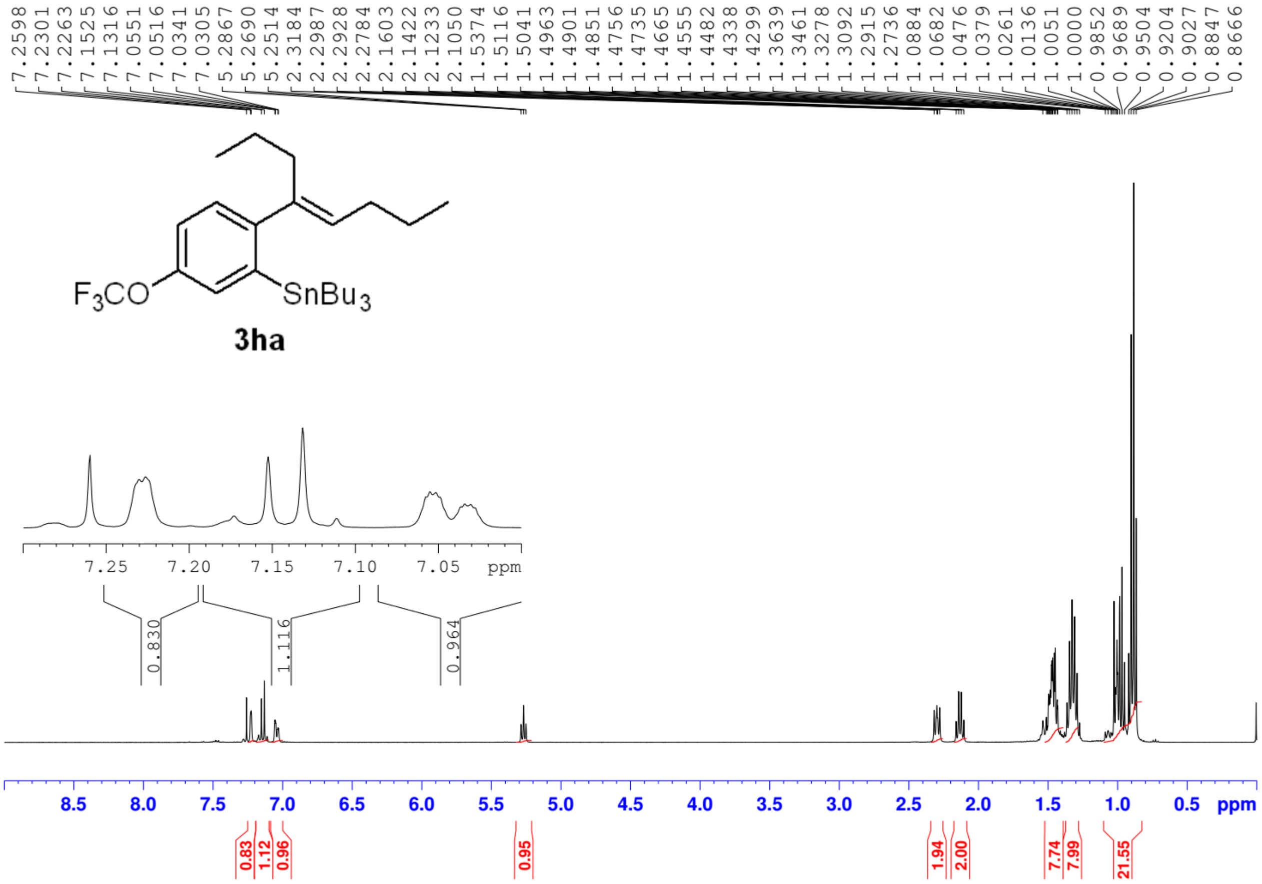

7.2598 7.2301 7.2263 7.1525 7.1316 7.0551 7.0516 7.0341 7.0305 5.2867 5.2690 5.2514 2.3184 2.2987 2.2928 2.2784 2.1603 2.1422 2.1233 2.1050 1.5374 1.5116 1.5041 1.4963 1.4901 1.4851 1.4756 1.4735 1.4665 1.4555 1.4482 1.4338 1.4299 1.3639 1.3461 1.3278 1.3092 1.2915 1.2736 1.0884 1.0682 1.0476 1.0379 1.0261 1.0136 1.0051 1.0000 0.9852 0.9689 0.9504 0.9204 0.9027 0.8847 0.8666
F3CO
SnBu3
3ha
7.25 7.20 7.15 7.10 7.05 ppm
0.830 1.116 0.964
8.5 8.0 7.5 7.0 6.5 6.0 5.5 5.0 4.5 4.0 3.5 3.0 2.5 2.0 1.5 1.0 0.5 ppm
0.83 1.12 0.96 0.95 1.94 2.00 7.74 7.99 21.55

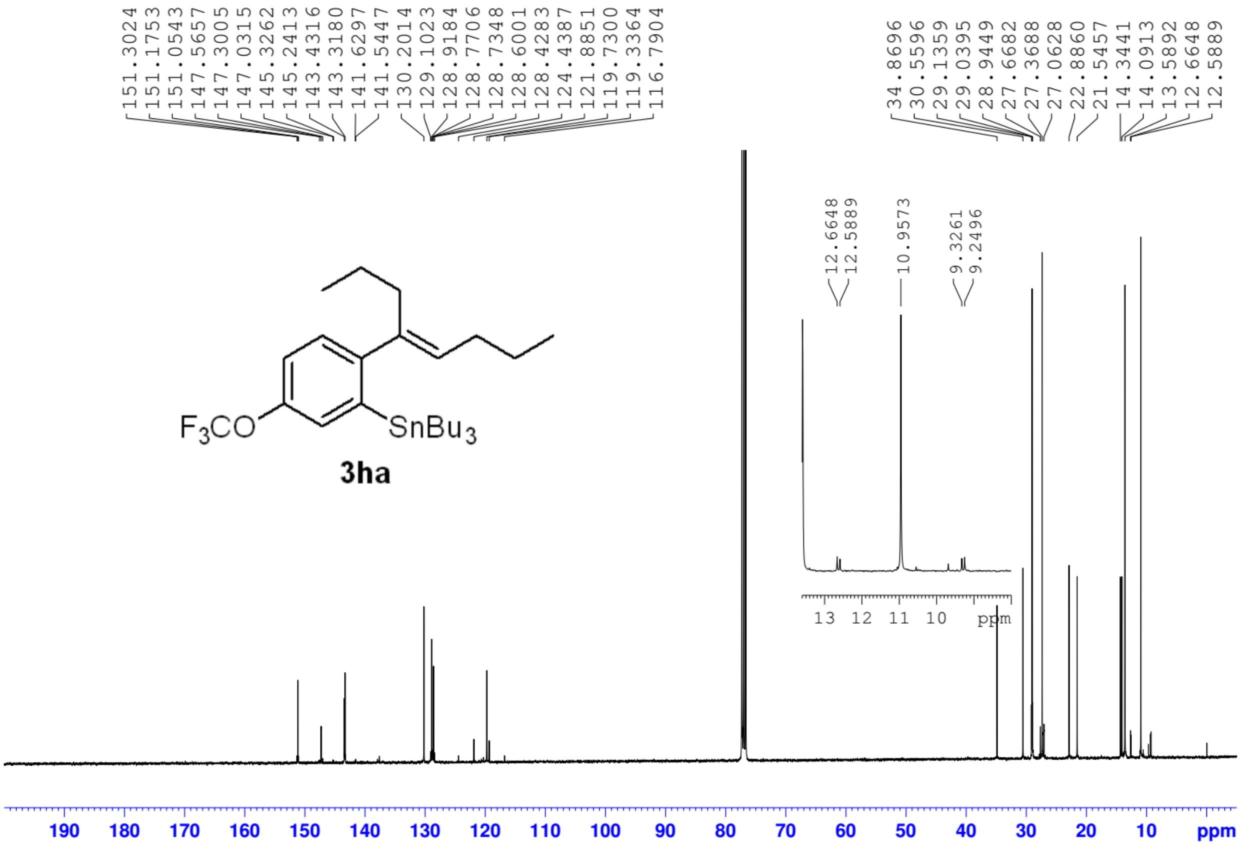

151.3024 151.1753 151.0543 147.5657 147.3005 147.0315 145.3262 145.2413 143.4316 143.3180 141.6297 141.5447 130.2014 129.1023 128.9184 128.7706 128.7348 128.6001 128.4283 124.4387 121.8851 119.7300 119.3364 116.7904
34.8696 30.5596 29.1359 29.0395 28.9449 27.6682 27.3688 27.0628 22.8860 21.5457 14.3441 14.0913 13.5892 12.6648 12.5889
12.6648 12.5889 10.9573 9.3261 9.2496
13 12 11 10 ppm
F3CO
SnBu3
3ha
190 180 170 160 150 140 130 120 110 100 90 80 70 60 50 40 30 20 10 ppm

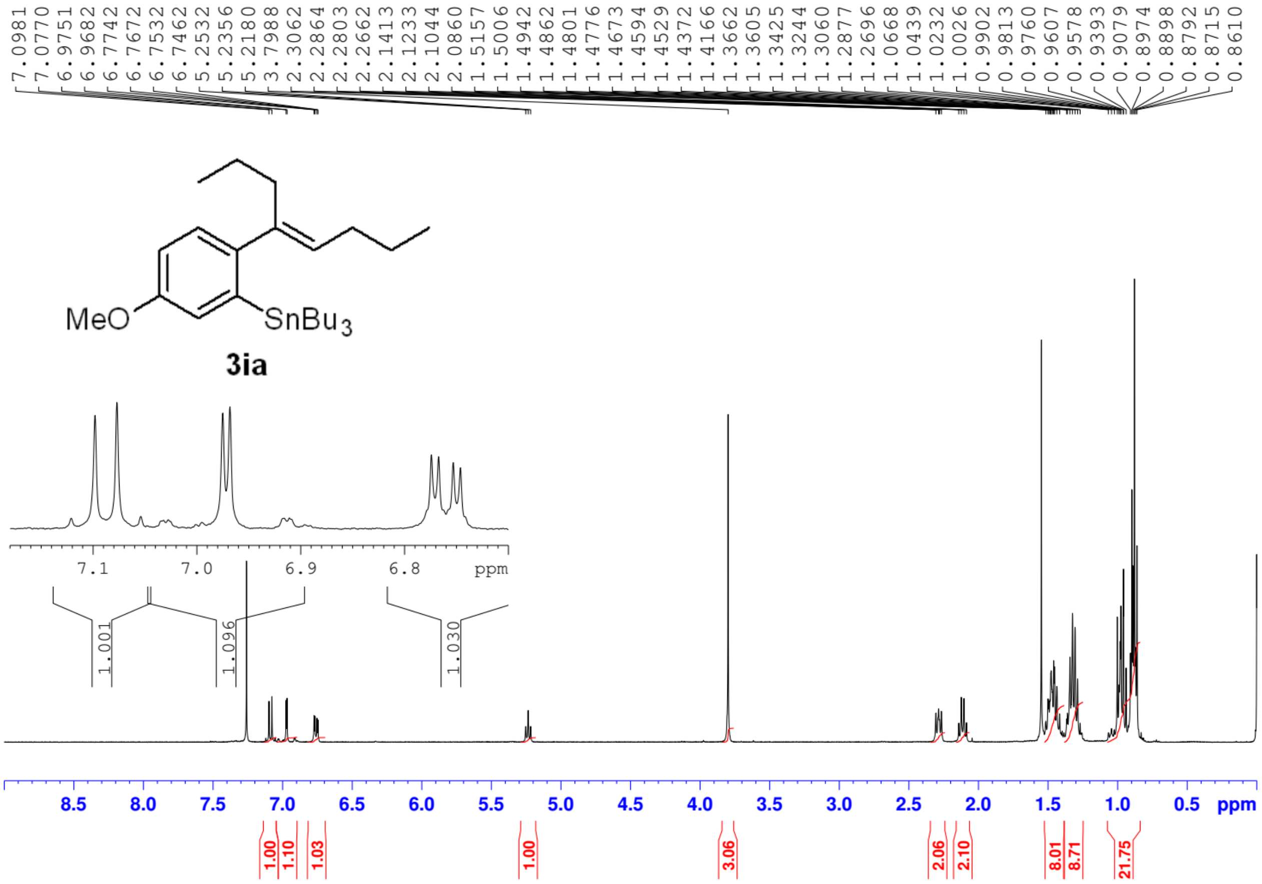
MeO
SnBu3
3ia
ppm

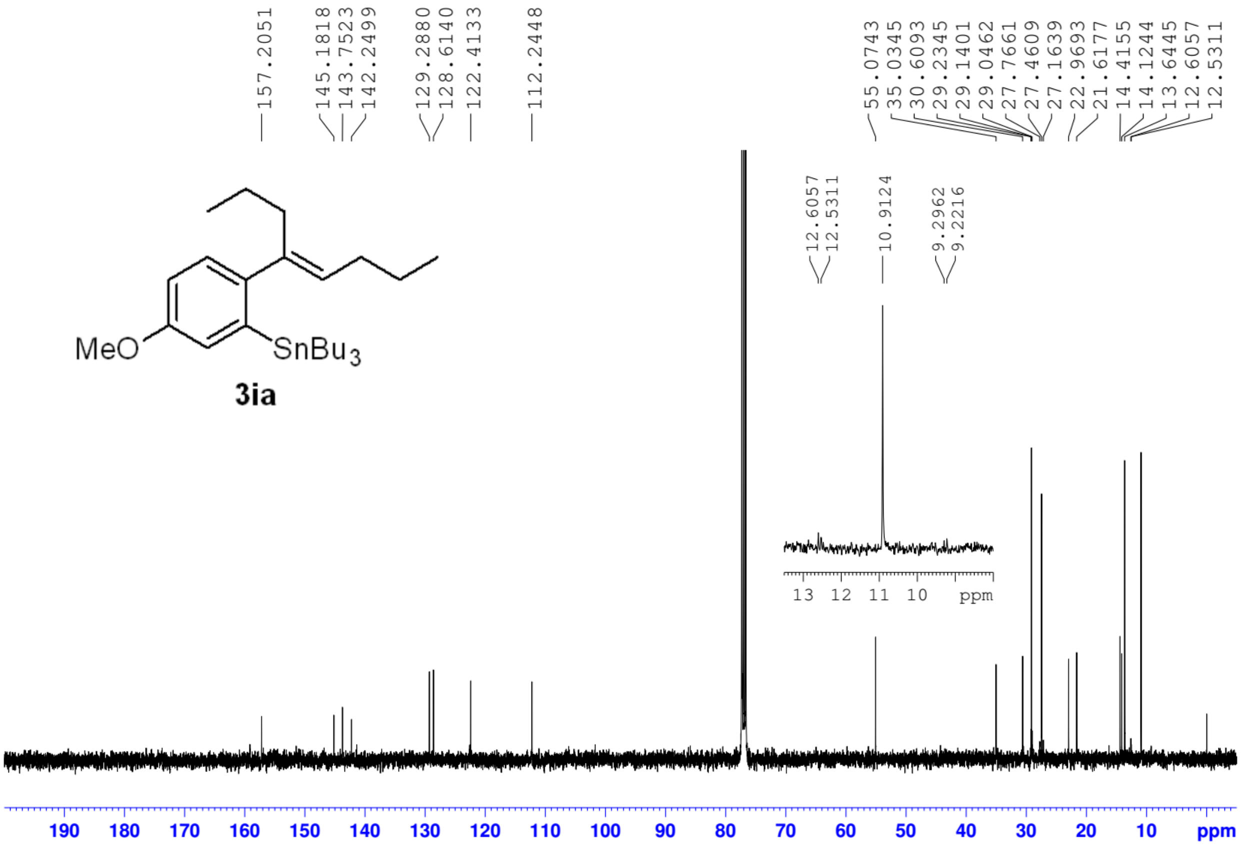
MeO
SnBu3
3ia
ppm

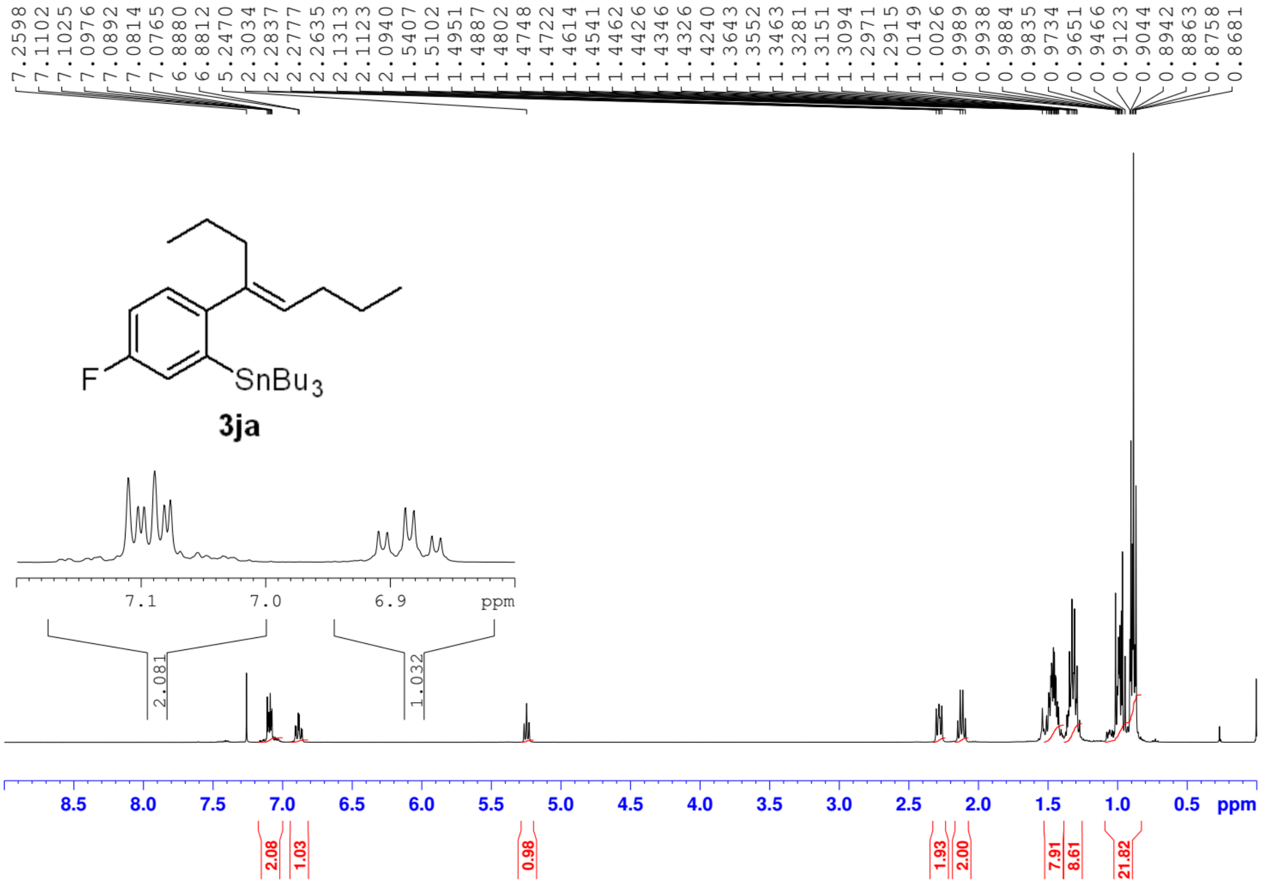

F
SnBu3
3ja
ppm

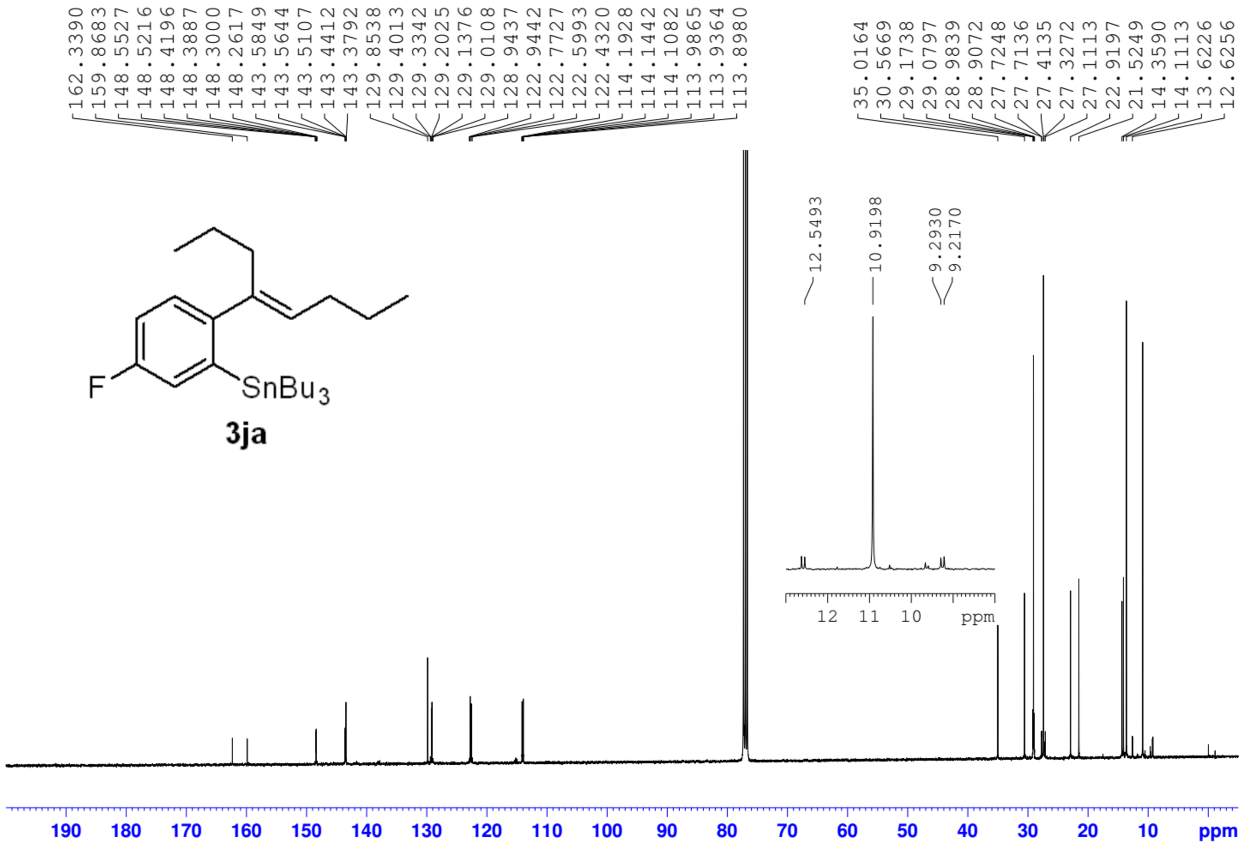

F
SnBu3
3ja
ppm

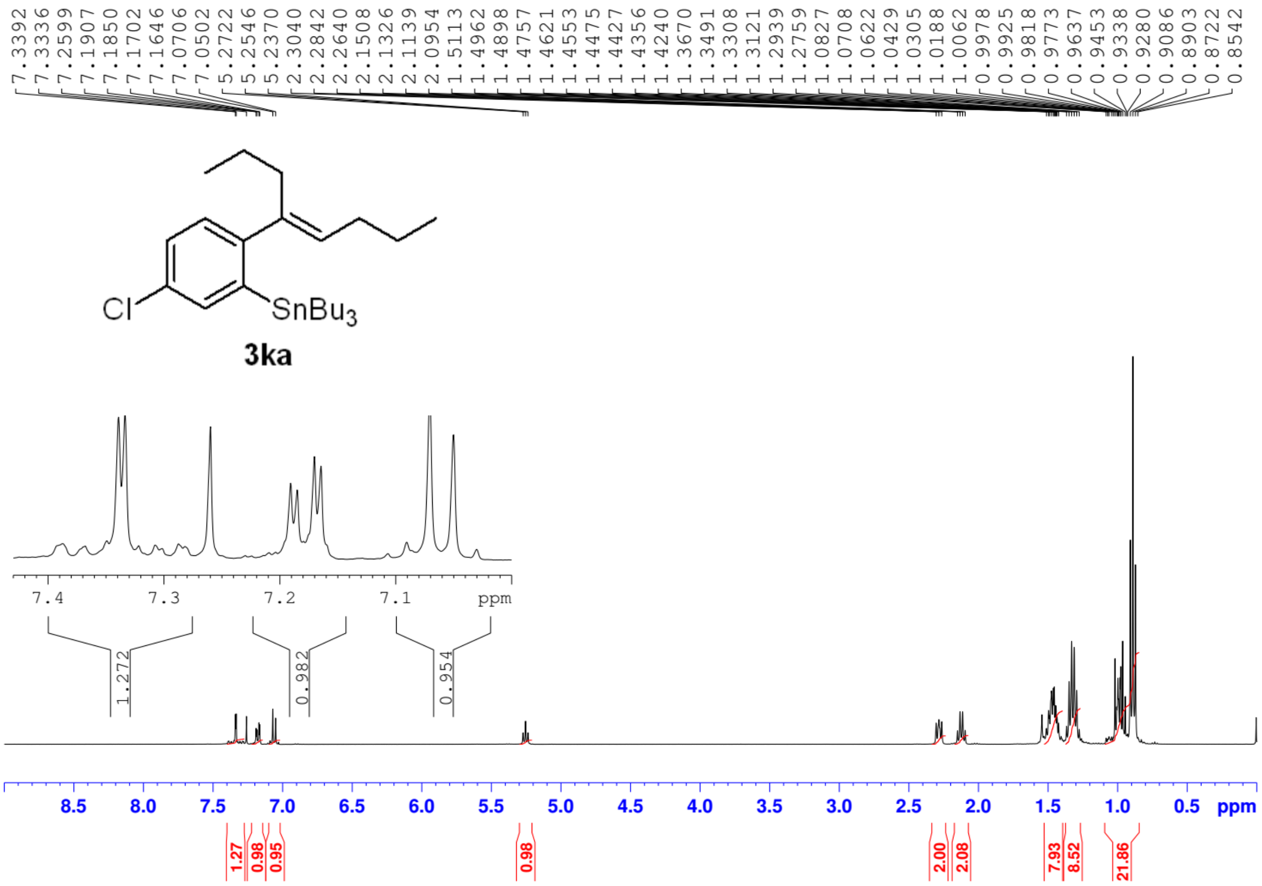
7.3392
7.3336
7.2599
7.1907
7.1850
7.1702
7.1646
7.0706
7.0502
5.2722
5.2546
5.2370
2.3040
2.2842
2.2640
2.1508
2.1326
2.1139
2.0954
1.5113
1.4962
1.4898
1.4757
1.4621
1.4553
1.4475
1.4427
1.4356
1.4240
1.3670
1.3491
1.3308
1.3121
1.2939
1.2759
1.0827
1.0708
1.0622
1.0429
1.0305
1.0188
1.0062
0.9978
0.9925
0.9818
0.9773
0.9637
0.9453
0.9338
0.9280
0.9086
0.8903
0.8722
0.8542
Cl
SnBu3
3ka
7.4
7.3
7.2
7.1
ppm
1.272
0.982
0.954
8.5
8.0
7.5
7.0
6.5
6.0
5.5
5.0
4.5
4.0
3.5
3.0
2.5
2.0
1.5
1.0
0.5
ppm
1.27
0.98
0.95
0.98
2.00
2.08
7.93
8.52
21.86

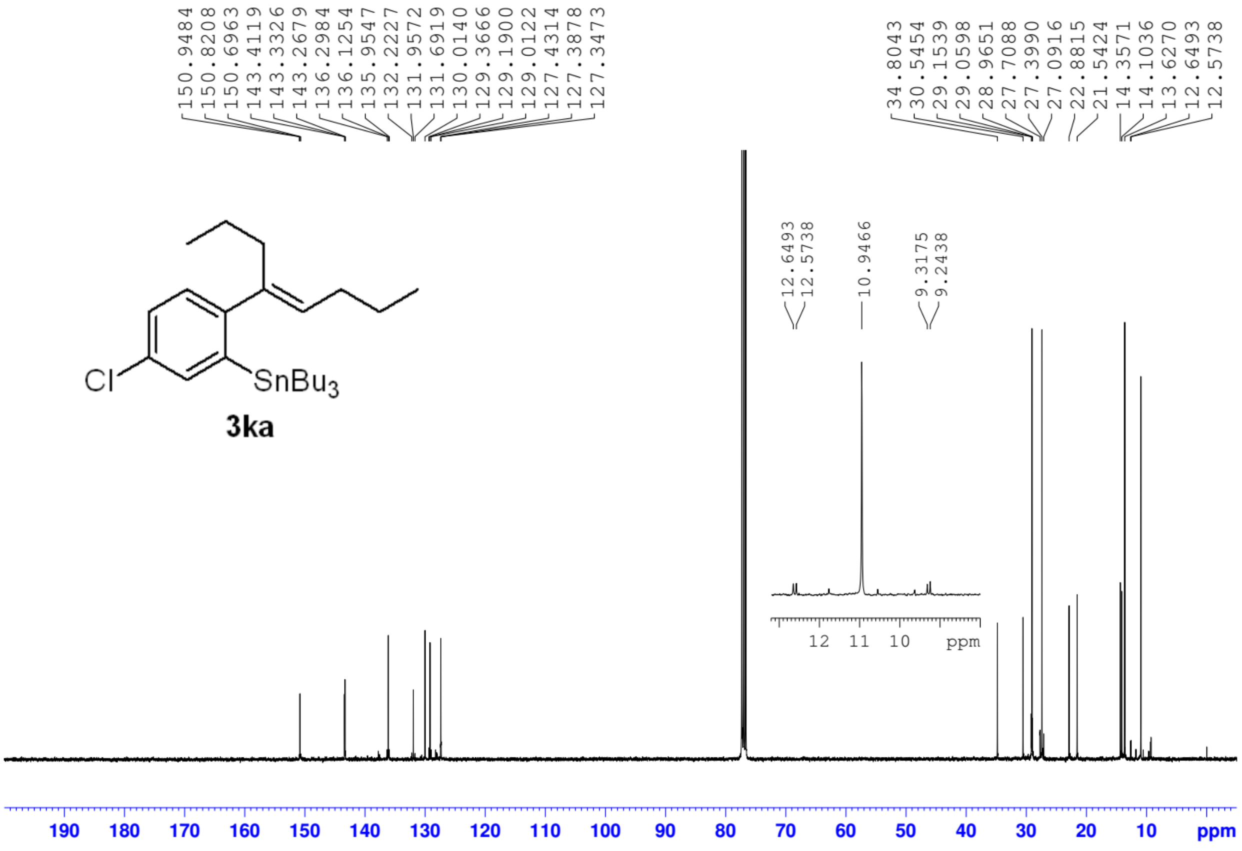
150.9484
150.8208
150.6963
143.4119
143.3326
143.2679
136.2984
136.1254
135.9547
132.2227
131.9572
131.6919
130.0140
129.3666
129.1900
129.0122
127.4314
127.3878
127.3473
34.8043
30.5454
29.1539
29.0598
28.9651
27.7088
27.3990
27.0916
22.8815
21.5424
14.3571
14.1036
13.6270
12.6493
12.5738
12.6493
12.5738
10.9466
9.3175
9.2438
Cl
SnBu3
3ka
12
11
10
ppm
190
180
170
160
150
140
130
120
110
100
90
80
70
60
50
40
30
20
10
ppm

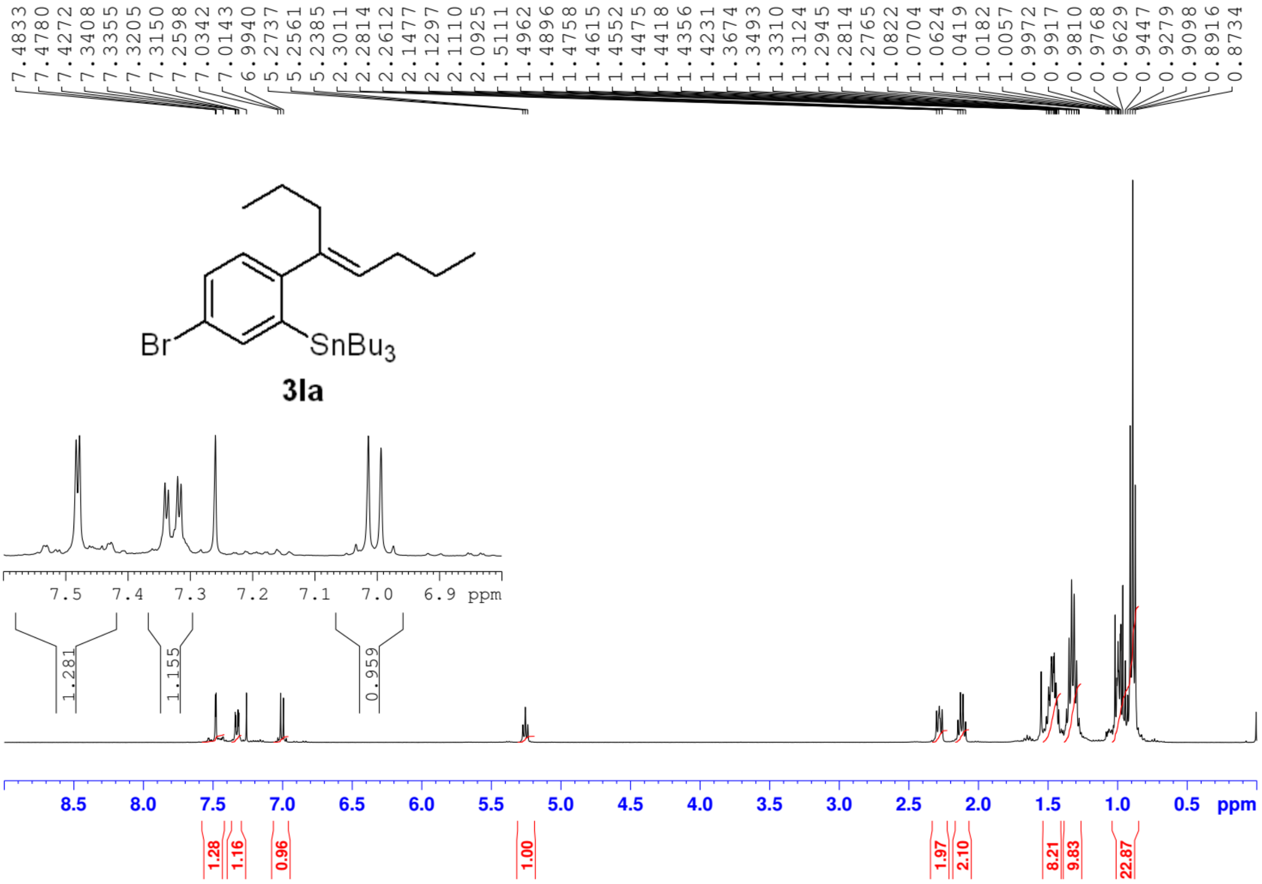
7.4833
7.4780
7.4272
7.3408
7.3355
7.3205
7.3150
7.2598
7.0342
7.0143
6.9940
5.2737
5.2561
5.2385
2.3011
2.2814
2.2612
2.1477
2.1297
2.1110
2.0925
1.5111
1.4962
1.4896
1.4758
1.4615
1.4552
1.4475
1.4418
1.4356
1.4231
1.3674
1.3493
1.3310
1.3124
1.2945
1.2814
1.2765
1.0822
1.0704
1.0624
1.0419
1.0182
1.0057
0.9972
0.9917
0.9810
0.9768
0.9629
0.9447
0.9279
0.9098
0.8916
0.8734
Br
SnBu3
3la
7.5 7.4 7.3 7.2 7.1 7.0 6.9 ppm
1.281
1.155
0.959
8.5 8.0 7.5 7.0 6.5 6.0 5.5 5.0 4.5 4.0 3.5 3.0 2.5 2.0 1.5 1.0 0.5 ppm
1.28
1.16
0.96
1.00
1.97
2.10
8.21
9.83
22.87

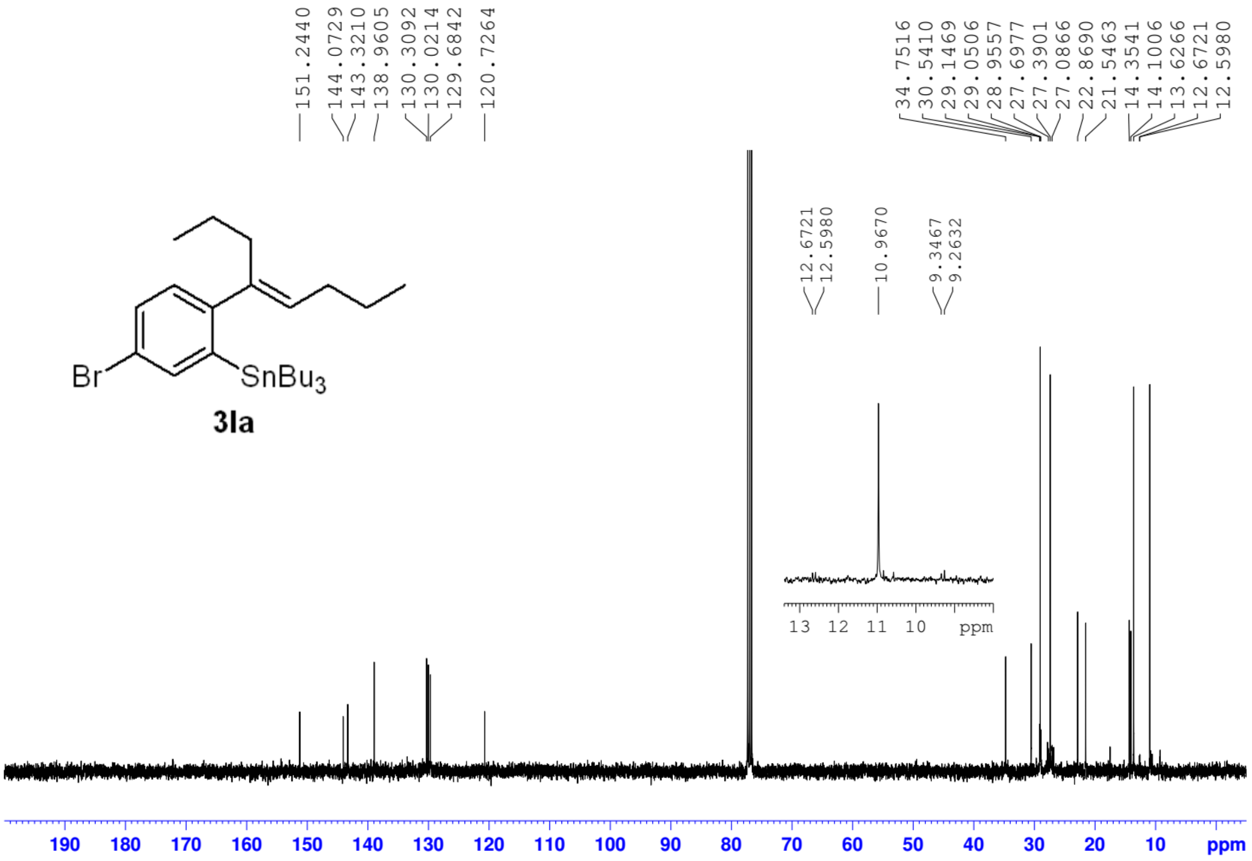
151.2440
144.0729
143.3210
138.9605
130.3092
130.0214
129.6842
120.7264
34.7516
30.5410
29.1469
29.0506
28.9557
27.6977
27.3901
27.0866
22.8690
21.5463
14.3541
14.1006
13.6266
12.6721
12.5980
12.6721
12.5980
10.9670
9.3467
9.2632
Br
SnBu3
3la
13 12 11 10 ppm
190 180 170 160 150 140 130 120 110 100 90 80 70 60 50 40 30 20 10 ppm

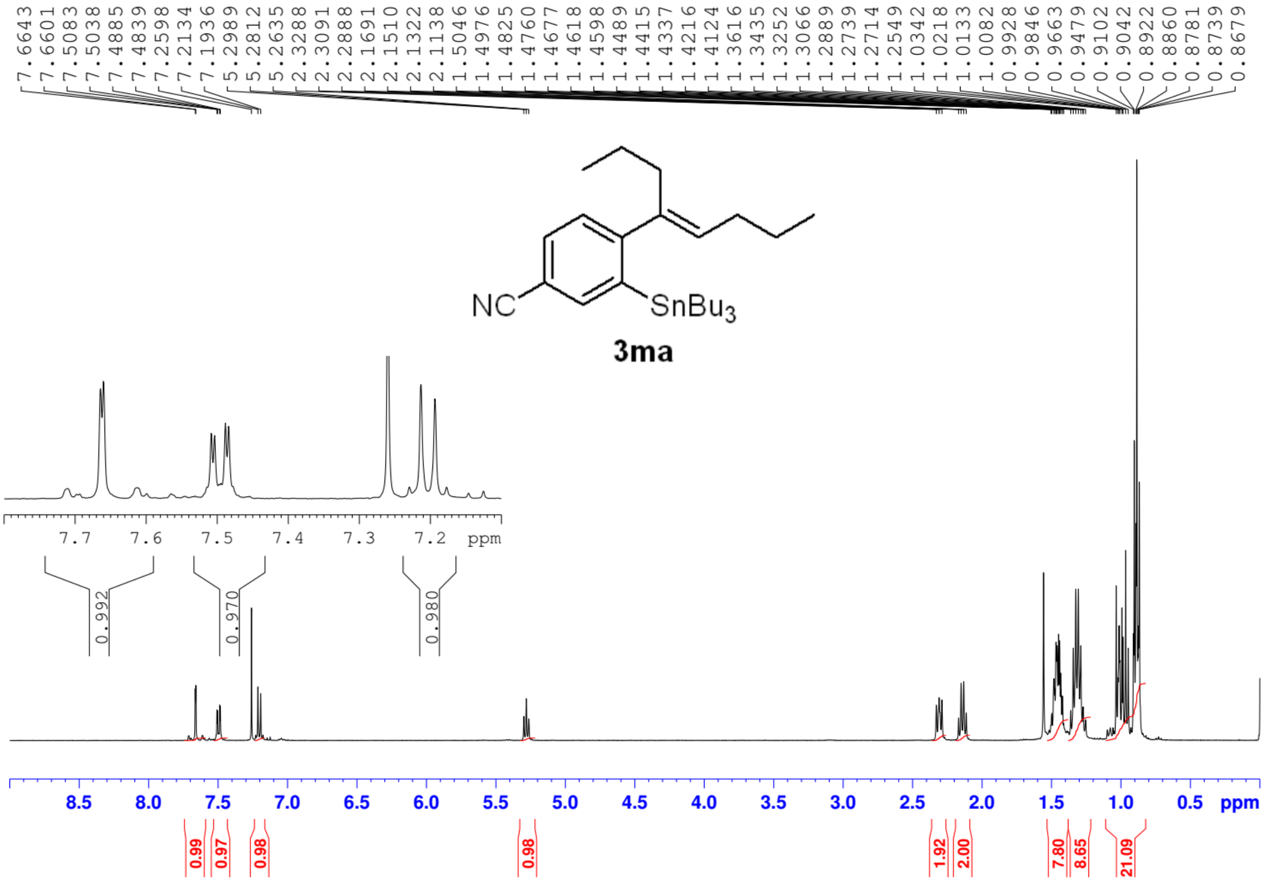
7.6643
7.6601
7.5083
7.5038
7.4885
7.4839
7.2598
7.2134
7.1936
5.2989
5.2812
5.2635
2.3288
2.3091
2.2888
2.1691
2.1510
2.1322
2.1138
1.5046
1.4976
1.4825
1.4760
1.4677
1.4618
1.4598
1.4489
1.4415
1.4337
1.4216
1.4124
1.3616
1.3435
1.3252
1.3066
1.2889
1.2739
1.2714
1.2549
1.0342
1.0218
1.0133
1.0082
0.9928
0.9846
0.9663
0.9479
0.9102
0.9042
0.8922
0.8860
0.8781
0.8739
0.8679
NC
SnBu3
3ma
7.7 7.6 7.5 7.4 7.3 7.2 ppm
0.992
0.970
0.980
8.5 8.0 7.5 7.0 6.5 6.0 5.5 5.0 4.5 4.0 3.5 3.0 2.5 2.0 1.5 1.0 0.5 ppm
0.99 0.97 0.98 0.98 1.92 2.00 7.80 8.65 21.09

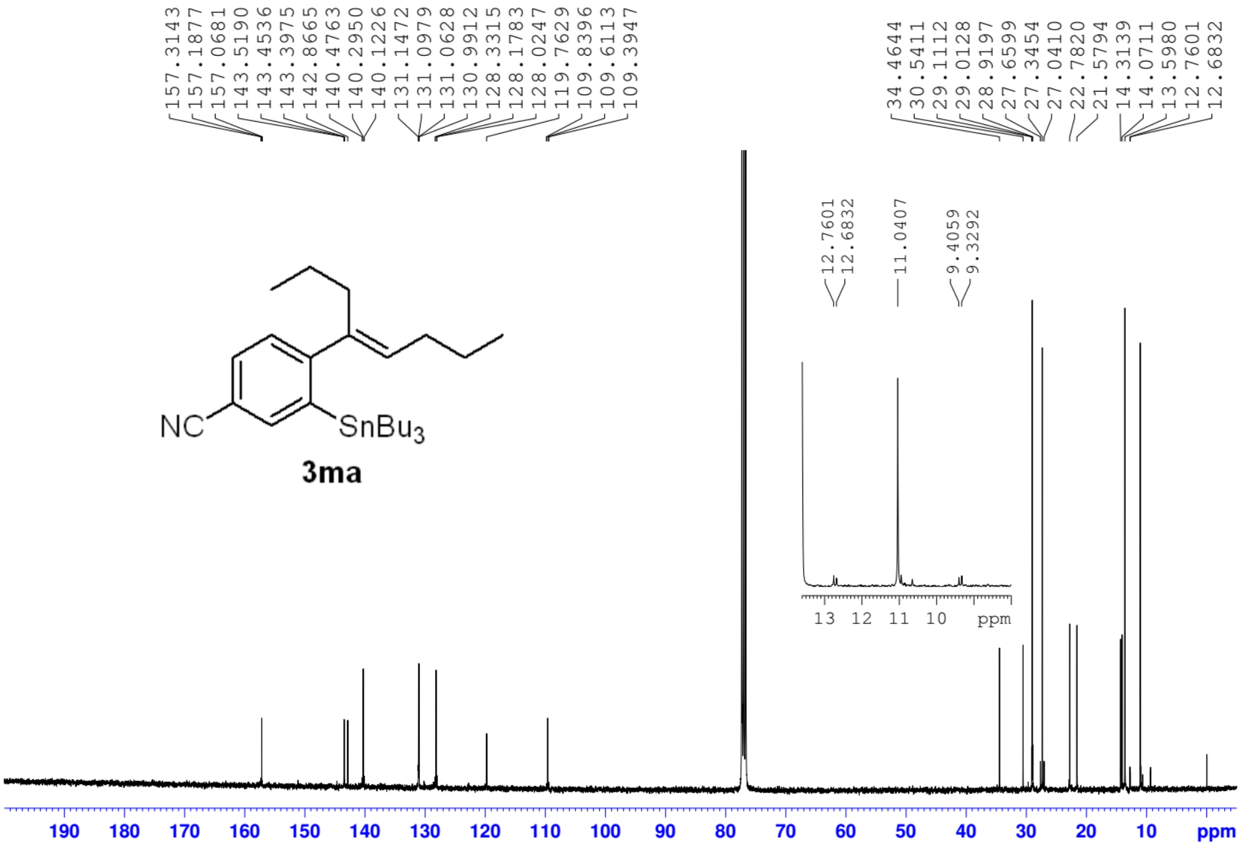
157.3143
157.1877
157.0681
143.5190
143.4536
143.3975
142.8665
140.4763
140.2950
140.1226
131.1472
131.0979
131.0628
130.9912
128.3315
128.1783
128.0247
119.7629
109.8396
109.6113
109.3947
34.4644
30.5411
29.1112
29.0128
28.9197
27.6599
27.3454
27.0410
22.7820
21.5794
14.3139
14.0711
13.5980
12.7601
12.6832
12.7601
12.6832
11.0407
9.4059
9.3292
NC
SnBu3
3ma
13 12 11 10 ppm
190 180 170 160 150 140 130 120 110 100 90 80 70 60 50 40 30 20 10 ppm

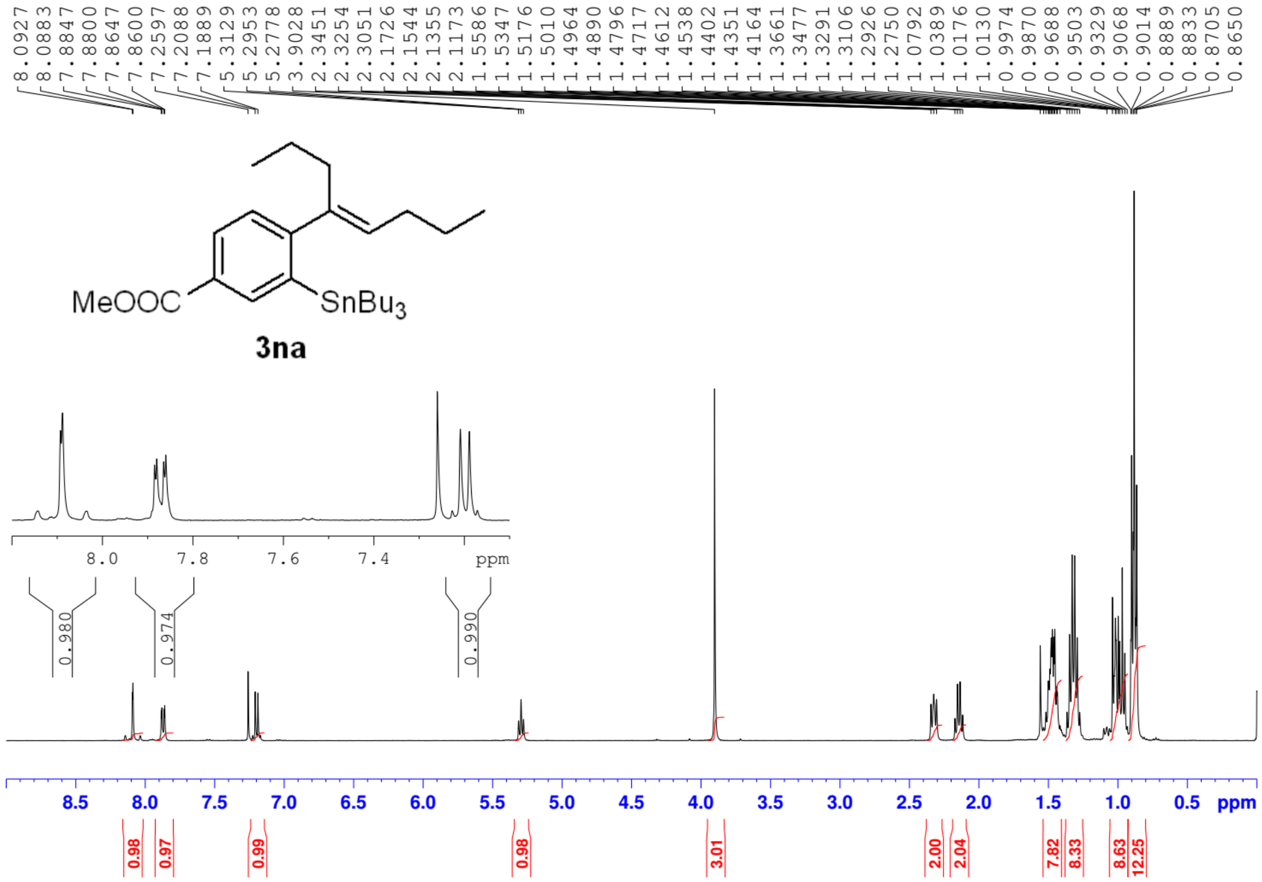
8.0927
8.0883
7.8847
7.8800
7.8647
7.8600
7.2597
7.2088
7.1889
5.3129
5.2953
5.2778
3.9028
2.3451
2.3254
2.3051
2.1726
2.1544
2.1355
2.1173
1.5586
1.5347
1.5176
1.5010
1.4964
1.4890
1.4796
1.4717
1.4612
1.4538
1.4402
1.4351
1.4164
1.3661
1.3477
1.3291
1.3106
1.2926
1.2750
1.0792
1.0389
1.0176
1.0130
0.9974
0.9870
0.9688
0.9503
0.9329
0.9068
0.9014
0.8889
0.8833
0.8705
0.8650
MeOOC
SnBu3
3na
0.980
0.974
0.990
ppm
0.98
0.97
0.99
0.98
3.01
2.00
2.04
7.82
8.33
8.63
12.25

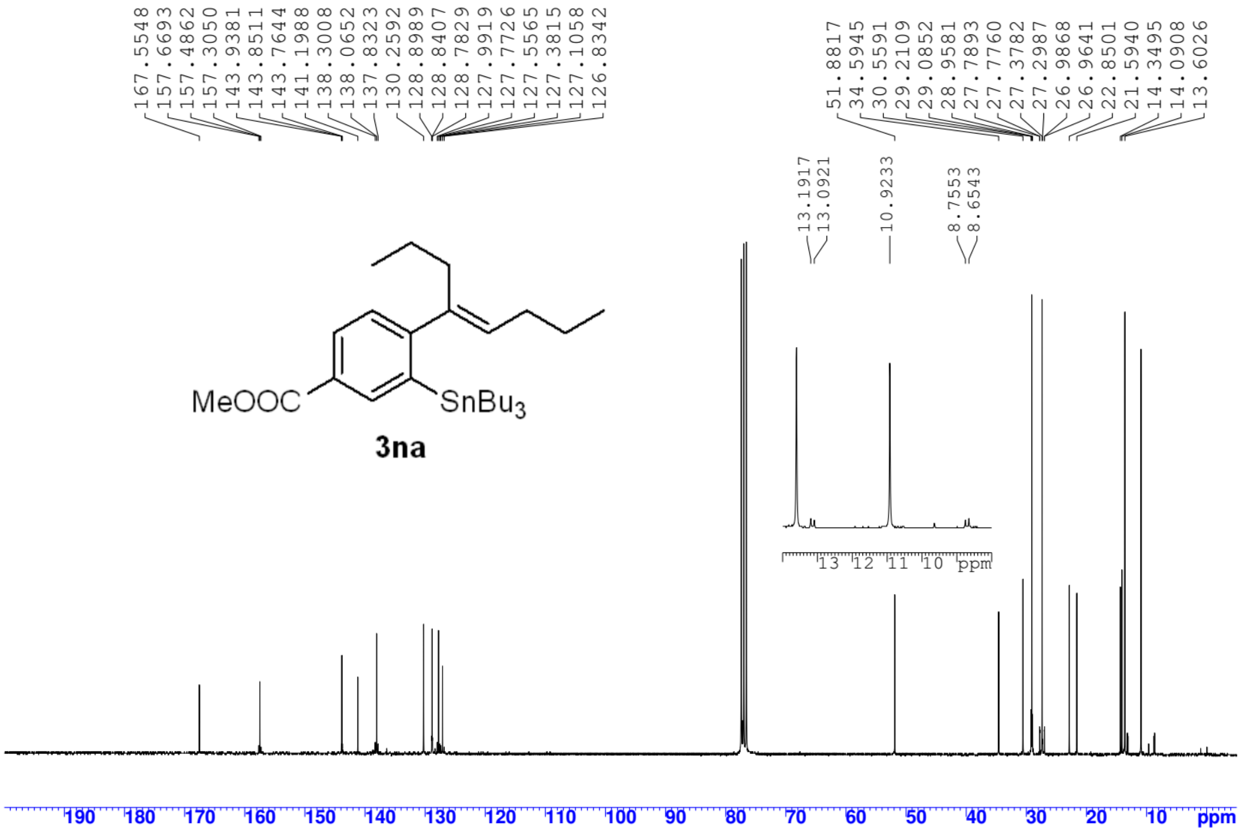
167.5548
157.6693
157.4862
157.3050
143.9381
143.8511
143.7644
141.1988
138.3008
138.0652
137.8323
130.2592
128.8989
128.8407
128.7829
127.9919
127.7726
127.5565
127.3815
127.1058
126.8342
51.8817
34.5945
30.5591
29.2109
29.0852
28.9581
27.7893
27.7760
27.3782
27.2987
26.9868
26.9641
22.8501
21.5940
14.3495
14.0908
13.6026
13.1917
13.0921
10.9233
8.7553
8.6543
MeOOC
SnBu3
3na
ppm

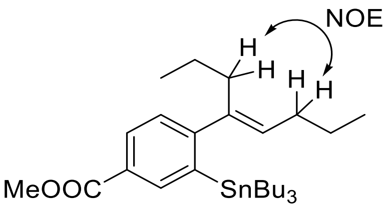
NOE
H
H
H
H
MeOOC
SnBu3

3na

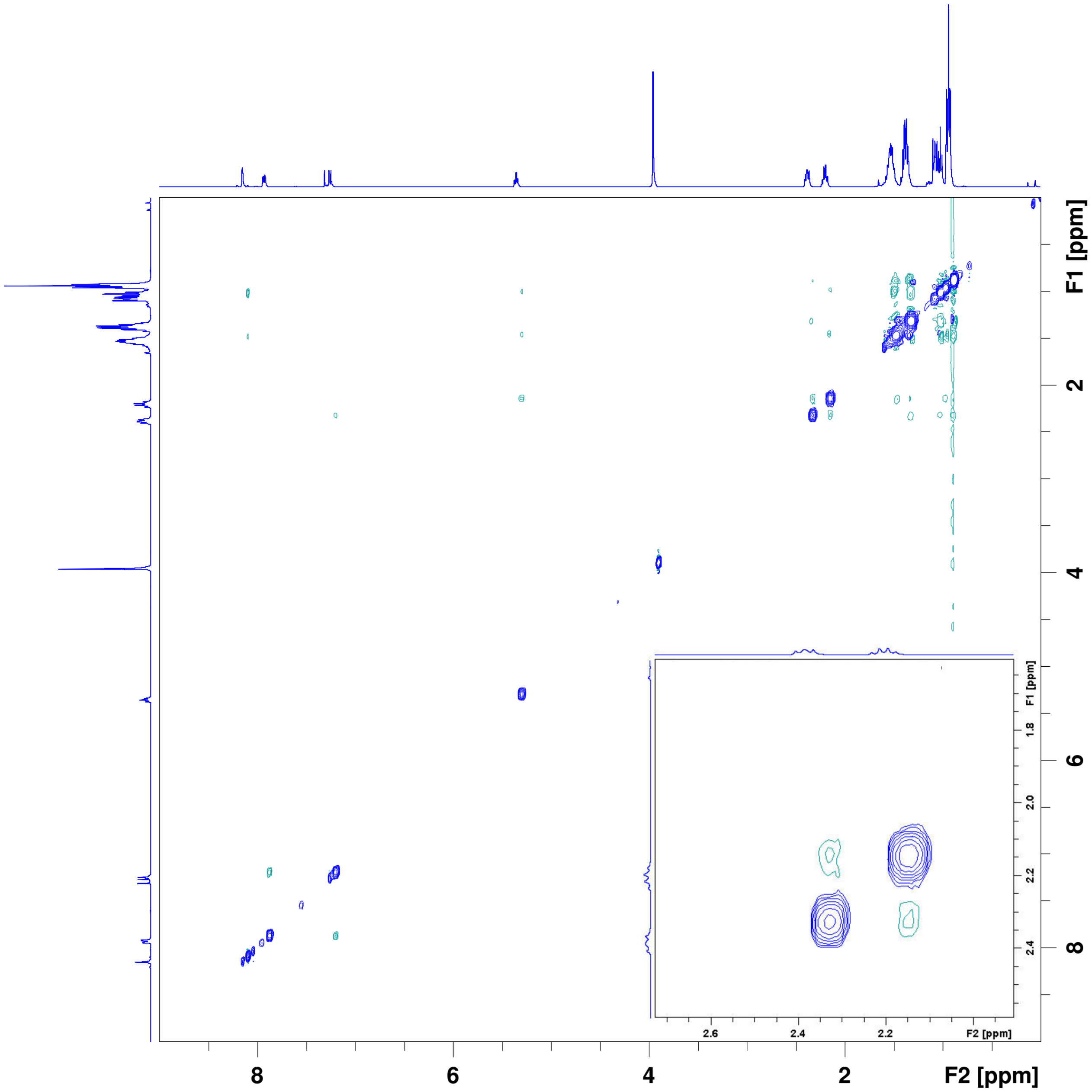
F1 [ppm]
2
4
6
8
F2 [ppm]
F1 [ppm]
1.8
2.0
2.2
2.4
2.6
2.4
2.2
F2 [ppm]

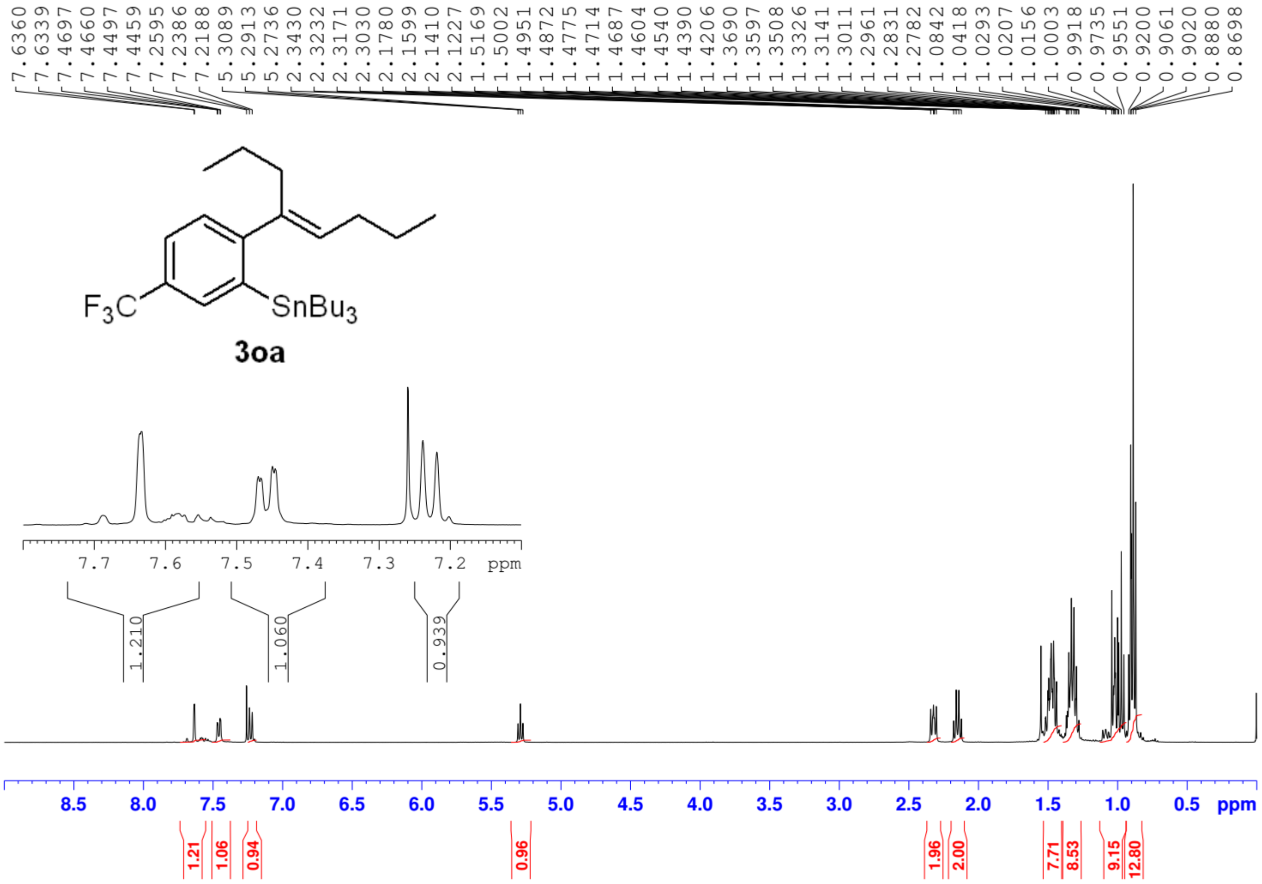
7.6360
7.6339
7.4697
7.4660
7.4497
7.4459
7.2595
7.2386
7.2188
5.3089
5.2913
5.2736
2.3430
2.3232
2.3171
2.3030
2.1780
2.1599
2.1410
2.1227
1.5169
1.5002
1.4951
1.4872
1.4775
1.4714
1.4687
1.4604
1.4540
1.4390
1.4206
1.3690
1.3597
1.3508
1.3326
1.3141
1.3011
1.2961
1.2831
1.2782
1.0842
1.0418
1.0293
1.0207
1.0156
1.0003
0.9918
0.9735
0.9551
0.9200
0.9061
0.9020
0.8880
0.8698
F3C
SnBu3
3oa
7.7 7.6 7.5 7.4 7.3 7.2 ppm
1.210
1.060
0.939
8.5 8.0 7.5 7.0 6.5 6.0 5.5 5.0 4.5 4.0 3.5 3.0 2.5 2.0 1.5 1.0 0.5 ppm
1.21
1.06
0.94
0.96
1.96
2.00
7.71
8.53
9.15
12.80

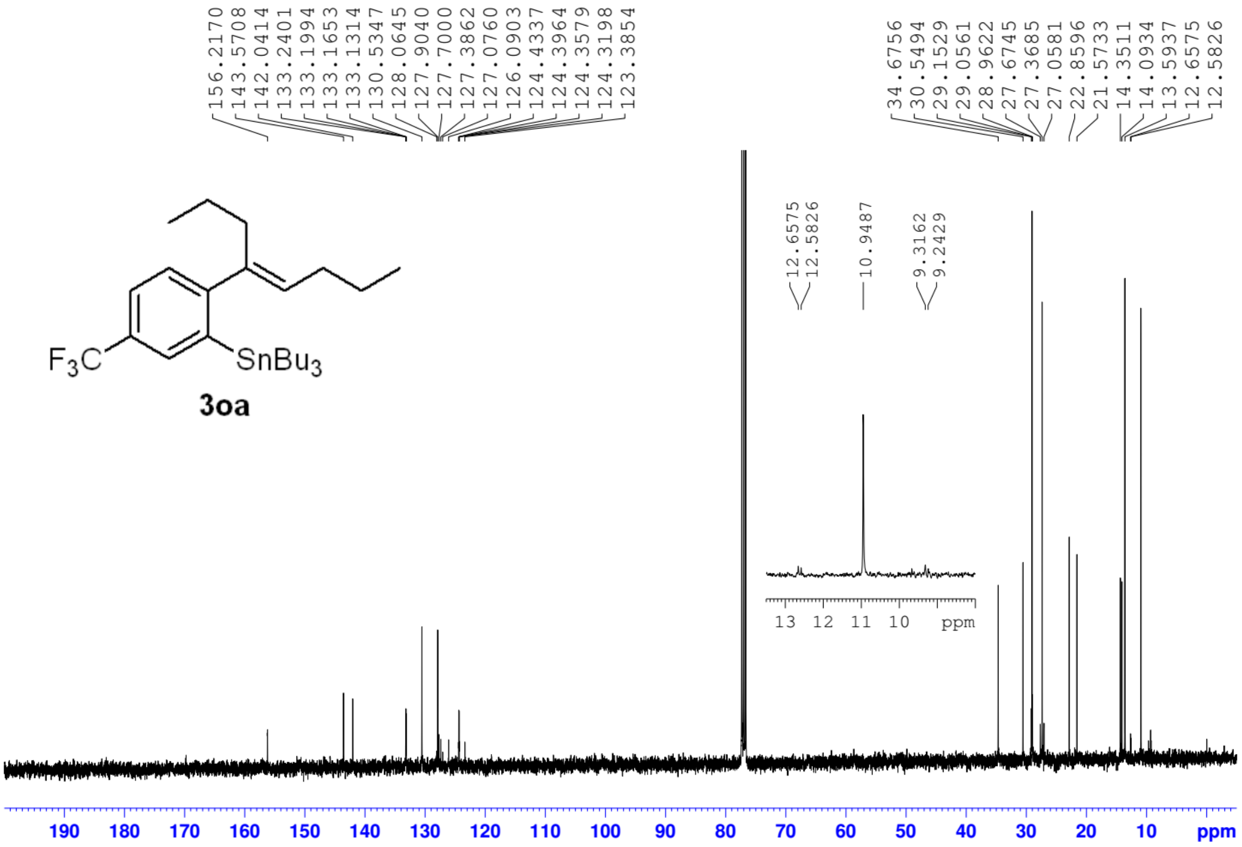
156.2170
143.5708
142.0414
133.2401
133.1994
133.1653
133.1314
130.5347
128.0645
127.9040
127.7000
127.3862
127.0760
126.0903
124.4337
124.3964
124.3579
124.3198
123.3854
34.6756
30.5494
29.1529
29.0561
28.9622
27.6745
27.3685
27.0581
22.8596
21.5733
14.3511
14.0934
13.5937
12.6575
12.5826
12.6575
12.5826
10.9487
9.3162
9.2429
F3C
SnBu3
3oa
13 12 11 10 ppm
190 180 170 160 150 140 130 120 110 100 90 80 70 60 50 40 30 20 10 ppm

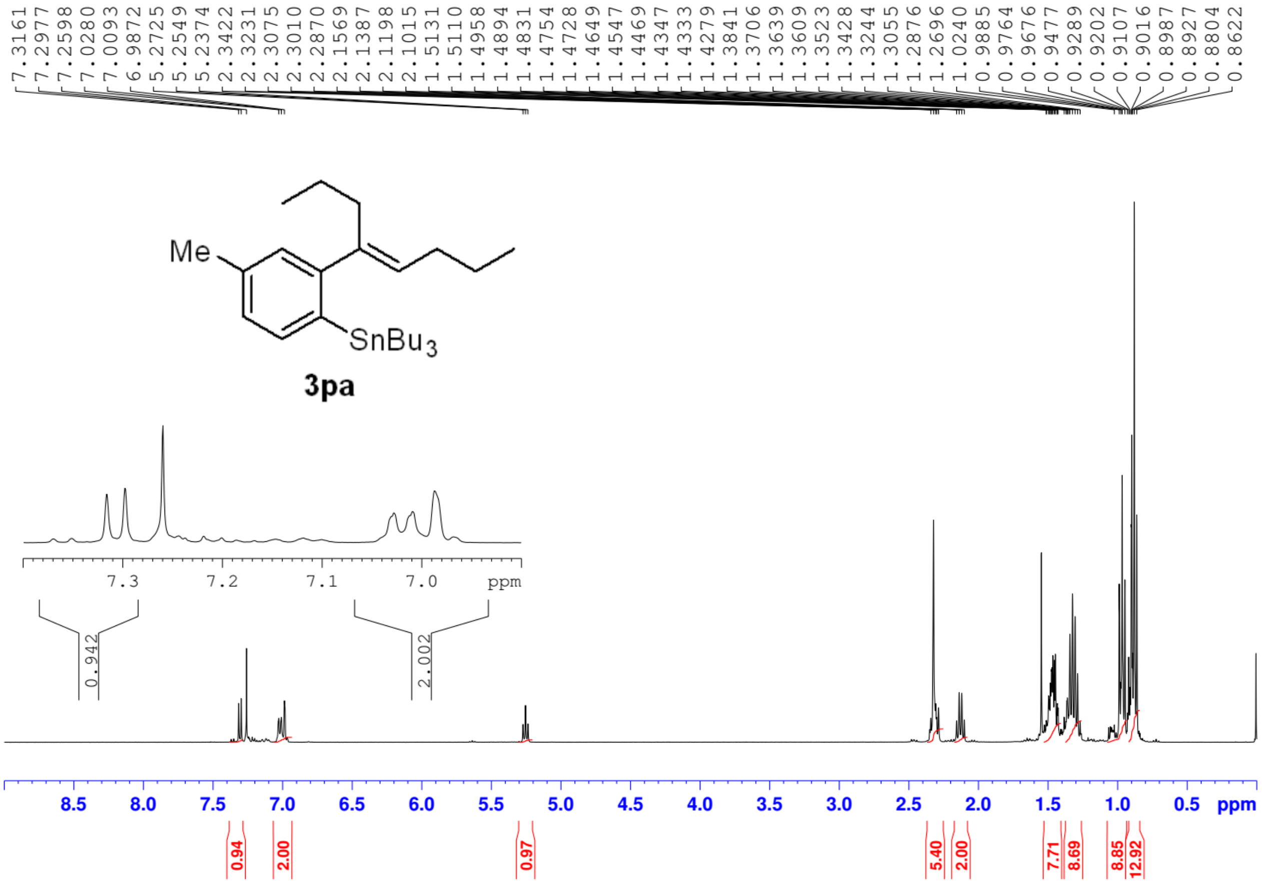

Me
SnBu3
3pa
ppm

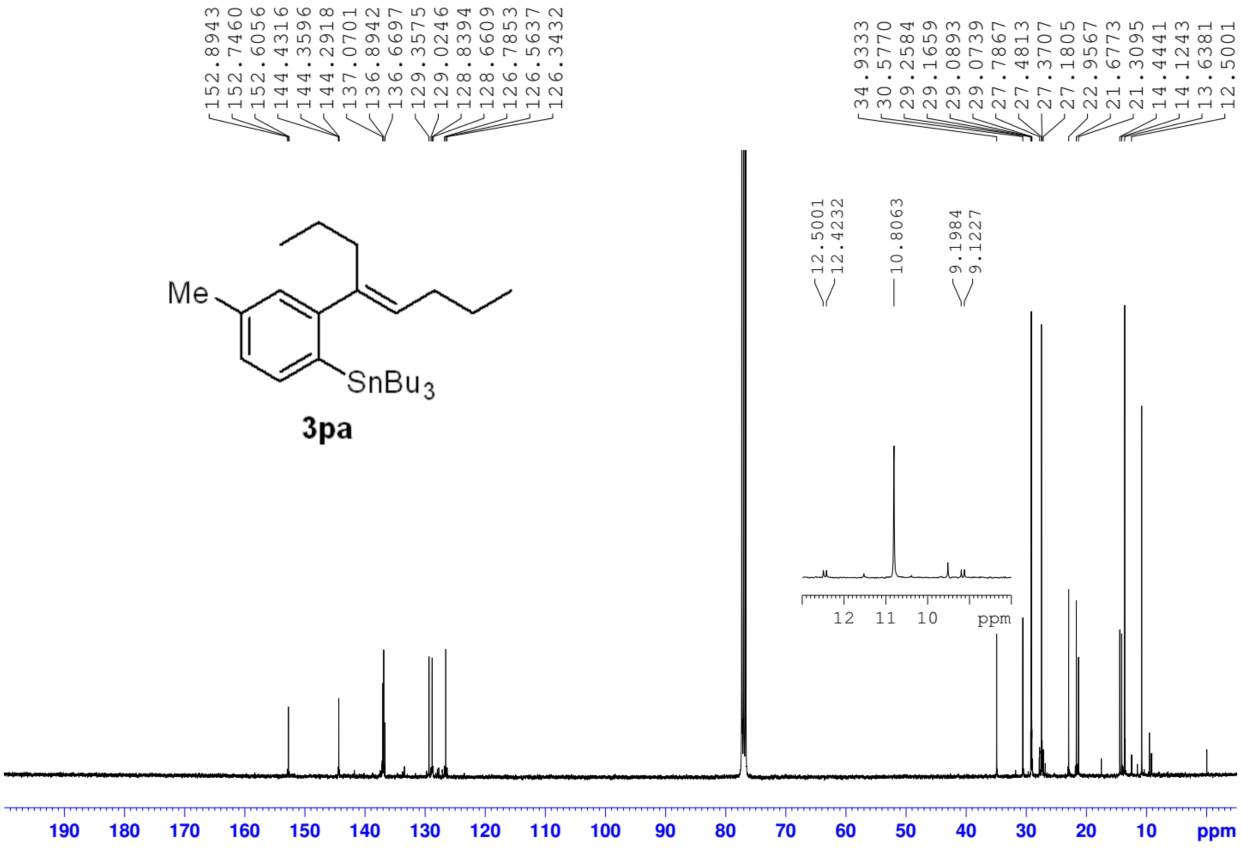

Me
SnBu3
3pa
ppm

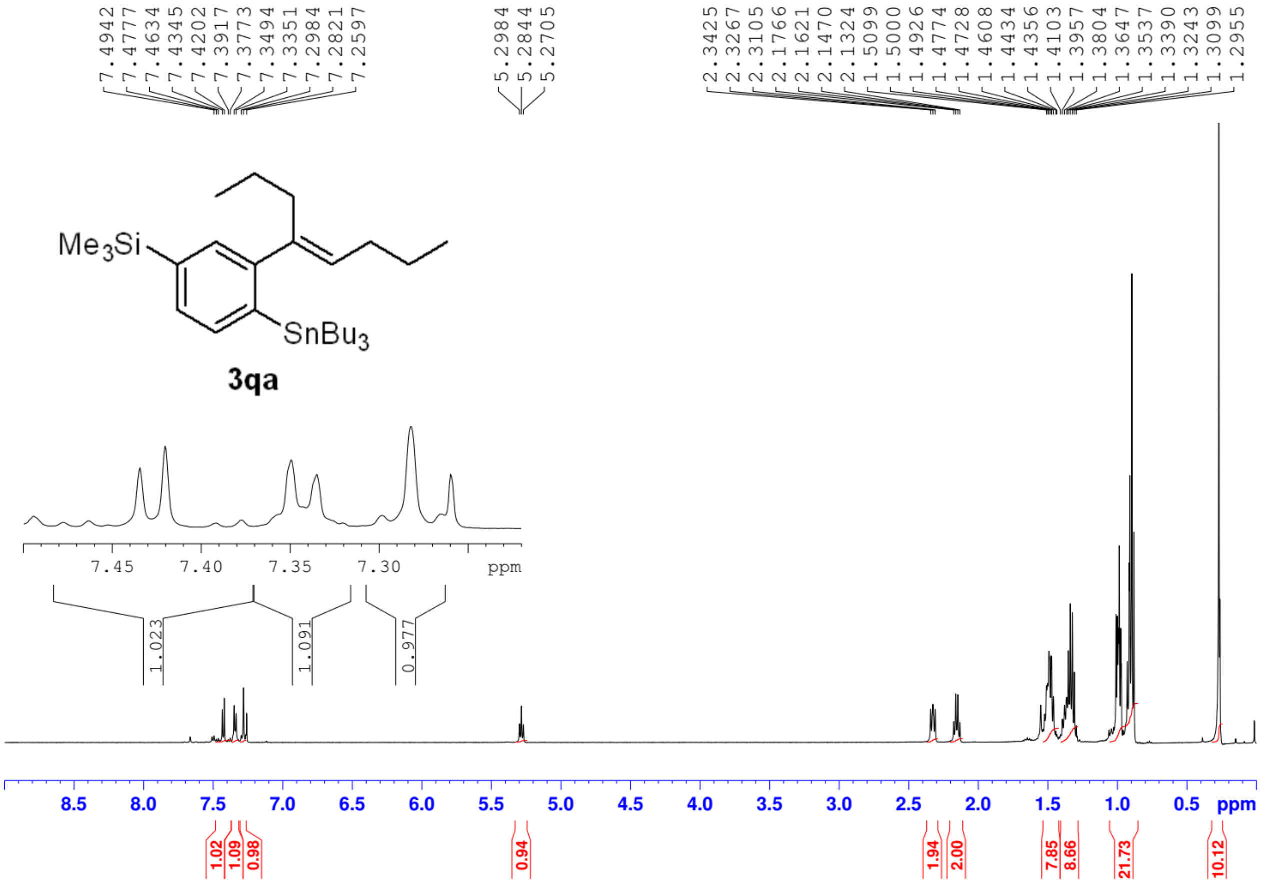
7.4942
7.4777
7.4634
7.4345
7.4202
7.3917
7.3773
7.3494
7.3351
7.2984
7.2821
7.2597
5.2984
5.2844
5.2705
2.3425
2.3267
2.3105
2.1766
2.1621
2.1470
2.1324
1.5099
1.5000
1.4926
1.4774
1.4728
1.4608
1.4434
1.4356
1.4103
1.3957
1.3804
1.3647
1.3537
1.3390
1.3243
1.3099
1.2955
Me3Si
SnBu3
3qa
7.45
7.40
7.35
7.30
ppm
1.023
1.091
0.977
8.5
8.0
7.5
7.0
6.5
6.0
5.5
5.0
4.5
4.0
3.5
3.0
2.5
2.0
1.5
1.0
0.5
ppm
1.02
1.09
0.98
0.94
1.94
2.00
7.85
8.66
21.73
10.12

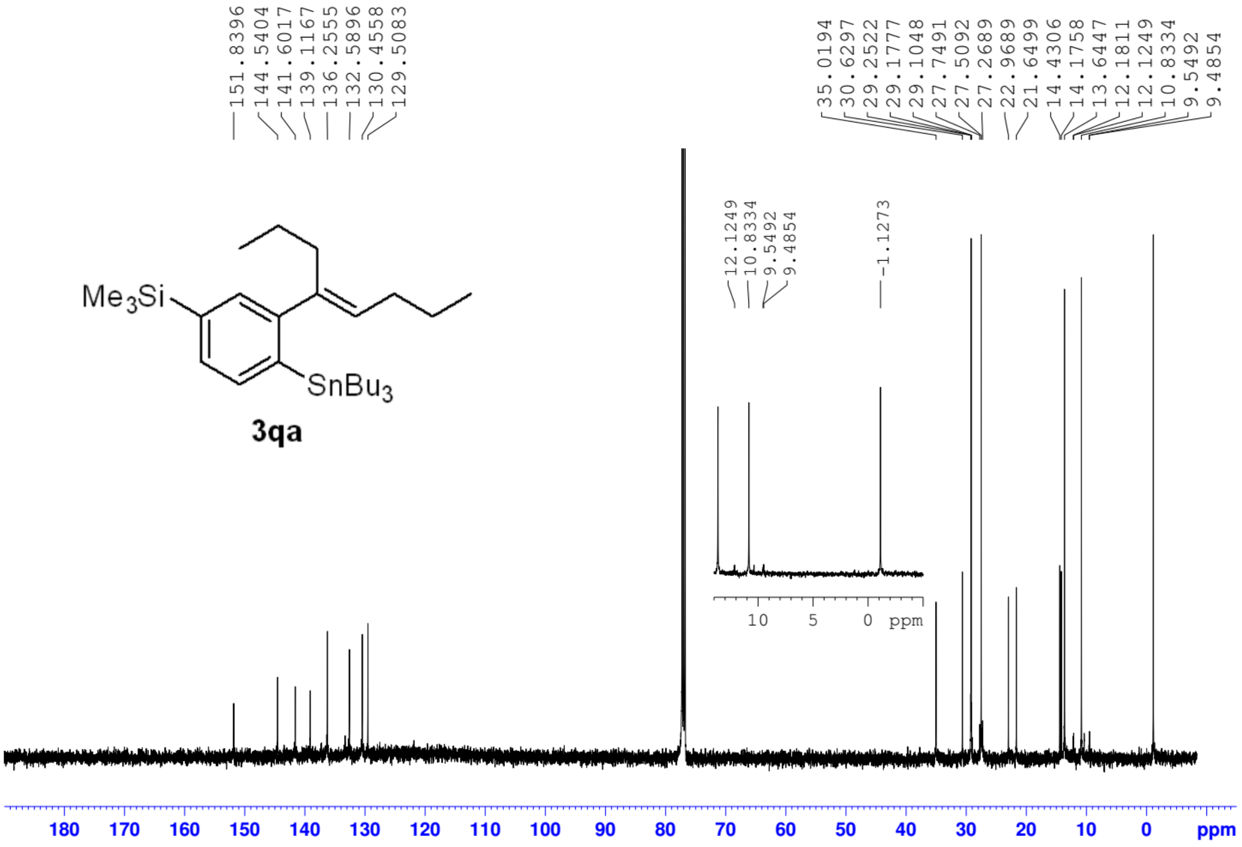
151.8396
144.5404
141.6017
139.1167
136.2555
132.5896
130.4558
129.5083
35.0194
30.6297
29.2522
29.1777
29.1048
27.7491
27.5092
27.2689
22.9689
21.6499
14.4306
14.1758
13.6447
12.1811
12.1249
10.8334
9.5492
9.4854
Me3Si
SnBu3
3qa
12.1249
10.8334
9.5492
9.4854
-1.1273
10
5
0
ppm
180
170
160
150
140
130
120
110
100
90
80
70
60
50
40
30
20
10
0
ppm

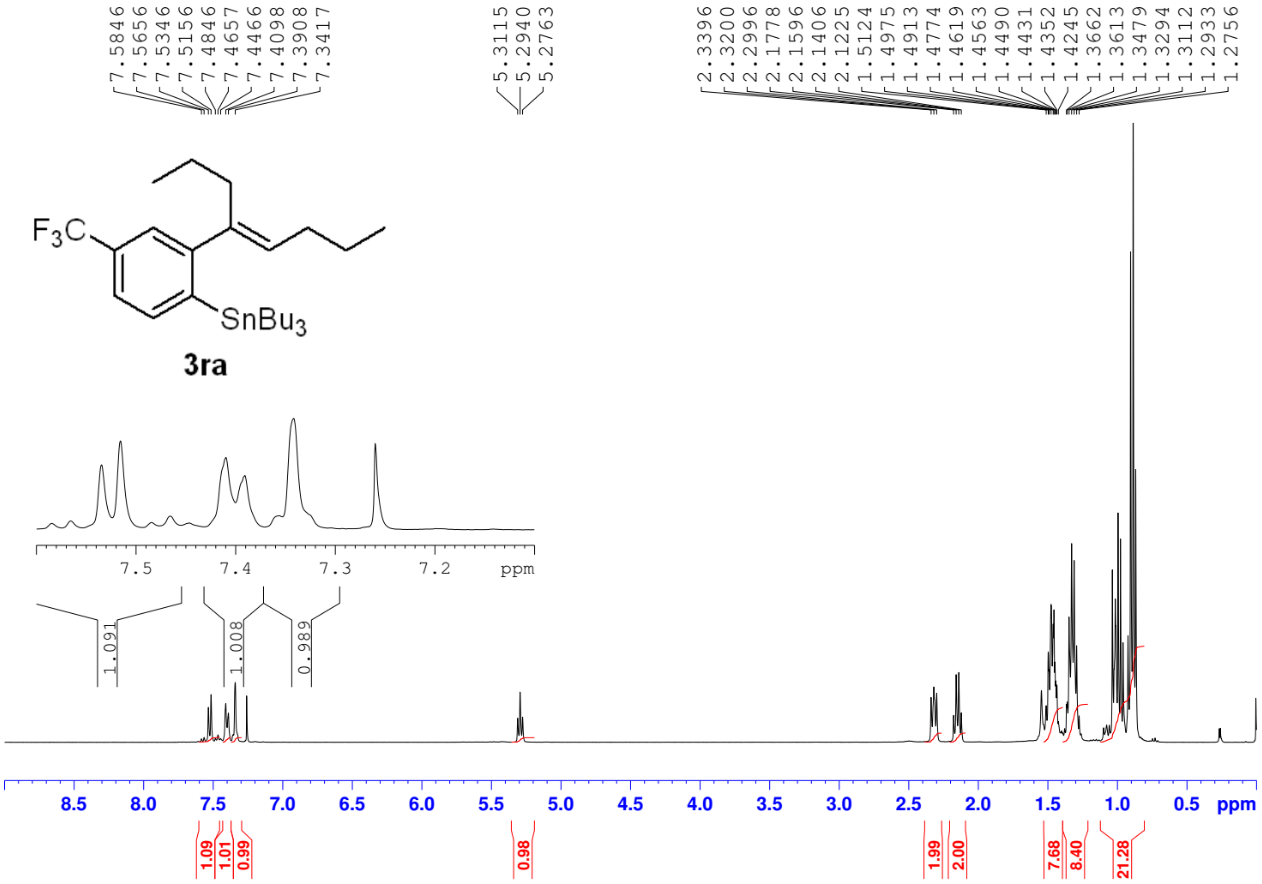
7.5846
7.5656
7.5346
7.5156
7.4846
7.4657
7.4466
7.4098
7.3908
7.3417
5.3115
5.2940
5.2763
2.3396
2.3200
2.2996
2.1778
2.1596
2.1406
2.1225
1.5124
1.4975
1.4913
1.4774
1.4619
1.4563
1.4490
1.4431
1.4352
1.4245
1.3662
1.3613
1.3479
1.3294
1.3112
1.2933
1.2756
F3C
SnBu3
3ra
1.091
1.008
0.989
1.09
1.01
0.99
0.98
1.99
2.00
7.68
8.40
21.28
ppm

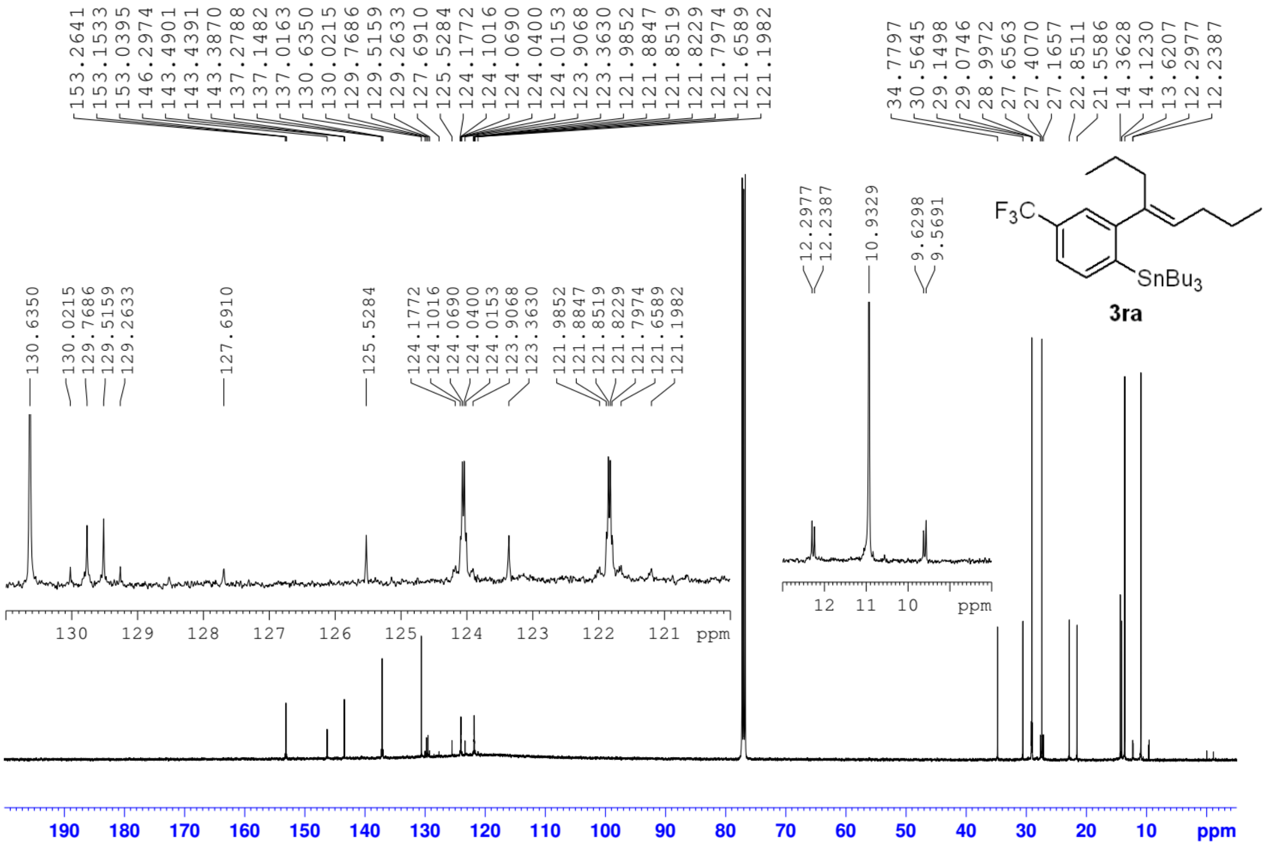
153.2641
153.1533
153.0395
146.2974
143.4901
143.4391
143.3870
137.2788
137.1482
137.0163
130.6350
130.0215
129.7686
129.5159
129.2633
127.6910
125.5284
124.1772
124.1016
124.0690
124.0400
124.0153
123.9068
123.3630
121.9852
121.8847
121.8519
121.8229
121.7974
121.6589
121.1982
34.7797
30.5645
29.1498
29.0746
28.9972
27.6563
27.4070
27.1657
22.8511
21.5586
14.3628
14.1230
13.6207
12.2977
12.2387
10.9329
9.6298
9.5691
F3C
SnBu3
3ra
ppm

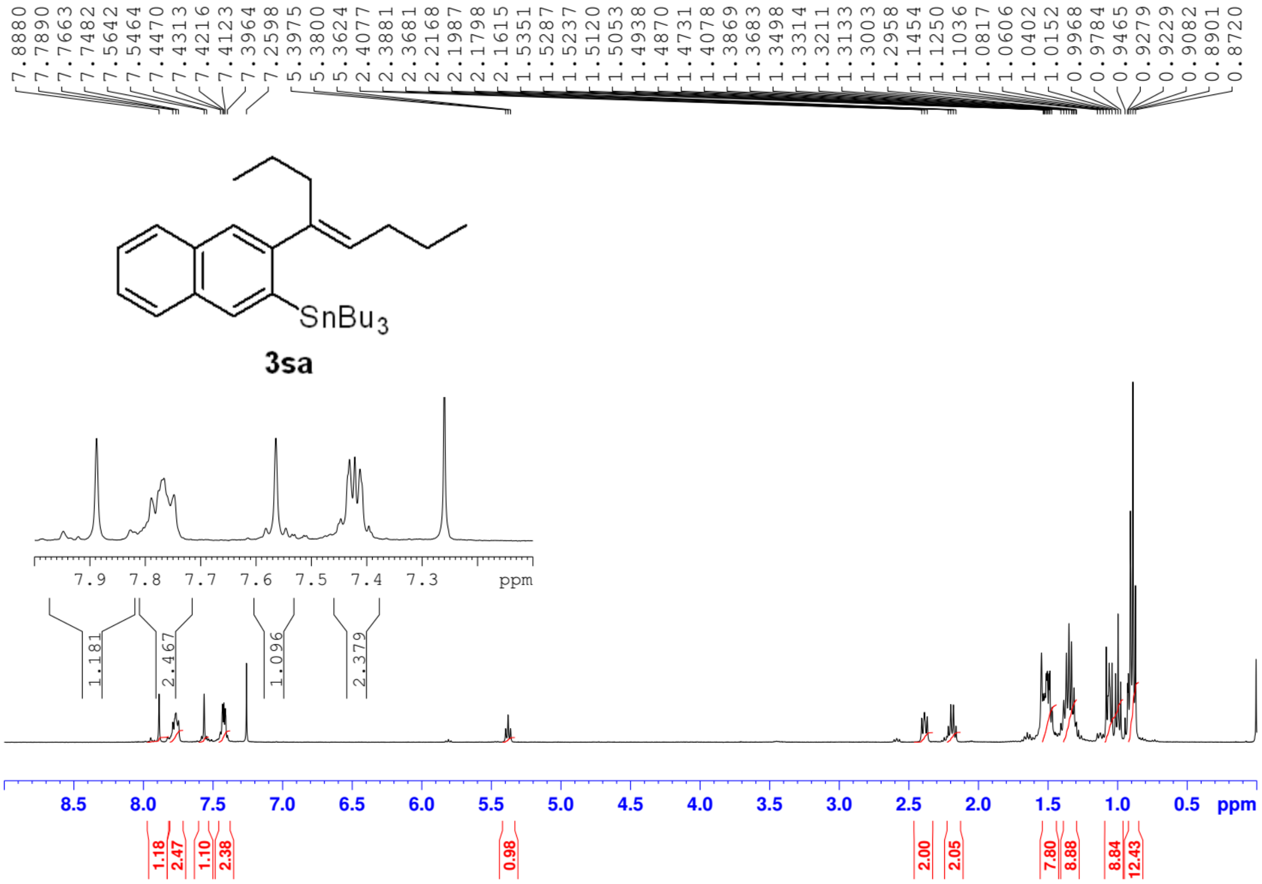
7.8880
7.7890
7.7663
7.7482
7.5642
7.5464
7.4470
7.4313
7.4216
7.4123
7.3964
7.2598
5.3975
5.3800
5.3624
2.4077
2.3881
2.3681
2.2168
2.1987
2.1798
2.1615
1.5351
1.5287
1.5237
1.5120
1.5053
1.4938
1.4870
1.4731
1.4078
1.3869
1.3683
1.3498
1.3314
1.3211
1.3133
1.3003
1.2958
1.1454
1.1250
1.1036
1.0817
1.0606
1.0402
1.0152
0.9968
0.9784
0.9465
0.9279
0.9229
0.9082
0.8901
0.8720
SnBu3
3sa
7.9 7.8 7.7 7.6 7.5 7.4 7.3 ppm
1.181
2.467
1.096
2.379
8.5 8.0 7.5 7.0 6.5 6.0 5.5 5.0 4.5 4.0 3.5 3.0 2.5 2.0 1.5 1.0 0.5 ppm
1.18
2.47
1.10
2.38
0.98
2.00
2.05
7.80
8.88
8.84
12.43

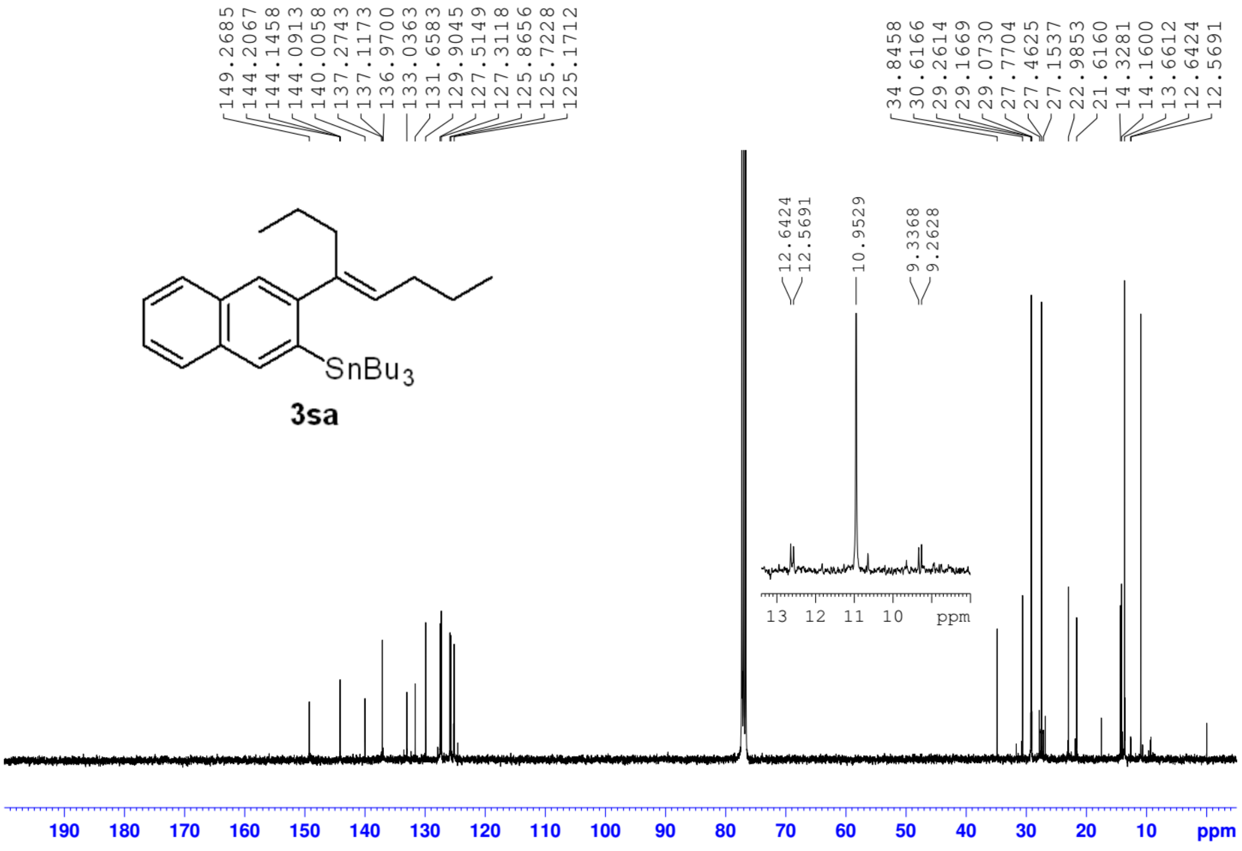
149.2685
144.2067
144.1458
144.0913
140.0058
137.2743
137.1173
136.9700
133.0363
131.6583
129.9045
127.5149
127.3118
125.8656
125.7228
125.1712
34.8458
30.6166
29.2614
29.1669
29.0730
27.7704
27.4625
27.1537
22.9853
21.6160
14.3281
14.1600
13.6612
12.6424
12.5691
SnBu3
3sa
12.6424
12.5691
10.9529
9.3368
9.2628
13 12 11 10 ppm
190 180 170 160 150 140 130 120 110 100 90 80 70 60 50 40 30 20 10 ppm

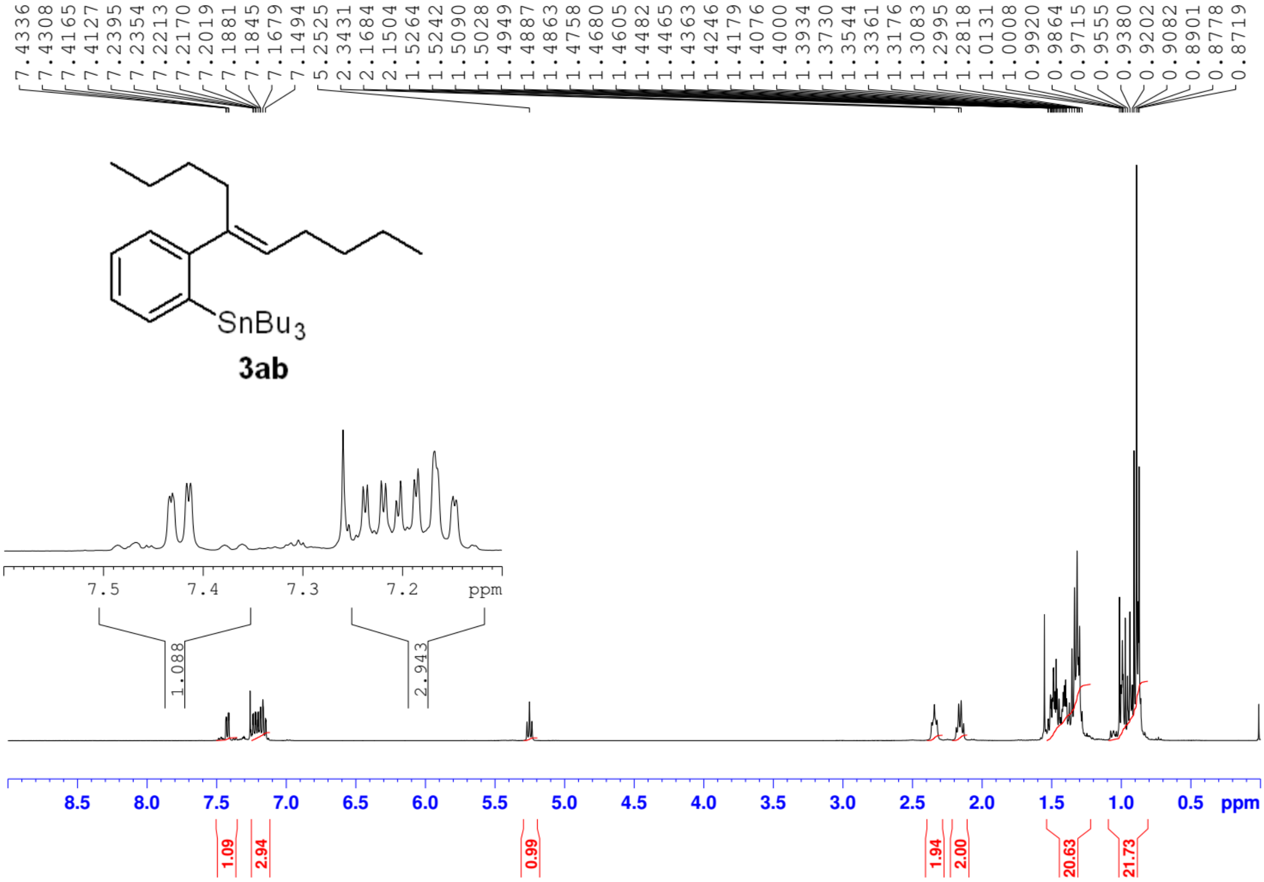
7.4336
7.4308
7.4165
7.4127
7.2395
7.2354
7.2213
7.2170
7.2019
7.1881
7.1845
7.1679
7.1494
5.2525
2.3431
2.1684
2.1504
1.5264
1.5242
1.5090
1.5028
1.4949
1.4887
1.4863
1.4758
1.4680
1.4605
1.4482
1.4465
1.4363
1.4246
1.4179
1.4076
1.4000
1.3934
1.3730
1.3544
1.3361
1.3176
1.3083
1.2995
1.2818
1.0131
1.0008
0.9920
0.9864
0.9715
0.9555
0.9380
0.9202
0.9082
0.8901
0.8778
0.8719
SnBu3
3ab
7.5 7.4 7.3 7.2 ppm
1.088
2.943
8.5 8.0 7.5 7.0 6.5 6.0 5.5 5.0 4.5 4.0 3.5 3.0 2.5 2.0 1.5 1.0 0.5 ppm
1.09 2.94 0.99 1.94 2.00 20.63 21.73

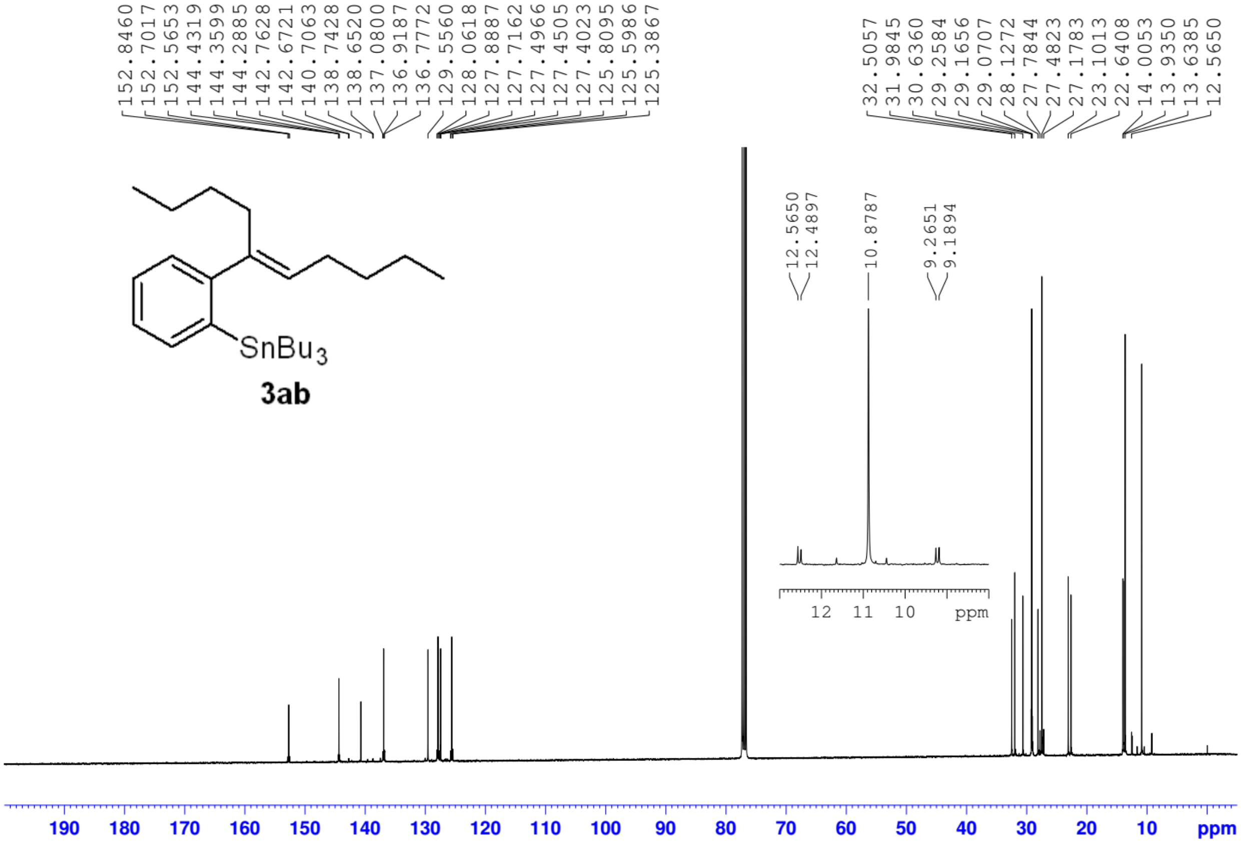
152.8460
152.7017
152.5653
144.4319
144.3599
144.2885
142.7628
142.6721
140.7063
138.7428
138.6520
137.0800
136.9187
136.7772
129.5560
128.0618
127.8887
127.7162
127.4966
127.4505
127.4023
125.8095
125.5986
125.3867
32.5057
31.9845
30.6360
29.2584
29.1656
29.0707
28.1272
27.7844
27.4823
27.1783
23.1013
22.6408
14.0053
13.9350
13.6385
12.5650
SnBu3
3ab
12.5650
12.4897
10.8787
9.2651
9.1894
12 11 10 ppm
190 180 170 160 150 140 130 120 110 100 90 80 70 60 50 40 30 20 10 ppm

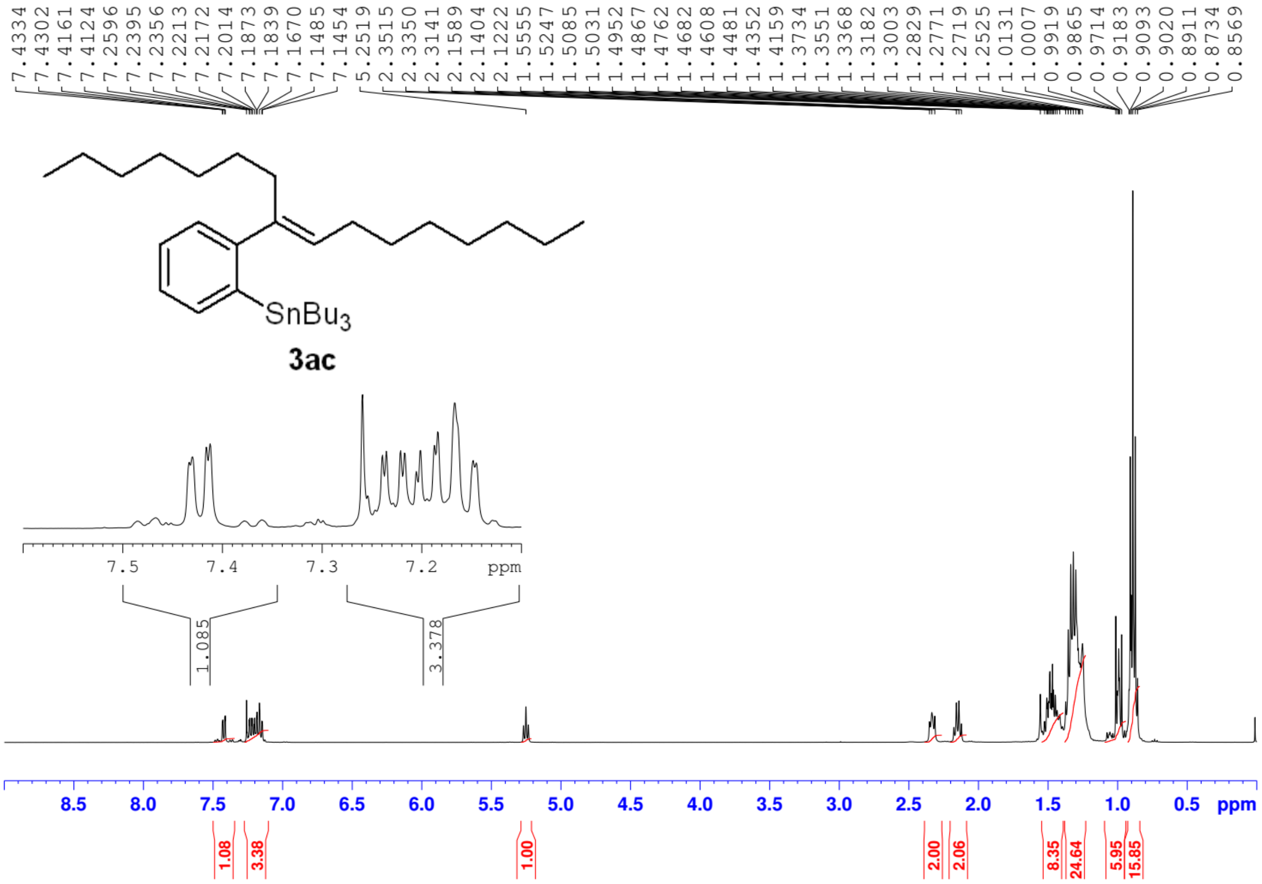
7.4334
7.4302
7.4161
7.4124
7.2596
7.2395
7.2356
7.2213
7.2172
7.2014
7.1873
7.1839
7.1670
7.1485
7.1454
5.2519
2.3515
2.3350
2.3141
2.1589
2.1404
2.1222
1.5555
1.5247
1.5085
1.5031
1.4952
1.4867
1.4762
1.4682
1.4608
1.4481
1.4352
1.4159
1.3734
1.3551
1.3368
1.3182
1.3003
1.2829
1.2771
1.2719
1.2525
1.0131
1.0007
0.9919
0.9865
0.9714
0.9183
0.9093
0.9020
0.8911
0.8734
0.8569
SnBu3
3ac
7.5
7.4
7.3
7.2
ppm
1.085
3.378
8.5
8.0
7.5
7.0
6.5
6.0
5.5
5.0
4.5
4.0
3.5
3.0
2.5
2.0
1.5
1.0
0.5
ppm
1.08
3.38
1.00
2.00
2.06
8.35
24.64
5.95
15.85

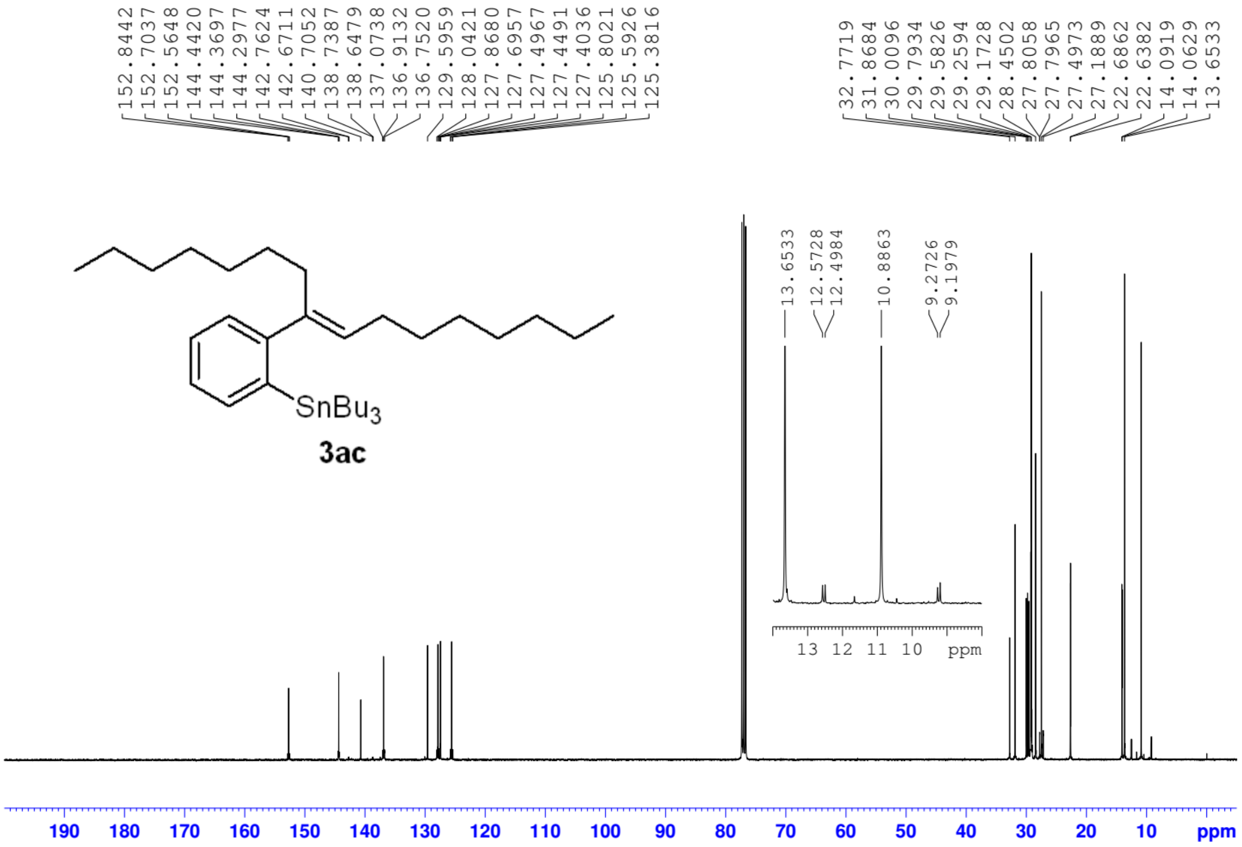
152.8442
152.7037
152.5648
144.4420
144.3697
144.2977
142.7624
142.6711
140.7052
138.7387
138.6479
137.0738
136.9132
136.7520
129.5959
128.0421
127.8680
127.6957
127.4967
127.4491
127.4036
125.8021
125.5926
125.3816
32.7719
31.8684
30.0096
29.7934
29.5826
29.2594
29.1728
28.4502
27.8058
27.7965
27.4973
27.1889
22.6862
22.6382
14.0919
14.0629
13.6533
13.6533
12.5728
12.4984
10.8863
9.2726
9.1979
13
12
11
10
ppm
SnBu3
3ac
190
180
170
160
150
140
130
120
110
100
90
80
70
60
50
40
30
20
10
ppm

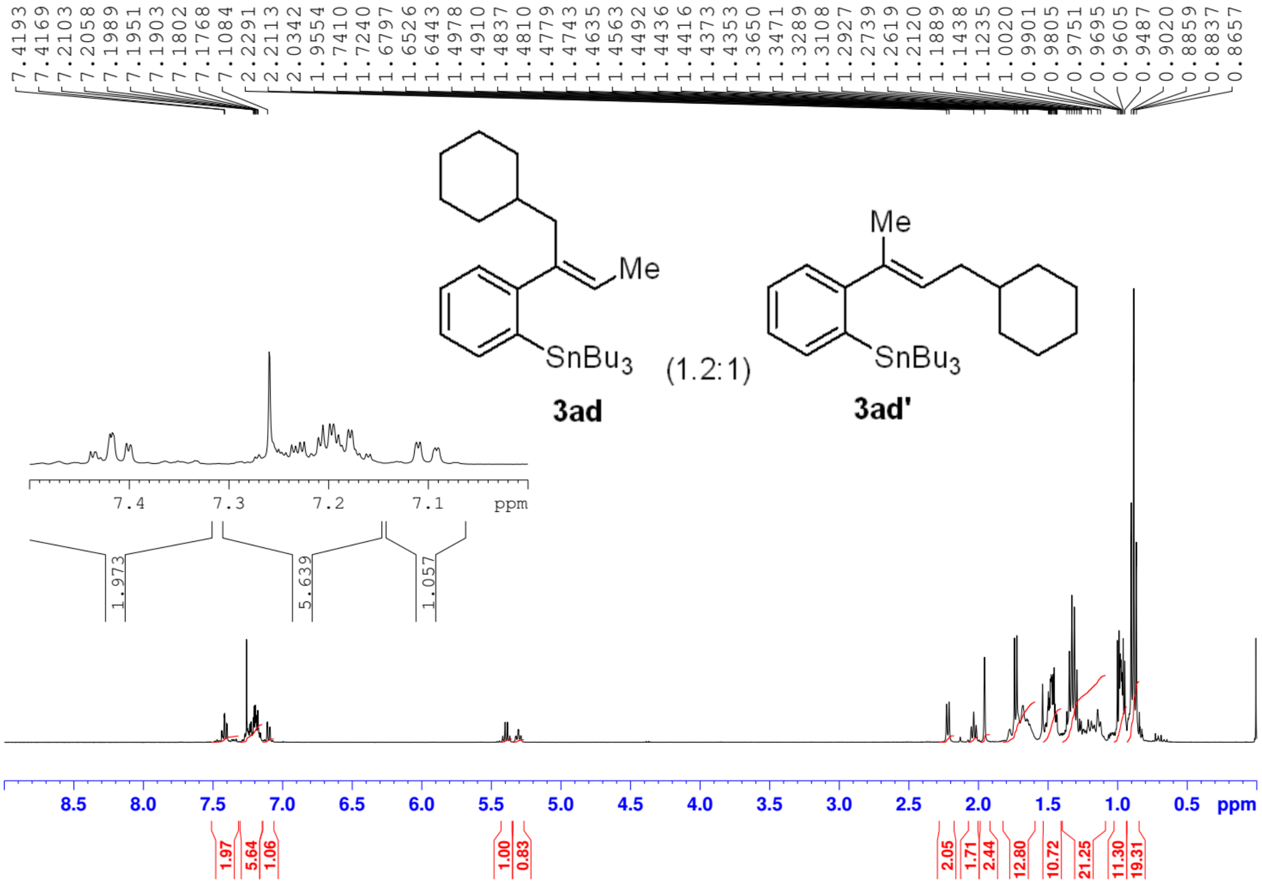
7.4193
7.4169
7.2103
7.2058
7.1989
7.1951
7.1903
7.1802
7.1768
7.1084
2.2291
2.2113
2.0342
1.9554
1.7410
1.7240
1.6797
1.6526
1.6443
1.4978
1.4910
1.4837
1.4810
1.4779
1.4743
1.4635
1.4563
1.4492
1.4436
1.4416
1.4373
1.4353
1.3650
1.3471
1.3289
1.3108
1.2927
1.2739
1.2619
1.2120
1.1889
1.1438
1.1235
1.0020
0.9901
0.9805
0.9751
0.9695
0.9605
0.9487
0.9020
0.8859
0.8837
0.8657
Me
Me
SnBu3
SnBu3
(1.2:1)
3ad
3ad'
ppm

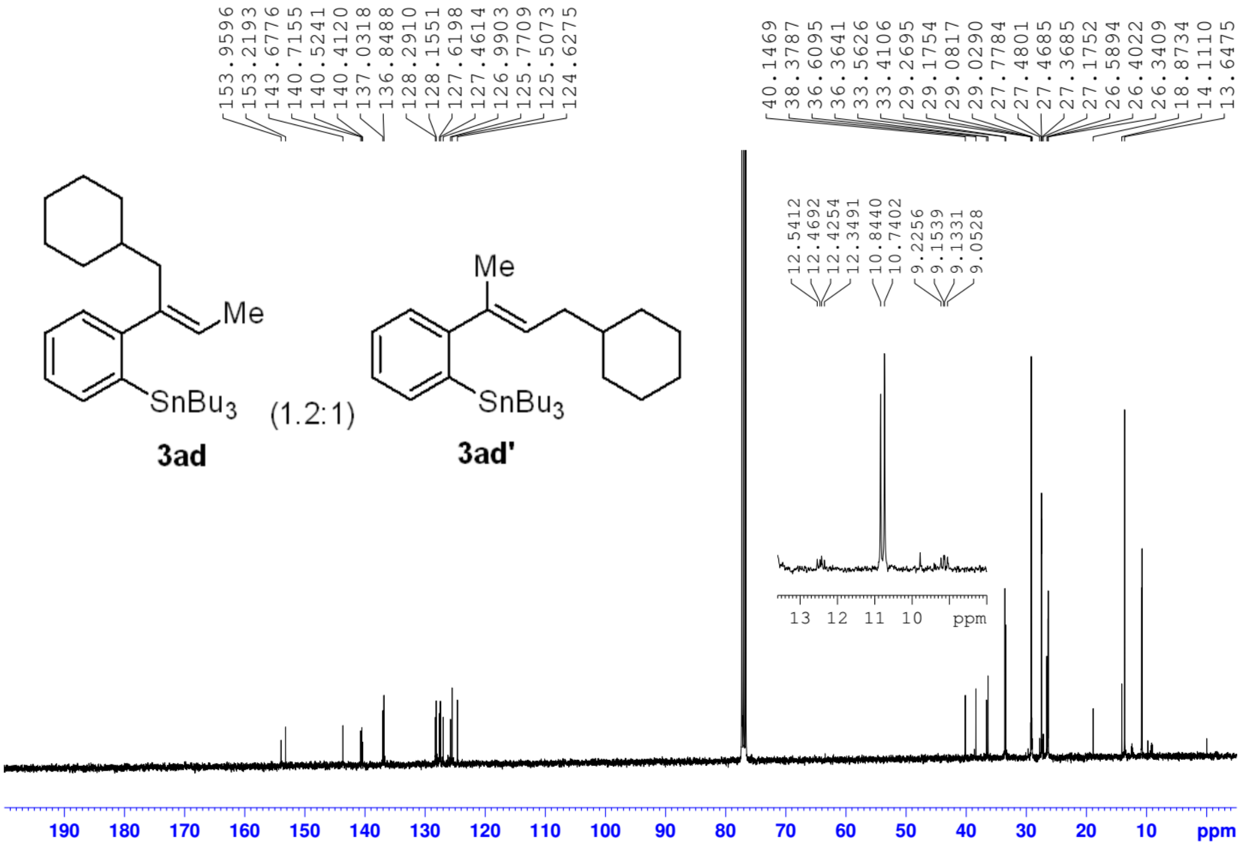
153.9596
153.2193
143.6776
140.7155
140.5241
140.4120
137.0318
136.8488
128.2910
128.1551
127.6198
127.4614
126.9903
125.7709
125.5073
124.6275
40.1469
38.3787
36.6095
36.3641
33.5626
33.4106
29.2695
29.1754
29.0817
29.0290
27.7784
27.4801
27.4685
27.3685
27.1752
26.5894
26.4022
26.3409
18.8734
14.1110
13.6475
12.5412
12.4692
12.4254
12.3491
10.8440
10.7402
9.2256
9.1539
9.1331
9.0528
Me
Me
SnBu3
SnBu3
(1.2:1)
3ad
3ad'
ppm

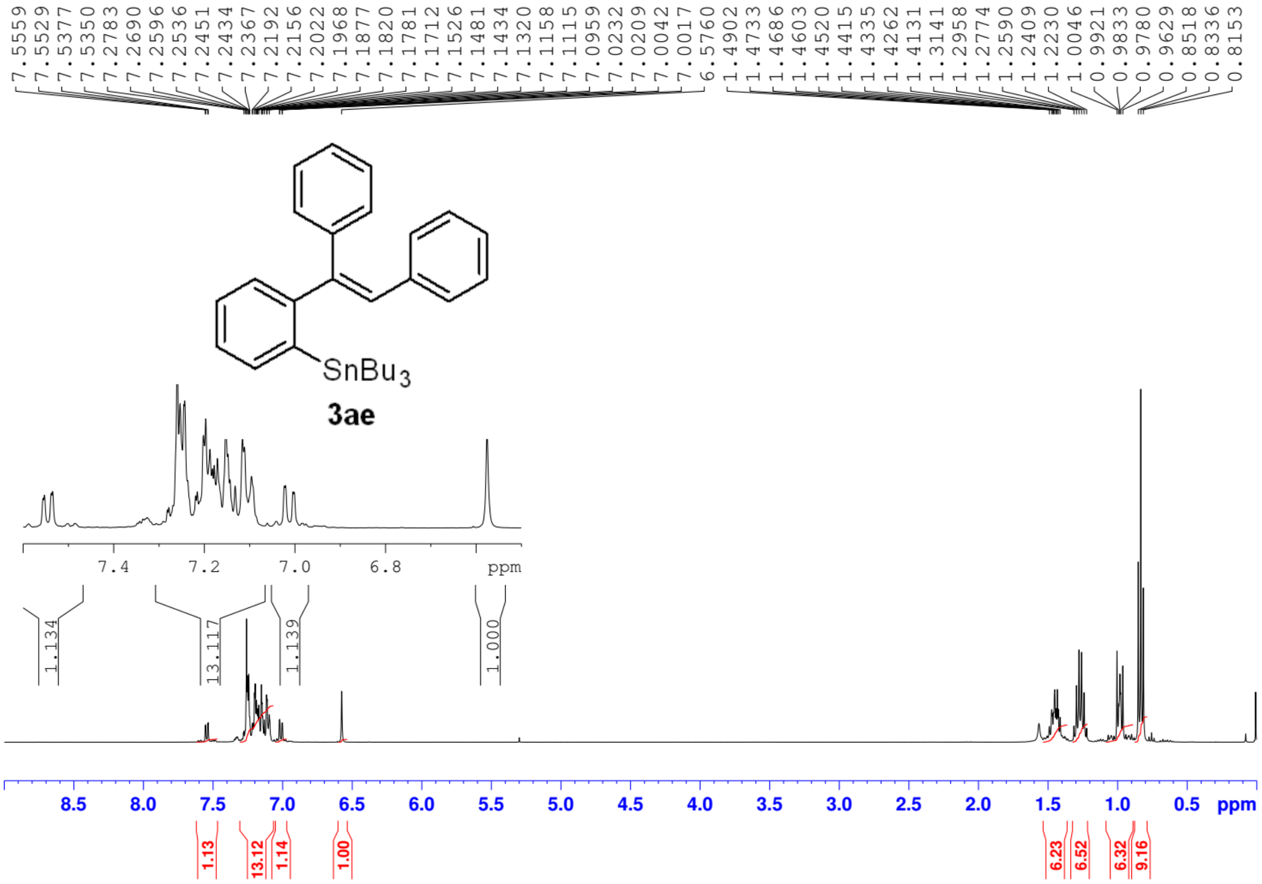
SnBu3
3ae
ppm

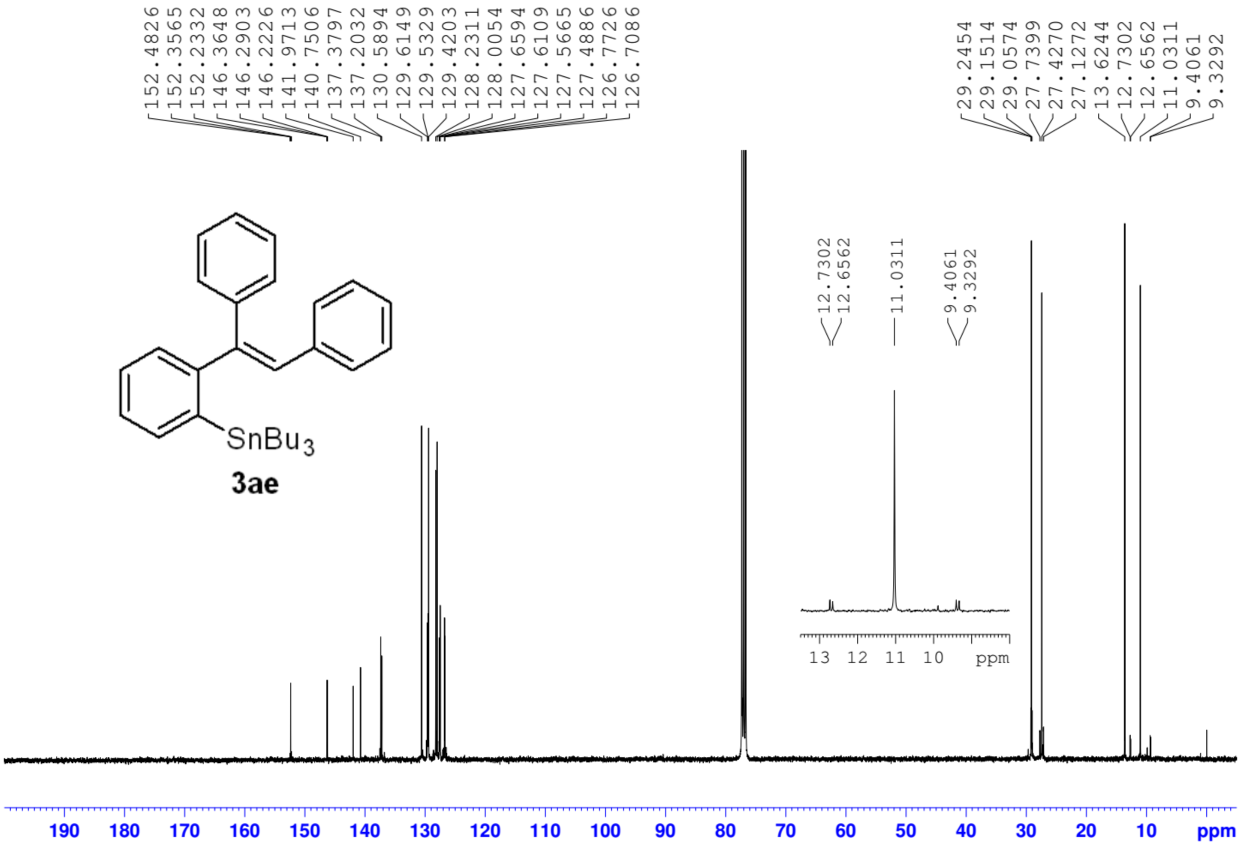
SnBu3
3ae
ppm

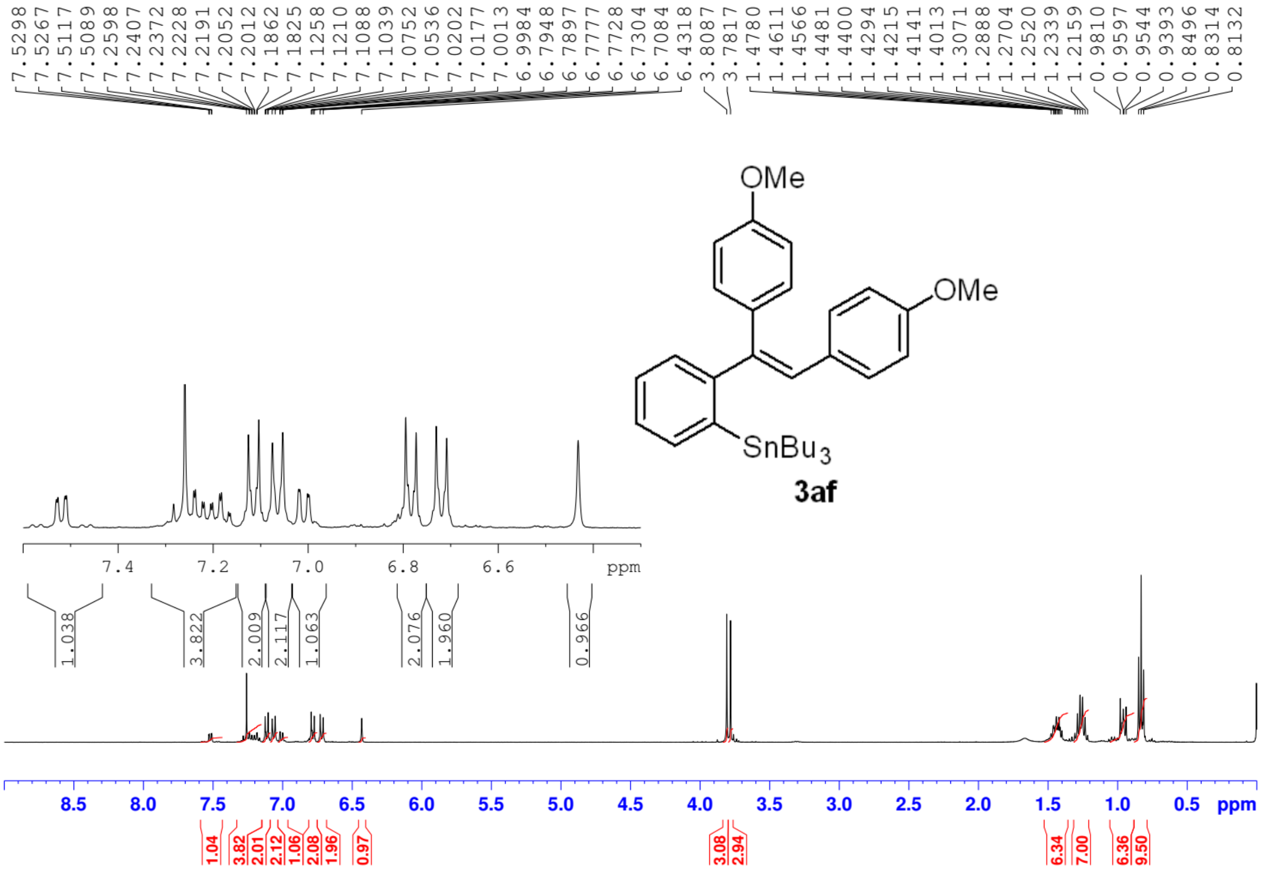
7.5298
7.5267
7.5117
7.5089
7.2598
7.2407
7.2372
7.2228
7.2191
7.2052
7.2012
7.1862
7.1825
7.1258
7.1210
7.1088
7.1039
7.0752
7.0536
7.0202
7.0177
7.0013
6.9984
6.7948
6.7897
6.7777
6.7728
6.7304
6.7084
6.4318
3.8087
3.7817
1.4780
1.4611
1.4566
1.4481
1.4400
1.4294
1.4215
1.4141
1.4013
1.3071
1.2888
1.2704
1.2520
1.2339
1.2159
0.9810
0.9597
0.9544
0.9393
0.8496
0.8314
0.8132
OMe
OMe
SnBu3
3af
7.4
7.2
7.0
6.8
6.6
ppm
1.038
3.822
2.009
2.117
1.063
2.076
1.960
0.966
8.5
8.0
7.5
7.0
6.5
6.0
5.5
5.0
4.5
4.0
3.5
3.0
2.5
2.0
1.5
1.0
0.5
ppm
1.04
3.82
2.01
2.12
1.06
2.08
1.96
0.97
3.08
2.94
6.34
7.00
6.36
9.50

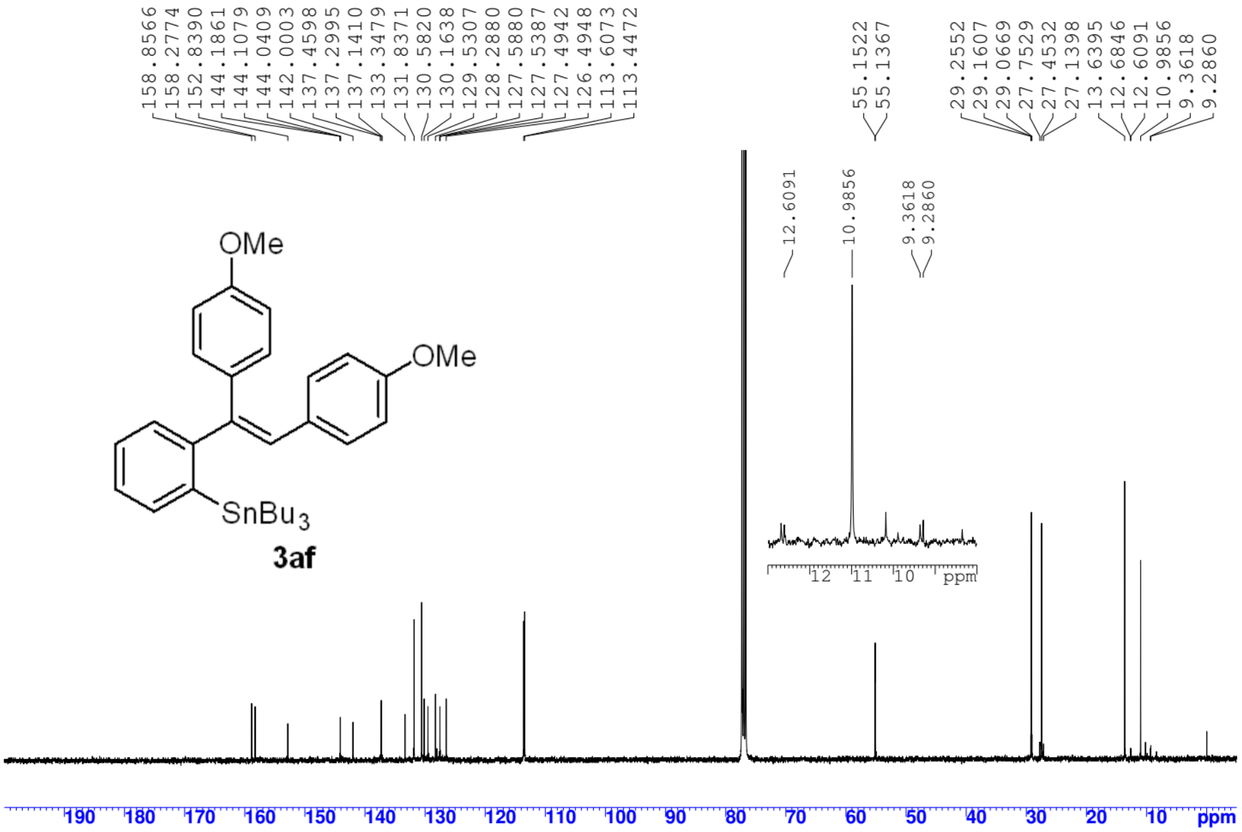
158.8566
158.2774
152.8390
144.1861
144.1079
144.0409
142.0003
137.4598
137.2995
137.1410
133.3479
131.8371
130.5820
130.1638
129.5307
128.2880
127.5880
127.5387
127.4942
126.4948
113.6073
113.4472
55.1522
55.1367
29.2552
29.1607
29.0669
27.7529
27.4532
27.1398
13.6395
12.6846
12.6091
10.9856
9.3618
9.2860
12.6091
10.9856
9.3618
9.2860
12
11
10
ppm
OMe
OMe
SnBu3
3af
190
180
170
160
150
140
130
120
110
100
90
80
70
60
50
40
30
20
10
ppm

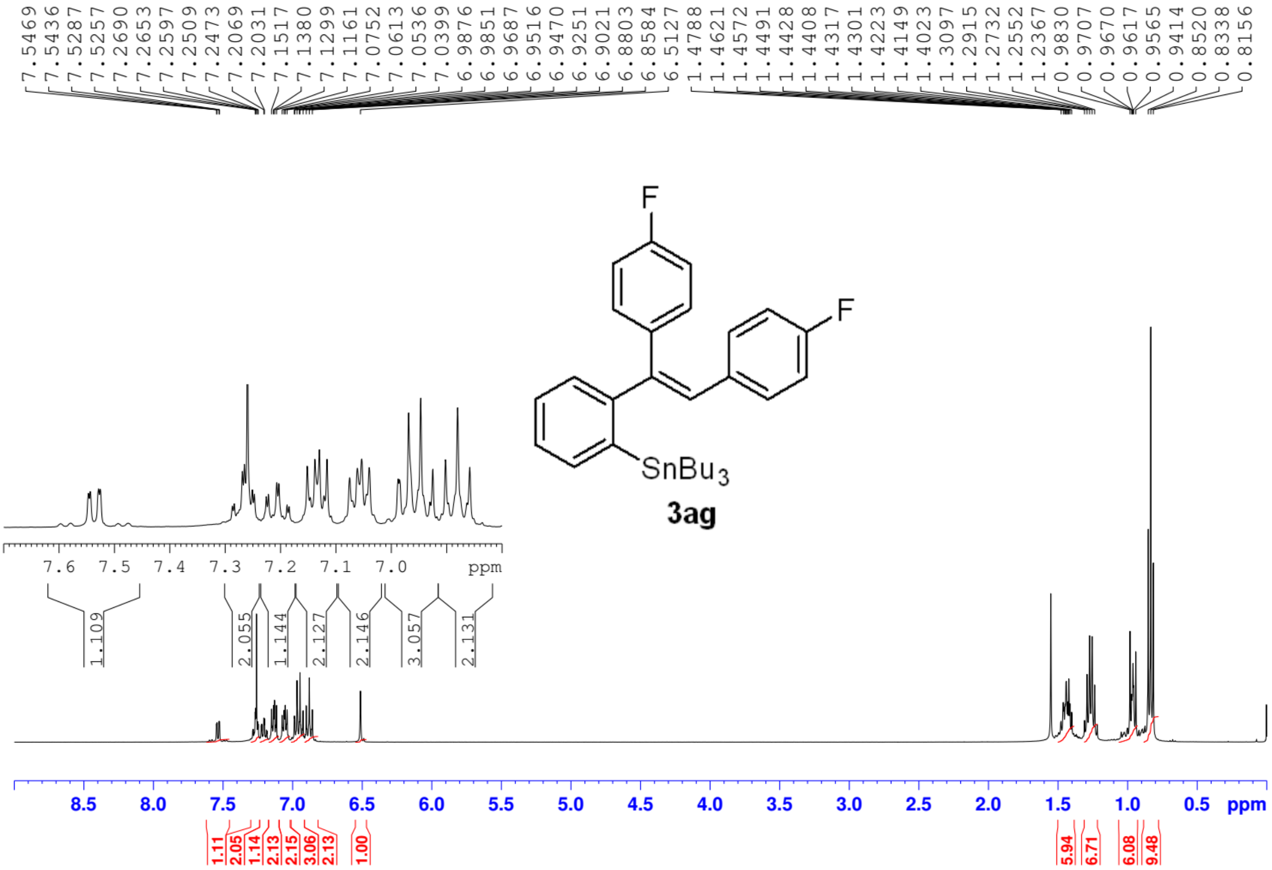
7.5469
7.5436
7.5287
7.5257
7.2690
7.2653
7.2597
7.2509
7.2473
7.2069
7.2031
7.1517
7.1380
7.1299
7.1161
7.0752
7.0613
7.0536
7.0399
6.9876
6.9851
6.9687
6.9516
6.9470
6.9251
6.9021
6.8803
6.8584
6.5127
1.4788
1.4621
1.4572
1.4491
1.4428
1.4408
1.4317
1.4301
1.4223
1.4149
1.4023
1.3097
1.2915
1.2732
1.2552
1.2367
0.9830
0.9707
0.9670
0.9617
0.9565
0.9414
0.8520
0.8338
0.8156
F
F
SnBu3
3ag
7.6 7.5 7.4 7.3 7.2 7.1 7.0 ppm
1.109
2.055
1.144
2.127
2.146
3.057
2.131
8.5 8.0 7.5 7.0 6.5 6.0 5.5 5.0 4.5 4.0 3.5 3.0 2.5 2.0 1.5 1.0 0.5 ppm
1.11
2.05
1.14
2.13
2.15
3.06
2.13
1.00
5.94
6.71
6.08
9.48

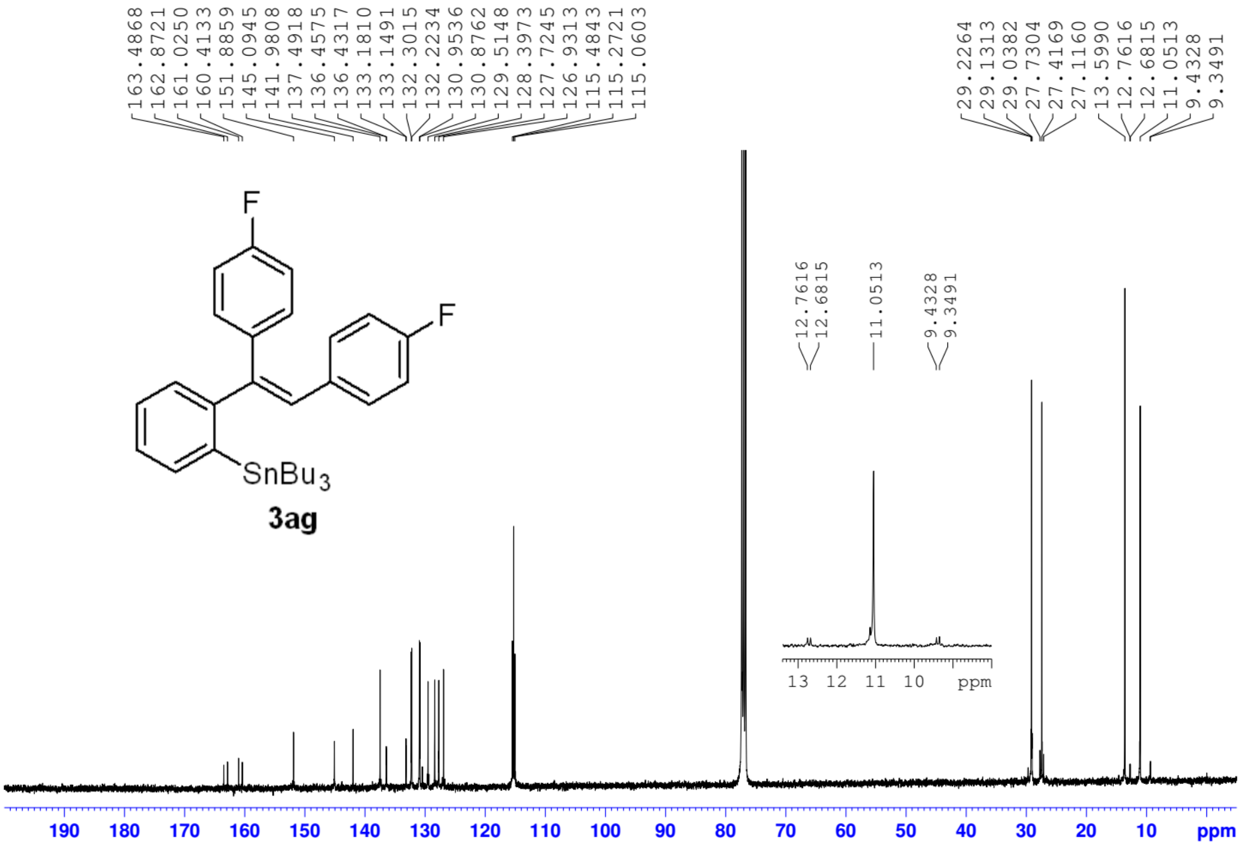
163.4868
162.8721
161.0250
160.4133
151.8859
145.0945
141.9808
137.4918
136.4575
136.4317
133.1810
133.1491
132.3015
132.2234
130.9536
130.8762
129.5148
128.3973
127.7245
126.9313
115.4843
115.2721
115.0603
29.2264
29.1313
29.0382
27.7304
27.4169
27.1160
13.5990
12.7616
12.6815
11.0513
9.4328
9.3491
F
F
SnBu3
3ag
12.7616
12.6815
11.0513
9.4328
9.3491
13 12 11 10 ppm
190 180 170 160 150 140 130 120 110 100 90 80 70 60 50 40 30 20 10 ppm

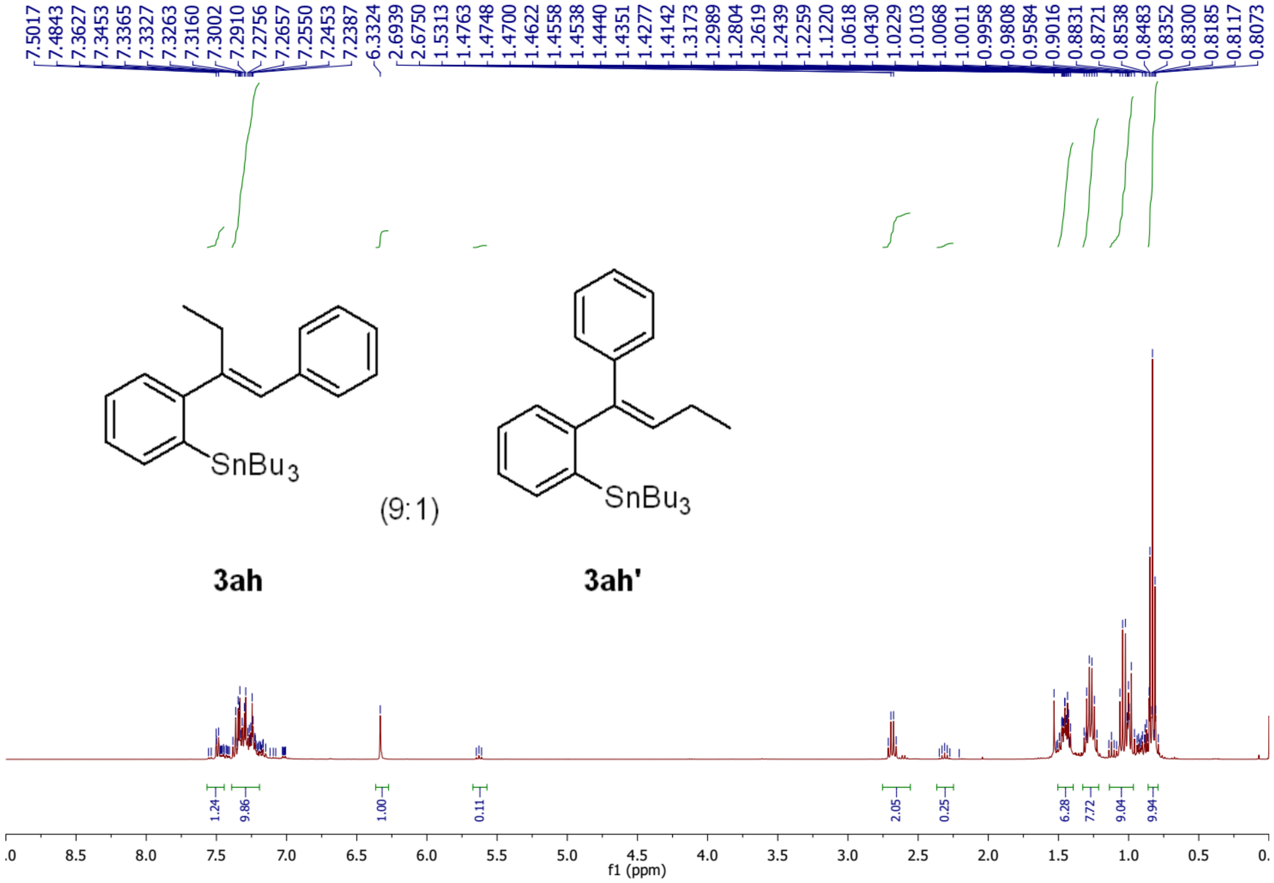
7.5017
7.4843
7.3627
7.3453
7.3365
7.3327
7.3263
7.3160
7.3002
7.2910
7.2756
7.2657
7.2550
7.2453
7.2387
6.3324
2.6939
2.6750
1.5313
1.4763
1.4748
1.4700
1.4622
1.4558
1.4538
1.4440
1.4351
1.4277
1.4142
1.3173
1.2989
1.2804
1.2619
1.2439
1.2259
1.1220
1.0618
1.0430
1.0229
1.0103
1.0068
1.0011
0.9958
0.9808
0.9584
0.9016
0.8831
0.8721
0.8538
0.8483
0.8352
0.8300
0.8185
0.8117
0.8073
SnBu3
(9:1)
SnBu3
3ah
3ah'
1.24
9.86
1.00
0.11
2.05
0.25
6.28
7.72
9.04
9.94
f1 (ppm)

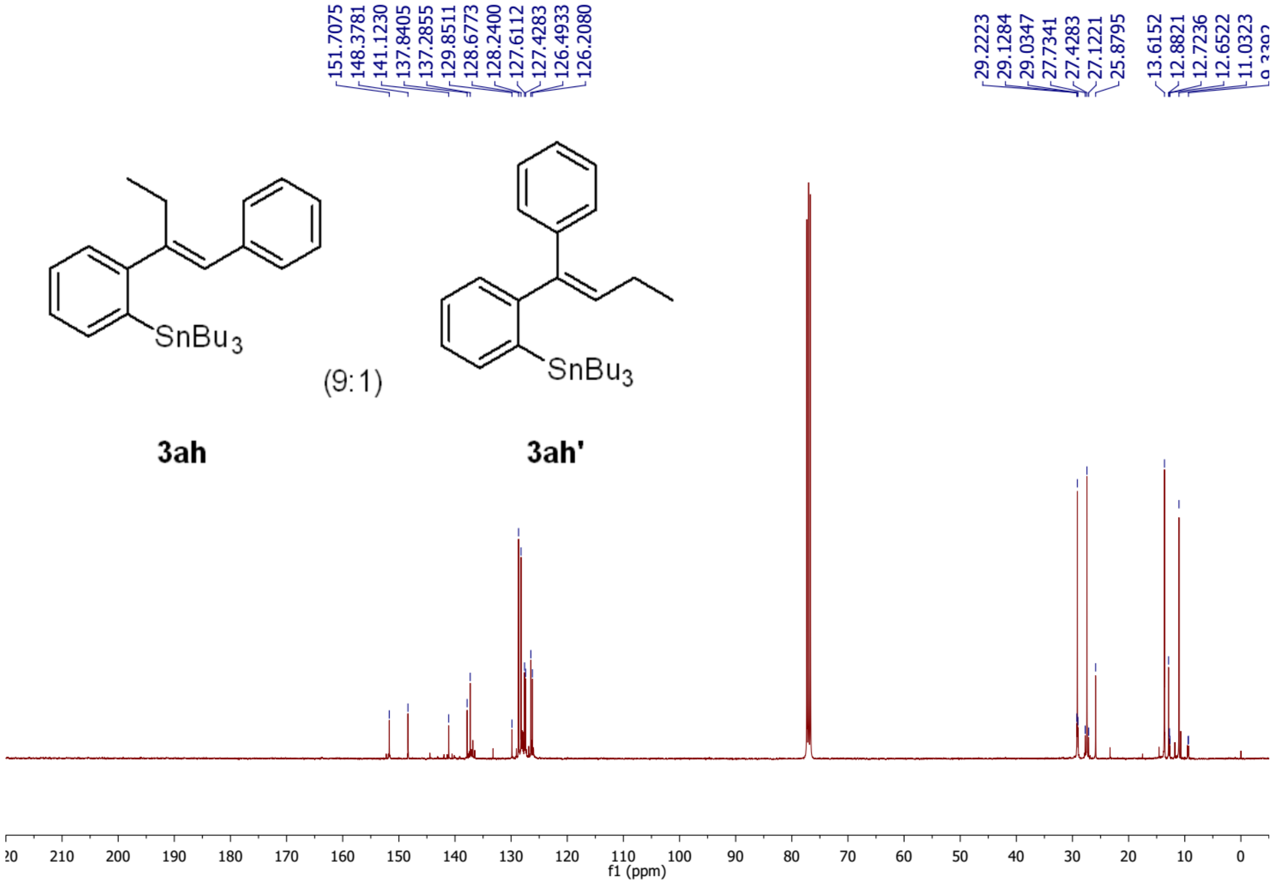
151.7075
148.3781
141.1230
137.8405
137.2855
129.8511
128.6773
128.2400
127.6112
127.4283
126.4933
126.2080
29.2223
29.1284
29.0347
27.7341
27.4283
27.1221
25.8795
13.6152
12.8821
12.7236
12.6522
11.0323
SnBu3
(9:1)
SnBu3
3ah
3ah'
f1 (ppm)

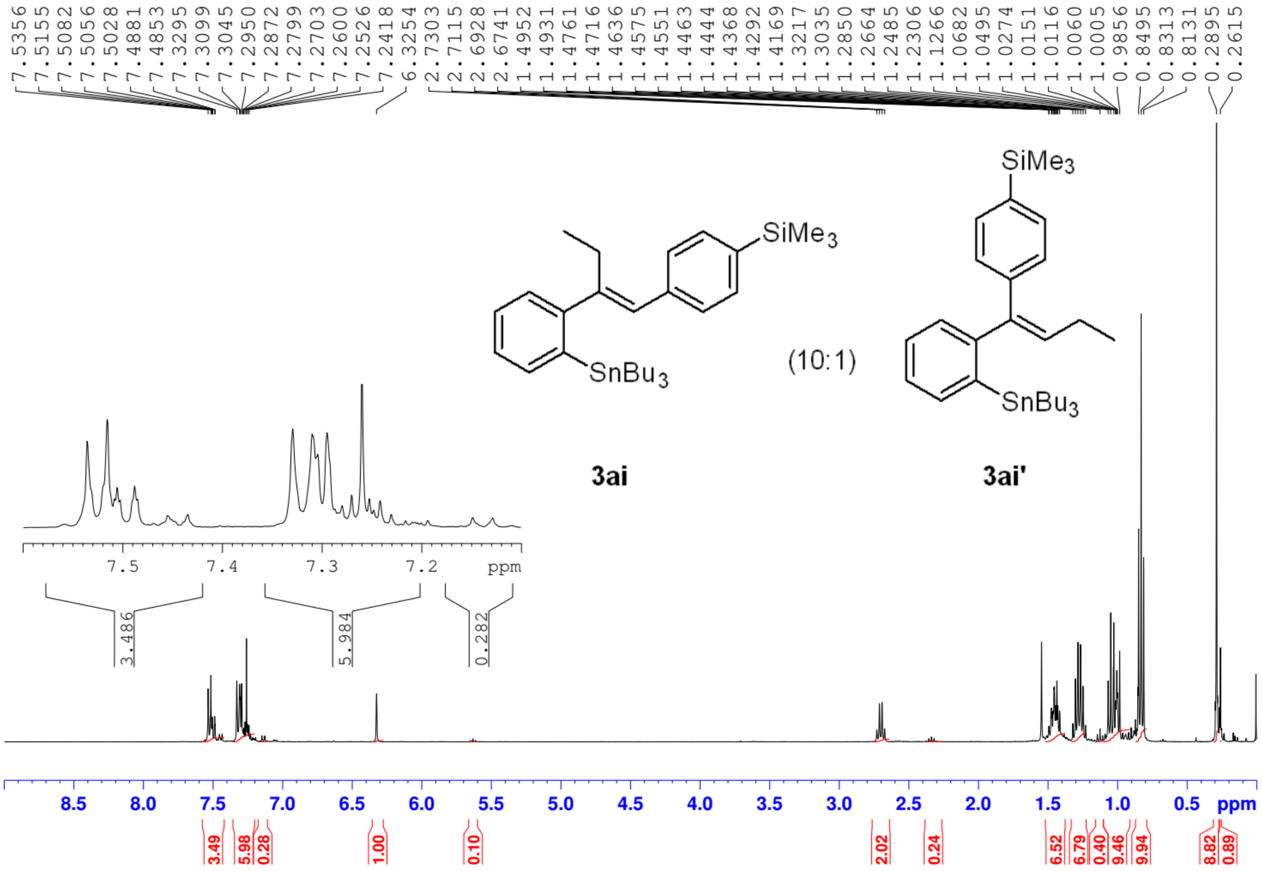
7.5356 7.5155 7.5082 7.5056 7.5028 7.4881 7.4853 7.3295 7.3099 7.3045 7.2950 7.2872 7.2799 7.2703 7.2600 7.2526 7.2418 6.3254 2.7303 2.7115 2.6928 2.6741 1.4952 1.4931 1.4761 1.4716 1.4636 1.4575 1.4551 1.4463 1.4444 1.4368 1.4292 1.4169 1.3217 1.3035 1.2850 1.2664 1.2485 1.2306 1.1266 1.0682 1.0495 1.0274 1.0151 1.0116 1.0060 1.0005 0.9856 0.8495 0.8313 0.8131 0.2895 0.2615
SiMe3
SnBu3
(10:1)
3ai
3ai'
3.486
5.984
0.282
ppm
3.49 5.98 0.28 1.00 0.10 2.02 0.24 6.52 6.79 0.40 9.46 9.94 8.82 0.89

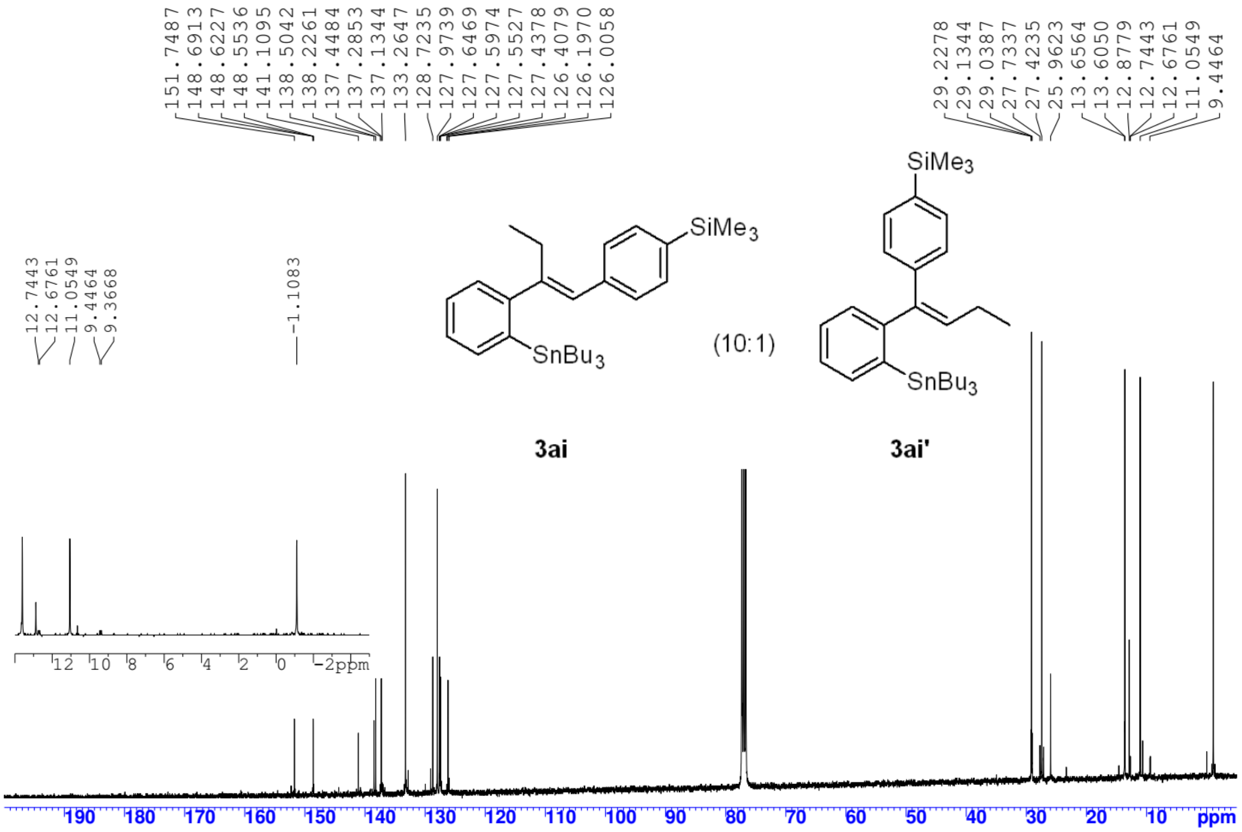
151.7487 148.6913 148.6227 148.5536 141.1095 138.5042 138.2261 137.4484 137.2853 137.1344 133.2647 128.7235 127.9739 127.6469 127.5974 127.5527 127.4378 126.4079 126.1970 126.0058
29.2278 29.1344 29.0387 27.7337 27.4235 25.9623 13.6564 13.6050 12.8779 12.7443 12.6761 11.0549 9.4464
12.7443 12.6761 11.0549 9.4464 9.3668
-1.1083
SiMe3
SnBu3
(10:1)
3ai
3ai'
ppm

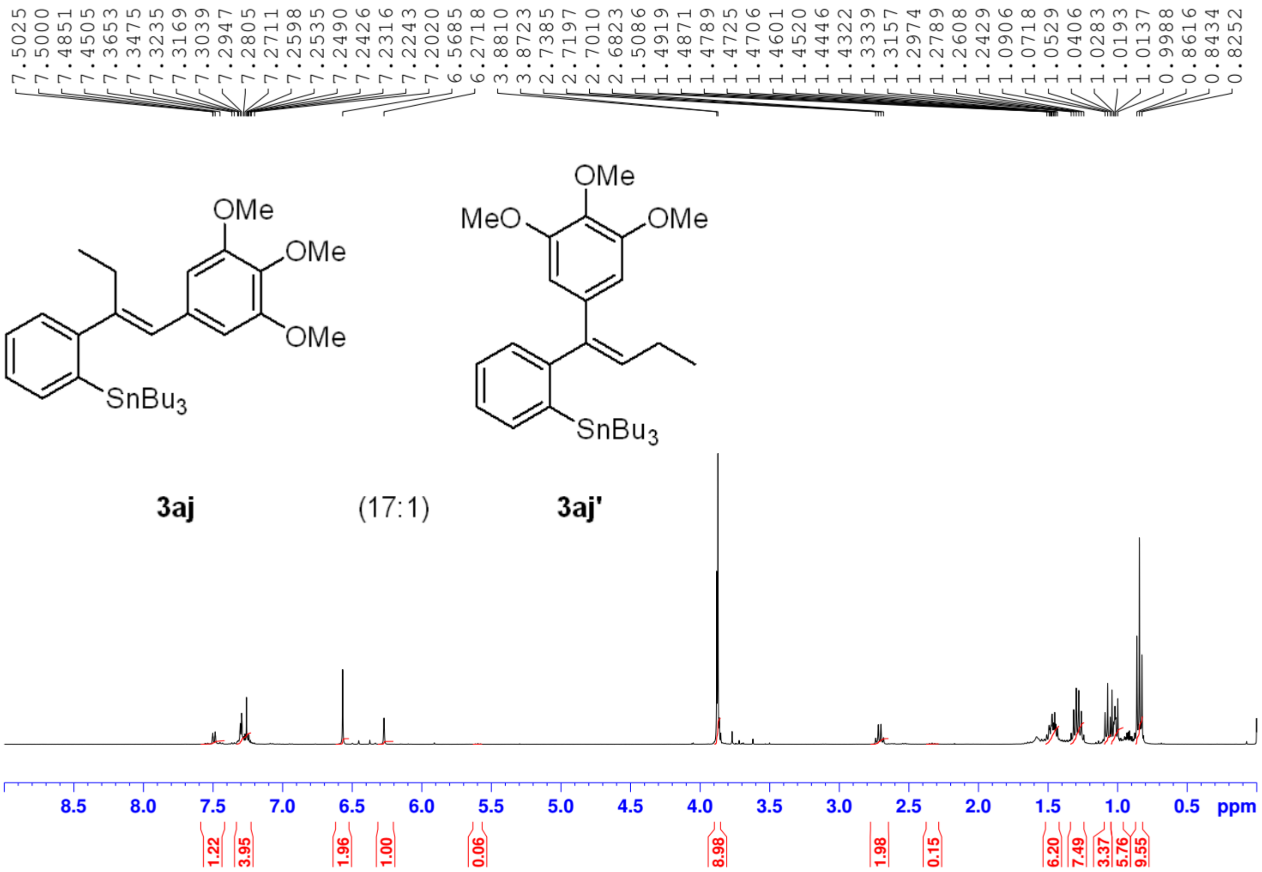
7.5025 7.5000 7.4851 7.4505 7.3653 7.3475 7.3235 7.3169 7.3039 7.2947 7.2805 7.2711 7.2598 7.2535 7.2490 7.2426 7.2316 7.2243 7.2020 6.5685 6.2718 3.8810 3.8723 2.7385 2.7197 2.7010 2.6823 1.5086 1.4919 1.4871 1.4789 1.4725 1.4706 1.4601 1.4520 1.4446 1.4322 1.3339 1.3157 1.2974 1.2789 1.2608 1.2429 1.0906 1.0718 1.0529 1.0406 1.0283 1.0193 1.0137 0.9988 0.8616 0.8434 0.8252
OMe
OMe
OMe
SnBu3
MeO
OMe
OMe
SnBu3
3aj
(17:1)
3aj'
1.22 3.95 1.96 1.00 0.06 8.98 1.98 0.15 6.20 7.49 3.37 5.76 9.55
ppm

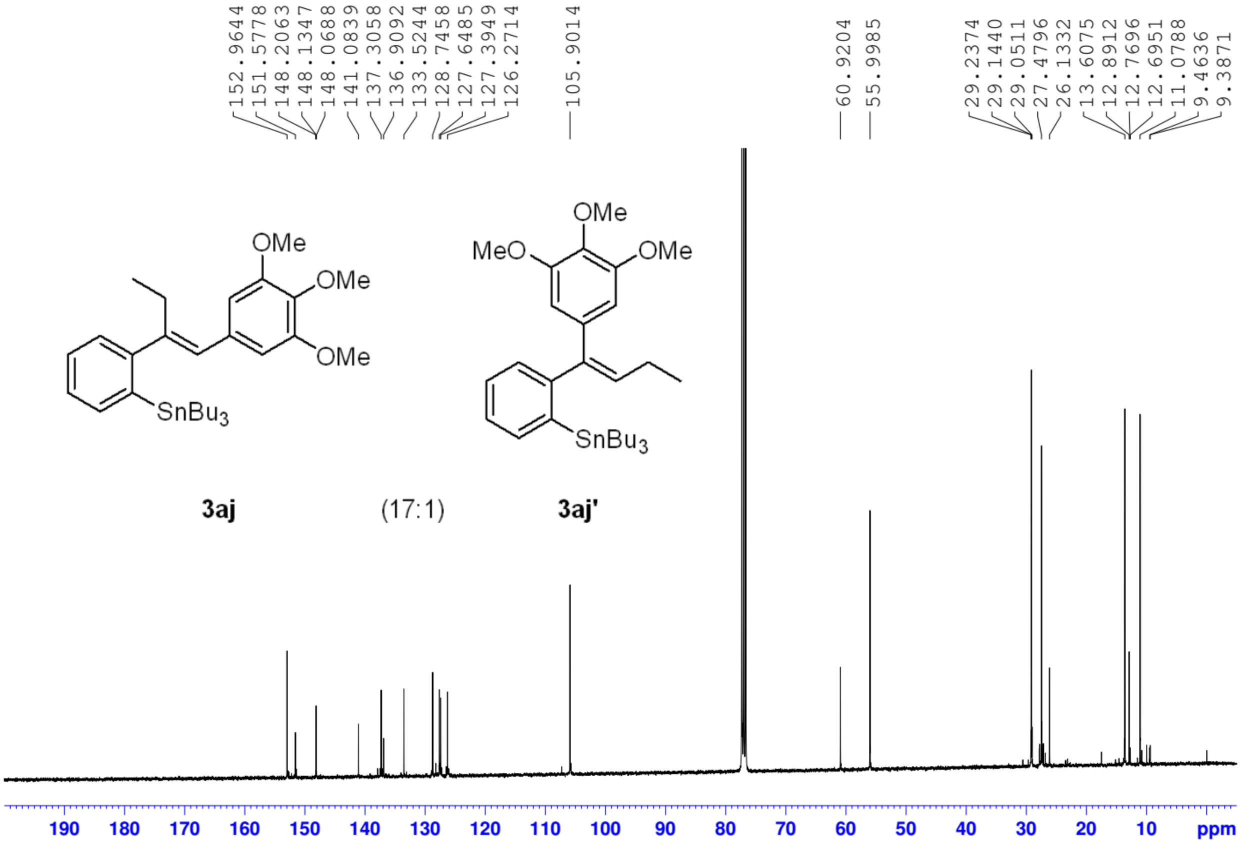
152.9644 151.5778 148.2063 148.1347 148.0688 141.0839 137.3058 136.9092 133.5244 128.7458 127.6485 127.3949 126.2714 105.9014 60.9204 55.9985 29.2374 29.1440 29.0511 27.4796 26.1332 13.6075 12.8912 12.7696 12.6951 11.0788 9.4636 9.3871
OMe
OMe
OMe
SnBu3
MeO
OMe
OMe
SnBu3
3aj
(17:1)
3aj'
ppm

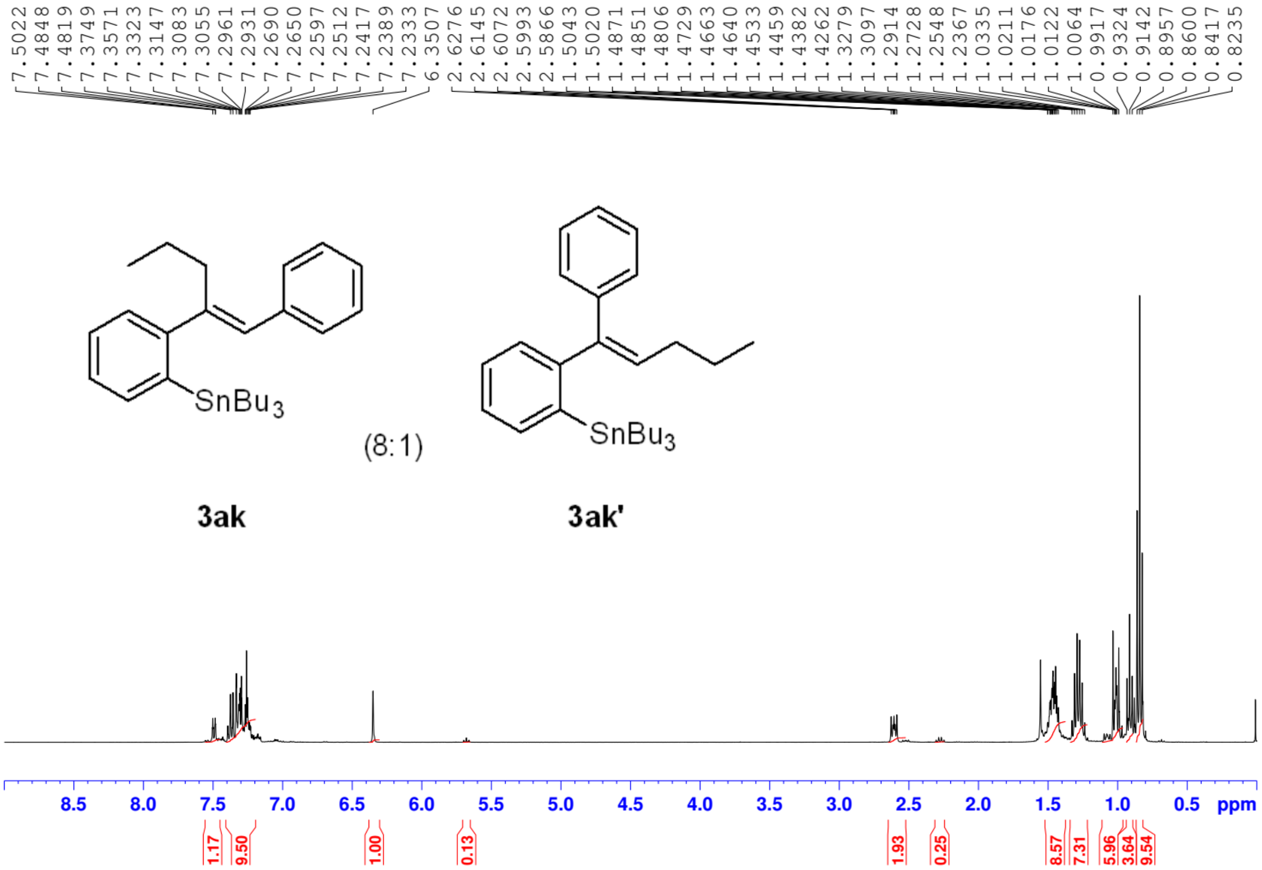
7.5022
7.4848
7.4819
7.3749
7.3571
7.3323
7.3147
7.3083
7.3055
7.2961
7.2931
7.2690
7.2650
7.2597
7.2512
7.2417
7.2389
7.2333
6.3507
2.6276
2.6145
2.6072
2.5993
2.5866
1.5043
1.5020
1.4871
1.4851
1.4806
1.4729
1.4663
1.4640
1.4533
1.4459
1.4382
1.4262
1.3279
1.3097
1.2914
1.2728
1.2548
1.2367
1.0335
1.0211
1.0176
1.0122
1.0064
0.9917
0.9324
0.9142
0.8957
0.8600
0.8417
0.8235
SnBu3
(8:1)
SnBu3
3ak
3ak'
8.5 8.0 7.5 7.0 6.5 6.0 5.5 5.0 4.5 4.0 3.5 3.0 2.5 2.0 1.5 1.0 0.5 ppm
1.17
9.50
1.00
0.13
1.93
0.25
8.57
7.31
5.96
3.64
9.54

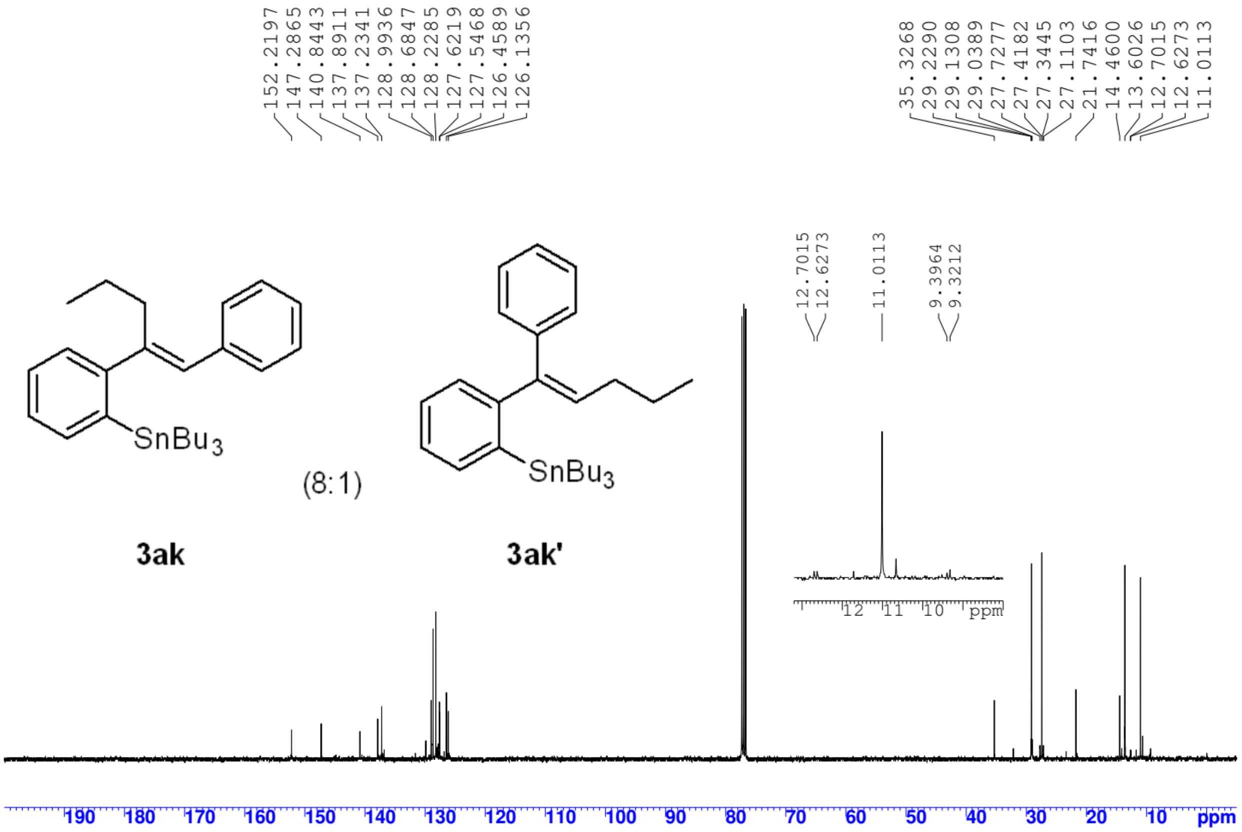
152.2197
147.2865
140.8443
137.8911
137.2341
128.9936
128.6847
128.2285
127.6219
127.5468
126.4589
126.1356
35.3268
29.2290
29.1308
29.0389
27.7277
27.4182
27.3445
27.1103
21.7416
14.4600
13.6026
12.7015
12.6273
11.0113
12.7015
12.6273
11.0113
9.3964
9.3212
SnBu3
(8:1)
SnBu3
3ak
3ak'
12 11 10 ppm
190 180 170 160 150 140 130 120 110 100 90 80 70 60 50 40 30 20 10 ppm

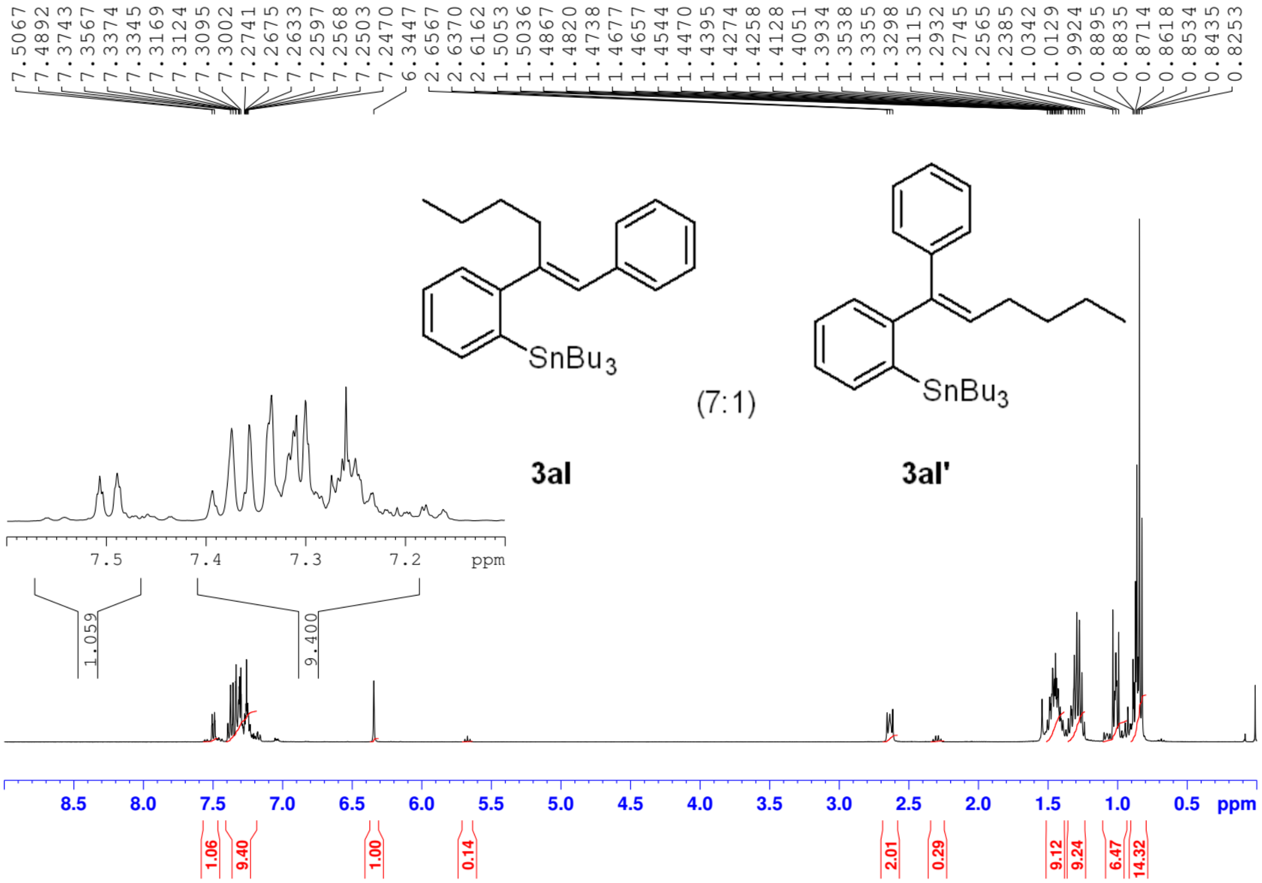
7.5067 7.4892 7.3743 7.3567 7.3374 7.3345 7.3169 7.3124 7.3095 7.3002 7.2741 7.2675 7.2633 7.2597 7.2568 7.2503 7.2470
6.3447
2.6567 2.6370 2.6162 1.5053 1.5036 1.4867 1.4820 1.4738 1.4677 1.4657 1.4544 1.4470 1.4395 1.4274 1.4258 1.4128 1.4051 1.3934 1.3538 1.3355 1.3298 1.3115 1.2932 1.2745 1.2565 1.2385 1.0342 1.0129 0.9924 0.8895 0.8835 0.8714 0.8618 0.8534 0.8435 0.8253
SnBu3
(7:1)
3al
3al'
1.059
9.400
ppm
1.06
9.40
1.00
0.14
2.01
0.29
9.12
9.24
6.47
14.32

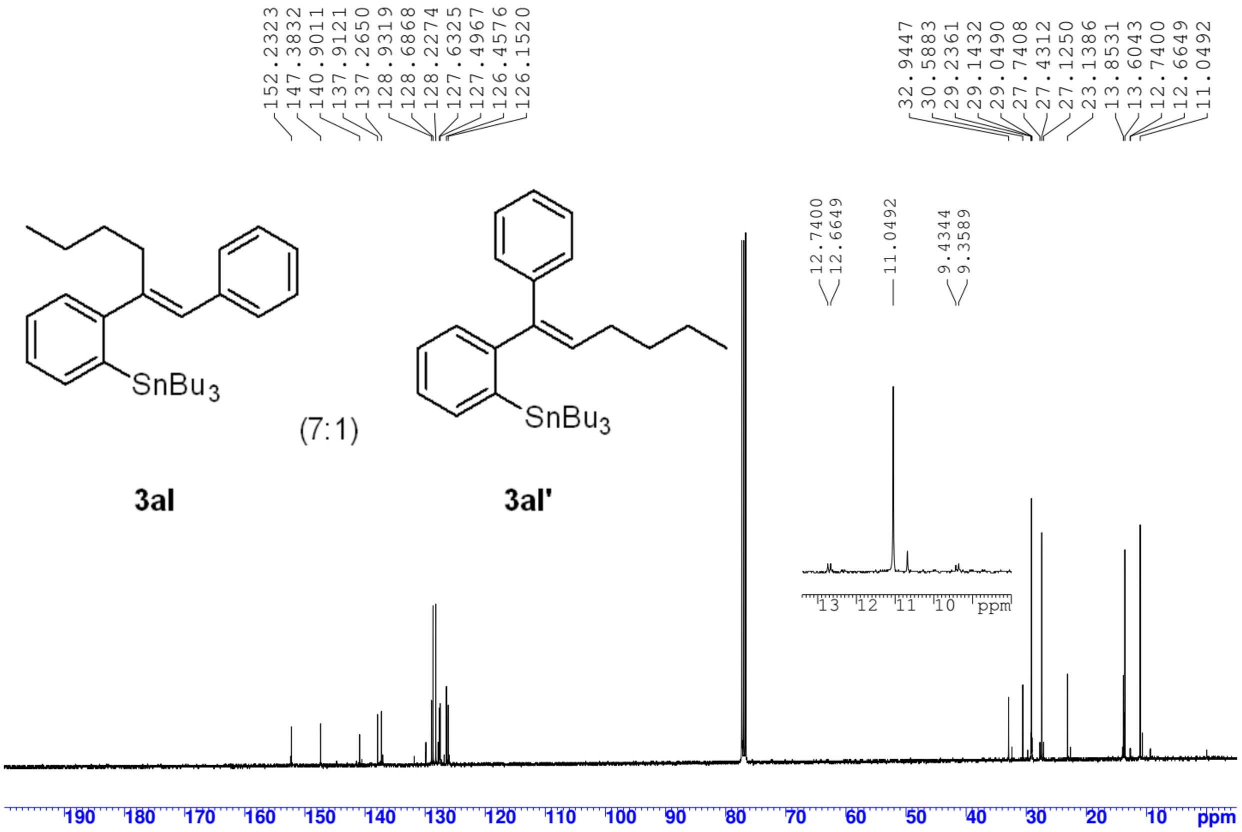
152.2323 147.3832 140.9011 137.9121 137.2650 128.9319 128.6868 128.2274 127.6325 127.4967 126.4576 126.1520
32.9447 30.5883 29.2361 29.1432 29.0490 27.7408 27.4312 27.1250 23.1386 13.8531 13.6043 12.7400 12.6649 11.0492
12.7400 12.6649
11.0492
9.4344 9.3589
SnBu3
(7:1)
3al
3al'
ppm

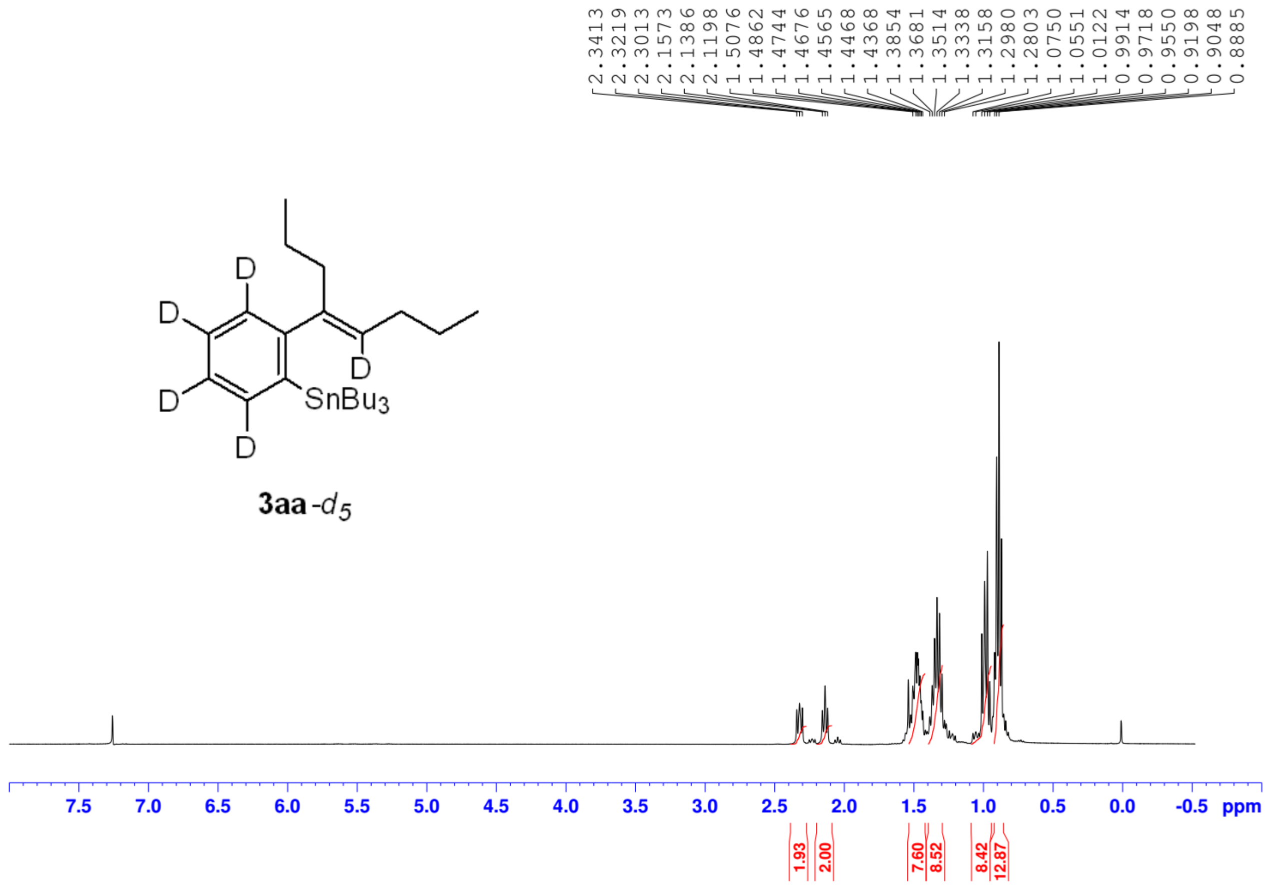
2.3413
2.3219
2.3013
2.1573
2.1386
2.1198
1.5076
1.4862
1.4744
1.4676
1.4565
1.4468
1.4368
1.3854
1.3681
1.3514
1.3338
1.3158
1.2980
1.2803
1.0750
1.0551
1.0122
0.9914
0.9718
0.9550
0.9198
0.9048
0.8885
D
D
D
D
D
SnBu3
3aa-d5
7.5 7.0 6.5 6.0 5.5 5.0 4.5 4.0 3.5 3.0 2.5 2.0 1.5 1.0 0.5 0.0 -0.5 ppm
1.93
2.00
7.60
8.52
8.42
12.87

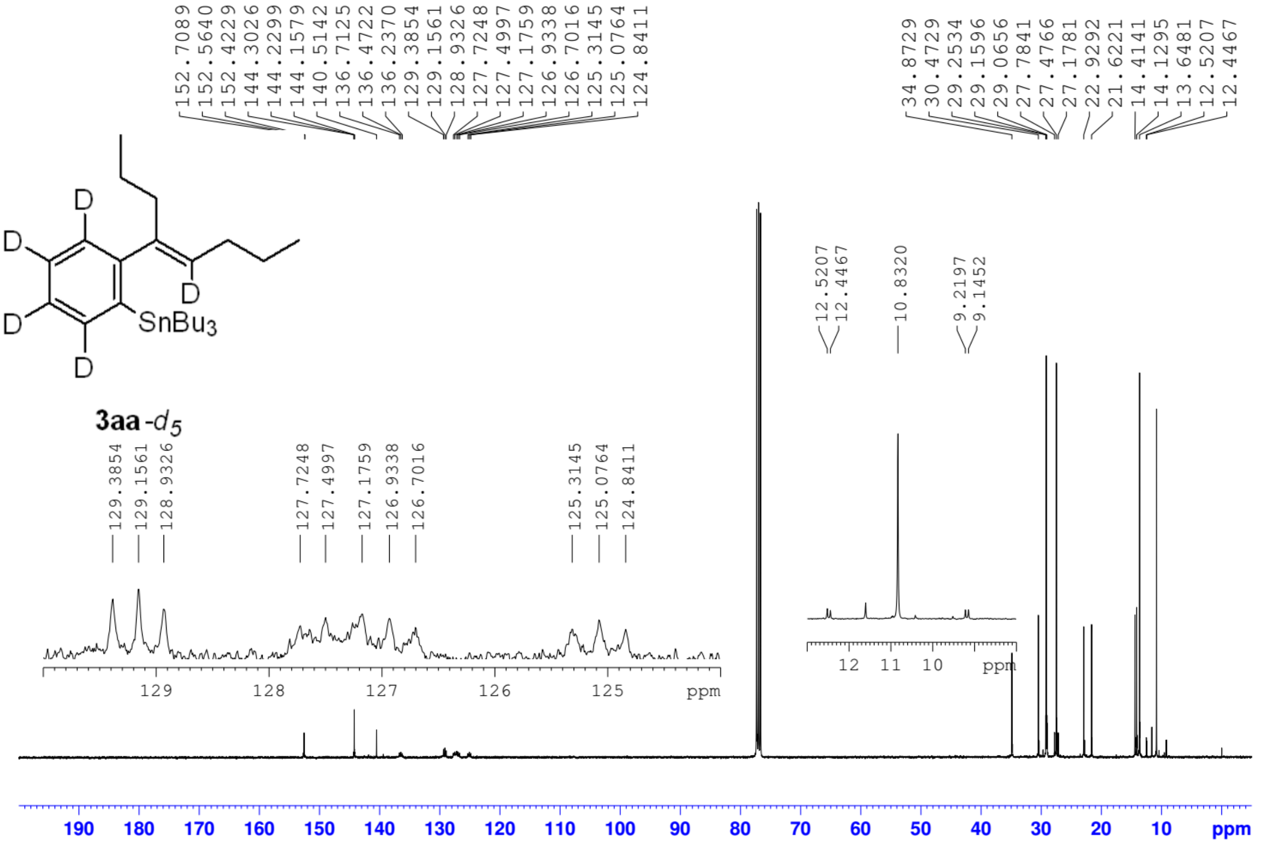
152.7089
152.5640
152.4229
144.3026
144.2299
144.1579
140.5142
136.7125
136.4722
136.2370
129.3854
129.1561
128.9326
127.7248
127.4997
127.1759
126.9338
126.7016
125.3145
125.0764
124.8411
34.8729
30.4729
29.2534
29.1596
29.0656
27.7841
27.4766
27.1781
22.9292
21.6221
14.4141
14.1295
13.6481
12.5207
12.4467
D
D
D
D
D
SnBu3
3aa-d5
129.3854
129.1561
128.9326
127.7248
127.4997
127.1759
126.9338
126.7016
125.3145
125.0764
124.8411
129 128 127 126 125 ppm
12.5207
12.4467
10.8320
9.2197
9.1452
12 11 10 ppm
190 180 170 160 150 140 130 120 110 100 90 80 70 60 50 40 30 20 10 ppm

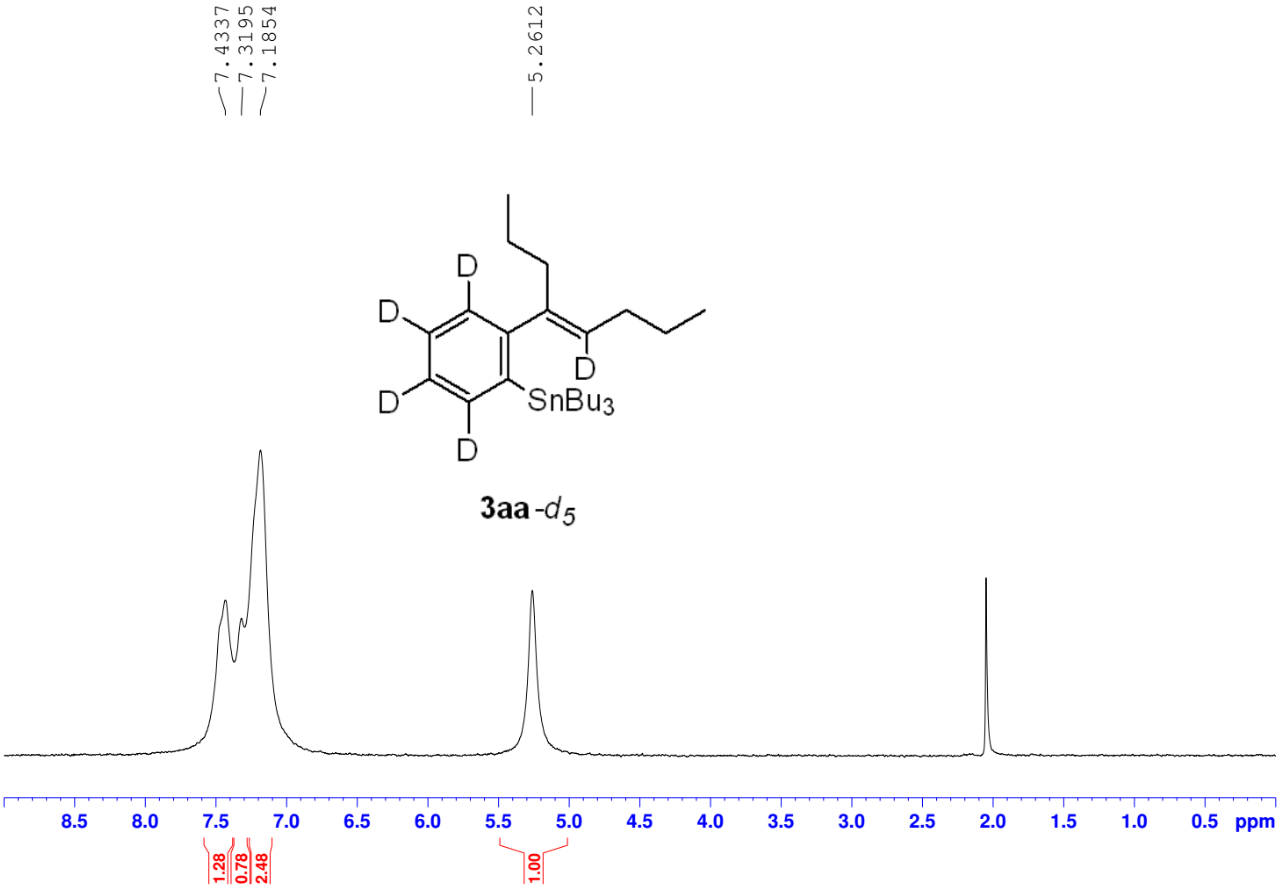
7.4337
7.3195
7.1854
5.2612
D
D
D
D
D
SnBu3
3aa-d5
8.5
8.0
7.5
7.0
6.5
6.0
5.5
5.0
4.5
4.0
3.5
3.0
2.5
2.0
1.5
1.0
0.5
ppm
1.28
0.78
2.48
1.00

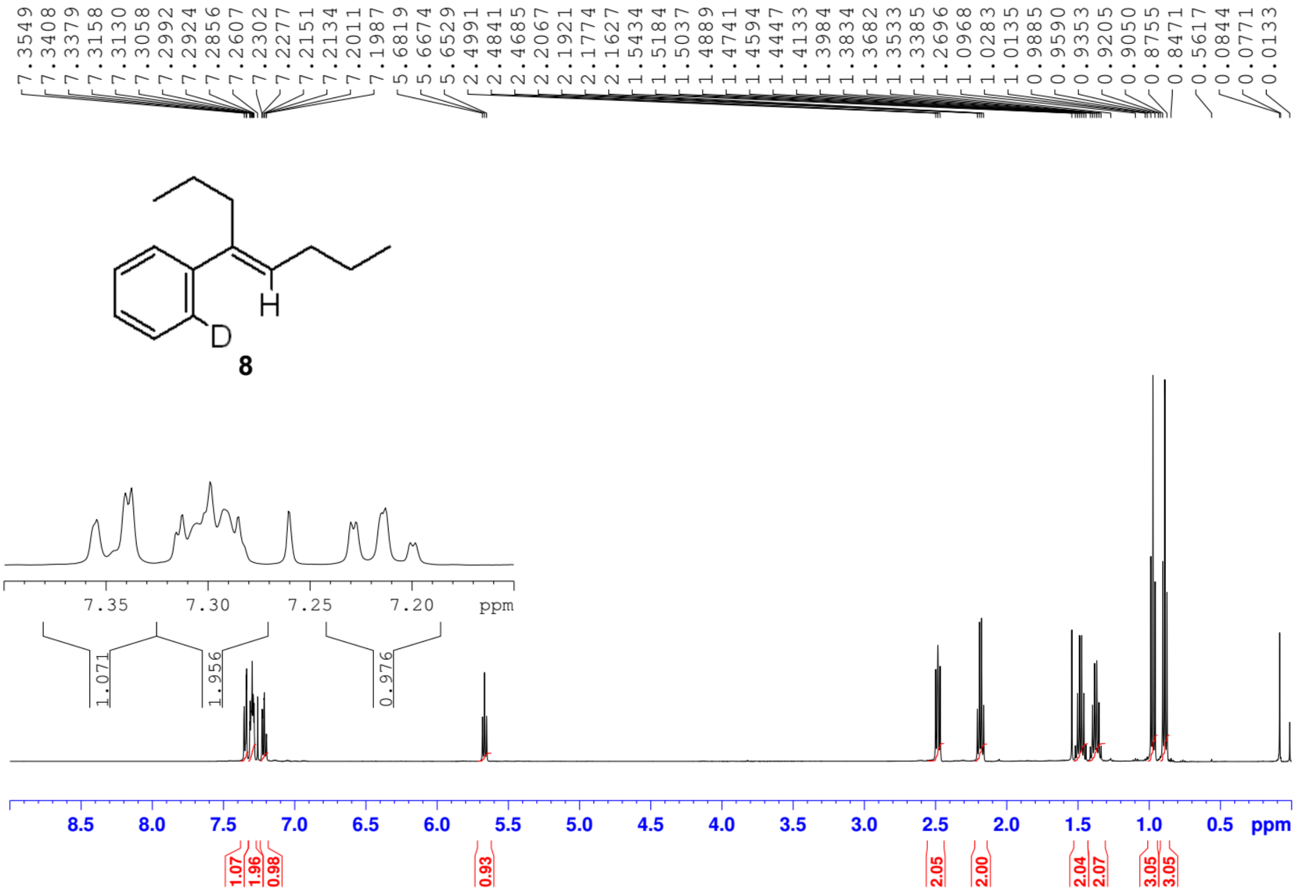
7.3549
7.3408
7.3379
7.3158
7.3130
7.3058
7.2992
7.2924
7.2856
7.2607
7.2302
7.2277
7.2151
7.2134
7.2011
7.1987
5.6819
5.6674
5.6529
2.4991
2.4841
2.4685
2.2067
2.1921
2.1774
2.1627
1.5434
1.5184
1.5037
1.4889
1.4741
1.4594
1.4447
1.4133
1.3984
1.3834
1.3682
1.3533
1.3385
1.2696
1.0968
1.0283
1.0135
0.9885
0.9590
0.9353
0.9205
0.9050
0.8755
0.8471
0.5617
0.0844
0.0771
0.0133
D
H
8
7.35
7.30
7.25
7.20
ppm
1.071
1.956
0.976
8.5
8.0
7.5
7.0
6.5
6.0
5.5
5.0
4.5
4.0
3.5
3.0
2.5
2.0
1.5
1.0
0.5
ppm
1.07
1.96
0.98
0.93
2.05
2.00
2.04
2.07
3.05
3.05

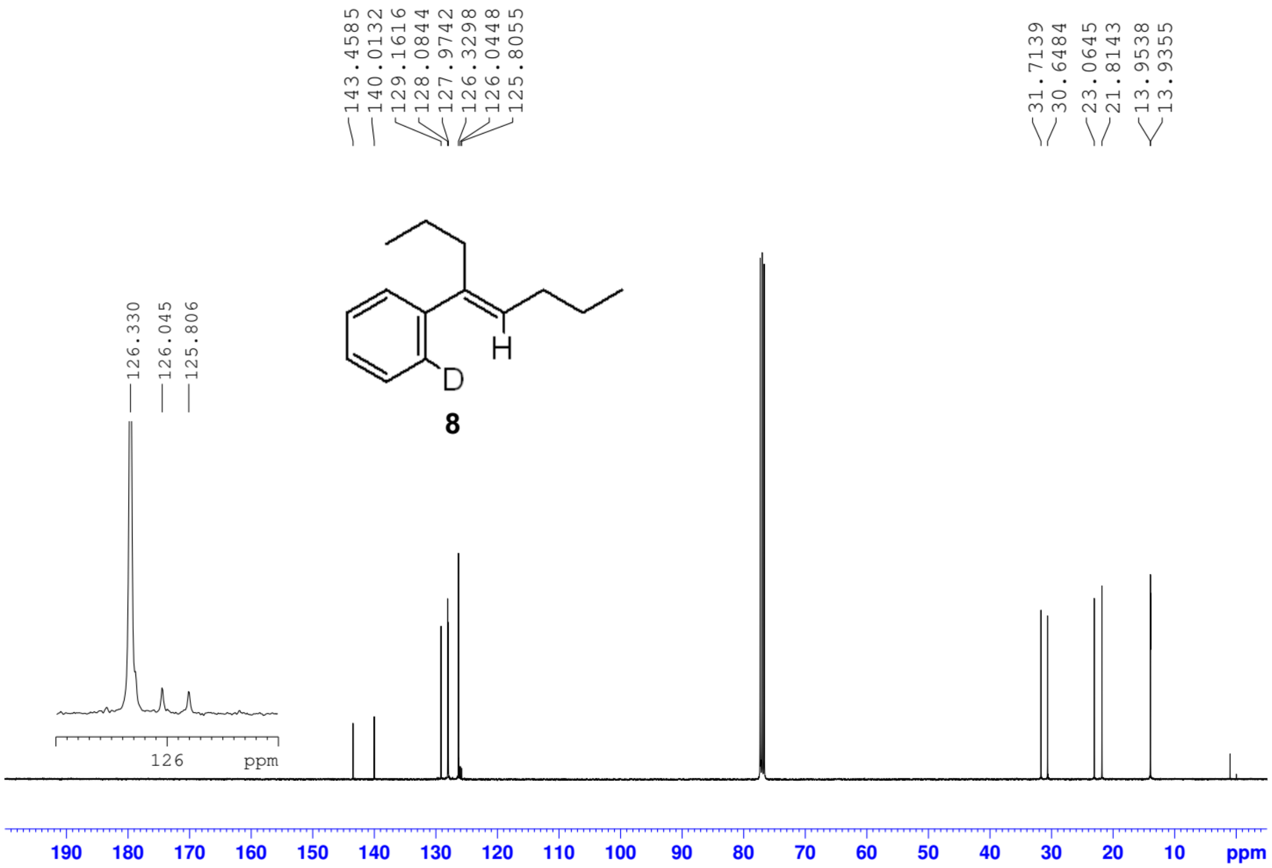
143.4585
140.0132
129.1616
128.0844
127.9742
126.3298
126.0448
125.8055
31.7139
30.6484
23.0645
21.8143
13.9538
13.9355
126.330
126.045
125.806
126
ppm
D
H
8
190
180
170
160
150
140
130
120
110
100
90
80
70
60
50
40
30
20
10
ppm

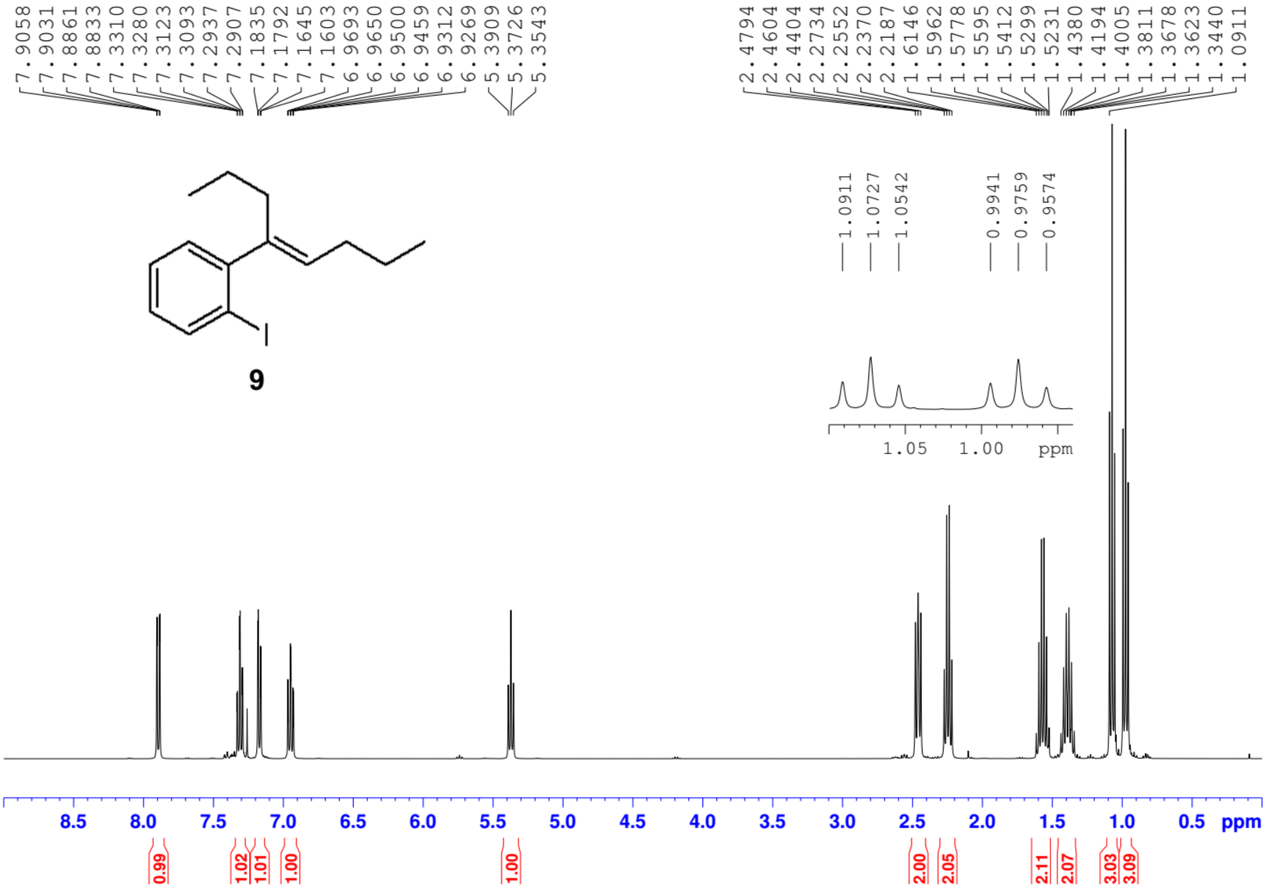
7.9058
7.9031
7.8861
7.8833
7.3310
7.3280
7.3123
7.3093
7.2937
7.2907
7.1835
7.1792
7.1645
7.1603
6.9693
6.9650
6.9500
6.9459
6.9312
6.9269
5.3909
5.3726
5.3543
2.4794
2.4604
2.4404
2.2734
2.2552
2.2370
2.2187
1.6146
1.5962
1.5778
1.5595
1.5412
1.5299
1.5231
1.4380
1.4194
1.4005
1.3811
1.3678
1.3623
1.3440
1.0911
1.0911
1.0727
1.0542
0.9941
0.9759
0.9574
1.05
1.00
ppm
I
9
8.5
8.0
7.5
7.0
6.5
6.0
5.5
5.0
4.5
4.0
3.5
3.0
2.5
2.0
1.5
1.0
0.5
ppm
0.99
1.02
1.01
1.00
1.00
2.00
2.05
2.11
2.07
3.03
3.09

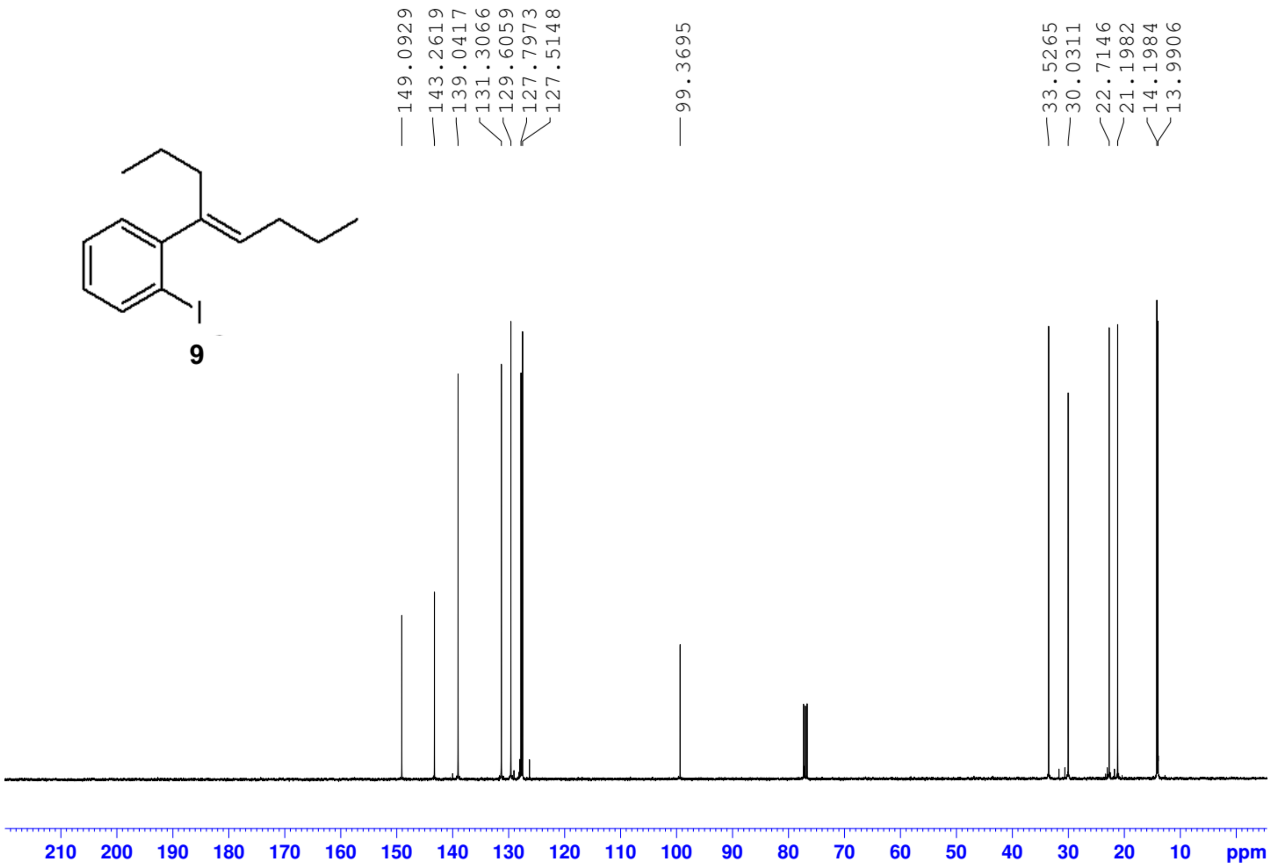
149.0929
143.2619
139.0417
131.3066
129.6059
127.7973
127.5148
99.3695
33.5265
30.0311
22.7146
21.1982
14.1984
13.9906
I
9
210
200
190
180
170
160
150
140
130
120
110
100
90
80
70
60
50
40
30
20
10
ppm

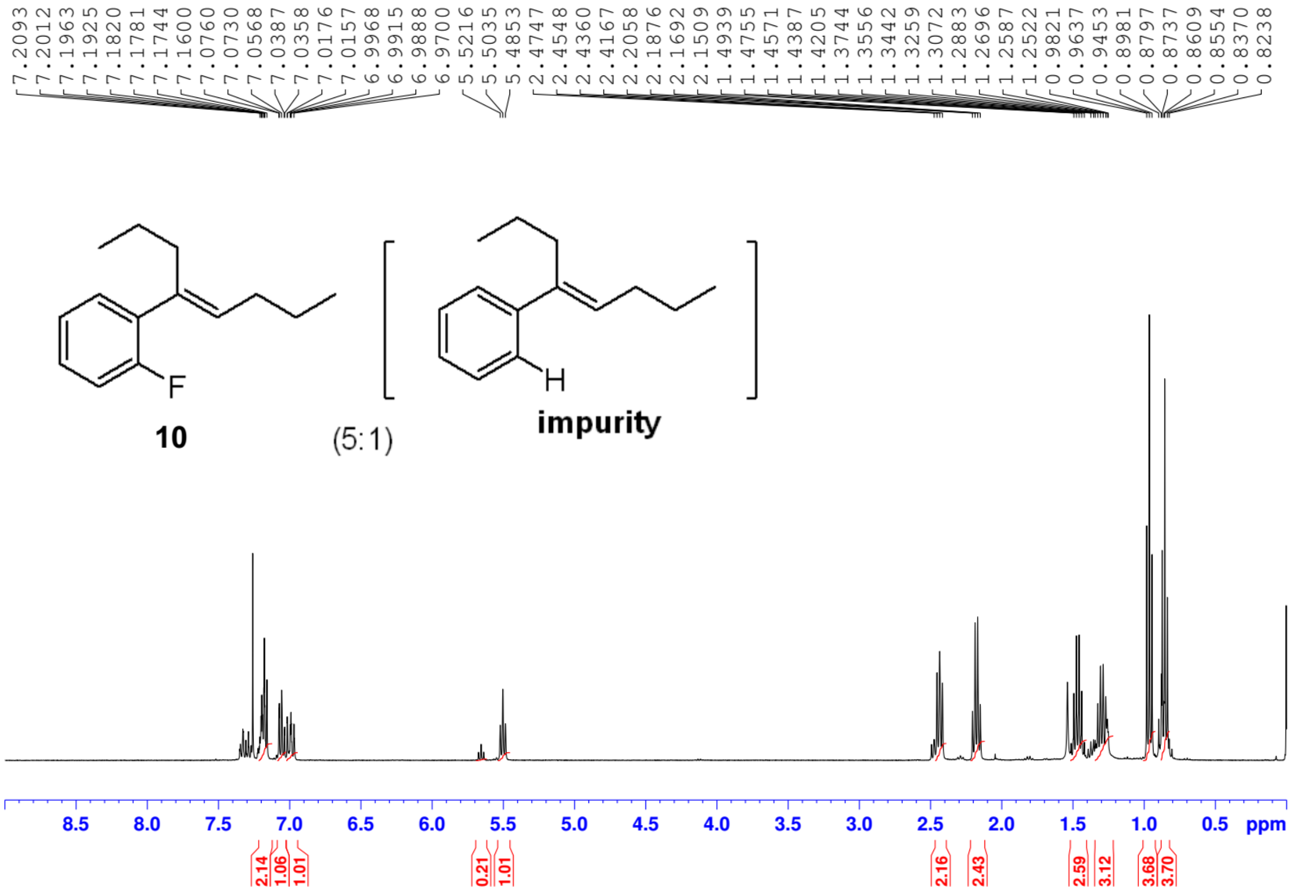
7.2093
7.2012
7.1963
7.1925
7.1820
7.1781
7.1744
7.1600
7.0760
7.0730
7.0568
7.0387
7.0358
7.0176
7.0157
6.9968
6.9915
6.9888
6.9700
5.5216
5.5035
5.4853
2.4747
2.4548
2.4360
2.4167
2.2058
2.1876
2.1692
2.1509
1.4939
1.4755
1.4571
1.4387
1.4205
1.3744
1.3556
1.3442
1.3259
1.3072
1.2883
1.2696
1.2587
1.2522
0.9821
0.9637
0.9453
0.8981
0.8797
0.8737
0.8609
0.8554
0.8370
0.8238
F
10
(5:1)
H
impurity
8.5
8.0
7.5
7.0
6.5
6.0
5.5
5.0
4.5
4.0
3.5
3.0
2.5
2.0
1.5
1.0
0.5
ppm
2.14
1.06
1.01
0.21
1.01
2.16
2.43
2.59
3.12
3.68
3.70

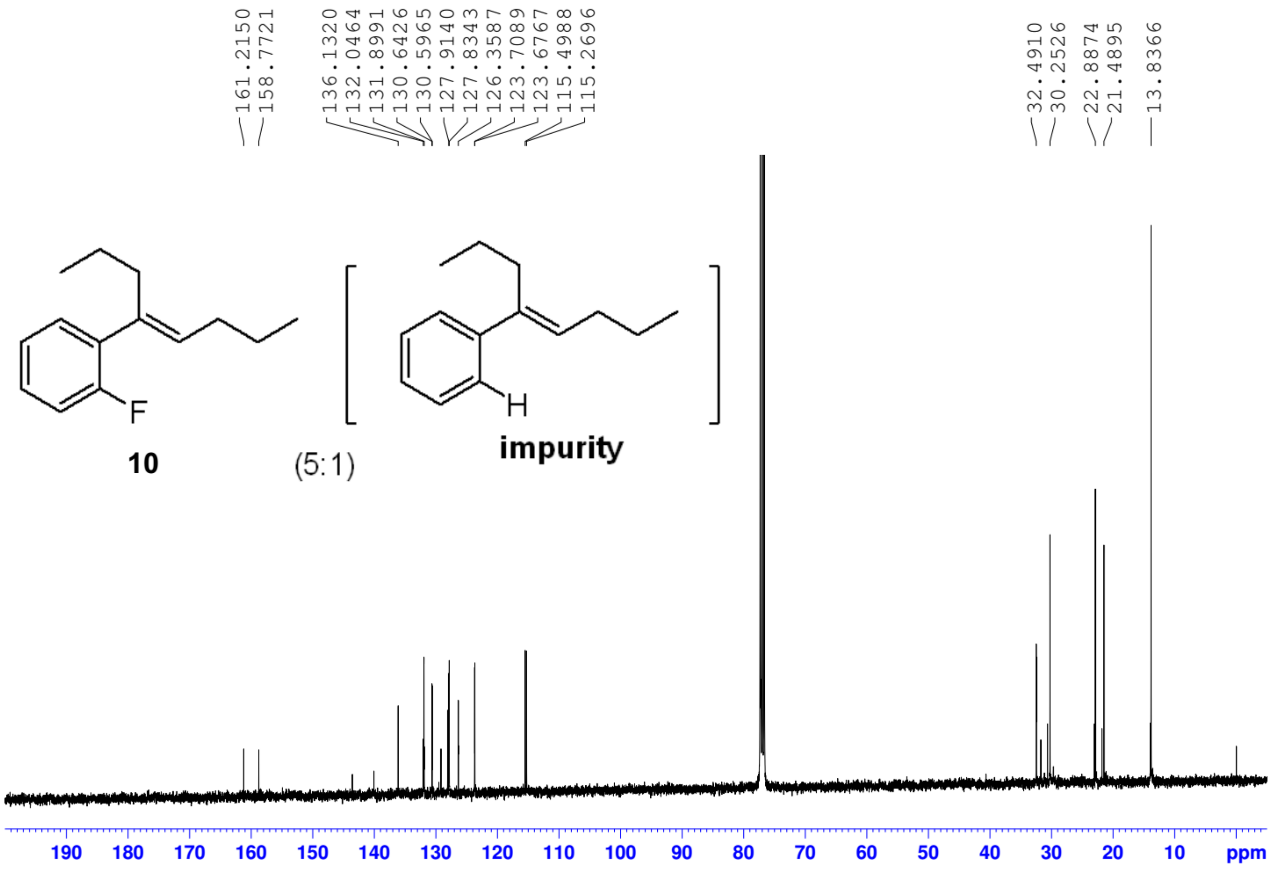
161.2150
158.7721
136.1320
132.0464
131.8991
130.6426
130.5965
127.9140
127.8343
126.3587
123.7089
123.6767
115.4988
115.2696
32.4910
30.2526
22.8874
21.4895
13.8366
F
10
(5:1)
H
impurity
190
180
170
160
150
140
130
120
110
100
90
80
70
60
50
40
30
20
10
ppm

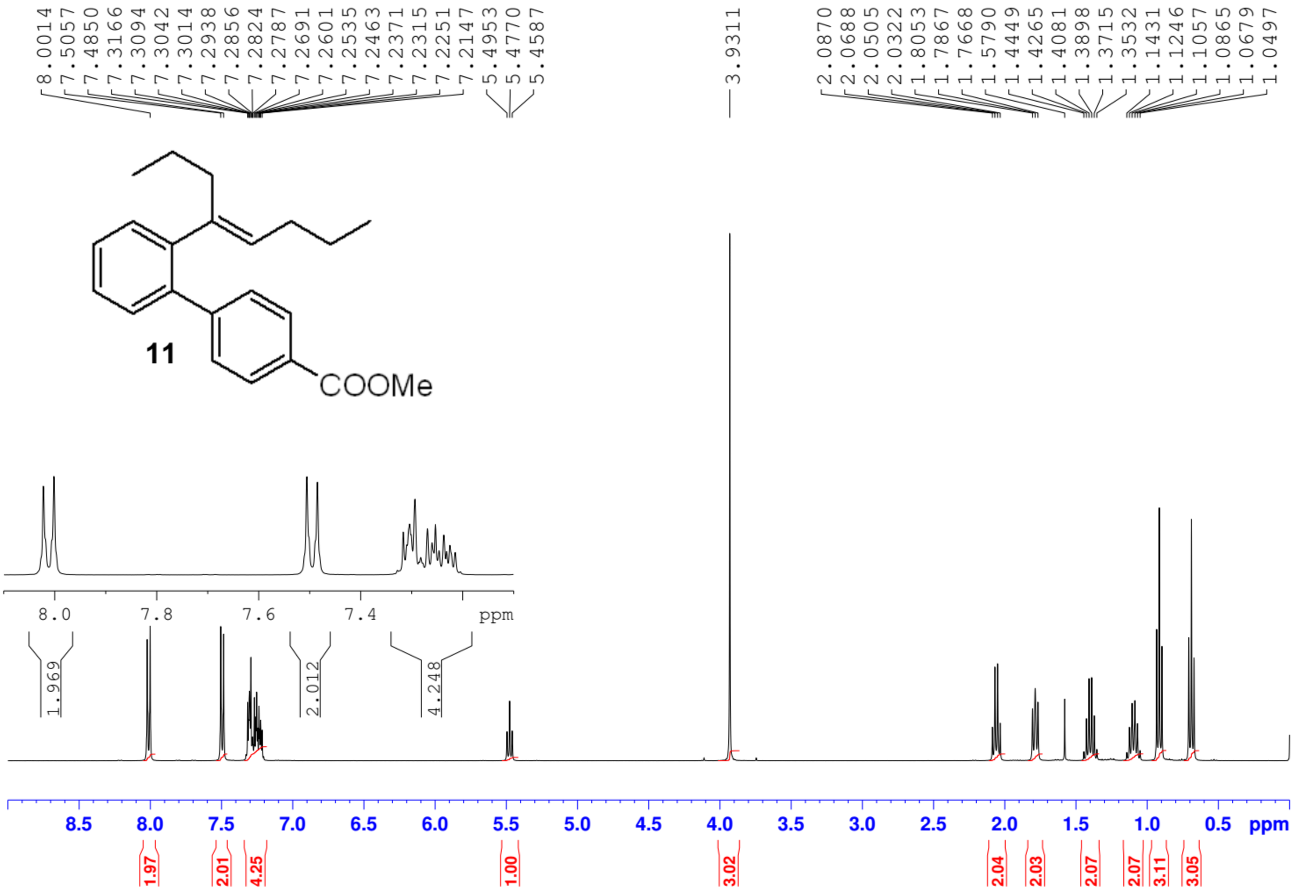
8.0014
7.5057
7.4850
7.3166
7.3094
7.3042
7.3014
7.2938
7.2856
7.2824
7.2787
7.2691
7.2601
7.2535
7.2463
7.2371
7.2315
7.2251
7.2147
5.4953
5.4770
5.4587
3.9311
2.0870
2.0688
2.0505
2.0322
1.8053
1.7867
1.7668
1.5790
1.4449
1.4265
1.4081
1.3898
1.3715
1.3532
1.1431
1.1246
1.1057
1.0865
1.0679
1.0497
11
COOMe
8.0
7.8
7.6
7.4
ppm
1.969
2.012
4.248
8.5
8.0
7.5
7.0
6.5
6.0
5.5
5.0
4.5
4.0
3.5
3.0
2.5
2.0
1.5
1.0
0.5
ppm
1.97
2.01
4.25
1.00
3.02
2.04
2.03
2.07
2.07
3.11
3.05

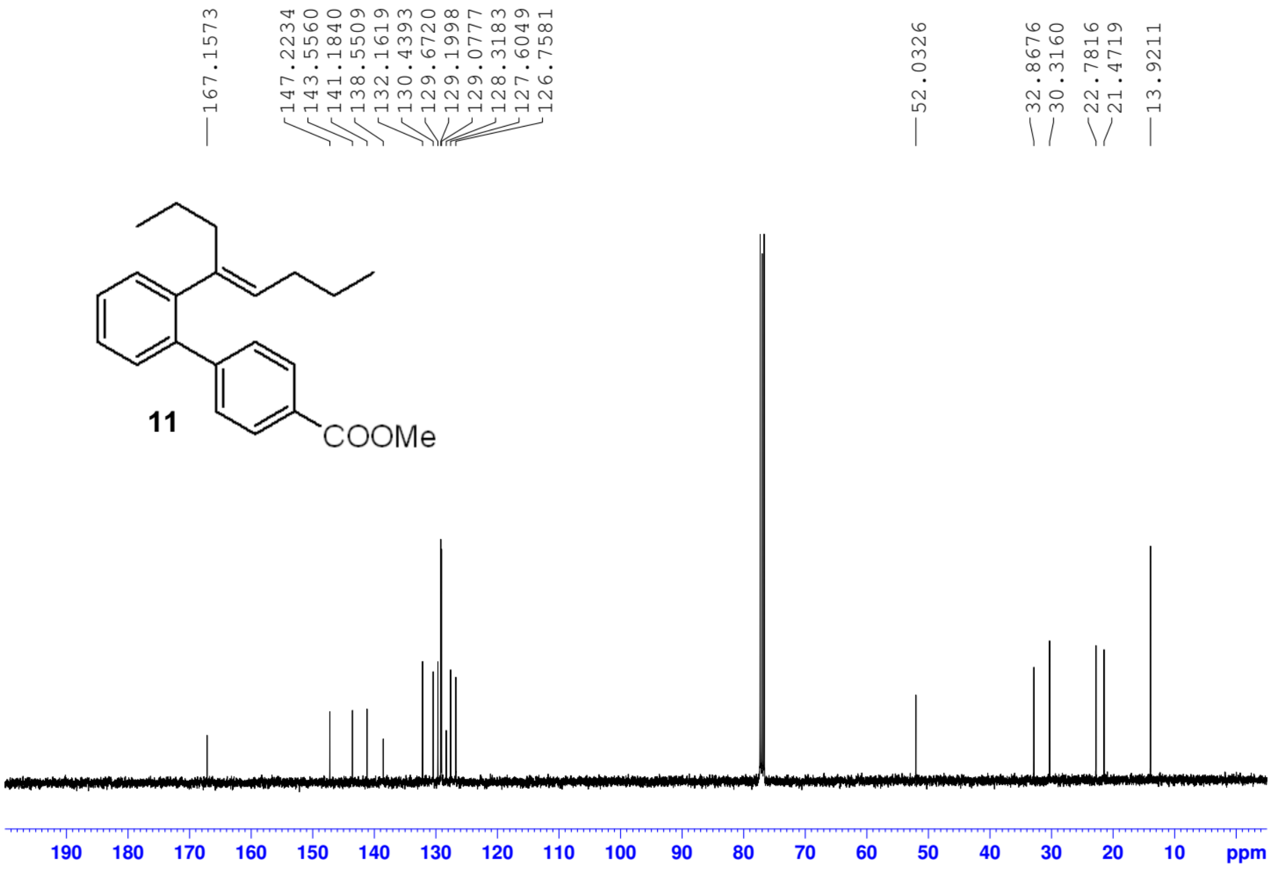
167.1573
147.2234
143.5560
141.1840
138.5509
132.1619
130.4393
129.6720
129.1998
129.0777
128.3183
127.6049
126.7581
52.0326
32.8676
30.3160
22.7816
21.4719
13.9211
11
COOMe
190
180
170
160
150
140
130
120
110
100
90
80
70
60
50
40
30
20
10
ppm

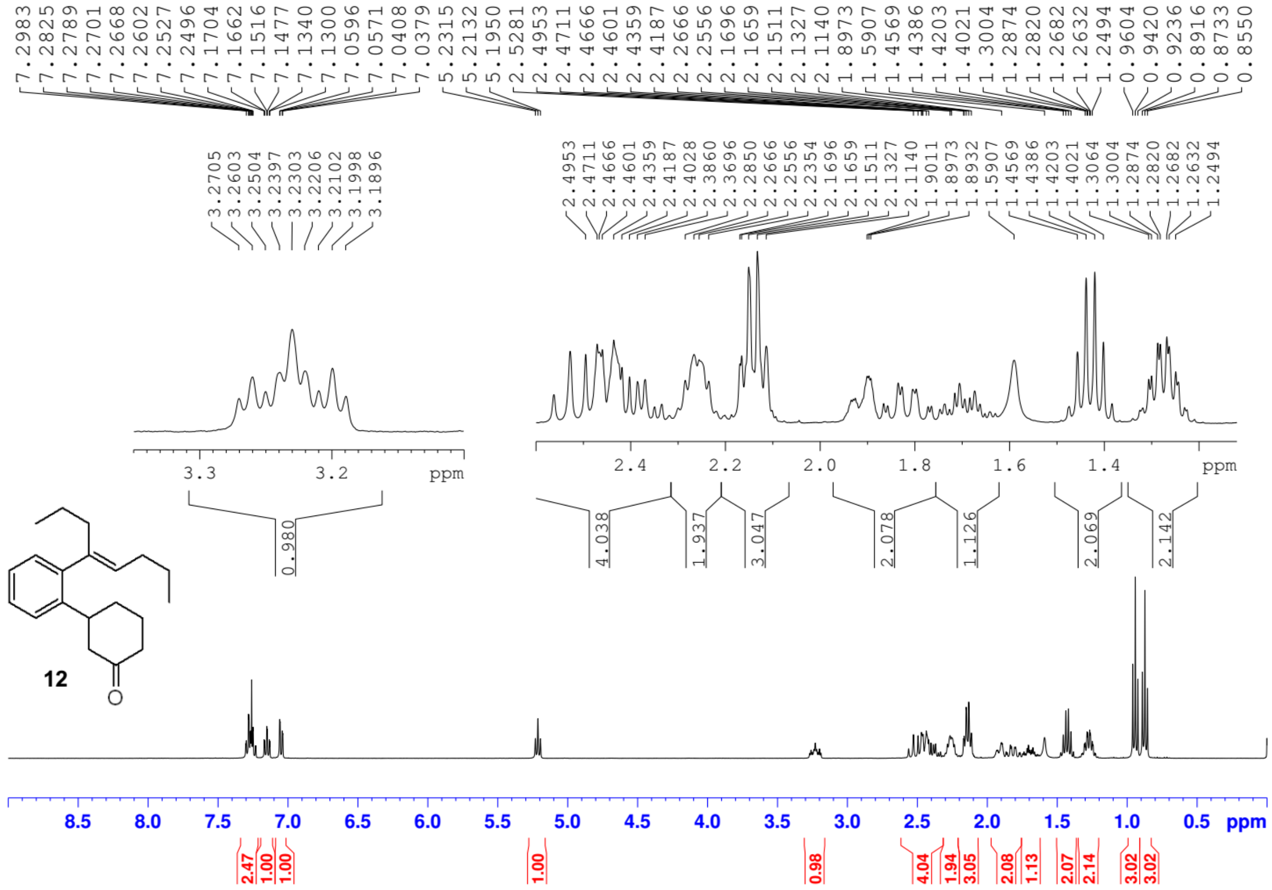

7.2983 7.2825 7.2789 7.2701 7.2668 7.2602 7.2527 7.2496 7.1704 7.1662 7.1516 7.1477 7.1340 7.1300 7.0596 7.0571 7.0408 7.0379 5.2315 5.2132 5.1950 2.5281 2.4953 2.4711 2.4666 2.4601 2.4359 2.4187 2.2666 2.2556 2.1696 2.1659 2.1511 2.1327 2.1140 1.8973 1.5907 1.4569 1.4386 1.4203 1.4021 1.3004 1.2874 1.2820 1.2682 1.2632 1.2494 0.9604 0.9420 0.9236 0.8916 0.8733 0.8550
3.2705 3.2603 3.2504 3.2397 3.2303 3.2206 3.2102 3.1998 3.1896
2.4953 2.4711 2.4666 2.4601 2.4359 2.4187 2.4028 2.3860 2.3696 2.2850 2.2666 2.2556 2.2354 2.1696 2.1659 2.1511 2.1327 2.1140 1.9011 1.8973 1.8932 1.5907 1.4569 1.4386 1.4203 1.4021 1.3064 1.3004 1.2874 1.2820 1.2682 1.2632 1.2494
3.3 3.2 ppm
0.980
2.4 2.2 2.0 1.8 1.6 1.4 ppm
4.038 1.937 3.047 2.078 1.126 2.069 2.142
12
O
8.5 8.0 7.5 7.0 6.5 6.0 5.5 5.0 4.5 4.0 3.5 3.0 2.5 2.0 1.5 1.0 0.5 ppm
2.47 1.00 1.00 1.00 0.98 4.04 1.94 3.05 2.08 1.13 2.07 2.14 3.02 3.02

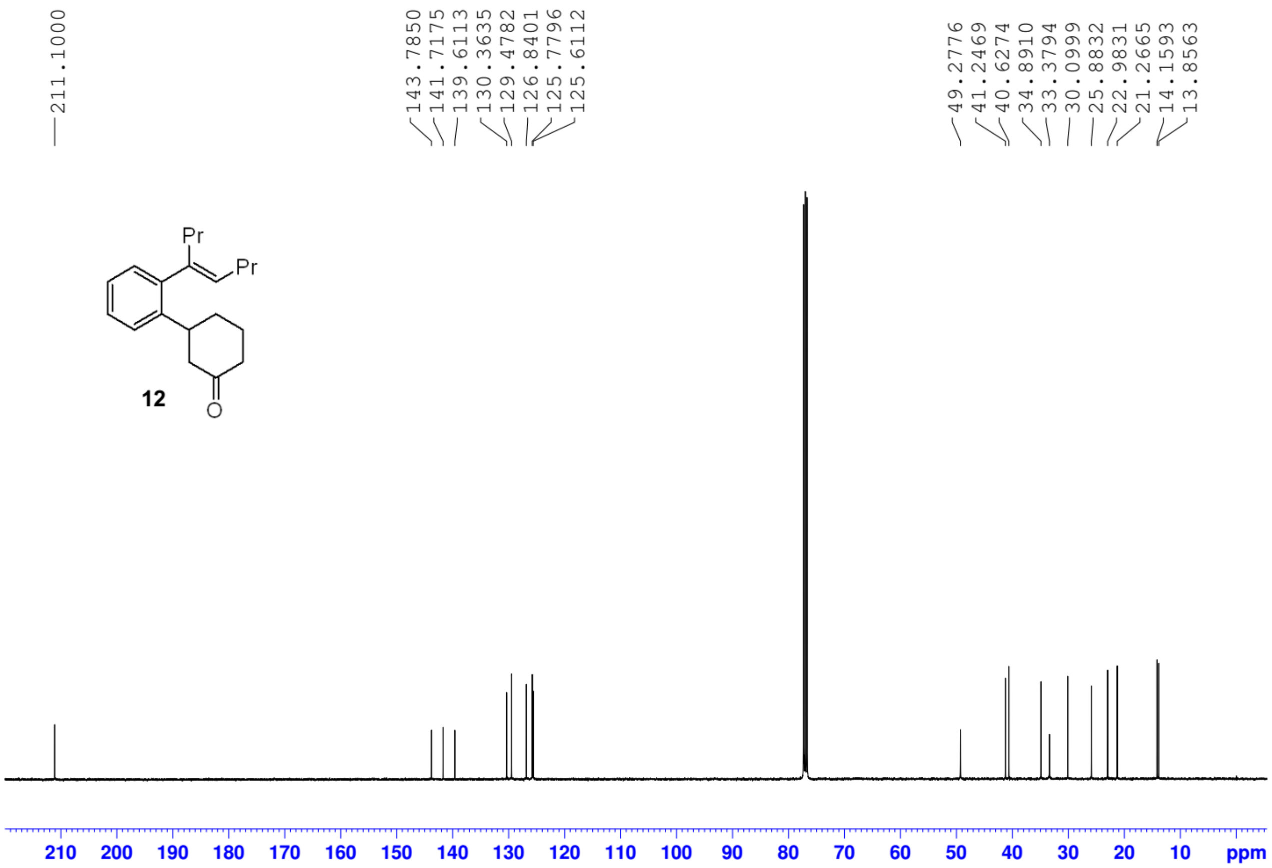

211.1000
143.7850 141.7175 139.6113 130.3635 129.4782 126.8401 125.7796 125.6112
49.2776 41.2469 40.6274 34.8910 33.3794 30.0999 25.8832 22.9831 21.2665 14.1593 13.8563
Pr
Pr
12
O
210 200 190 180 170 160 150 140 130 120 110 100 90 80 70 60 50 40 30 20 10 ppm
